# Design, Synthesis, and Biological Evaluation of Novel Dithiocarbamate-Based Human Carbonic Anhydrase IX/XII Inhibitors as Potential Anti-Glioblastoma Agents

**DOI:** 10.3390/molecules31203593

**Published:** 2026-10-09

**Authors:** Mehlika Dilek Altıntop, Hayrunnisa Nadaroglu, Fatma Yesilyurt, Nalan Yılmaz Savaş, Ruken Avsaroglu, Azize Alaylı, Kaan Küçükoğlu, Ahmet Hacimuftuoglu, Ahmet Özdemir

**Affiliations:** 1 Department of Pharmaceutical Chemistry, Faculty of Pharmacy, Anadolu University, 26470 Eskişehir, Türkiye; ahmeto@anadolu.edu.tr; 2 Department of Food Technology, Vocational College of Technical Science, Ataturk University, 25240 Erzurum, Türkiye; hnisa25@atauni.edu.tr; 3 Department of Nanoscience and Nanoengineering, Institute of Natural and Applied Sciences, Ataturk University, 25240 Erzurum, Türkiye; 4 Department of Pharmacology, Faculty of Dentistry, Atatürk University, 25240 Erzurum, Türkiye; fatmayesilyurt@atauni.edu.tr; 5 Vocational School of Health Services, Department of Pharmacy Services, Ataturk University, 25240 Erzurum, Türkiye; 6 Department of Pharmaceutical Chemistry, Graduate School, Anadolu University, 26470 Eskişehir, Türkiye; nalan_y@anadolu.edu.tr; 7 Department of Medical Pharmacology, Faculty of Medicine, Ataturk University, 25240 Erzurum, Türkiye; rukenavsaroglu@gmail.com (R.A.); ahmeth@atauni.edu.tr (A.H.); 8 Department of Nursing, Faculty of Health Sciences, Sakarya University of Applied Sciences, 54187 Sakarya, Türkiye; aalayli@subu.edu.tr; 9 Department of Pharmaceutical Chemistry, Faculty of Pharmacy, Selçuk University, 42130 Konya, Türkiye; kaan.kucukoglu@selcuk.edu.tr

**Keywords:** carbonic anhydrase IX, carbonic anhydrase XII, cell death, cytotoxic agents, dithiocarbamates, glioblastoma, inflammation, oxidative stress

## Abstract

To develop human carbonic anhydrase (hCA) IX/XII-targeted therapeutics for glioblastoma multiforme (GBM), novel dithiocarbamate (DTC)-based compounds were synthesized and evaluated for their inhibitory activity against hCA I, II, IX, and XII isoforms. The most potent hCA IX/XII inhibitors were further investigated for their cytotoxic activity against U87 and T98 glioblastoma cells, selectivity toward healthy neuronal cells, and their effects on oxidative stress, inflammatory mediators, and cell death-related pathways. Compounds **4**, **9**, and **30** emerged as the most promising candidates, with compounds **9** and **30** exhibiting potent cytotoxic activity against both U87 and T98 cells. These compounds acted as dual hCA IX/XII inhibitors with a clear preference for hCA XII. Compounds **9** and **30** modulated the oxidative stress–inflammation axis through regulation of cellular redox balance, and inflammation-associated mediator profiles, including IL-1β, IL-10, TNF-α, and TGF-β. Compound **4** also attenuated oxidative stress and inflammatory signaling in U87 cells. Molecular docking studies supported the experimental findings by revealing favorable binding interactions within the active sites of hCA IX and XII, providing a structural basis for their inhibitory activities. Collectively, these findings identify compounds **4**, **9**, and **30** as promising DTC-based compounds for GBM therapy through the combined modulation of tumor-associated CAs, oxidative stress, inflammatory, and cell death-related pathways.

## 1. Introduction

**Figure 1:**
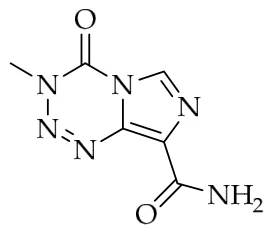
TMZ.

GBM is considered the most malignant form of infiltrating brain cancers and is one of the most aggressive human malignancies [1,2]. GBM remains difficult to treat because of its high mitotic index, hallmark features such as diffuse infiltration into normal brain tissues, angiogenesis, genomic aberrations, resistance to apoptosis, and presence of necrotic areas [3,4,5]. Current treatment protocol involves surgery followed by radiotherapy and concomitant and adjuvant chemotherapy with temozolomide (TMZ) (Figure 1) [2]. Despite considerable efforts to improve therapeutic strategies for GBM, the prognosis of patients with GBM remains poor, with a median overall survival of approximately 15 months [6].

Chemotherapy plays a pivotal role in the standard-of-care treatment for GBM. However, its efficacy is limited by intrinsic and acquired resistance, as well as restricted drug delivery across the blood–brain barrier (BBB) and the blood–brain tumor barrier (BBTB) [2]. Furthermore, clinical applications are hampered by adverse effects, including bone marrow suppression and genotoxic, teratogenic, and fetotoxic effects [7]. The limitations of current chemotherapy for GBM have driven the development of targeted therapies that target specific molecular pathways involved in glioma pathogenesis [8,9].

Carbonic anhydrases (CAs; EC 4.2.1.1) are zinc-containing metalloenzymes found in both prokaryotes and eukaryotes and are encoded by eight genetically distinct families: α-, β-, γ-, δ-, ζ-, η-, θ-, and ι-CAs [10,11]. CAs, which catalyze the reversible hydration of carbon dioxide into bicarbonate and protons (CO_2_ + H_2_O ⇌ HCO_3_^−^ + H^+^), are important targets in drug discovery due to their crucial roles in various physiological and pathological processes, including CO_2_/bicarbonate transport between tissues and the lungs, respiration, pH and CO_2_ homeostasis, electrolyte secretion in various tissues and organs, biosynthetic processes (e.g., gluconeogenesis, lipogenesis, and ureagenesis), bone resorption, calcification, and tumorigenesis [12]. CA inhibitors have been clinically utilized as diuretics, antiglaucoma agents, and antiepileptic drugs. Beyond these conventional applications, recent advances have highlighted their emerging potential in the treatment of cancer, neuropathic pain, sleep apnea, migraine, and cerebral ischemia [10,11,12].

The majority of hCA isoenzymes represent important therapeutic targets owing to their potential to be modulated for the treatment of several diseases. Studies have revealed that hCA I contributes to the pathogenesis of retinal and cerebral edema, indicating that its inhibition may provide a promising therapeutic approach for alleviating these conditions. Furthermore, hCA II has been associated with various pathological disorders, including glaucoma, edema, epilepsy, and altitude sickness, highlighting its significance as a potential drug target [13].

Tumor-associated transmembrane hCA isoenzymes, hCA IX and XII, are key pH regulators that create a distinct pH microenvironment in hypoxic solid tumors and enable tumor cell survival under stressful conditions. The overexpression of these enzymes contributes to tumor progression by promoting tumor growth, activating the metastatic cascade, and reducing the efficacy of chemotherapy. Consequently, the inhibition of hCA IX and XII isoenzymes has emerged as a promising therapeutic strategy for cancer treatment. The therapeutic relevance of these enzymes stems from the presence of a targetable, extracellular catalytic domain in both isoforms, making them attractive drug targets. Consequently, charged, membrane-impermeable compounds can be designed to selectively inhibit hCA IX and XII while minimizing undesirable off-target interactions with cytosolic and mitochondrial CA isoforms [14,15].

hCA IX is significantly overexpressed in many types of tumors, including GBM, whereas its expression in normal tissues is restricted. This differential expression profile is expected to reduce off-target toxicity associated with hCA inhibition and supports the development of hCA IX inhibitors as a promising strategy for targeted anticancer therapy [15,16,17,18]. Accumulating evidence has demonstrated that hCA XII is also a clinically significant, molecular tumor subtype-specific therapeutic target in GBM with the potential to suppress the invasion of brain tumor cells [19]. Moreover, hCA II expression has been reported in several types of brain tumors, including gliomas [20,21].

DTCs are considered versatile pharmacophores and privileged structural motifs due to their ability to engage in diverse interactions with biological targets [22,23]. The sulfur atom of the DTC group, characterized by its strong nucleophilicity and distinctive redox properties, plays a pivotal role in enzyme catalysis, protein folding, and redox signaling and regulation [24]. In particular, DTCs have emerged as a promising class of hCA inhibitors, exhibiting potent inhibitory activity against several hCA isoforms, including hCA I, II, IX, and XII [22,23,24,25,26,27,28]. Furthermore, numerous studies have demonstrated that DTCs possess marked anticancer activity against various malignancies, including gliomas, highlighting their potential as promising anticancer agents [28,29,30].

To the best of our knowledge, DTC derivatives possessing both anti-GBM activity and hCA IX and/or XII inhibitory activity have not yet been reported. In this paper, in the pursuit of hCA-targeted cytotoxic agents for the treatment of GBM, new DTC-based compounds were synthesized and evaluated for their inhibitory activities against hCA isoforms (I, II, IX, and XII). The most potent hCA IX and/or XII inhibitors were assessed for their cytotoxic effects on human glioblastoma cell lines (U87 and T98) and healthy neuronal cells. DTC derivatives possessing both anti-GBM activity and hCA IX and/or XII inhibitory activity were also investigated for their effects on oxidative stress, inflammation, and cell death-related pathways in U87 and T98 cells. Molecular docking studies were carried out to explore the possible binding modes of the most promising hCA IX and/or XII inhibitors. Moreover, the most active compounds, identified based on their promising hCA-targeted cytotoxic activity against GBM cells, were further subjected to in silico pharmacokinetic evaluation.

## 2. Results

The synthesis of the hitherto unreported DTCs (**1–44**) is depicted in Figure 2. Sodium 4-alkylpiperazine-1-carbodithioate derivatives were obtained by the reaction of 1-alkylpiperazine with carbon disulfide in the presence of sodium hydroxide. 2-Chloro-*N*-(thiazol/benzothiazol-2-yl)acetamide derivatives were synthesized via the reaction of 2-aminothiazoles/2-aminobenzothiazoles with chloroacetyl chloride in the presence of triethylamine (TEA). The reaction of 2-chloro-*N*-(thiazol/benzothiazol-2-yl)acetamide derivatives with sodium 4-alkylpiperazine-1-carbodithioate derivatives afforded compounds **1–44**. The chemical structures of all compounds were verified by Infrared (IR), Nuclear Magnetic Resonance (NMR, ^1^H and ^13^C), and High Resolution Mass Spectrometry (HRMS) data (Figures S1–S132).

The inhibitory activities of compounds **1–44** against hCA I, II, IX, and XII isoforms were investigated, with acetazolamide (AAZ), a well-known hCA inhibitor, serving as the positive control (Table 1 and Table 2).

Among these compounds, compounds **12** (IC_50_ = 3.26 µM) and **42** (IC_50_ = 3.27 µM) were identified as the most potent hCA I inhibitors. Compound **12** was found to inhibit the hCA I enzyme competitively (*K*_i_ = 1.63 µM), whereas compound **42** exhibited a noncompetitive inhibition pattern against hCA I (*K*_i_ = 1.25 µM).

Among the tested compounds, compounds **37** (IC_50_ = 2.76 µM) and **40** (IC_50_ = 2.35 µM) were identified as the most potent hCA II inhibitors. Kinetic studies demonstrated that both compounds inhibited the hCA II enzyme with a competitive inhibition pattern. The *K*_i_ values of compounds **37** and **40** were detected as 1.14 and 1.11 µM, respectively.

The inhibitory effects of compounds **1–44** against the tumor-associated transmembrane hCA IX and XII isoforms are presented in Table 2. Most of the compounds were found to exhibit inhibitory activity against hCA IX and/or XII. Compounds **14** (IC_50_ = 34.06 nM), **20** (IC_50_ = 14.72 nM), **21** (IC_50_ = 14.70 nM), **36** (IC_50_ = 32.17 nM), and **39** (IC_50_ = 28.15 nM) showed stronger inhibitory activity against hCA IX than AAZ (IC_50_ = 35.26 nM), whereas compound **41** (IC_50_ = 35.62 nM) exhibited hCA IX inhibitory activity comparable to that of AAZ. These compounds were determined to inhibit the hCA IX enzyme through a competitive inhibition pattern.

Compounds **4**, **9**, **15**, **16**, **17**, **18**, **20**, **29**, and **30** were found to exhibit stronger inhibitory activity against hCA XII with the IC_50_ values of 21.41, 20.95, 16.29, 29.84, 12.01, 20.85, 17.61, 30.04, and 29.83 nM, respectively than AAZ (IC_50_ = 30.13 nM). These compounds were determined to inhibit the hCA XII enzyme through a competitive inhibition pattern.

The cytotoxic effects of the most potent hCA IX and/or XII inhibitors on T98 and U87 human glioblastoma cells were evaluated at 24, 48, and 72 h using the MTT assay (Table 3). Compounds **9**, **17**, and **30** were found as the most potent cytotoxic agents against the T98 cell line at 72 h with the IC_50_ values of 69.69, 96.62, and 57.71 µM, respectively.

The tested compounds were found to exhibit stronger cytotoxic effects against the U87 cell line. The IC_50_ values of compound **4** against U87 cells at 24, 48, and 72 h were determined to be 33.85, 35.56, and 3.86 µM, respectively. The IC_50_ values of compound **9** against the U87 cell line at 24, 48, and 72 h were determined to be 74.26, 35.64, and 120.80 µM, respectively. The initial decrease in IC_50_ at 48 h followed by an increase at 72 h indicates a time-dependent fluctuation in the cytotoxic activity of compound **9**. This variation may reflect differential cellular responses to prolonged exposure, including the activation of adaptive or compensatory survival mechanisms. The cytotoxic effect of compound **14** against the U87 cell line increased in a time-dependent manner, and its IC_50_ value at 72 h was determined to be 41.73 µM. The cytotoxic effect of compound **15** against the U87 cell line also enhanced in a time-dependent manner, and the IC_50_ values at 48 and 72 h were determined to be 73.19 and 4.79 µM, respectively. The IC_50_ values of compounds **17** and **23** against the U87 cell line at 72 h were determined to be 87.39 and 20.33 µM, respectively. The IC_50_ values of compound **30** against the U87 cell line at 24, 48, and 72 h were determined to be 61.28, 17.76, and 19.51 µM, respectively.

An interesting observation from the in vitro cytotoxicity assays was the time-dependent fluctuation in the cytotoxic activity of compound **20** against T98 glioblastoma cells. Notably, the cytotoxic activity of compound **20** against T98 cells increased from 24 to 48 h, as evidenced by a decrease in the IC_50_ value from 539.70 µM at 24 h to 261.10 µM at 48 h. However, at 72 h, the IC_50_ value increased markedly to 548.00 µM, indicating a reduction in cytotoxic activity compared with that observed at 48 h (Table 3). This biphasic response may reflect the complex and dynamic nature of cellular responses to prolonged compound exposure. One possible explanation is that prolonged incubation may lead to partial degradation or reduced stability of the compound in the culture medium, thereby decreasing the amount of biologically active compound available at later time points. Alternatively, glioblastoma cells may activate adaptive survival responses following prolonged cytotoxic stress. Such responses may include the upregulation of drug efflux mechanisms and/or activation of pro-survival pathways, such as autophagy, which could transiently reduce cellular sensitivity to the compound at later stages of treatment. Although these mechanisms were not directly investigated in the present study, they provide plausible explanations for the observed increase in the IC_50_ value of compound **20** at 72 h and warrant further investigation.

**Figure 3:**
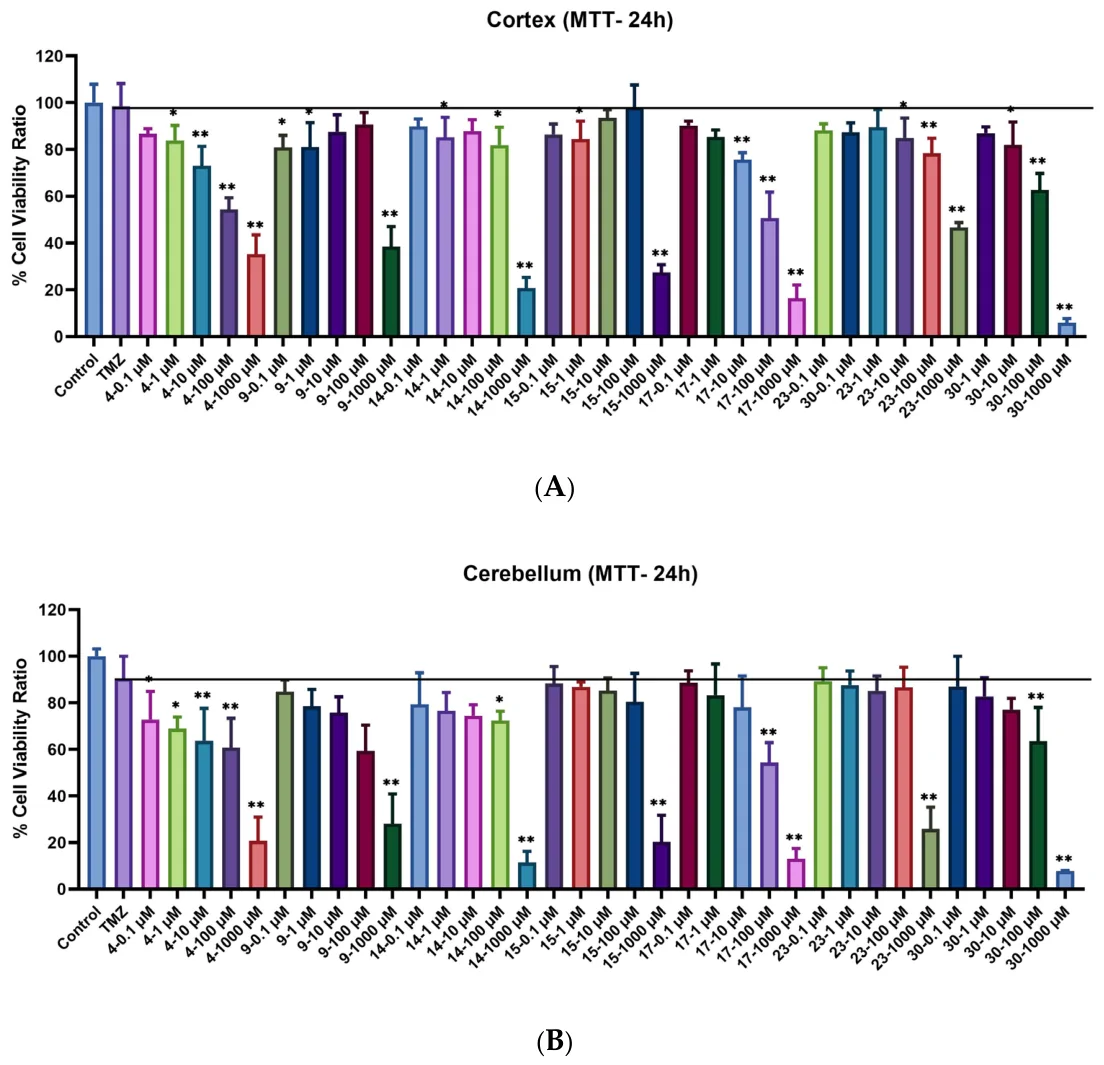
Cytotoxic effects of compounds **4**, **9**, **14**, **15**, **17**, **23**, and **30** at various concentrations on the cortex (**A**) and cerebellum (**B**) after 24 h. * *p* < 0.05, ** *p* < 0.001. The horizontal line represents the baseline cell viability of cells treated with the positive control, TMZ, for comparison.

The results of the primary neuron culture experiment are presented in Figure 3, which illustrates the cytotoxic effects of compounds **4**, **9**, **14**, **15**, **17**, **23**, and **30** at various concentrations. Compounds **4**, **9**, **14**, **15**, **17**, **23**, and **30** did not exhibit cytotoxic effects on cortical and cerebellar cells cultured from neonatal rat brain tissue. Among the tested compounds, cytotoxicity was observed only at higher concentrations.

To further characterize the selectivity profile of the most promising cytotoxic agents, the selectivity index (SI) values were calculated for compounds **4**, **9** and **30** (Table 4). The SI was defined as the ratio of the IC_50_ value in primary healthy cultures to that in glioblastoma cells. Given that the IC_50_ values for normal primary cells (cerebellum and cortex) were >1000 µM, the calculated SI values indicate a favorable selectivity profile, particularly for compounds **4** and **30** at 72 h in U87 cells.

To elucidate the effects of the compounds on cancer-associated oxidative stress, inflammation, and cell death mechanisms, their effects on total oxidative status (TOS), total antioxidant status (TAS), interleukin-1 beta (IL-1β), interleukin-10 (IL-10), tumor necrosis factor-alpha (TNF-α), and transforming growth factor-beta (TGF-β) levels were evaluated.

**Figure 4:**
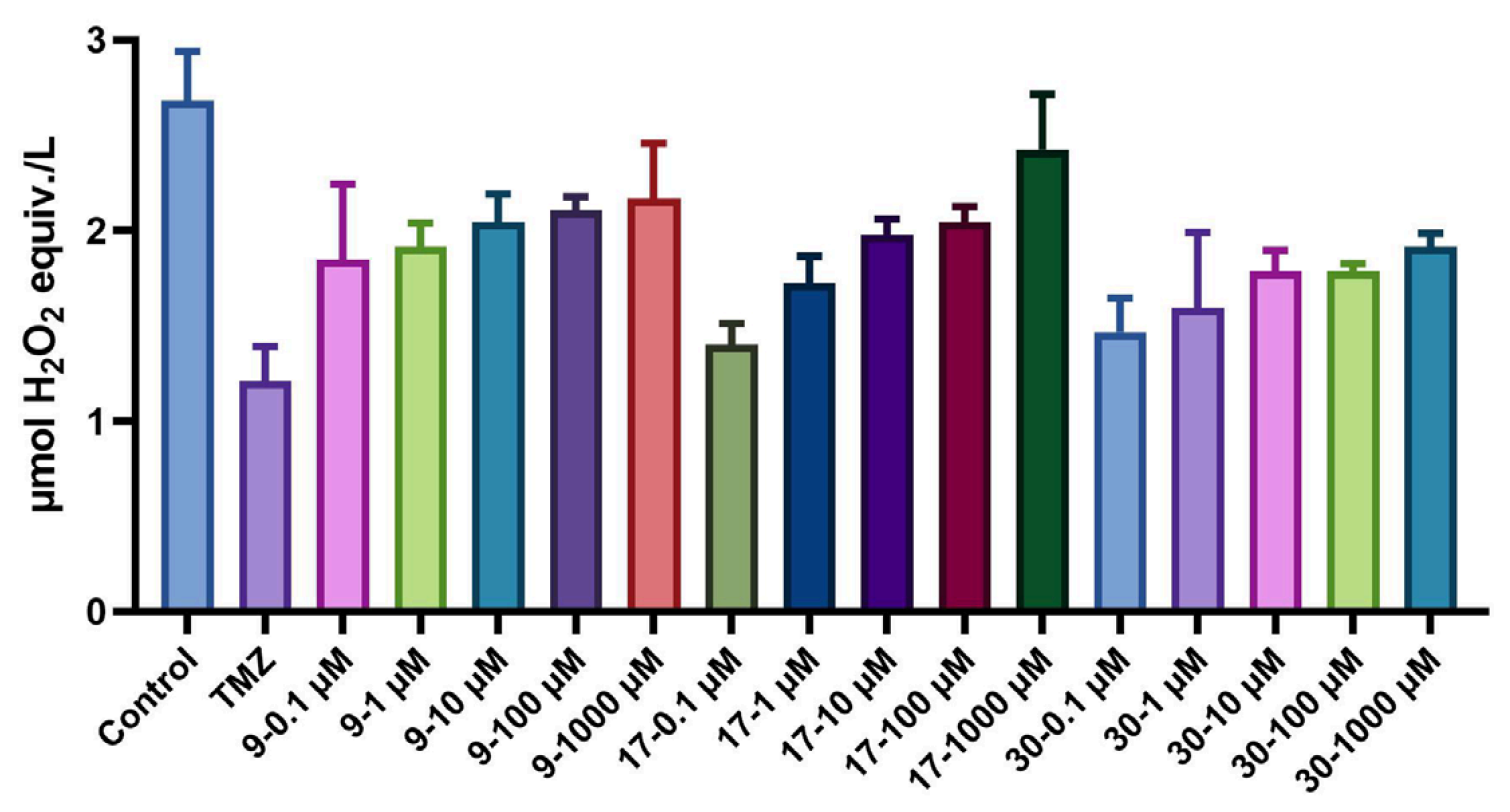
Effects of compounds **9**, **17**, and **30** at various concentrations on TOS levels in T98 cells after 72 h.

Compared with the control group, compound **30** was found to decrease TOS levels in T98 cells at all tested concentrations, whereas compound **17** was determined to be particularly effective at a concentration of 0.1 µM (Figure 4).

**Figure 5:**
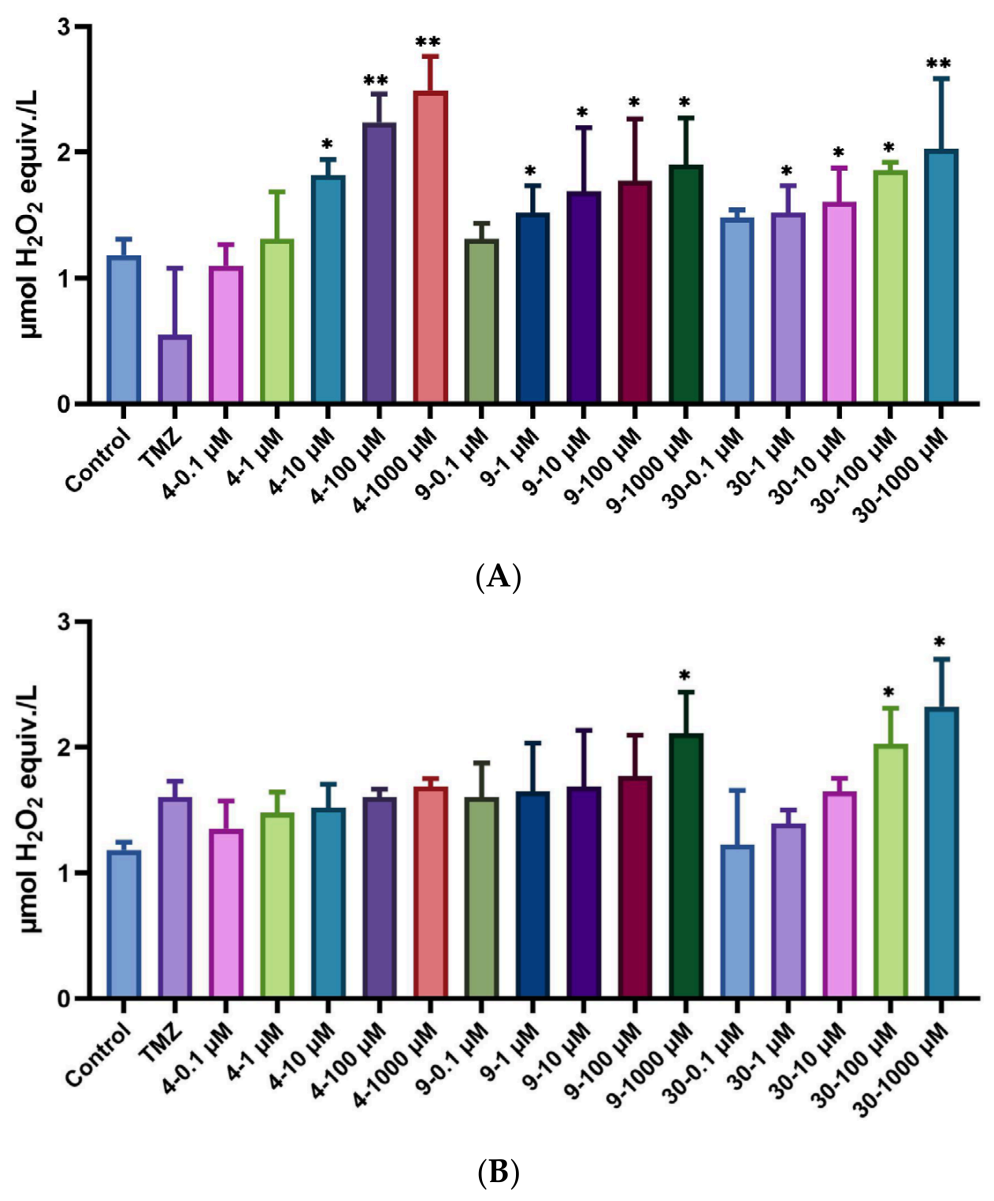
Effects of compounds **4**, **9**, and **30** at various concentrations on TOS levels in U87 cells after 24 (**A**) and 48 (**B**) hours. * *p* < 0.05, ** *p* < 0.001.

**Figure 6:**
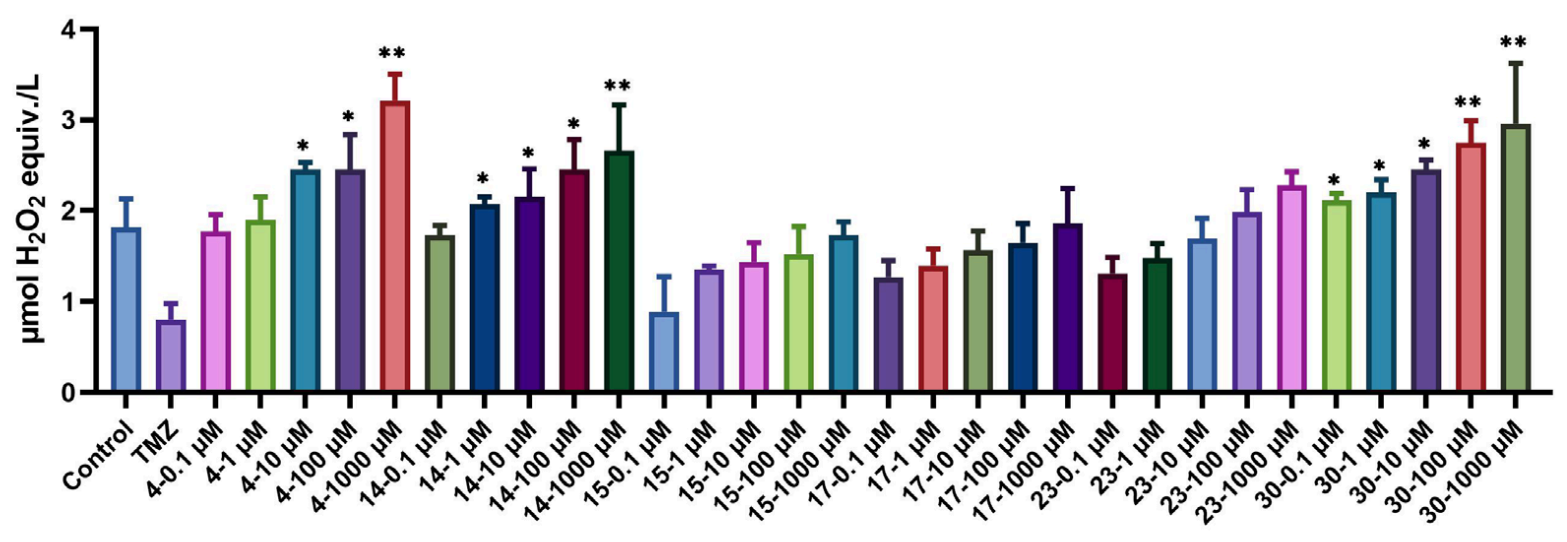
Effects of compounds **4**, **14**, **15**, **17**, **23**, and **30** at various concentrations on TOS levels in U87 cells after 72 h. * *p* < 0.05, ** *p* < 0.001.

Compared with the control group, the 24-h results showed that compound **4** decreased TOS levels in U87 cells at a concentration of 0.1 µM (Figure 5). According to the 72-h results, compound **15** reduced TOS levels in U87 cells at a concentration of 0.1 µM, similarly to TMZ (Figure 6).

**Figure 7:**
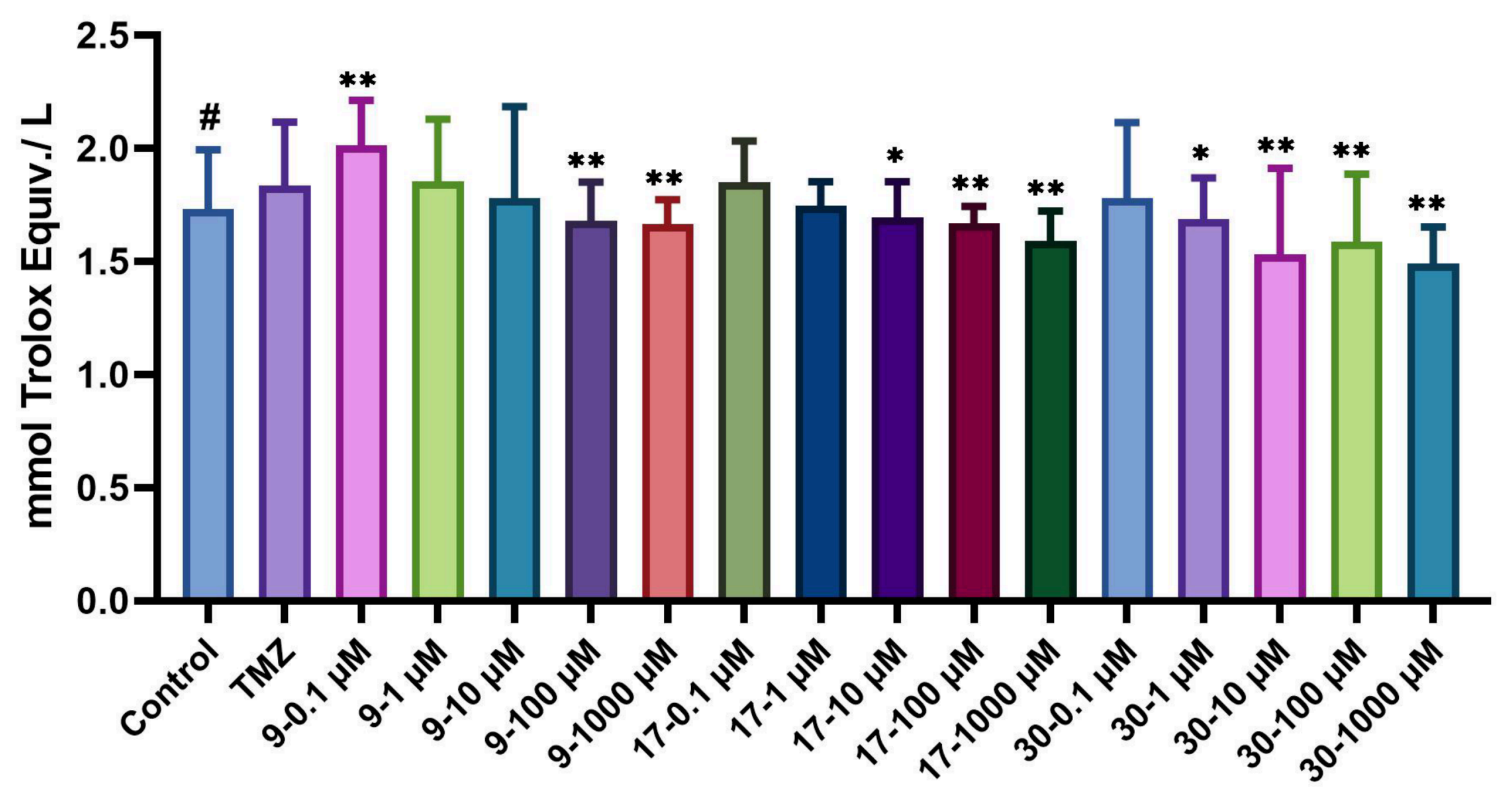
Effects of compounds **9**, **17**, and **30** at various concentrations on TAS levels in T98 cells after 72 h. # Comparison between TMZ and control groups, *p* < 0.05; * *p* < 0.05, ** *p* < 0.001.

Compared with the control group, compound **9** was found to significantly increase TAS levels in T98 cells, particularly at a concentration of 0.1 µM (*p* < 0.001). Compounds **17** and **30** were also determined to increase TAS levels in T98 cells at a concentration of 0.1 µM (Figure 7).

**Figure 8:**
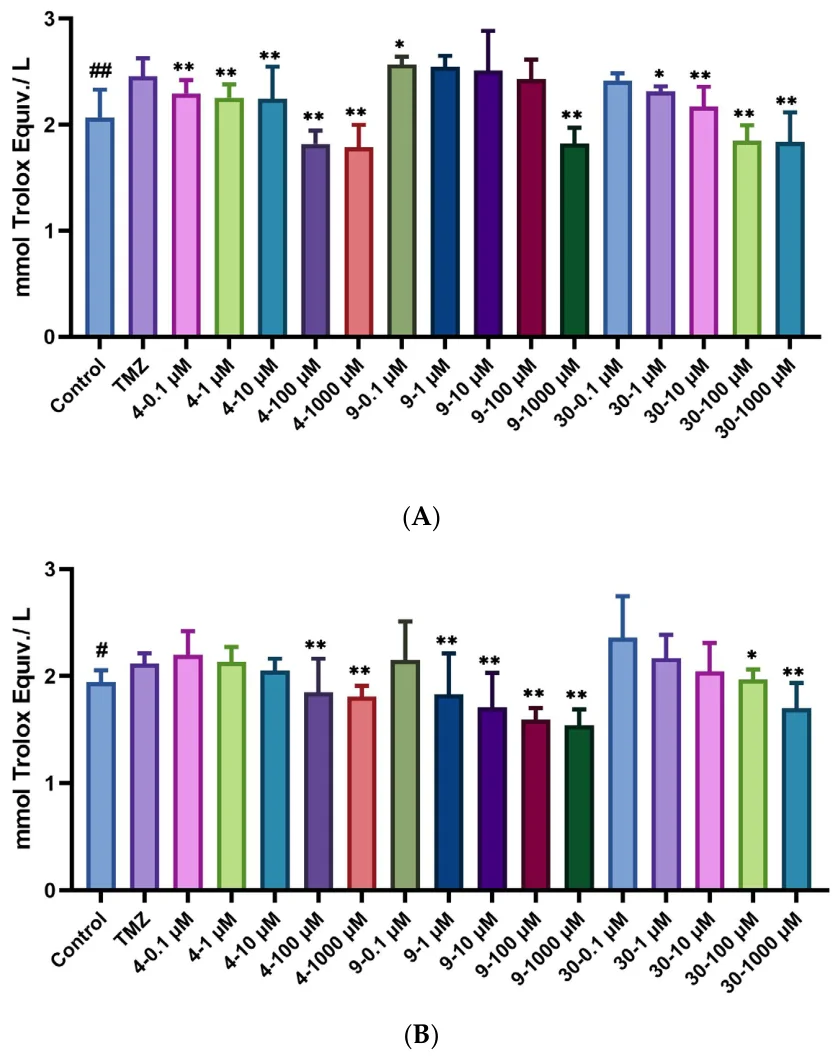
Effects of compounds **4**, **9**, and **30** at various concentrations on TAS levels in U87 cells after 24 (**A**) and 48 (**B**) hours. # Comparison between TMZ and control groups, *p* < 0.05; ## comparison between TMZ and control groups, *p* < 0.001; * *p* < 0.05, ** *p* < 0.001.

Compared with the control group, the 24-h results showed that compound **9** significantly increased TAS levels in U87 cells, particularly at a concentration of 0.1 µM (*p* < 0.05) (Figure 8). According to the 48-h results, compounds **4**, **9**, and **30** increased TAS levels in U87 cells at a concentration of 0.1 µM compared with the control group.

**Figure 9:**
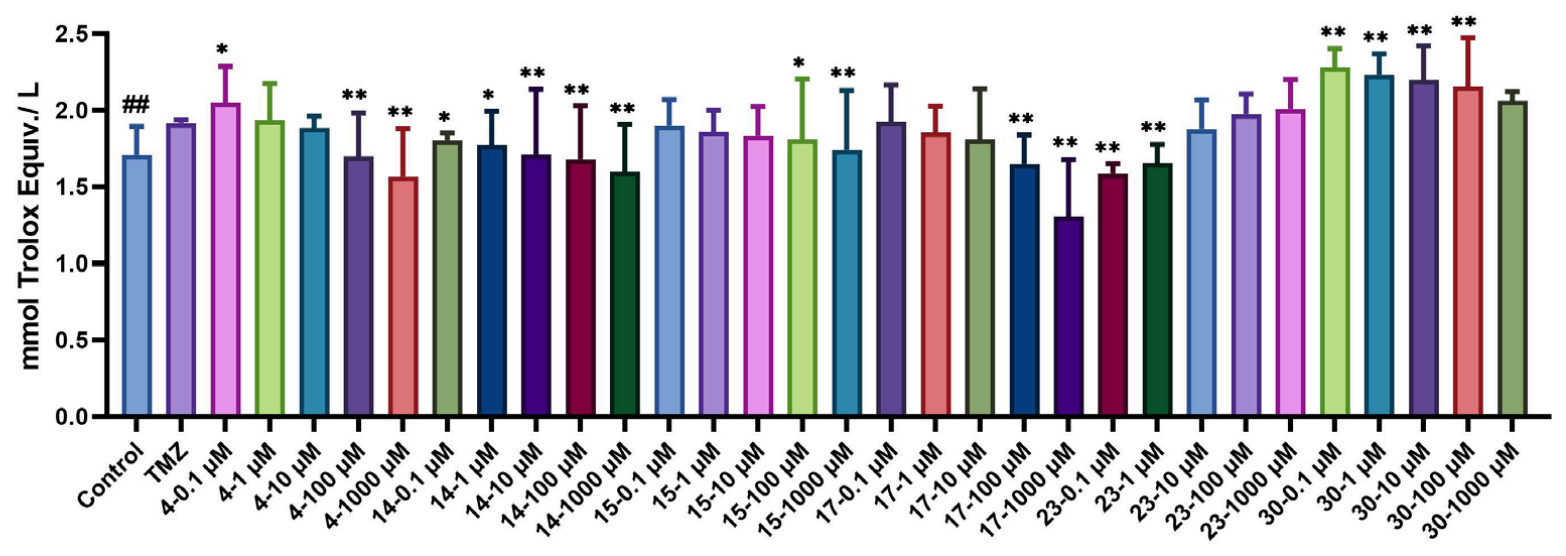
Effects of compounds **4**, **14**, **15**, **17**, **23**, and **30** at various concentrations on TAS levels in U87 cells after 72 h. ## Comparison between TMZ and control groups, *p* < 0.001; * *p* < 0.05, ** *p* < 0.001.

According to the 72-h results, compound **30** was found to significantly increase TAS levels in U87 cells, particularly at concentrations of 0.1, 1, 10, and 100 µM (*p* < 0.001) (Figure 9).

**Figure 10:**
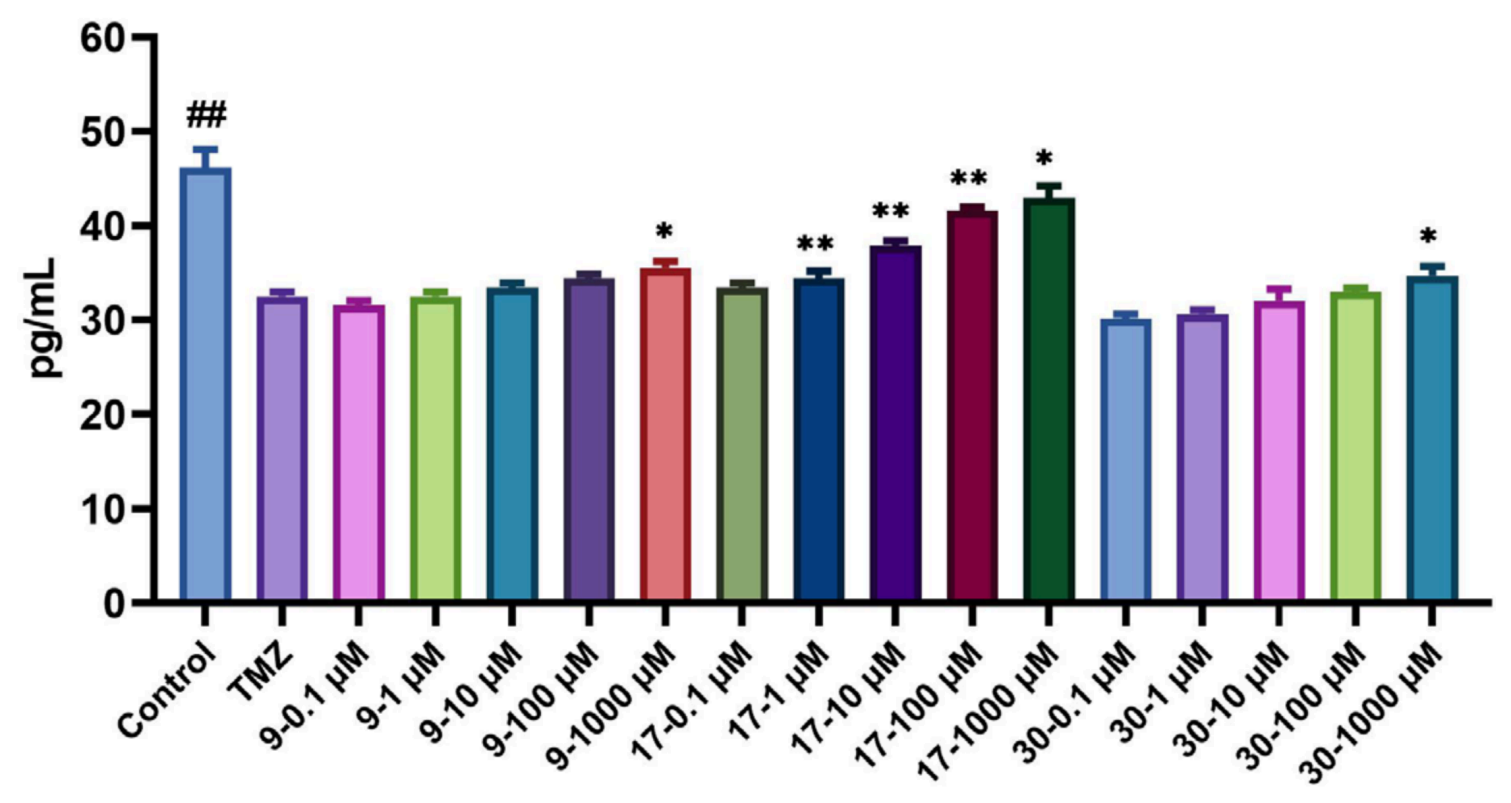
Effects of compounds **9**, **17**, and **30** at various concentrations on IL-1β levels in T98 cells after 72 h. ## Comparison between TMZ and control groups, *p* < 0.001; * *p* < 0.05, ** *p* < 0.001.

Compared with TMZ, compound **30** was found to decrease IL-1β levels in T98 cells, particularly at concentrations of 0.1, 1, and 10 µM. Compound **9** was also determined to reduce IL-1β levels in T98 cells, especially at a concentration of 0.1 µM (Figure 10).

**Figure 11:**
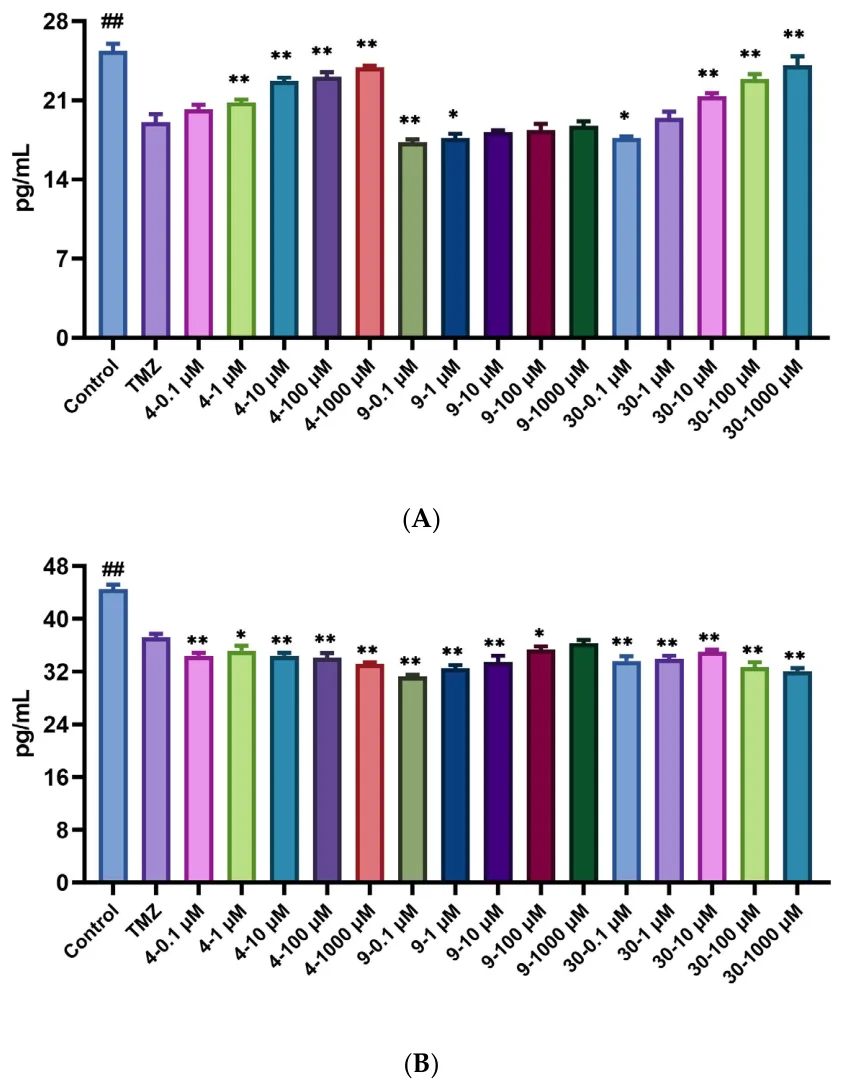
Effects of compounds **4**, **9**, and **30** at various concentrations on IL-1β levels in U87 cells after 24 (**A**) and 48 (**B**) hours. ## Comparison between TMZ and control groups, *p* < 0.001; * *p* < 0.05, ** *p* < 0.001.

According to the 24-h results, compound **9** was found to significantly decrease IL-1β levels in U87 cells, particularly at concentrations of 0.1 and 1 µM (*p* < 0.001 and *p* < 0.05, respectively). Compound **30** was also determined to significantly reduce IL-1β levels in U87 cells, particularly at a concentration of 0.1 µM (*p* < 0.05). According to the 48-h results, compounds **4**, **9**, and **30** significantly decreased IL-1β levels in U87 cells (Figure 11).

**Figure 12:**
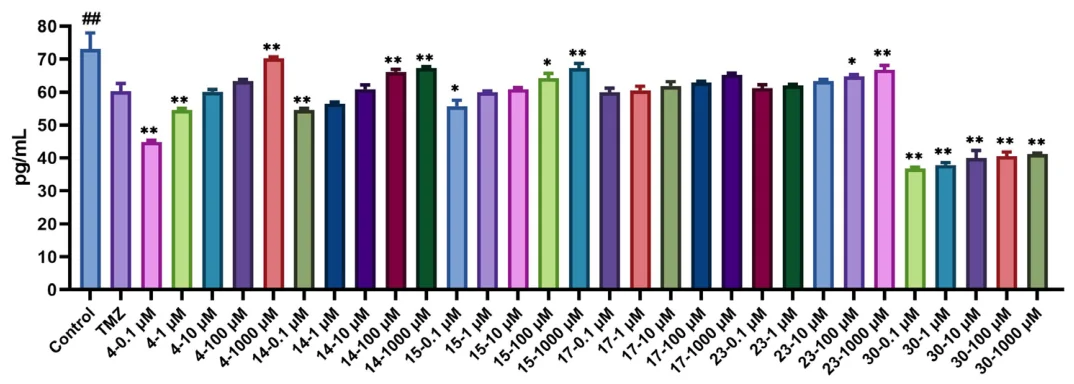
Effects of compounds **4**, **14**, **15**, **17**, **23**, and **30** at various concentrations on IL-1β levels in U87 cells after 72 h. ## Comparison between TMZ and control groups, *p* < 0.001; * *p* < 0.05, ** *p* < 0.001.

According to the 72-h results, compound **4** was found to significantly decrease IL-1β levels in U87 cells, particularly at concentrations of 0.1 and 1 µM (*p* < 0.001). Compound **30** was determined to significantly reduce IL-1β levels in U87 cells at all tested concentrations (*p* < 0.001) (Figure 12).

**Figure 13:**
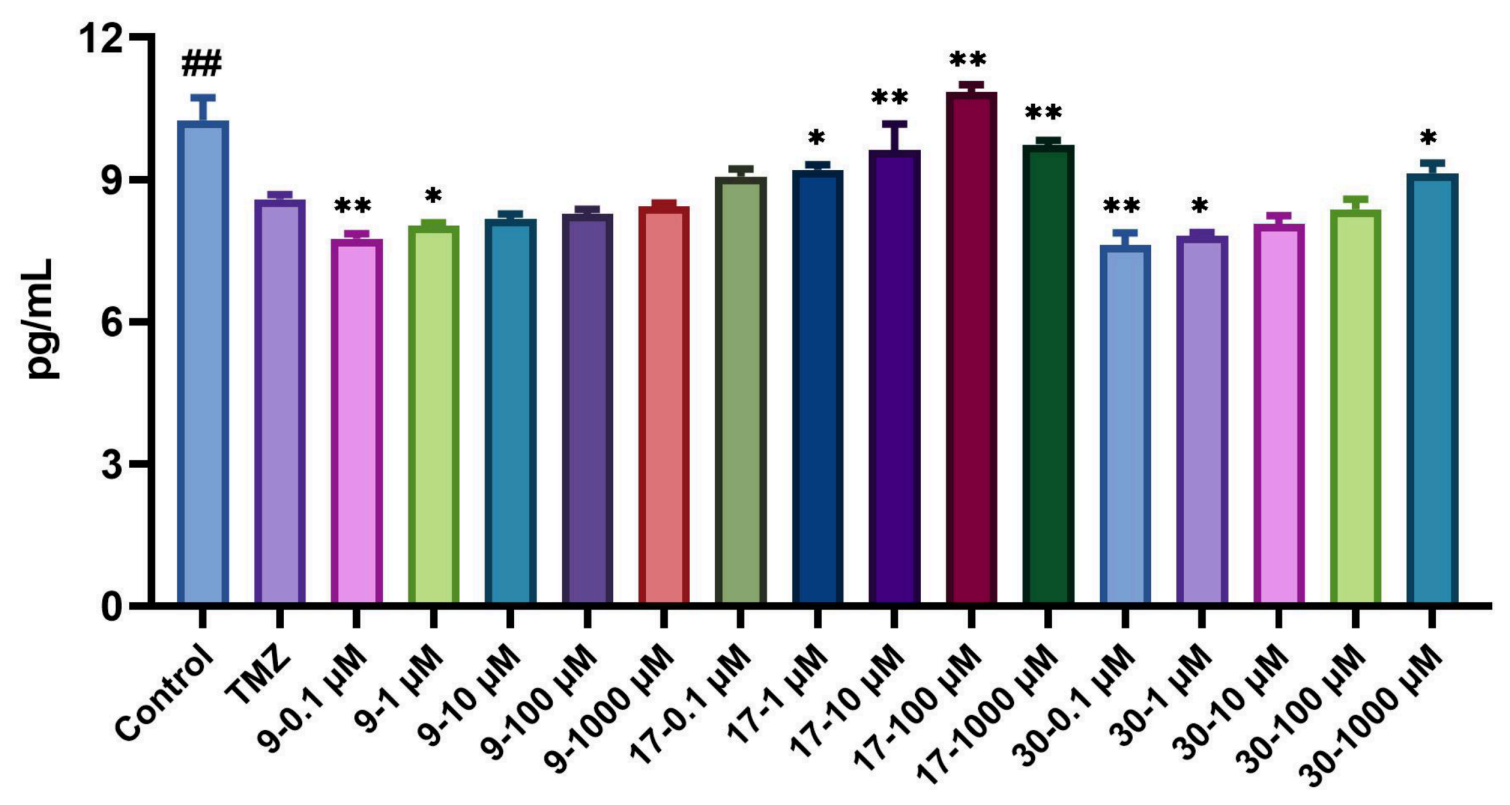
Effects of compounds **9**, **17**, and **30** at various concentrations on IL-10 levels in T98 cells after 72 h. ## Comparison between TMZ and control groups, *p* < 0.001; * *p* < 0.05, ** *p* < 0.001.

Compound **9** was found to significantly decrease IL-10 levels in T98 cells, particularly at concentrations of 0.1 and 1 µM (*p* < 0.001 and *p* < 0.05, respectively). Compound **30** was also determined to reduce IL-10 levels in T98 cells (Figure 13).

**Figure 14:**
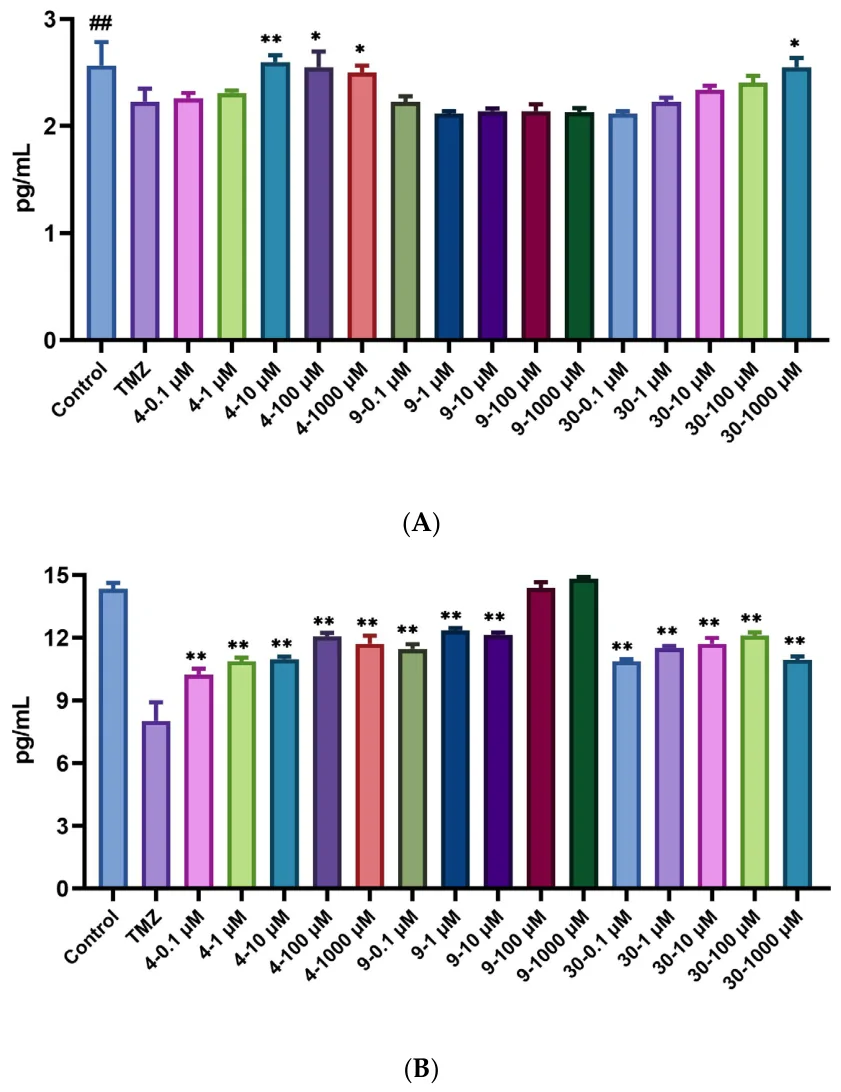
Effects of compounds **4**, **9**, and **30** at various concentrations on IL-10 levels in U87 cells at 24 (**A**) and 48 (**B**) hours. ## Comparison between TMZ and control groups, *p* < 0.001; * *p* < 0.05, ** *p* < 0.001.

According to the 24-h results, compound **9** was found to decrease IL-10 levels in U87 cells. Compared with TMZ, compound **30** was determined to reduce IL-10 levels in U87 cells, particularly at a concentration of 0.1 µM. According to the 48-h results, compounds **4**, **9** (except at concentrations of 100 and 1000 µM), and **30** were found to significantly decrease IL-10 levels in U87 cells compared with the control group (*p* < 0.001) (Figure 14).

**Figure 15:**
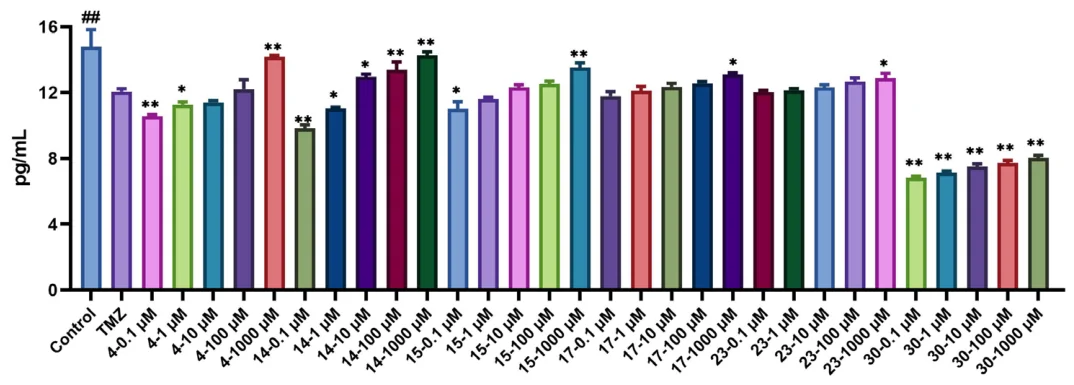
Effects of compounds **4**, **14**, **15**, **17**, **23**, and **30** at various concentrations on IL-10 levels in U87 cells at 72 h. ## Comparison between TMZ and control groups, *p* < 0.001; * *p* < 0.05, ** *p* < 0.001.

According to the 72-h results, compound **4** was found to significantly decrease IL-10 levels in U87 cells, particularly at concentrations of 0.1 and 1 µM (*p* < 0.001 and *p* < 0.05, respectively). Compound **30** was determined to significantly reduce IL-10 levels in U87 cells at all tested concentrations (*p* < 0.001) (Figure 15).

**Figure 16:**
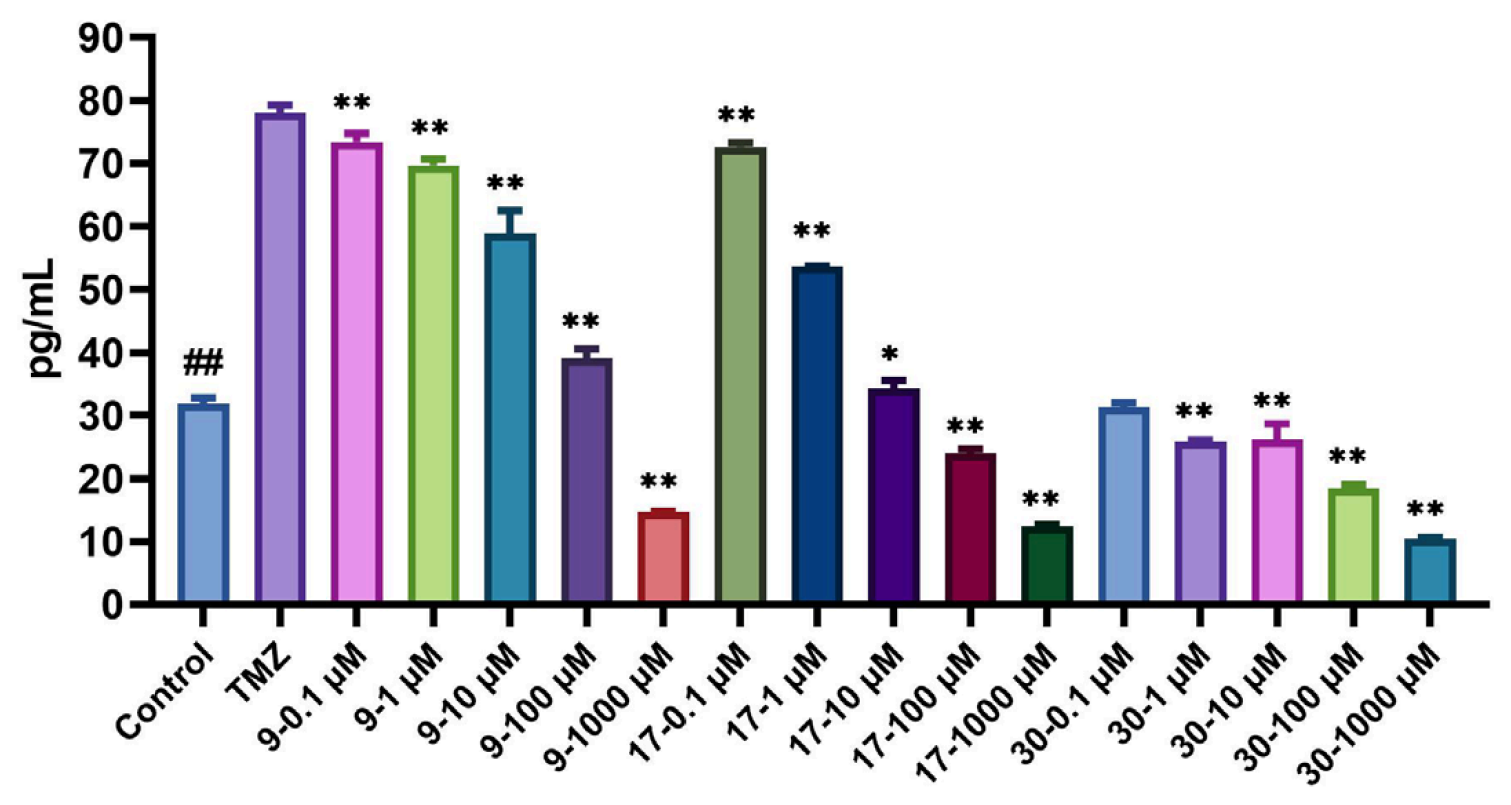
Effects of compounds **9**, **17**, and **30** at various concentrations on TNF-α levels in T98 cells after 72 h. ## Comparison between TMZ and control groups, *p* < 0.001; * *p* < 0.05, ** *p* < 0.001.

Compared with the control group, compound **9** significantly decreased TNF-α levels in T98 cells at a concentration of 1000 µM (*p* < 0.001), whereas it significantly increased TNF-α levels at the other tested concentrations (*p* < 0.001). Compound **17** decreased TNF-α levels in T98 cells at concentrations of 100 and 1000 µM, while it significantly increased TNF-α levels at the other concentrations. Compound **30** significantly decreased TNF-α levels in T98 cells at all tested concentrations (*p* < 0.001) (Figure 16).

**Figure 17:**
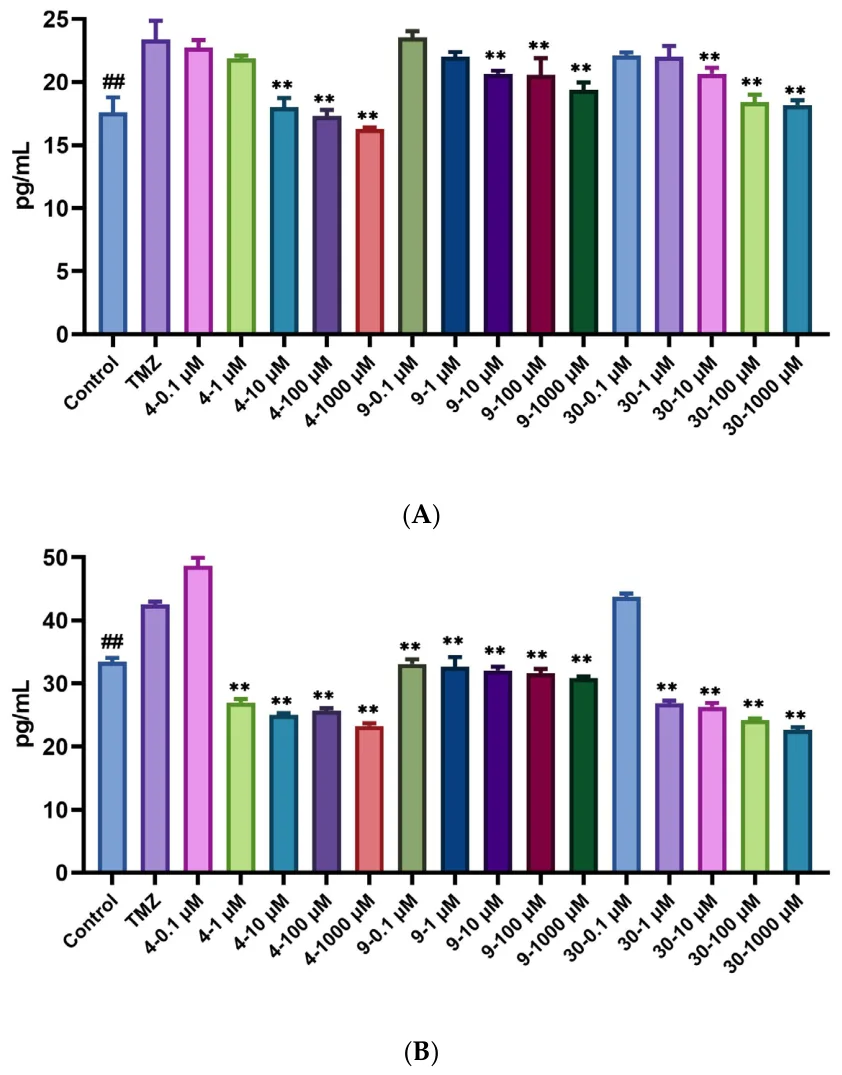
Effects of compounds **4**, **9**, and **30** at various concentrations on TNF-α levels in U87 cells after 24 (**A**) and 48 (**B**) hours. ## Comparison between TMZ and control groups, *p* < 0.001; ** *p* < 0.001.

According to the 24-h results, compound **4** significantly decreased TNF-α levels in U87 cells at concentrations of 100 and 1000 µM (*p* < 0.001), whereas it increased TNF-α levels at the other tested concentrations. Additionally, compounds **9** and **30** were found to increase TNF-α levels at all tested concentrations (Figure 17A).

According to the 48-h results, compounds **4** and **30** increased TNF-α levels at a concentration of 0.1 µM, while these compounds decreased TNF-α levels at the other tested concentrations. Furthermore, compound **9** was determined to decrease TNF-α levels at all tested concentrations (Figure 17B).

**Figure 18:**
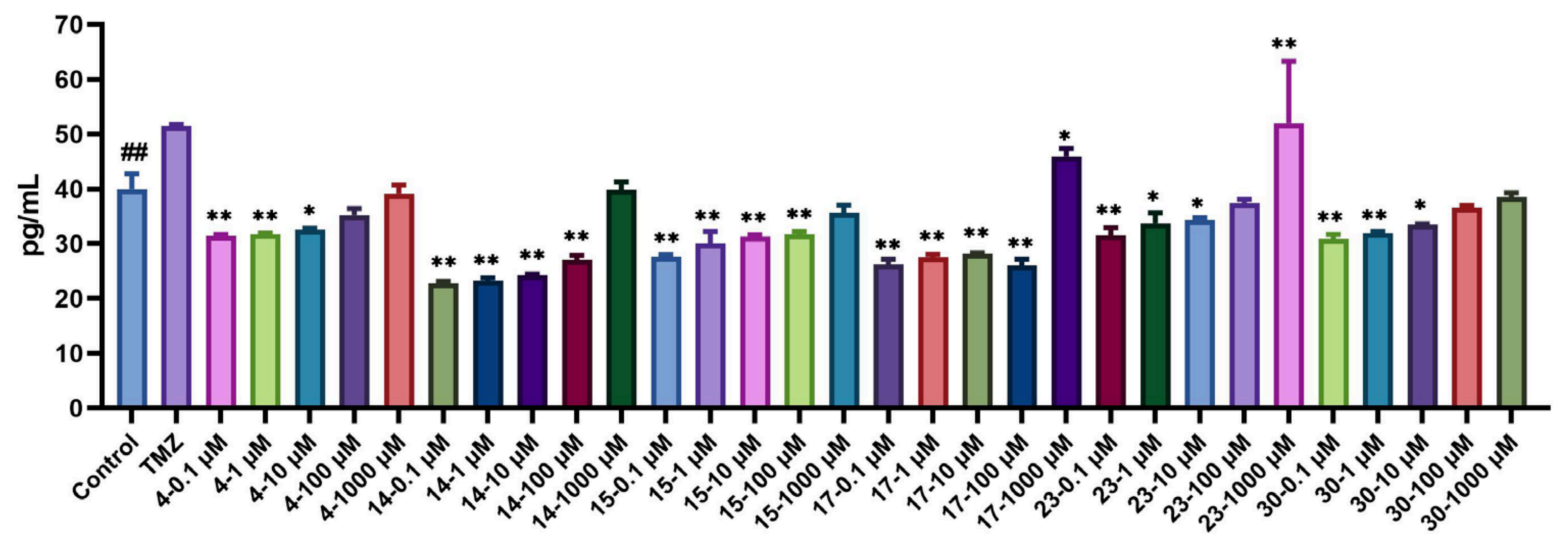
Effects of compounds **4**, **14**, **15**, **17**, **23**, and **30** at various concentrations on TNF-α levels in U87 cells after 72 h. ## Comparison between TMZ and control groups, *p* < 0.001; * *p* < 0.05, ** *p* < 0.001.

Based on the 72-h results, compounds **17** and **23** were found to increase TNF-α levels in U87 cells at a concentration of 1000 µM, while these compounds decreased TNF-α levels at the other tested concentrations. Compounds **4**, **14**, **15**, and **30** were determined to decrease TNF-α levels in U87 cells at all tested concentrations (Figure 18).

**Figure 19:**
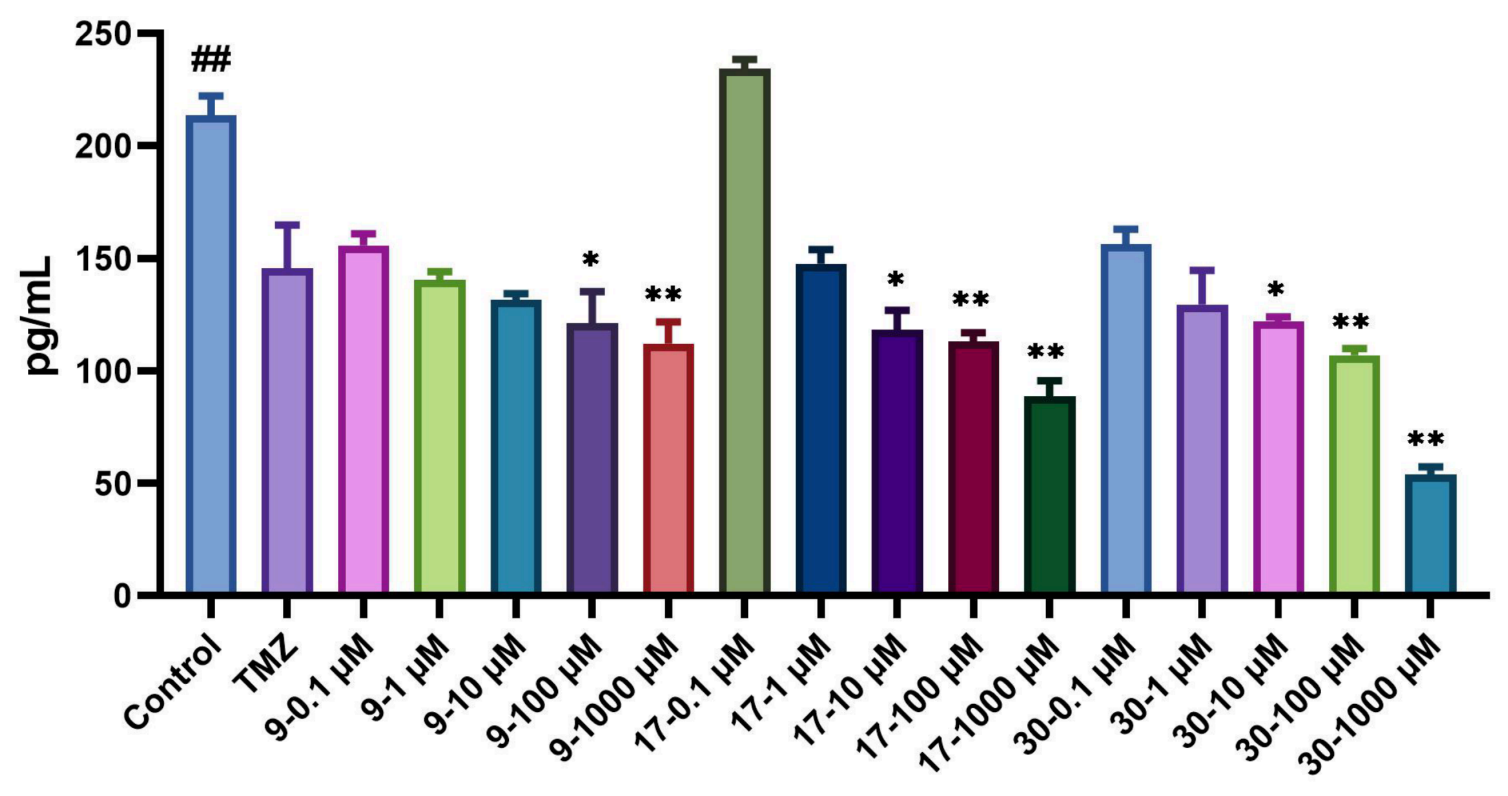
Effects of compounds **9**, **17**, and **30** at various concentrations on TGF-β levels in T98 cells after 72 h. ## Comparison between TMZ and control groups, *p* < 0.001; * *p* < 0.05, ** *p* < 0.001.

Compared with the control group, compounds **9** and **30** were found to decrease TGF-β levels in T98 cells in a dose-dependent manner. Compound **17** increased TGF-β levels in T98 cells at a concentration of 0.1 µM, whereas it decreased TGF-β levels at the other tested concentrations (Figure 19).

**Figure 20:**
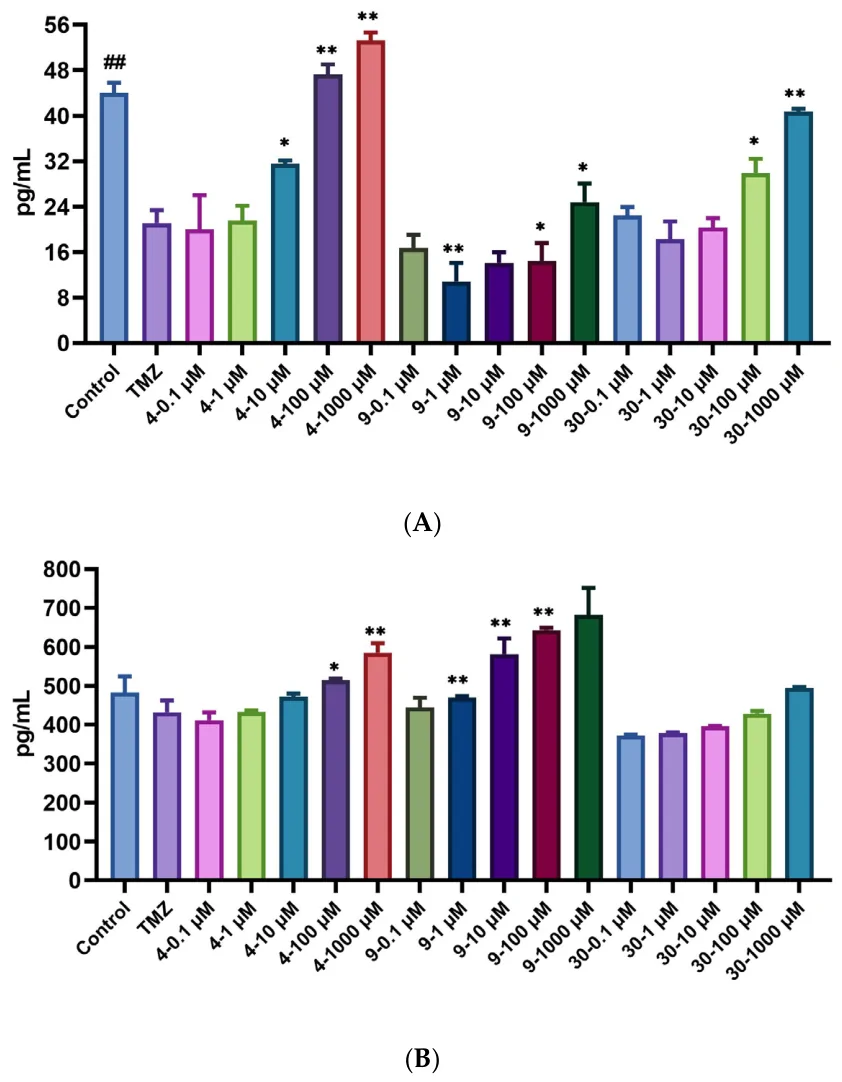
Effects of compounds **4**, **9**, and **30** at various concentrations on TGF-β levels in U87 cells after 24 (**A**) and 48 (**B**) hours. ## Comparison between TMZ and control groups, *p* < 0.001; * *p* < 0.05, ** *p* < 0.001.

According to the 24- and 48-h results, compound **4** increased TGF-β levels in U87 cells at concentrations of 100 and 1000 µM, whereas it decreased TGF-β levels at the other tested concentrations. Based on the 24-h results, compounds **9** and **30** were found to decrease TGF-β levels in U87 cells compared with the control group. According to the 48-h results, compound **9** increased TGF-β levels in U87 cells at concentrations of 10, 100, and 1000 µM, while it decreased TGF-β levels at the other tested concentrations. Compound **30** increased TGF-β levels in U87 cells at a concentration of 1000 µM, whereas it decreased TGF-β levels at the other tested concentrations (Figure 20).

**Figure 21:**
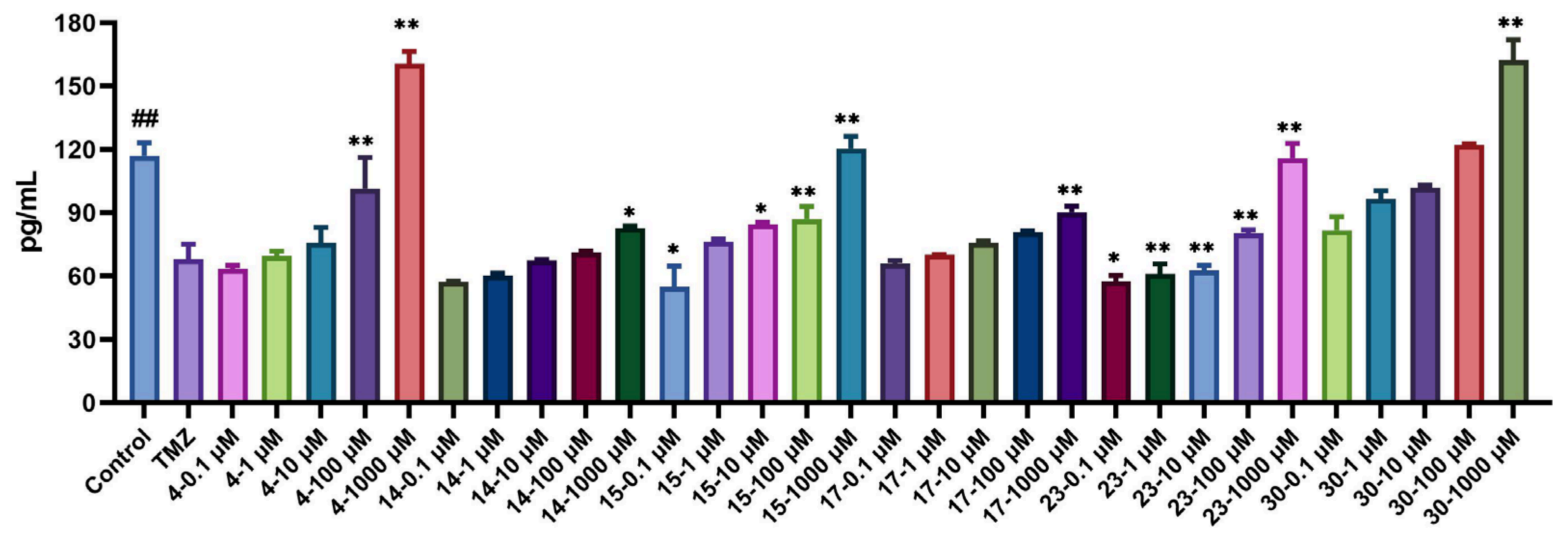
Effects of compounds **4**, **14**, **15**, **17**, **23**, and **30** at various concentrations on TGF-β levels in U87 cells after 72 h. ## Comparison between TMZ and control groups, *p* < 0.001; * *p* < 0.05, ** *p* < 0.001.

Based on the 72-h results, the tested compounds were found to decrease TGF-β levels in U87 cells. However, compounds **4** and **15** were determined to increase TGF-β levels in U87 cells at a concentration of 1000 µM. Compound **30** increased TGF-β levels at concentrations of 100 and 1000 µM (Figure 21).

**Figure 22:**
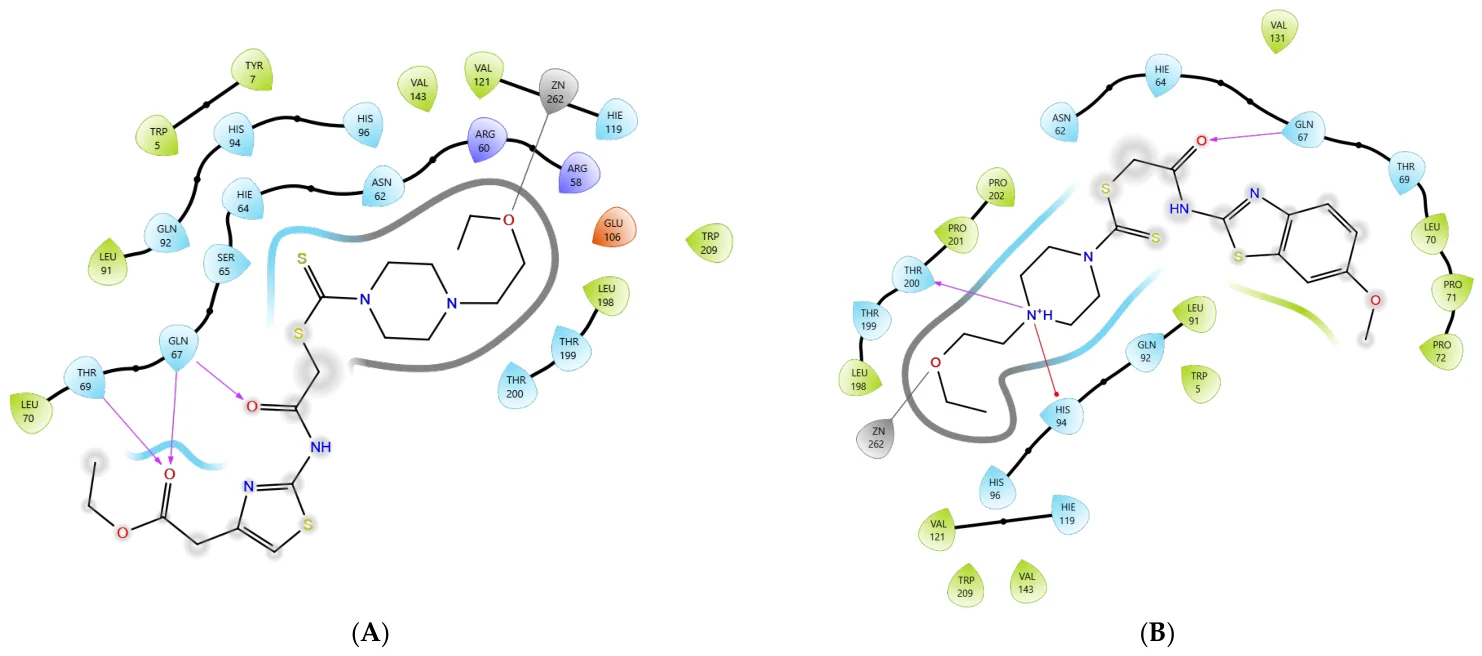

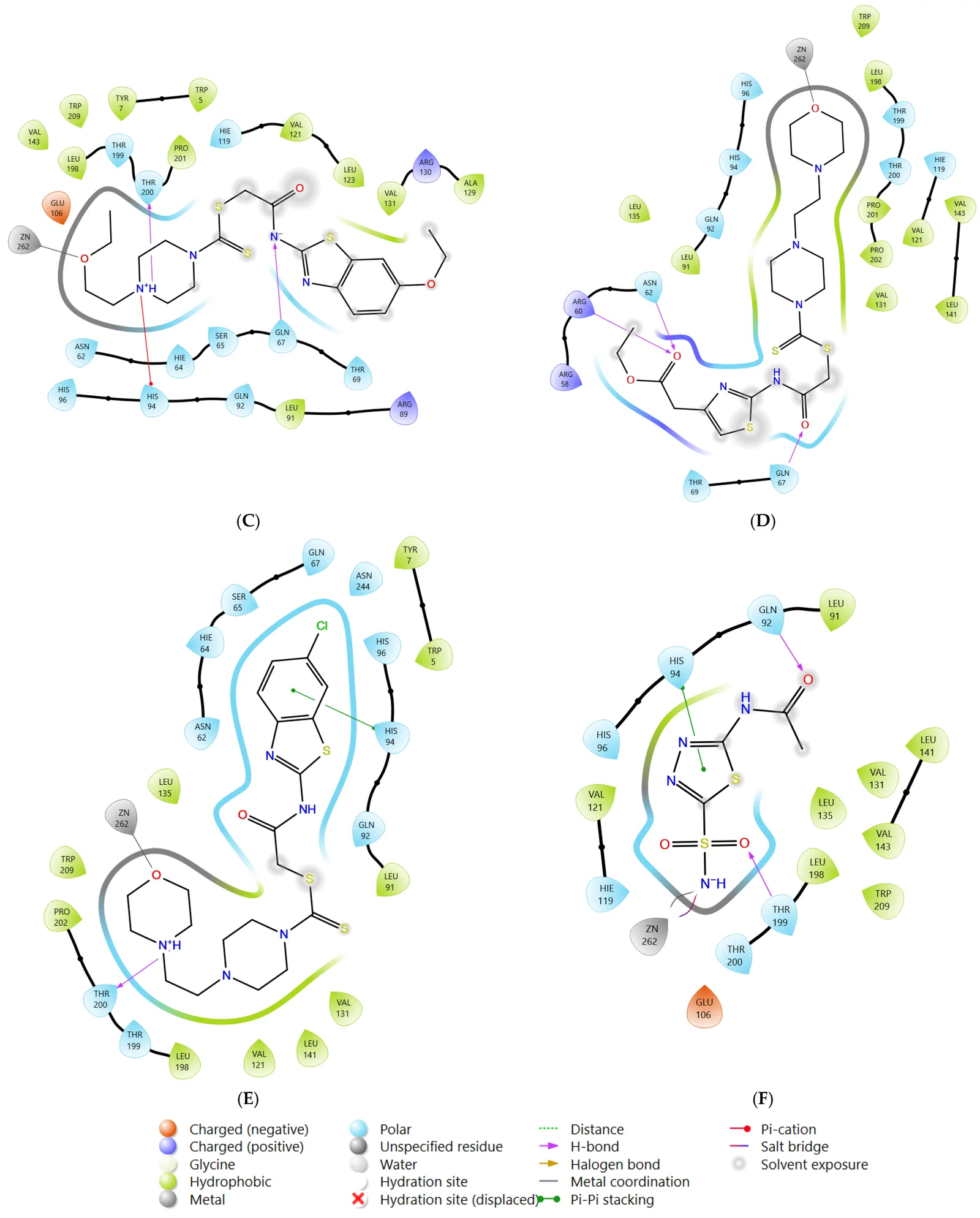
Docking interactions of compound **14** (**A**), compound **20** (**B**), compound **21** (**C**), compound **36** (**D**), compound **39** (**E**), and AAZ (**F**) within the catalytic domain of hCA IX (PDB ID: 3IAI).

According to in vitro studies, compounds **14**, **20**, **21**, **36**, and **39** were found to exhibit stronger hCA IX inhibitory activity than AAZ. Therefore, molecular docking studies were performed using the crystal structure of the catalytic domain of tumor-associated hCA IX (PDB ID: 3IAI). All compounds form coordination bonds with Zn262 (Figure 22).

Compounds **14**, **20**, **21**, and **36** interact with Gln67 through hydrogen bonding, whereas AAZ forms a hydrogen bond with Gln92 (Figure S133). Compound **14** interacts with Thr69 through hydrogen bonding, while compounds **20**, **21**, and **39** form hydrogen bonds with Thr200. AAZ forms a hydrogen bond with the Thr199 amino acid residue. Compounds **20** and **21** exhibit a π–cation interaction between the nitrogen atom of the piperazine ring and the His94 residue, as well as a hydrogen bond interaction with the Thr200 residue. The thiadiazole ring of AAZ exhibits a π–π interaction with the His94 residue. Compound **39** exhibits a π–π interaction between its benzothiazole ring and the His94 amino acid residue. Compound **36** interacts with both Asn62 and Arg60 amino acid residues via hydrogen bonding through the carbonyl group of the acetate moiety attached at the 4th position of the thiazole ring. The docking scores of compounds **14**, **20**, **21**, **36**, and **39** were found to be −3.499, −3.812, −3.976, −3.513, and −3.575 kcal/mol, respectively. The docking scores were also in agreement with the in vitro enzyme assay data, supporting the observed hCA IX inhibitory effects of these compounds.

**Figure 23:**
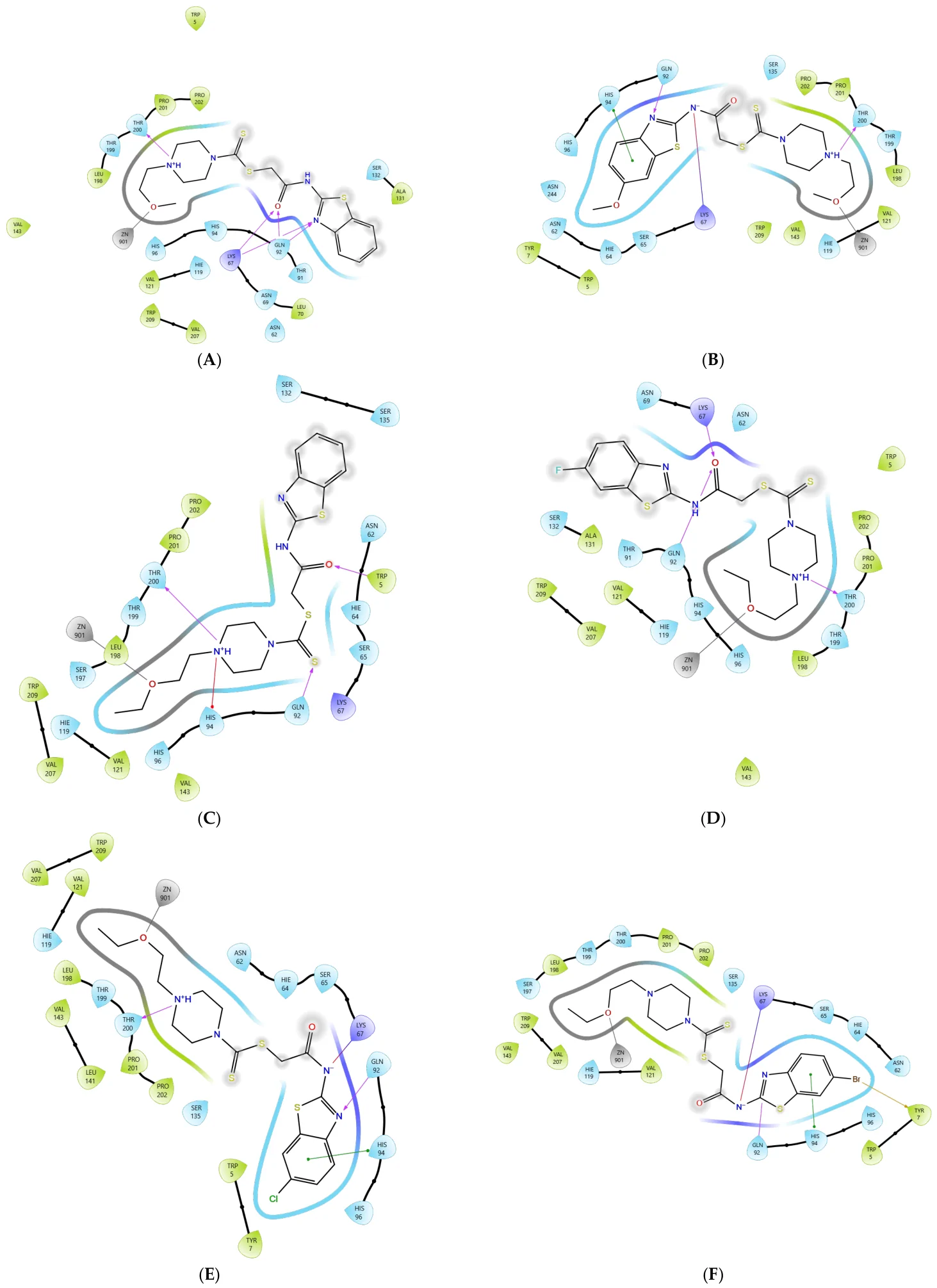

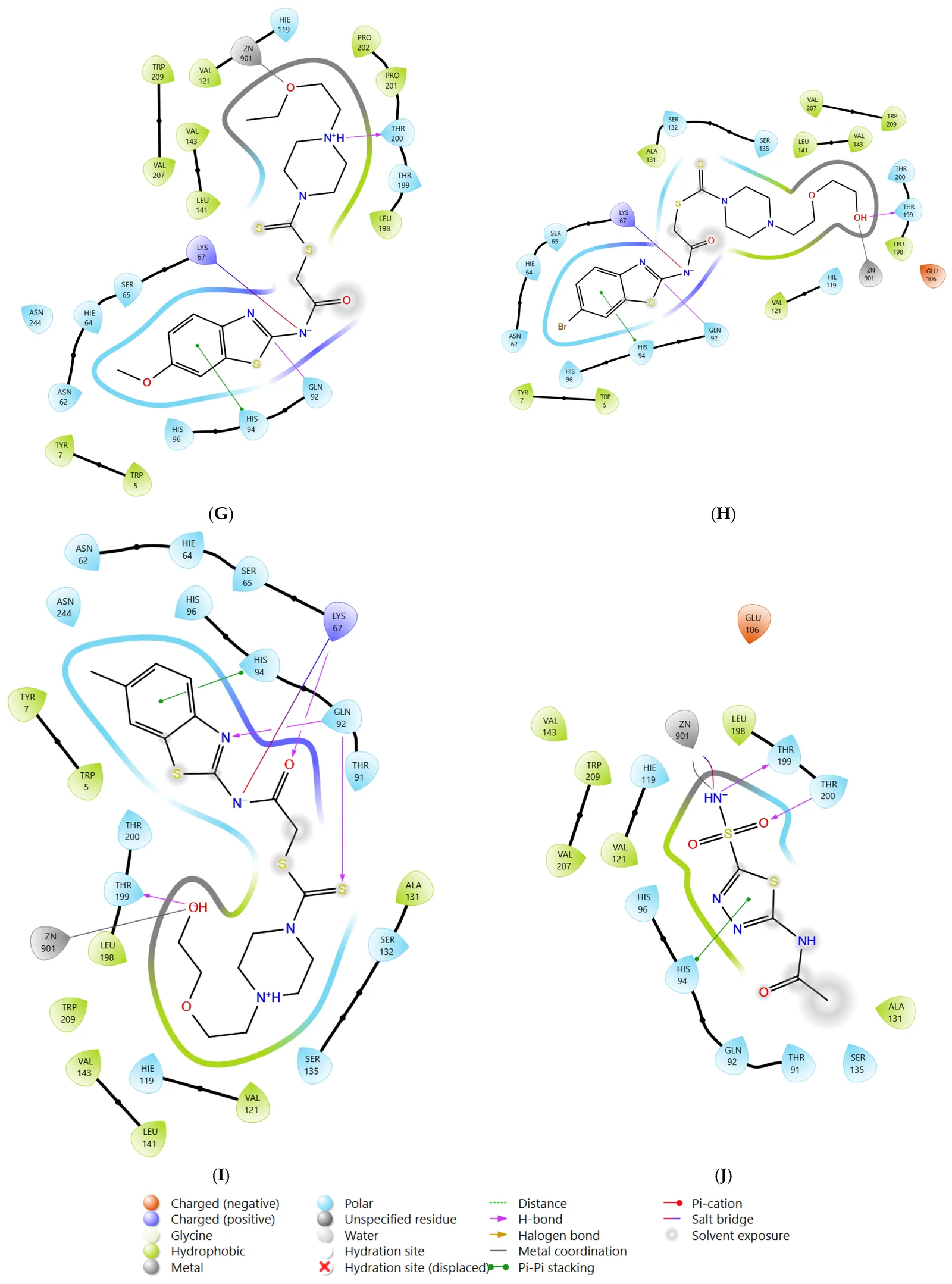
Docking interactions of compound **4** (**A**), compound **9** (**B**), compound **15** (**C**), compound **16** (**D**), compound **17** (**E**), compound **18** (**F**), compound **20** (**G**), compound **29** (**H**), compound **30** (**I**), and AAZ (**J**) in the catalytic domain of hCA XII (PDB ID: 1JD0).

Based on the results of the in vitro studies, compounds **4**, **9**, **15**, **16**, **17**, **18**, **20**, **29**, and **30** were identified as more potent hCA XII inhibitors than AAZ. Consequently, molecular docking studies were carried out using the crystal structure of the catalytic domain of hCA XII (PDB ID: 1JD0). All compounds interact with Zn901 through coordination bonds (Figure 23). AAZ and compounds **4**, **9**, **15**, **16**, **17**, and **20** interact with the Thr200 residue through hydrogen bonding. AAZ and compounds **29** and **30** interact with the Thr199 amino acid residue through hydrogen bonding. In contrast, no hydrogen bond interactions were observed between compound **18** and either Thr199 or Thr200 (Figure S134). Compounds **4**, **9**, **16**, **17**, **18**, **20**, **29**, and **30** exhibit hydrogen bond and/or salt bridge interactions with the Lys67 amino acid residue. All compounds interact with the Gln92 amino acid residue through hydrogen bonding. AAZ and compounds **9**, **17**, **18**, **20**, **29**, and **30** exhibit π–π interactions with His94, whereas compound **15** interacts with the same amino acid residue through a π–cation interaction. Compound **15** also forms a hydrogen bond with Trp5. Compound **18** interacts with the Tyr7 residue via a halogen bond mediated by its Br atom. The docking scores of compounds **4**, **9**, **15**, **16**, **17**, **18**, **20**, **29**, and **30** were found as −3.198, −3.294, −4.187, −2.916, −5.632, −3.481, −3.525, −2.656, and −2.935 kcal/mol, respectively. The docking scores were also in agreement with the in vitro enzyme assay results, supporting the observed hCA XII inhibitory effects of these compounds.

Compounds **4**, **9** and **30**, which exhibited marked cytotoxic activity against GBM cells by modulating oxidative stress and inflammatory mediators and inhibiting tumor-associated hCA isoforms, were further subjected to in silico pharmacokinetic evaluation by running QikProp, a predictive Absorption, Distribution, Metabolism, and Excretion (ADME) module within the Maestro suite produced by Schrödinger (Table 5). For the three compounds, the total solvent accessible surface area (SASA), predicted octanol/water partition coefficient (QPlogPo/w), predicted aqueous solubility (QPlogS), and predicted human serum albumin binding (QPlogKhsa), and predicted brain/blood partition coefficient (QPlogBB) values were all found to be within the recommended ranges. Furthermore, all three compounds were found to comply with Lipinski’s Rule of Five (Ro5) and Jorgensen’s Rule of Three (Ro3).

## 3. Discussion

In recent years, the DTC pharmacophore has emerged as an important scaffold in the design and development of hCA inhibitors [22,23,24,25,26,27,28]. Although numerous DTC derivatives with potent hCA inhibitory activity have been reported, no DTC-based hCA inhibitor has yet been approved as a therapeutic agent. Moreover, previous studies on DTC-based hCA inhibitors have primarily focused on their potential application in the treatment of glaucoma [26,27].

**Figure 24:**
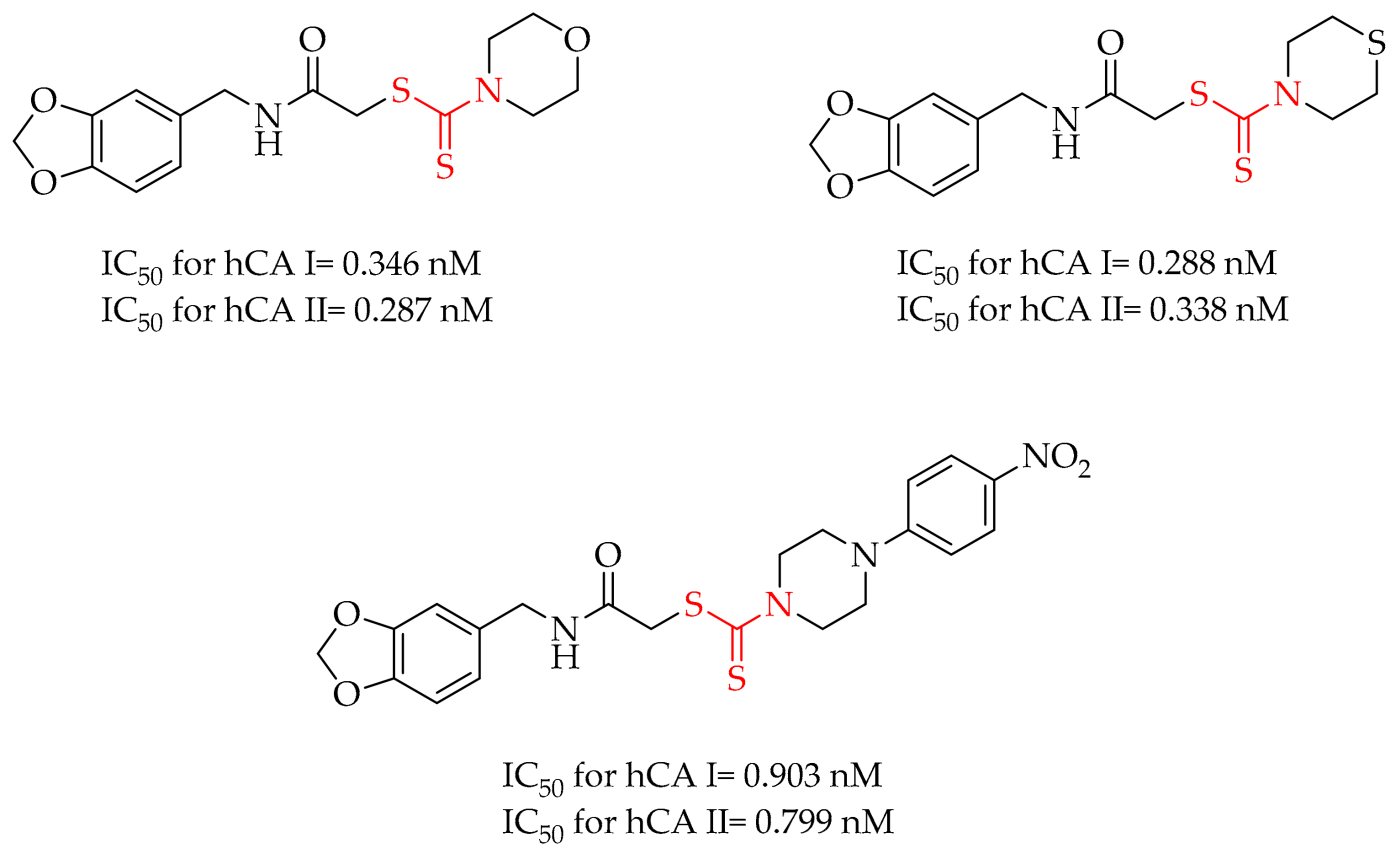
IC_50_ values of DTCs with significant inhibitory activity against hCA I and II.

In our study published in 2017 [28], ten DTC derivatives were synthesized and evaluated for their cytotoxic activity against both A549 human lung adenocarcinoma and C6 rat glioma cell lines, as well as their inhibitory effects on hCA I and II. All compounds exhibited potent hCA inhibitory activity, with IC_50_ values in the low nanomolar range. The IC_50_ values of the most active hCA I and II inhibitors identified in this study are presented in Figure 24. Among these hCA inhibitors, the compound bearing a 4-(4-nitrophenyl)piperazin-1-yl moiety exhibited cytotoxic activity against the C6 cell line (IC_50_ = 23.33 µg/mL) comparable to that of cisplatin (IC_50_ = 19.00 µg/mL). However, none of the synthesized compounds showed significant cytotoxic activity against the A549 cell line.

Building upon the findings of our previous study, which demonstrated the hCA inhibitory effects and antiglioma activity of a piperazine-containing dithiocarbamate derivative (Figure 24) [28], forty-four novel DTC derivatives (Table 6) were designed and synthesized. Their chemical structures were elucidated using spectroscopic techniques. The in vitro inhibitory activities of these compounds against hCA I, II, IX, and XII were evaluated.

**Figure 25:**
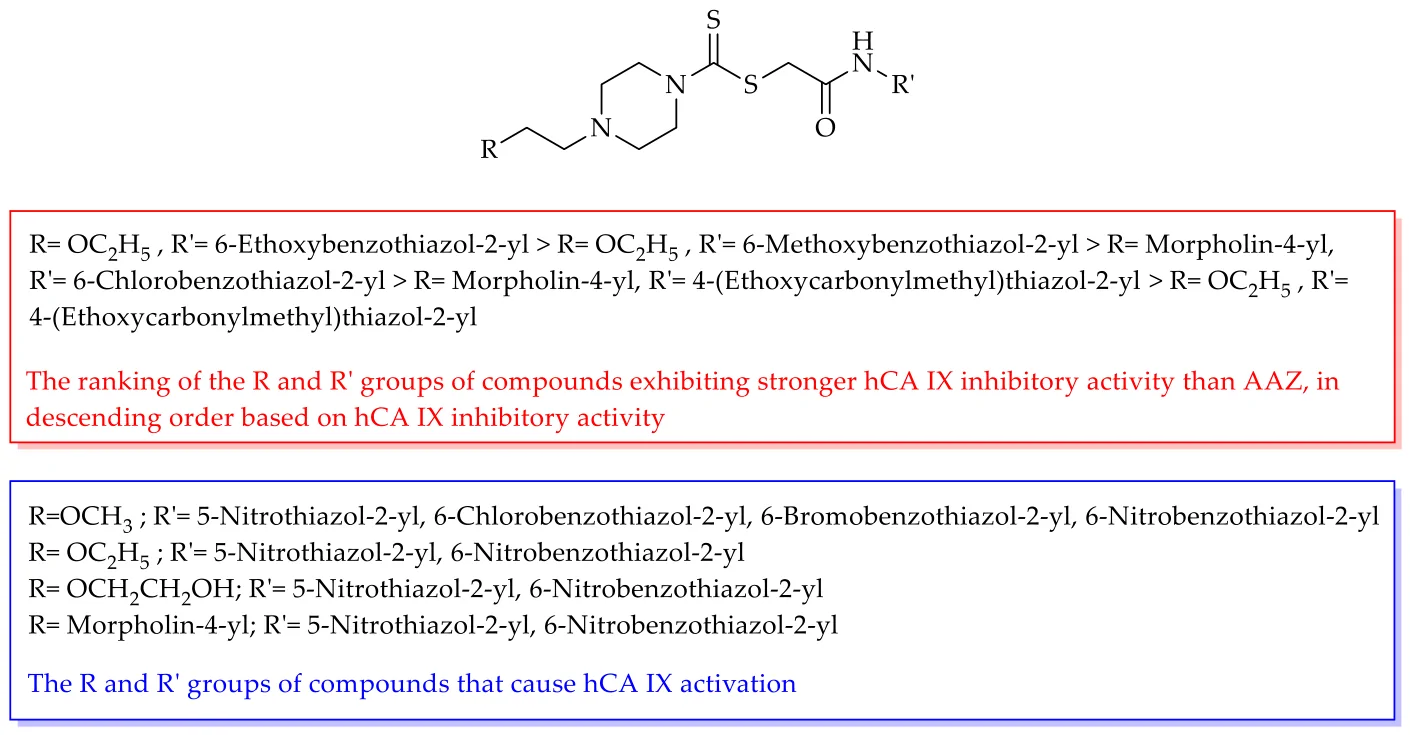
Summary of the structure–activity relationships for hCA IX IC_50_ values.

Most of the synthesized compounds exhibited inhibitory activity against the tumor-associated hCA isoforms hCA IX and/or hCA XII. Among them, compounds **14**, **20**, **21**, **36**, and **39** displayed more potent hCA IX inhibitory activity than AAZ (Figure 25), while compound **41** exhibited inhibitory potency comparable to that of AAZ. Enzyme kinetic studies revealed that these compounds inhibited hCA IX in a competitive manner.

**Figure 26:**
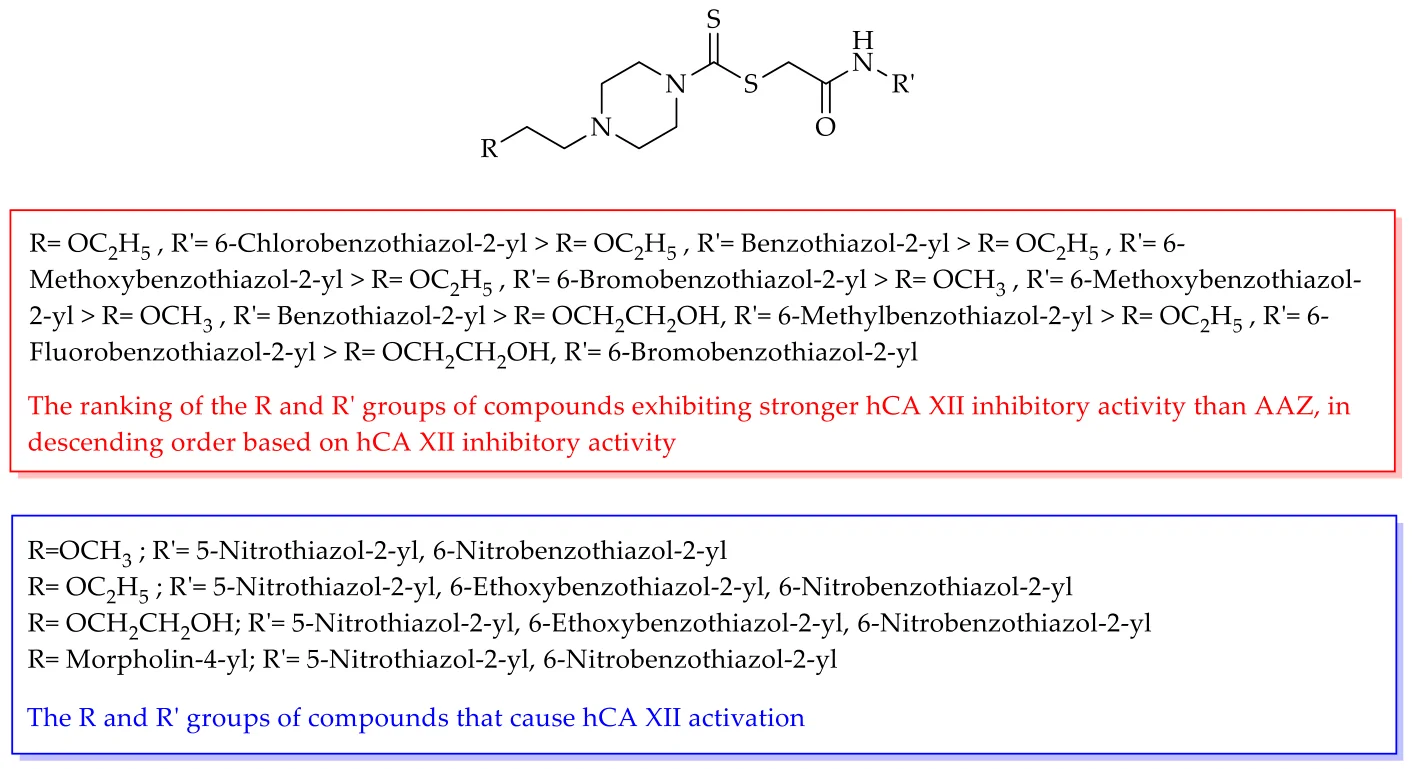
Summary of the structure–activity relationships for hCA XII IC_50_ values.

Compounds **4**, **9**, **15**, **16**, **17**, **18**, **20**, **29**, and **30** exhibited stronger hCA XII inhibitory activity than AAZ (Figure 26) and were identified as competitive inhibitors of hCA XII. Notably, compound **20** was identified as a potent competitive inhibitor of both hCA IX (IC_50_ = 14.72 nM, *K*_i_ = 13.73 nM) and XII (IC_50_ = 17.61 nM, *K*_i_ = 11.99 nM) isoforms.

DTC-based compounds bearing 5-nitrothiazole (compounds **2**, **13**, **24** and **35**) and 6-nitrobenzothiazole (**11**, **22**, **33** and **44**) moieties acted as activators of hCA IX and XII (Figure 25 and Figure 26). The unexpected activation of hCA IX and XII by the 5-nitrothiazole- and 6-nitrobenzothiazole-containing DTC derivatives may be attributed to the strong electron-withdrawing nature of the nitro substituent. The nitro substituent is likely to alter the electronic distribution of the heterocyclic scaffolds (thiazole and benzothiazole), thereby modifying the binding orientation of the DTC moiety within the enzyme active sites of hCA IX and XII, disfavoring the classical Zn^2+^-coordination mode responsible for hCA IX and XII inhibition [27], and consequently promoting enzyme activation.

In addition, molecular docking studies were performed for compounds exhibiting inhibitory activity against hCA IX and/or XII to investigate their potential binding modes and molecular interactions with the target enzymes. Consistent with the experimental findings, in silico molecular docking studies demonstrated that these compounds established the expected interactions with key amino acid residues within the active sites of the tumor-associated hCA IX and/or XII isoforms, thereby providing a rationale for their inhibitory activities.

The cytotoxic effects of compounds showing potent hCA IX and/or hCA XII inhibitory activity were evaluated against U87 and T98 human glioblastoma cell lines, as well as healthy neuronal cells. Among the tested compounds, compound **30** exhibited the highest cytotoxic activity against T98 cells (IC_50_ = 57.71 µM), followed by compounds **9** (IC_50_ = 69.69 µM) and **17** (IC_50_ = 96.62 µM). These compounds exhibited notable cytotoxic activity, with reference to the previously reported IC_50_ value of TMZ (438.30 µM) [31].

The tested compounds were found to show more potent cytotoxic effects against the U87 cell line. Based on the 24 h cytotoxicity results, compounds **4**, **30**, and **9** showed the highest cytotoxic activity against the U87 cell line among the tested compounds, with IC_50_ values of 33.85, 61.28, and 74.26 µM, respectively. The 48 h cytotoxicity results revealed that compounds **30**, **4**, **9**, and **15** exhibited the most potent cytotoxic activity against the U87 cell line, with the IC_50_ values of 17.76, 35.56, 35.64, and 73.19 µM, respectively. Based on the 72 h cytotoxicity results, compounds **4**, **15**, **30**, **23**, **14**, and **17** displayed the highest cytotoxic activity against the U87 cell line, with the IC_50_ values of 3.86, 4.79, 19.51, 20.33, 41.73, and 87.39 µM, respectively. Moreover, compounds **4**, **9**, **14**, **15**, **17**, **23**, and **30** exhibited no significant toxicity toward cerebellar and cortical cells derived from primary cultures of neonatal rat brain.

The effects of the most active anti-GBM agents on oxidative stress, inflammation, and cell death-related pathways in glioblastoma cells were also investigated to provide further insight into their underlying mechanisms of action.

Several studies have reported that oxidative stress is increased in patients with glioma, as evidenced by elevated TOS levels and reduced TAS levels [32,33]. Notably, compound **30** significantly reduced TOS levels in T98 cells at all tested concentrations, whereas compound **17** exerted an attenuating effect particularly at a concentration of 0.1 µM. Furthermore, both compounds **17** and **30** increased TAS levels in T98 cells at 0.1 µM concentration. Compound **9** also significantly enhanced TAS levels in T98 cells, especially at the 0.1 µM concentration.

Cytokines play a crucial role in regulating immune responses within the glioma tumor microenvironment (TME). IL-1β promotes inflammation and tumor progression by enhancing angiogenesis and recruiting immunosuppressive cells. Additionally, it creates a pro-inflammatory environment that supports tumor survival. IL-10 is a key immunosuppressive cytokine in the glioma TME, promoting tumor growth and immune evasion by suppressing T cell activity and inducing M2-like macrophage polarization [34].

TNF-α is a pleiotropic pro-inflammatory cytokine that regulates diverse cellular processes, including proliferation, survival, differentiation, and cell death [35,36]. Inhibition of TNF-α signaling has been reported to suppress glioma-associated macrophage-induced endothelial cell activation and improve survival in mouse glioma models [37].

TGF-β is a multifunctional cytokine that plays a pivotal role in regulating a wide range of cellular processes, including cell proliferation, differentiation, and tissue homeostasis. Dysregulation of TGF-β signaling is commonly observed in cancer, and aberrant activation of this pathway contributes, at least in part, to tumor initiation and progression across multiple malignancies, including GBM [38]. TGF-β signaling has also been reported to contribute to resistance against chemotherapy, targeted therapy, and immunotherapy, thereby leading to treatment failure. In GBM, TGF-β contributes to TMZ resistance by promoting O^6^-methylguanine-DNA methyltransferase (MGMT) accumulation and downregulating miR-198 expression, highlighting the potential of combining TGF-β inhibitors with therapeutic approaches such as TMZ or immunotherapy to enhance treatment efficacy [39].

Compared with TMZ, compound **30** reduced IL-1β levels in T98 cells at concentrations of 0.1, 1, and 10 µM. In addition, compound **30** was found to decrease IL-10 levels in T98 cells. Compound **9** also reduced IL-1β levels in T98 cells, particularly at the 0.1 µM concentration. Furthermore, compound **9** significantly decreased IL-10 levels in T98 cells, especially at 0.1 and 1 µM concentrations.

Compound **30** significantly reduced TNF-α levels in T98 cells at all tested concentrations. On the other hand, compounds **9** and **30** were found to decrease TGF-β levels in T98 cells in a dose-dependent manner.

In terms of their effects on the parameters associated with oxidative stress, inflammation, and cell death mechanisms in T98 glioblastoma cells, compounds **9** and **30** were found to be the most prominent compounds in T98 cells.

According to the 24-h results, compound **4** was found to decrease TOS levels in U87 cells at a concentration of 0.1 µM. Based on the 72-h results, compound **15** reduced TOS levels in U87 cells at a concentration of 0.1 µM, similarly to TMZ. According to the 24-h results, compound **9** significantly increased TAS levels in U87 cells, particularly at a concentration of 0.1 µM. The 48-h results showed that compounds **4**, **9**, and **30** increased TAS levels in U87 cells at a concentration of 0.1 µM. According to the 72-h results, compound **30** was found to significantly increase TAS levels in U87 cells, particularly at concentrations of 0.1, 1, 10, and 100 µM.

According to the 24-h results, compound **9** was found to significantly decrease IL-1β levels in U87 cells, particularly at concentrations of 0.1 and 1 µM. Compound **30** was determined to significantly reduce IL-1β levels in U87 cells, especially at a concentration of 0.1 µM. The 48-h results showed that compounds **4**, **9**, and **30** significantly decreased IL-1β levels in U87 cells. According to the 72-h results, compound **4** significantly reduced IL-1β levels in U87 cells, particularly at concentrations of 0.1 and 1 µM. Compound **30** was found to significantly decrease IL-1β levels in U87 cells at all tested concentrations.

According to the 24-h results, compound **9** was found to decrease IL-10 levels in U87 cells. Compared with TMZ, compound **30** was determined to reduce IL-10 levels in U87 cells, particularly at a concentration of 0.1 µM. The 48-h results revealed that, compared with the control group, compounds **4**, **9** (except at 100 and 1000 µM concentrations), and **30** significantly decreased IL-10 levels in U87 cells. According to the 72-h results, compound **4** significantly reduced IL-10 levels in U87 cells, particularly at concentrations of 0.1 and 1 µM. Compound **30** was found to significantly decrease IL-10 levels in U87 cells at all tested concentrations.

Compound **4** exhibited a time- and concentration-dependent effect on TNF-α production in U87 cells, showing significant inhibition at high concentrations (100 and 1000 µM) after 24 h, suppression at all concentrations except 0.1 µM after 48 h, and consistent reduction of TNF-α levels across all tested concentrations after 72 h, suggesting a sustained anti-inflammatory effect with prolonged exposure. Compound **9** exhibited a clear time-dependent effect on TNF-α production in U87 cells, increasing TNF-α levels at all tested concentrations after 24 h but consistently reducing TNF-α levels across all tested concentrations after 48 h, suggesting a time-dependent modulation of the inflammatory response in U87 cells. Compound **30** exhibited a time-dependent effect on TNF-α production in U87 cells, initially increasing TNF-α levels at all tested concentrations after 24 h, reducing TNF-α levels at all concentrations except 0.1 µM after 48 h, and consistently suppressing TNF-α production across all tested concentrations after 72 h, suggesting that its anti-inflammatory activity became more pronounced with prolonged exposure.

Compound **4** exhibited a concentration-dependent effect on TGF-β production in U87 glioblastoma cells, increasing TGF-β levels at higher concentrations (100 and 1000 µM) after 24 and 48 h, whereas lower concentrations reduced TGF-β production. However, after 72 h, TGF-β levels were decreased at all tested concentrations except 1000 µM, suggesting enhanced suppression of TGF-β production following prolonged exposure. Compound **9** demonstrated a time- and concentration-dependent modulation of TGF-β production, reducing TGF-β levels at all tested concentrations after 24 h while increasing TGF-β levels at 10–1000 µM after 48 h, indicating a transient alteration in TGF-β regulation during intermediate exposure. Compound **30** predominantly reduced TGF-β production in U87 glioblastoma cells, although increased TGF-β levels were observed at the highest concentration (1000 µM) after 48 h and at higher concentrations (100 and 1000 µM) after 72 h, suggesting that its suppressive effect on TGF-β production is reduced at elevated concentrations.

When the tested compounds were evaluated regarding their effects on the parameters related to oxidative stress, inflammation, and cell death mechanisms in U87 glioblastoma cells, compounds **4**, **9**, and **30** emerged as the most promising compounds in U87 cells.

In silico ADME screening accelerates lead identification and optimization while reducing the cost, time, and ethical burden associated with extensive in vivo studies [40,41,42]. The in silico evaluation of compounds **4**, **9**, and **30** indicated that these compounds possess drug-like characteristics and may have potential for oral administration.

In the development of antineoplastic agents for the treatment of GBM, the ability of compounds to cross the BBB and reach the target tumor is of critical importance, as the BBB represents a major barrier to drug delivery to brain tumors and may consequently limit therapeutic efficacy [43]. Based on the QPlogBB values of compounds **4**, **9**, and **30**, which were within the recommended range, these compounds are predicted to have the potential to cross the BBB and reach the target tumor.

## 4. Materials and Methods

### 4.1. Chemistry

#### 4.1.1. Chemicals and Instrumentation

The chemicals used in this work were purchased from Acros Organics (Geel, Belgium), Maybridge (Loughborough, UK), Merck (Darmstadt, Germany), and Sigma-Aldrich (St. Louis, MO, USA). The melting points (M.p.) of the compounds were determined by a Mettler Toledo Melting Point System MP90 (Columbus, OH, USA) and were uncorrected. IR spectra were recorded on an IRPrestige-21 Fourier Transform (FT) IR spectrophotometer (Shimadzu, Tokyo, Japan). ^1^H and ^13^C NMR spectra were recorded on a Bruker FT NMR spectrometer (Billerica, MA, USA) operating at 300 MHz and 75 MHz, respectively. HRMS spectra were recorded on a LCMS-IT-TOF system (Shimadzu, Kyoto, Japan). Thin Layer Chromatography (TLC) was employed to track the progress of all reactions and determine the purity of the synthesized compounds.

#### 4.1.2. General Procedure for the Preparation of Sodium 4-Alkylpiperazine-1-carbodithioate Derivatives

A solution of sodium hydroxide in ethanol was cooled in an ice bath and mixed with the appropriate 1-alkylpiperazine derivative. A solution of carbon disulfide in ethanol was added dropwise to the reaction mixture, which was then stirred at room temperature for 2 h. The reaction progress was monitored by TLC. After completion, as confirmed by TLC, the solvent was removed under reduced pressure using a Rotavapor^®^ R-100 rotary evaporator (Büchi, Flawil, Switzerland). The resulting residue was washed with dry diethyl ether [44].

#### 4.1.3. General Procedure for the Preparation of 2-Chloro-*N*-(aryl)acetamide Derivatives

An aromatic amine (0.1 mol) was dissolved in anhydrous toluene, followed by the addition of an equimolar amount of TEA. An equimolar amount of chloroacetyl chloride was separately dissolved in anhydrous toluene in a dropping funnel. The chloroacetyl chloride solution was added dropwise to the aromatic amine solution under continuous stirring while maintaining the reaction mixture in an ice bath. The progress of the reaction was monitored by TLC. Upon completion of the reaction, the solvent was removed under reduced pressure using a rotary evaporator. The resulting solid product was collected and washed with distilled water [45].

#### 4.1.4. General Procedure for the Preparation of New DTC Derivatives (**1**–**44**)

Equivalent amounts of the sodium salt of *N*,*N*-disubstituted dithiocarbamic acid and 2-chloro-*N*-(aryl)acetamide were stirred in acetone at room temperature for 8 h in the presence of an equivalent amount of potassium carbonate as the catalyst. Upon completion of the reaction, the solvent was removed under reduced pressure. The resulting solid product was washed with water and recrystallized from ethanol.

##### 2-Oxo-2-[(thiazol-2-yl)amino]ethyl 4-(2-methoxyethyl)piperazine-1-carbodithioate (**1**)

White powder. Yield: 85%. M.p.: 198 °C. IR ν_max_ (cm^−1^): 3271.27, 3188.33, 3120.82, 3068.75, 2976.16, 2941.44, 2899.01, 2846.93, 2810.28, 1668.43, 1583.56, 1463.97, 1429.25, 1398.39, 1325.10, 1307.74, 1269.16, 1220.94, 1170.79, 1147.65, 1112.93, 1070.49, 1035.77, 1010.70, 993.34, 964.41, 929.69, 902.69, 879.54, 839.03, 817.82, 777.31, 711.73, 690.52, 651.94, 626.87. ^1^H NMR (300 MHz, DMSO-d_6_): 2.49–2.53 (m, 6H), 3.23 (s, 3H), 3.45 (t, *J* = 5.61, 5.64 Hz, 2H), 3.93 (brs, 2H), 4.18 (brs, 2H), 4.35 (s, 2H), 7.21 (d, *J* = 3.54 Hz, 1H), 7.47 (d, *J* = 3.57 Hz, 1H), 12.40 (s, 1H) (Figure S1). ^13^C NMR (75 MHz, DMSO-d_6_) δ (ppm): 40.08 (CH_2_), 50.26 (CH_2_), 51.56 (CH_2_), 52.82 (CH_2_), 56.75 (2CH_2_), 58.48 (CH_3_), 70.23 (CH_2_), 113.98 (CH), 138.15 (CH), 158.42 (C), 166.25 (C), 194.41 (C) (Figure S2). HRMS (ESI) (*m*/*z*) [M + H]^+^ calcd. for C_13_H_20_N_4_O_2_S_3_: 361.0821, found: 361.0817 (Figure S3).

##### 2-Oxo-2-[(5-nitrothiazol-2-yl)amino]ethyl 4-(2-methoxyethyl)piperazine-1-carbodithioate (**2**)

Light brown powder. Yield: 74%. M.p.: 141.7 °C. IR ν_max_ (cm^−1^): 3234.62, 3095.75, 2949.16, 2879.72, 1678.00, 1537.24, 1469.75, 1427.32, 1348.24, 1303.88, 1290.38, 1273.02, 1234.44, 1201.65, 1170.79, 1134.14, 1107.14, 1039.63, 1028.06, 995.27, 962.48, 930.69, 900.76, 869.90, 842.89, 815.89, 800.46, 734.88, 690.52, 651.94. ^1^H NMR (300 MHz, DMSO-d_6_): 2.49–2.51 (m, 4H), 2.58 (t, *J* = 5.55 Hz, 2H), 3.24 (s, 3H), 3.46 (t, *J* = 5.55, 5.61 Hz, 2H), 3.94 (brs, 2H), 4.18 (brs, 2H), 4.42 (s, 2H), 8.63 (s, 1H), 13.21 (brs, 1H) (Figure S4). ^13^C NMR (75 MHz, DMSO-d_6_) δ (ppm): 40.30 (CH_2_), 50.18 (CH_2_), 51.44 (CH_2_), 52.69 (CH_2_), 56.65 (2CH_2_), 58.49 (CH_3_), 69.98 (CH_2_), 142.00 (C), 143.46 (CH), 162.72 (C), 168.76 (C), 194.17 (C) (Figure S5). HRMS (ESI) (*m*/*z*) [M + H]^+^ calcd. for C_13_H_19_N_5_O_4_S_3_: 406.0672, found: 406.0677 (Figure S6).

##### 2-Oxo-2-[(4-(ethoxycarbonylmethyl)thiazol-2-yl)amino]ethyl 4-(2-methoxyethyl)piperazine-1-carbodithioate (**3**)

White powder. Yield: 84%. M.p.: 102.3 °C. IR ν_max_ (cm^−1^): 3278.99, 3228.84, 3107.32, 2981.95, 2939.52, 2897.08, 2843.07, 2812.21, 1735.93, 1687.71, 1660.71, 1550.77, 1458.18, 1431.18, 1367.53, 1346.31, 1315.45, 1288.45, 1267.23, 1240.23, 1176.58, 1153.43, 1118.71, 1070.49, 1045.42, 1031.92, 999.13, 968.27, 931.62, 873.75, 837.11, 796.60, 717.52, 696.30, 636.51. ^1^H NMR (300 MHz, DMSO-d_6_): 1.18 (t, *J* = 7.08, 7.11 Hz, 3H), 2.50–2.52 (m, 2H), 3.23 (s, 3H), 3.34–3.46 (m, 6H), 3.68 (s, 2H), 3.92 (brs, 2H), 4.08 (q, *J* = 7.08, 7.11 Hz, 2H), 4.17 (brs, 2H), 4.32 (s, 2H), 6.98 (s, 1H), 12.46 (s, 1H) (Figure S7). ^13^C NMR (75 MHz, DMSO-d_6_) δ (ppm): 14.57 (CH_3_), 37.07 (CH_2_), 40.01 (CH_2_), 50.29 (CH_2_), 51.61 (CH_2_), 52.79 (CH_2_), 56.76 (2CH_2_), 58.47 (CH_3_), 60.81 (CH_2_), 70.25 (CH_2_), 110.95 (CH), 144.13 (C), 158.05 (C), 166.32 (C), 170.54 (C), 194.35 (C) (Figure S8). HRMS (ESI) (*m*/*z*) [M + H]^+^ calcd. for C_17_H_26_N_4_O_4_S_3_: 447.1189, found: 447.1185 (Figure S9).

##### 2-Oxo-2-[(benzothiazol-2-yl)amino]ethyl 4-(2-methoxyethyl)piperazine-1-carbodithioate (**4**)

Pale yellow powder. Yield: 86%. M.p.: 164.1 °C. IR ν_max_ (cm^−1^): 3246.20, 3215.34, 3078.39, 3001.24, 2931.80, 2916.37, 2893.22, 2812.21, 1676.14, 1598.99, 1544.98, 1471.69, 1438.90, 1421.54, 1373.32, 1346.31, 1315.45, 1259.52, 1230.58, 1207.44, 1157.29, 1093.64, 1029.99, 987.55, 933.55, 925.83, 879.54, 867.97, 842.89, 831.32, 796.60, 759.95, 731.02, 717.52, 704.02, 673.16, 651.94. ^1^H NMR (300 MHz, DMSO-d_6_): 2.49–2.55 (m, 6H), 3.23 (s, 3H), 3.44 (t, *J* = 2.64, 2.94 Hz, 2H), 3.94 (brs, 2H), 4.18 (brs, 2H), 4.42 (s, 2H), 7.30 (td, *J* = 1.05, 1.08, 7.49, 7.65 Hz, 1H), 7.44 (td, *J* = 1.20, 1.26, 7.20, 8.13 Hz, 1H), 7.75 (d, *J* = 7.92 Hz, 1H), 7.98 (d, *J* = 7.38 Hz, 1H), 12.69 (s, 1H) (Figure S10). ^13^C NMR (75 MHz, DMSO-d_6_) δ (ppm): 40.29 (CH_2_), 50.35 (CH_2_), 51.66 (CH_2_), 52.99 (CH_2_), 56.77 (2CH_2_), 58.48 (CH_3_), 70.26 (CH_2_), 121.04 (CH), 122.22 (CH), 124.05 (CH), 126.63 (CH), 131.84 (C), 148.98 (C), 158.36 (C), 167.43 (C), 194.29 (C) (Figure S11). HRMS (ESI) (*m*/*z*) [M + H]^+^ calcd. for C_17_H_22_N_4_O_2_S_3_: 411.0978, found: 411.0973 (Figure S12).

##### 2-Oxo-2-[(6-fluorobenzothiazol-2-yl)amino]ethyl 4-(2-methoxyethyl)piperazine-1-carbodithioate (**5**)

White powder. Yield: 81%. M.p.: 198.5 °C. IR ν_max_ (cm^−1^): 3251.98. 3126.61, 3068.75, 3043.67, 2983.88, 2912.51, 2843.07, 2814.14, 1703.14, 1608.63, 1558.48, 1543.05, 1469.76, 1458.18, 1438.90, 1411.89, 1379.10, 1303.88, 1265.30, 1244.09, 1219.01, 1199.72, 1147.65, 1112.93, 1072.42, 1049.28, 1028.06, 995.27, 979.84, 964.41, 927.76, 914.26, 877.61, 867.97, 840.96, 806.25, 783.10, 748.38, 705.95, 665.44, 646.15. ^1^H NMR (300 MHz, DMSO-d_6_): 2.49–2.54 (m, 6H), 3.23 (s, 3H), 3.44 (t, *J* = 5.55, 5.67 Hz, 2H), 3.94 (brs, 2H), 4.18 (brs, 2H), 4.42 (s, 2H), 7.29 (td, *J* = 2.67, 2.73, 9.06, 9.09 Hz, 1H), 7.76 (dd, *J* = 4.83, 8.88 Hz, 1H), 7.90 (dd, *J* = 2.67, 8.73 Hz, 1H), 12.72 (s, 1H) (Figure S13). ^13^C NMR (75 MHz, DMSO-d_6_) δ (ppm): 44.99 (CH_2_), 55.08 (CH_2_), 56.38 (CH_2_), 57.55 (CH_2_), 57.71 (CH_2_), 61.50 (CH_2_), 63.23 (CH_3_), 74.97 (CH_2_), 113.44 (d, *J* = 26.25 Hz, CH), 119.48 (d, *J* = 24.00 Hz, CH), 126.90 (d, *J* = 9.0 Hz, CH), 137.85 (d, *J* = 11.25 Hz, C), 150.46 (C), 162.25 and 163.09 (2s, C), 165.43 (C), 172.28 (C), 199.03 (C) (Figure S14). HRMS (ESI) (*m*/*z*) [M + H]^+^ calcd. for C_17_H_21_FN_4_O_2_S_3_: 429.0883, found: 429.0883 (Figure S15).

##### 2-Oxo-2-[(6-chlorobenzothiazol-2-yl)amino]ethyl 4-(2-methoxyethyl)piperazine-1-carbodithioate (**6**)

Off-white powder. Yield: 88%. M.p.: 178.5 °C. IR ν_max_ (cm^−1^): 3248.13, 3111.18, 3057.17, 3034.03, 2943.37, 2912.51, 2837.29, 2812.21, 1707.00, 1595.13, 1550.77, 1533.41, 1471.69, 1433.11, 1398.39, 1379.10, 1301.95, 1265.30, 1217.08, 1147.65, 1112.93, 1099.43, 1053.13, 1026.13, 993.34, 977.91, 964.41, 923.90, 879.54, 844.82, 810.10, 763.81, 746.45, 690.52, 663.51, 642.30. ^1^H NMR (300 MHz, DMSO-d_6_): 2.50–2.55 (m, 6H), 3.23 (s, 3H), 3.44 (t, *J* = 5.43, 5.61 Hz, 2H), 3.93 (brs, 2H), 4.18 (brs, 2H), 4.42 (s, 2H), 7.45 (dd, *J* = 2.13, 2.16, 8.66 Hz, 1H), 7.74 (d, *J* = 8.64 Hz, 1H), 8.13 (d, *J* = 2.04 Hz, 1H), 12.79 (s, 1H) (Figure S16). ^13^C NMR (75 MHz, DMSO-d_6_) δ (ppm): 40.26 (CH_2_), 50.34 (CH_2_), 51.63 (CH_2_), 52.82 (CH_2_), 52.96 (CH_2_), 56.75 (CH_2_), 58.48 (CH_3_), 70.24 (CH_2_), 121.95 (CH), 122.25 (CH), 126.97 (CH), 128.06 (C), 133.57 (C), 147.89 (C), 159.20 (C), 167.66 (C), 194.25 (C) (Figure S17). HRMS (ESI) (*m*/*z*) [M + H]^+^ calcd. for C_17_H_21_ClN_4_O_2_S_3_: 445.0588, found: 445.0584 (Figure S18).

##### 2-Oxo-2-[(6-bromobenzothiazol-2-yl)amino]ethyl 4-(2-methoxyethyl)piperazine-1-carbodithioate (**7**)

White powder. Yield: 89%. M.p.: 158.8 °C. IR ν_max_ (cm^−1^): 3250.05, 3138.18, 3109.25, 3032.10, 2941.44, 2914.44, 2875.86, 2837.29, 2798.71, 1705.07, 1589.34, 1533.41, 1471.69, 1431.18, 1382.96, 1301.95, 1263.37, 1217.08, 1143.79, 1112.93, 1083.99, 1026.13, 993.34, 977.91, 923.90, 877.61, 842.89, 810.10, 742.59, 682.80. ^1^H NMR (300 MHz, DMSO-d_6_): 2.49–2.56 (m, 6H), 3.23 (s, 3H), 3.45 (t, *J* = 5.70, 6.57 Hz, 2H), 3.94 (brs, 2H), 4.18 (brs, 2H), 4.42 (s, 2H), 7.57 (dd, *J* = 2.07, 8.61 Hz, 1H), 7.69 (d, *J* = 8.64 Hz, 1H), 8.26 (d, *J* = 1.98 Hz, 1H), 12.78 (s, 1H) (Figure S19). ^13^C NMR (75 MHz, DMSO-d_6_) δ (ppm): 40.26 (CH_2_), 50.32 (CH_2_), 51.71 (CH_2_), 52.99 (CH_2_), 56.76 (2CH_2_), 58.48 (CH_3_), 70.25 (CH_2_), 115.96 (C), 122.66 (CH), 124.79 (CH), 129.65 (CH), 134.06 (C), 148.19 (C), 159.18 (C), 167.68 (C), 194.24 (C) (Figure S20). HRMS (ESI) (*m*/*z*) [M + H]^+^ calcd. for C_17_H_21_BrN_4_O_2_S_3_: 489.0083, found: 489.0089 (Figure S21).

##### 2-Oxo-2-[(6-methylbenzothiazol-2-yl)amino]ethyl 4-(2-methoxyethyl)piperazine-1-carbodithioate (**8**)

Off-white powder. Yield: 80%. M.p.: 197.5 °C. IR ν_max_ (cm^−1^): 3244.27, 3124.68, 3053.32, 2947.23, 2910.58, 2839.22, 2810.28, 1703.14, 1604.77, 1556.55, 1541.12, 1471.69, 1431.18, 1379.10, 1303.88, 1265.30, 1217.08, 1155.36, 1120.64, 1068.56, 1028.06, 993.34, 977.91, 964.41, 925.83, 906.54, 877.61, 808.17, 746.45, 667.37, 644.22. ^1^H NMR (300 MHz, DMSO-d_6_): 2.40 (s, 3H), 2.49–2.55 (m, 6H), 3.23 (s, 3H), 3.45 (t, *J* = 5.43, 5.64 Hz, 2H), 3.94 (brs, 2H), 4.18 (brs, 2H), 4.41 (s, 2H), 7.25 (dd, *J* = 1.29, 1.38, 8.30 Hz, 1H), 7.63 (d, *J* = 8.22 Hz, 1H), 7.76 (brs, 1H), 12.60 (s, 1H) (Figure S22). ^13^C NMR (75 MHz, DMSO-d_6_) δ (ppm): 21.45 (CH_3_), 40.29 (CH_2_), 50.36 (CH_2_), 51.65 (CH_2_), 52.97 (CH_2_), 56.76 (2CH_2_), 58.48 (CH_3_), 70.25 (CH_2_), 120.67 (CH), 121.80 (CH), 127.94 (CH), 131.99 (C), 133.49 (C), 146.95 (C), 157.47 (C), 167.25 (C), 194.32 (C) (Figure S23). HRMS (ESI) (*m*/*z*) [M + H]^+^ calcd. for C_18_H_24_N_4_O_2_S_3_: 425.1134, found: 425.1143 (Figure S24).

##### 2-Oxo-2-[(6-methoxybenzothiazol-2-yl)amino]ethyl 4-(2-methoxyethyl)piperazine-1-carbodithioate (**9**)

Beige powder. Yield: 81%. M.p.: 72.8 °C. IR ν_max_ (cm^−1^): 3282.84, 3165.19, 3066.82, 2922.16, 2837.29, 1672.28, 1606.70, 1556.55, 1469.76, 1431.18, 1375.25, 1365.60, 1319.31, 1255.66, 1224.80, 1166.93, 1139.93, 1114.86, 1056.99, 1020.34, 991.41, 950.91, 925.83, 902.69, 825.53, 734.88, 700.16, 665.44. ^1^H NMR (300 MHz, DMSO-d_6_): 2.49–2.55 (m, 6H), 3.23 (s, 3H), 3.44 (t, *J* = 5.49 Hz, 2H), 3.80 (s, 3H), 3.93 (brs, 2H), 4.18 (brs, 2H), 4.40 (s, 2H), 7.02 (dd, *J* = 2.61, 2.64, 8.84 Hz, 1H), 7.57 (d, *J* = 2.58 Hz, 1H), 7.64 (d, *J* = 8.85 Hz, 1H), 12.55 (s, 1H) (Figure S25). ^13^C NMR (75 MHz, DMSO-d_6_) δ (ppm): 40.25 (CH_2_), 50.31 (CH_2_), 51.66 (CH_2_), 52.96 (CH_2_), 56.06 (CH_3_), 56.75 (2CH_2_), 58.48 (CH_3_), 70.23 (CH_2_), 105.13 (CH), 115.42 (CH), 121.64 (CH), 133.16 (C), 143.04 (C), 156.29 (C), 156.57 (C), 167.09 (C), 194.34 (C) (Figure S26). HRMS (ESI) (*m*/*z*) [M + H]+ calcd. for C_18_H_24_N_4_O_3_S_3_: 441.1083, found: 441.1093 (Figure S27).

##### 2-Oxo-2-[(6-ethoxybenzothiazol-2-yl)amino]ethyl 4-(2-methoxyethyl)piperazine-1-carbodithioate (**10**)

Off-white powder. Yield: 82%. M.p.: 118.7 °C. IR ν_max_ (cm^−1^): 3292.49, 3140.11, 3066.82, 2974.23, 2927.94, 2895.15, 2839.22, 1668.43, 1604.77, 1566.20, 1556.55, 1458.18, 1431.18, 1365.60, 1311.59, 1276.88, 1255.66, 1232.51, 1219.01, 1176.58, 1157.29, 1109.07, 1083.99, 1056.99, 1039.63, 1014.56, 991.41, 975.98, 956.69, 921.97, 871.82, 840.96, 821.68, 786.96, 736.81, 700.16, 665.44. ^1^H NMR (300 MHz, DMSO-d_6_): 1.34 (t, *J* = 6.93, 6.99 Hz, 3H), 2.49–2.55 (m, 6H), 3.23 (s, 3H), 3.45 (t, *J* = 5.61 Hz, 2H), 3.94 (brs, 2H), 4.05 (q, *J* = 6.93, 6.96 Hz, 2H), 4.18 (brs, 2H), 4.40 (s, 2H), 7.01 (dd, *J* = 2.55, 2.58, 8.84 Hz, 1H), 7.55 (d, *J* = 2.49 Hz, 1H), 7.63 (d, *J* = 8.82 Hz, 1H), 12.54 (s, 1H) (Figure S28). ^13^C NMR (75 MHz, DMSO-d_6_) δ (ppm): 15.17 (CH_3_), 40.25 (CH_2_), 50.34 (CH_2_), 51.68 (CH_2_), 52.83 (CH_2_), 56.76 (2CH_2_), 58.48 (CH_3_), 64.01 (CH_2_), 70.25 (CH_2_), 105.75 (CH), 115.74 (CH), 121.63 (CH), 133.15 (C), 142.96 (C), 155.81 (C), 156.25 (C), 167.07 (C), 194.33 (C) (Figure S29). HRMS (ESI) (*m*/*z*) [M + H]^+^ calcd. for C_19_H_26_N_4_O_3_S_3_: 455.1240, found: 455.1238 (Figure S30).

##### 2-Oxo-2-[(6-nitrobenzothiazol-2-yl)amino]ethyl 4-(2-methoxyethyl)piperazine-1-carbodithioate (**11**)

Light brown powder. Yield: 81%. M.p.: 196.7 °C. IR ν_max_ (cm^−1^): 3250.05, 3167.12, 3068, 75, 2987.74, 2943.37, 2891.30, 2827.64, 1708.93, 1606.70, 1573.91, 1539.20, 1504.48, 1460.11, 1444.68, 1433.11, 1381.03, 1344.38, 1303.88, 1282.66, 1271.09, 1219.01, 1153.43, 1128.36, 1103.28, 1066.64, 1035.77, 1010.70, 995.27, 979.84, 958.62, 927.76, 900.76, 891.11, 827.46, 788.89, 750.31, 740.67, 719.45, 686.66. ^1^H NMR (300 MHz, DMSO-d_6_): 2.50–2.55 (m, 6H), 3.23 (s, 3H), 3.45 (t, *J* = 5.55, 5.61 Hz, 2H), 3.94 (brs, 2H), 4.18 (brs, 2H), 4.46 (s, 2H), 7.91 (d, *J* = 8.94 Hz, 1H), 8.28 (dd, *J* = 2.37, 2.40, 8.96 Hz, 1H), 9.06 (d, *J* = 2.31 Hz, 1H), 13.08 (s, 1H) (Figure S31). ^13^C NMR (75 MHz, DMSO-d_6_) δ (ppm): 40.32 (CH_2_), 50.38 (CH_2_), 51.67 (CH_2_), 52.80 (CH_2_), 52.95 (CH_2_), 56.75 (CH_2_), 58.48 (CH_3_), 70.23 (CH_2_), 119.58 (CH), 121.10 (CH), 122.28 (CH), 132.61 (C), 143.42 (C), 153.96 (C), 163.92 (C), 168.27 (C), 194.17 (C) (Figure S32). HRMS (ESI) (*m*/*z*) [M + H]^+^ calcd. for C_17_H_21_N_5_O_4_S_3_: 456.0828, found: 456.0829 (Figure S33).

##### 2-Oxo-2-[(thiazol-2-yl)amino]ethyl 4-(2-ethoxyethyl)piperazine-1-carbodithioate (**12**)

White powder. Yield: 90%. M.p.: 176.0 °C. IR ν_max_ (cm^−1^): 3255.84, 3186.40, 3126.61, 3070.68, 2976.16, 2937.59, 2895.15, 2860.43, 2837.29, 1672.28, 1581.63, 1496.76, 1460.11, 1423.47, 1396.46, 1325.10, 1269.16, 1244.09, 1220.94, 1170.79, 1118.71, 1062.78, 1037.70, 1014.56, 991.41, 968.27, 929.69, 904.61, 887.26, 875.68, 837.11, 817.82, 775.38, 707.88, 688.59, 655.80, 623.01. ^1^H NMR (300 MHz, DMSO-d_6_): 1.10 (t, *J* = 6.99 Hz, 3H), 2.49–2.54 (m, 6H), 3.41 (q, *J* = 6.99, 7.02 Hz, 2H), 3.49 (t, *J* = 5.73, 5.76 Hz, 2H), 3.93 (brs, 2H), 4.18 (brs, 2H), 4.36 (s, 2H), 7.21 (d, *J* = 3.54 Hz, 1H), 7.47 (d, *J* = 3.57 Hz, 1H), 12.41 (s, 1H) (Figure S34). ^13^C NMR (75 MHz, DMSO-d_6_) δ (ppm): 15.61 (CH_3_), 40.08 (CH_2_), 50.26 (CH_2_), 51.56 (CH_2_), 52.85 (CH_2_), 56.98 (2CH_2_), 65.93 (CH_2_), 68.07 (CH_2_), 113.98 (CH), 138.14 (CH), 158.42 (C), 166.24 (C), 194.41 (C) (Figure S35). HRMS (ESI) (*m*/*z*) [M + H]^+^ calcd. for C_14_H_22_N_4_O_2_S_3_: 375.0978, found: 375.0975 (Figure S36).

##### 2-Oxo-2-[(5-nitrothiazol-2-yl)amino]ethyl 4-(2-ethoxyethyl)piperazine-1-carbodithioate (**13**)

Light brown powder. Yield: 88%. M.p.: 115.0 °C. IR ν_max_ (cm^−1^): 3234.62, 3138.18, 3095.75, 2972.31, 2949.16, 2879.72, 2837.29, 1680.00, 1537.27, 1469.76, 1427.32, 1348.24, 1303.88, 1290.38, 1273.02, 1234.44, 1201.65, 1170.79, 1134.14, 1107.14, 1039.63, 1028.06, 995.27, 962.48, 931.62, 900.76, 869.90, 842.89, 815.89, 800.46, 734.88, 682.80, 651.94, 628.79. ^1^H NMR (300 MHz, DMSO-d_6_): 1.10 (t, *J* = 6.99 Hz, 3H), 2.49–2.51 (m, 4H), 2.58–2.60 (m, 2H), 3.42 (q, *J* = 6.99, 7.02 Hz, 2H), 3.50 (t, *J* = 5.67, 5.76 Hz, 2H), 3.95 (brs, 2H), 4.18 (brs, 2H), 4.42 (s, 2H), 8.63 (s, 1H), 13.09 (brs, 1H) (Figure S37). ^13^C NMR (75 MHz, DMSO-d_6_) δ (ppm): 15.59 (CH_3_), 40.30 (CH_2_), 50.17 (CH_2_), 51.42 (CH_2_), 52.84 (CH_2_), 56.88 (2CH_2_), 65.95 (CH_2_), 67.84 (CH_2_), 142.07 (C), 143.43 (CH), 162.70 (C), 168.75 (C), 194.18 (C) (Figure S38). HRMS (ESI) (*m*/*z*) [M + H]^+^ calcd. for C_14_H_21_N_5_O_4_S_3_: 420.0828, found: 420.0824 (Figure S39).

##### 2-Oxo-2-[(4-(ethoxycarbonylmethyl)thiazol-2-yl)amino]ethyl 4-(2-ethoxyethyl)piperazine-1-carbodithioate (**14**)

White powder. Yield: 85%. M.p.: 72.0 °C. IR ν_max_ (cm^−1^): 3288.63, 3138.18, 3089.96, 2978.09, 2918.30, 2891.30, 2864.29, 2827.64, 2806.43, 1734.01, 1685.79, 1660.71, 1548.84, 1458.18, 1429.25, 1367.53, 1317.38, 1290.38, 1267.23, 1232.51, 1149.57, 1118.71, 1105.21, 1035.77, 995.27, 960.55, 931.62, 873.75, 835.18, 798.53, 738.74, 665.44. ^1^H NMR (300 MHz, DMSO-d_6_): 1.10 (t, *J* = 6.99 Hz, 3H), 1.18 (t, *J* = 7.08, 7.11 Hz, 3H), 2.49–2.55 (m, 6H), 3.41 (q, *J* = 6.96, 6.99, 7.05 Hz, 2H), 3.48 (t, *J* = 5.70, 5.79 Hz, 2H), 3.68 (s, 2H), 3.93 (brs, 2H), 4.08 (q, *J* = 7.11 Hz, 2H), 4.18 (brs, 2H), 4.32 (s, 2H), 6.98 (s, 1H), 12.45 (s, 1H) (Figure S40). ^13^C NMR (75 MHz, DMSO-d_6_) δ (ppm): 14.57 (CH_3_), 15.61 (CH_3_), 37.08 (CH_2_), 40.04 (CH_2_), 50.31 (CH_2_), 51.60 (CH_2_), 52.99 (CH_2_), 57.01 (2CH_2_), 60.80 (CH_2_), 65.92 (CH_2_), 68.12 (CH_2_), 110.94 (CH), 144.13 (C), 158.05 (C), 166.32 (C), 170.53 (C), 194.36 (C) (Figure S41). HRMS (ESI) (*m*/*z*) [M + H]^+^ calcd. for C_18_H_28_N_4_O_4_S_3_: 461.1345, found: 461.1341 (Figure S42).

##### 2-Oxo-2-[(benzothiazol-2-yl)amino]ethyl 4-(2-ethoxyethyl)piperazine-1-carbodithioate (**15**)

Off-white powder. Yield: 89%. M.p.: 132.0 °C. IR ν_max_ (cm^−1^): 3255.84, 3169.04, 3051.39, 2945.30, 2900.94, 2858.51, 2806.43, 1685.79, 1651.07, 1597.06, 1558.48, 1469.76, 1454.33, 1438.90, 1427.32, 1354.03, 1311.59, 1267.23, 1228.66, 1205.51, 1168.86, 1149.57, 1118.71, 1101.35, 1014.56, 995.27, 975.98, 927.76, 869.90, 806.25, 758.02, 731.02, 677.01, 638.44. ^1^H NMR (300 MHz, DMSO-d_6_): 1.10 (t, *J* = 6.99 Hz, 3H), 2.49–2.55 (m, 6H), 3.41 (q, *J* = 6.99, 7.26 Hz, 2H), 3.48 (t, *J* = 5.67, 5.76, 2H), 3.94 (brs, 2H), 4.18 (brs, 2H), 4.42 (s, 2H), 7.30 (td, *J* = 1.08, 1.11, 7.50, 7.64 Hz, 1H), 7.44 (td, *J* = 1.02, 1.23, 1.29, 7.22, 8.12 Hz, 1H), 7.75 (d, *J* = 7.92 Hz, 1H), 7.97 (d, *J* = 7.32 Hz, 1H), 12.66 (brs, 1H) (Figure S43). ^13^C NMR (75 MHz, DMSO-d_6_) δ (ppm): 15.61 (CH_3_), 40.31 (CH_2_), 50.33 (CH_2_), 51.66 (CH_2_), 52.86 (CH_2_), 57.01 (2CH_2_), 65.92 (CH_2_), 68.13 (CH_2_), 121.03 (CH), 122.20 (CH), 124.04 (CH), 126.62 (CH), 131.85 (C), 148.98 (C), 158.35 (C), 167.42 (C), 194.30 (C) (Figure S44). HRMS (ESI) (*m*/*z*) [M + H]^+^ calcd. for C_18_H_24_N_4_O_2_S_3_: 425.1134, found: 425.1133 (Figure S45).

##### 2-Oxo-2-[(6-fluorobenzothiazol-2-yl)amino]ethyl 4-(2-ethoxyethyl)piperazine-1-carbodithioate (**16**)

Off-white powder. Yield: 88%. M.p.: 140.0 °C. IR ν_max_ (cm^−1^): 3296.35, 3140.11, 3066.82, 2964.59, 2927.94, 2889.37, 2848.86, 1676.14, 1606.70, 1560.41, 1456.26, 1438.90, 1357.89, 1317.38, 1276.88, 1257.59, 1230.58, 1207.44, 1178.51, 1161.15, 1116.78, 1041.56, 1010.70, 993.34, 977.91, 927.76, 912.33, 887.26, 871.82, 846.75, 823.60, 813.96, 794.67, 734.88, 702.09, 663.51. ^1^H NMR (300 MHz, DMSO-d_6_): 1.10 (t, *J* = 6.99 Hz, 3H), 2.49–2.54 (m, 6H), 3.37–3.44 (m, 2H), 3.48 (t, *J* = 5.70, 5.79 Hz, 2H), 3.94 (brs, 2H), 4.18 (brs, 2H), 4.42 (s, 2H), 7.29 (td, *J* = 2.67, 2.73, 9.06, 9.09 Hz, 1H), 7.76 (dd, *J* = 4.80, 4.83, 8.90 Hz, 1H), 7.89 (dd, *J* = 2.67, 8.73 Hz, 1H), 12.69 (brs, 1H) (Figure S46). ^13^C NMR (75 MHz, DMSO-d_6_) δ (ppm): 15.61 (CH_3_), 40.20 (CH_2_), 50.36 (CH_2_), 51.66 (CH_2_), 52.85 (CH_2_), 57.01 (2CH_2_), 65.92 (CH_2_), 68.13 (CH_2_), 108.67 (d, *J* = 27.00 Hz, CH), 114.73 (d, *J* = 24.75 Hz, CH), 122.15 (d, *J* = 9.75 Hz, CH), 133.11 (d, *J* = 11.25 Hz, C), 145.72 (C), 157.51 and 158.37 (2s, C), 160.68 (C), 167.53 (C), 194.27 (C) (Figure S47). HRMS (ESI) (*m*/*z*) [M + H]^+^ calcd. for C_18_H_23_FN_4_O_2_S_3_: 443.1040, found: 443.1052 (Figure S48).

##### 2-Oxo-2-[(6-chlorobenzothiazol-2-yl)amino]ethyl 4-(2-ethoxyethyl)piperazine-1-carbodithioate (**17**)

Off-white powder. Yield: 86%. M.p.: 138.0 °C. IR ν_max_ (cm^−1^): 3265.49, 3163.26, 3059.10, 2970.38, 2924.09, 2881.65, 2858.51, 1712.79, 1593.20, 1556.55, 1458.18, 1427.32, 1369.46, 1301.95, 1263.37, 1224.80, 1199.72, 1147.65, 1111.00, 1093.64, 1070.49, 1035.77, 1008.77, 995.27, 972.12, 939.33, 908.47, 887.26, 871.82, 842.89, 808.17, 763.81, 734.88, 690.52, 665.44, 653.87. ^1^H NMR (300 MHz, DMSO-d_6_): 1.09 (t, *J* = 6.99 Hz, 3H), 2.51–2.54 (m, 6H), 3.37–3.44 (m, 2H), 3.48 (t, *J* = 5.73, 5.76 Hz, 2H), 3.94 (brs, 2H), 4.18 (brs, 2H), 4.42 (s, 2H), 7.45 (dd, *J* = 2.19, 8.64 Hz, 1H), 7.74 (d, *J* = 8.64 Hz, 1H), 8.12 (d, *J* = 2.10 Hz, 1H), 12.76 (brs, 1H) (Figure S49). ^13^C NMR (75 MHz, DMSO-d_6_) δ (ppm): 15.61 (CH_3_), 40.47 (CH_2_), 50.35 (CH_2_), 51.67 (CH_2_), 52.85 (CH_2_), 57.00 (2CH_2_), 65.92 (CH_2_), 68.12 (CH_2_), 121.93 (CH), 122.24 (CH), 126.96 (CH), 128.06 (C), 133.57 (C), 147.89 (C), 159.20 (C), 167.65 (C), 194.24 (C) (Figure S50). HRMS (ESI) (*m*/*z*) [M + H]^+^ calcd. for C_18_H_23_ClN_4_O_2_S_3_: 459.0744, found: 459.0754 (Figure S51).

##### 2-Oxo-2-[(6-bromobenzothiazol-2-yl)amino]ethyl 4-(2-ethoxyethyl)piperazine-1-carbodithioate (**18**)

Pale yellow shiny powder. Yield: 89%. M.p.: 88.0 °C. IR ν_max_ (cm^−1^): 3273.20, 3159.40, 3118.90, 3074.53, 2976.16, 2922.16, 2891.30, 2875.86, 2845.00, 1678.07, 1593.20, 1550.77, 1485.19, 1463.97, 1436.97, 1377.17, 1361.74, 1319.31, 1273.02, 1255.66, 1232.51, 1207.44, 1161.15, 1149.57, 1105.21, 1078.21, 1062.78, 1041.56, 1020.34, 1001.06, 974.05, 943.19, 908.47, 867.97, 839.03, 825.53, 800.46, 775.38, 744.52, 731.02, 682.80, 655.80. ^1^H NMR (300 MHz, DMSO-d_6_): 1.10 (t, *J* = 6.99, 7.02 Hz, 3H), 2.49–2.55 (m, 6H), 3.37–3.44 (m, 2H), 3.48 (t, *J* = 5.58, 5.82 Hz, 2H), 3.93 (brs, 2H), 4.18 (brs, 2H), 4.42 (s, 2H), 7.57 (dd, *J* = 2.04, 2.07, 8.60 Hz, 1H), 7.69 (d, *J* = 8.64 Hz, 1H), 8.26 (d, *J* = 1.98 Hz, 1H), 12.77 (brs, 1H) (Figure S52). ^13^C NMR (75 MHz, DMSO-d_6_) δ (ppm): 15.61 (CH_3_), 40.19 (CH_2_), 50.38 (CH_2_), 51.66 (CH_2_), 52.98 (CH_2_), 57.00 (2CH_2_), 65.93 (CH_2_), 68.12 (CH_2_), 115.96 (C), 122.66 (CH), 124.78 (CH), 129.65 (CH), 134.07 (C), 148.19 (C), 159.17 (C), 167.68 (C), 194.24 (C) (Figure S53). HRMS (ESI) (*m*/*z*) [M + H]^+^ calcd. for C_18_H_23_BrN_4_O_2_S_3_: 503.0239, found: 503.0244 (Figure S54).

##### 2-Oxo-2-[(6-methylbenzothiazol-2-yl)amino]ethyl 4-(2-ethoxyethyl)piperazine-1-carbodithioate (**19**)

Beige powder. Yield: 85%. M.p.: 163.0 °C. IR ν_max_ (cm^−1^): 3250.05, 3126.61, 3049.46, 2970.38, 2916.37, 2850.79, 2798.71, 1697.36, 1604.77, 1539.20, 1473.62, 1427.32, 1382.96, 1359.82, 1342.46, 1305.81, 1286.52, 1263.37, 1219.01, 1209.37, 1157.29, 1147.65, 1112.93, 1060.85, 1028.06, 1016.49, 999.13, 979.84, 954.76, 904.61, 881.47, 829.39, 808.17, 746.45, 705.95, 667.37, 634.58. ^1^H NMR (300 MHz, DMSO-d_6_): 1.10 (t, *J* = 6.96, 7.02 Hz, 3H), 2.40 (s, 3H), 2.49–2.55 (m, 6H), 3.41 (q, *J* = 6.96, 7.02 Hz, 2H), 3.48 (t, *J* = 5.67, 5.79 Hz, 2H), 3.94 (brs, 2H), 4.18 (brs, 2H), 4.41 (s, 2H), 7.25 (dd, *J* = 1.29, 1.32, 8.30 Hz, 1H), 7.63 (d, *J* = 8.22 Hz, 1H), 7.76 (brs, 1H), 12.59 (brs, 1H) (Figure S55). ^13^C NMR (75 MHz, DMSO-d_6_) δ (ppm): 15.62 (CH_3_), 21.45 (CH_3_), 40.20 (CH_2_), 50.36 (CH_2_), 51.65 (CH_2_), 52.87 (CH_2_), 56.36 (CH_2_), 57.01 (CH_2_), 65.92 (CH_2_), 68.13 (CH_2_), 120.67 (CH), 121.79 (CH), 127.93 (CH), 131.99 (C), 133.49 (C), 146.95 (C), 157.47 (C), 167.25 (C), 194.30 (C) (Figure S56). HRMS (ESI) (*m*/*z*) [M + H]^+^ calcd. for C_19_H_26_N_4_O_2_S_3_: 439.1291, found: 439.1301 (Figure S57).

##### 2-Oxo-2-[(6-methoxybenzothiazol-2-yl)amino]ethyl 4-(2-ethoxyethyl)piperazine-1-carbodithioate (**20**)

Light tan powder. Yield: 86%. M.p.: 108.0 °C. IR ν_max_ (cm^−1^): 3282.84, 3138.18, 3064.89, 2966.52, 2927.94, 2885.51, 2846.93, 1672.28, 1602.85, 1556.55, 1465.90, 1429.25, 1365.60, 1330.88, 1311.59, 1276.88, 1255.66, 1222.87, 1209.37, 1176.58, 1159.22, 1118.71, 1105.21, 1056.99, 1041.56, 1026.13, 1012.63, 991.41, 975.98, 925.83, 902.69, 883.40, 873.75, 840.96, 819.75, 790.81, 736.81, 700.16, 663.51, 626.87. ^1^H NMR (300 MHz, DMSO-d_6_): 1.10 (t, *J* = 6.99 Hz, 3H), 2.49–2.55 (m, 6H), 3.41 (q, *J* = 6.99 Hz, 2H), 3.49 (t, *J* = 5.76 Hz, 5.82 Hz, 2H), 3.80 (s, 3H), 3.94 (brs, 2H), 4.18 (brs, 2H), 4.40 (s, 2H), 7.03 (dd, *J* = 2.61 Hz, 8.85 Hz, 1H), 7.57 (d, *J* = 2.58 Hz, 1H), 7.64 (d, *J* = 8.85 Hz, 1H), 12.54 (brs, 1H) (Figure S58). ^13^C NMR (75 MHz, DMSO-d_6_) δ (ppm): 15.61 (CH_3_), 40.20 (CH_2_), 50.29 (CH_2_), 51.64 (CH_2_), 52.84 (CH_2_), 56.06 (CH_3_), 57.00 (2CH_2_), 65.93 (CH_2_), 68.12 (CH_2_), 105.14 (CH), 115.42 (CH), 121.64 (CH), 133.17 (C), 143.05 (C), 156.29 (C), 156.58 (C), 167.08 (C), 194.33 (C) (Figure S59). HRMS (ESI) (*m*/*z*) [M + H]^+^ calcd. for C_19_H_26_N_4_O_3_S_3_: 455.1240, found: 455.1246 (Figure S60).

##### 2-Oxo-2-[(6-ethoxybenzothiazol-2-yl)amino]ethyl 4-(2-ethoxyethyl)piperazine-1-carbodithioate (**21**)

Off-white powder. Yield: 85%. M.p.: 74.0 °C. IR ν_max_ (cm^−1^): 3280.92, 3149.76, 3068.75, 2972.31, 2927.94, 2877.79, 2845.00, 1672.28, 1604.77, 1568.13, 1558.48, 1460.11, 1436.97, 1371.39, 1359.82, 1321.24, 1274.95, 1253.73, 1232.51, 1213.23, 1165.00, 1149.57, 1103.28, 1055.06, 1039.63, 1020.34, 999.13, 975.98, 941.26, 918.12, 908.47, 883.40, 869.90, 829.39, 819.75, 798.53, 773.46, 732.95, 700.16, 661.58. ^1^H NMR (300 MHz, DMSO-d_6_): 1.10 (t, *J* = 6.99 Hz, 3H), 1.34 (t, *J* = 6.93, 6.99 Hz, 3H), 2.50–2.55 (m, 6H), 3.41 (q, *J* = 6.99, 7.02 Hz, 2H), 3.48 (t, *J* = 5.70, 5.79 Hz, 2H), 3.94 (brs, 2H), 4.05 (q, *J* = 6.90, 6.96, 6.99 Hz, 2H), 4.18 (brs, 2H), 4.40 (s, 2H), 7.01 (dd, *J* = 2.58, 8.82 Hz, 1H), 7.55 (d, *J* = 2.52 Hz, 1H), 7.63 (d, *J* = 8.82 Hz, 1H), 12.52 (brs, 1H) (Figure S61). ^13^C NMR (75 MHz, DMSO-d_6_) δ (ppm): 15.17 (CH_3_), 15.62 (CH_3_), 40.20 (CH_2_), 50.34 (CH_2_), 51.65 (CH_2_), 52.86 (CH_2_), 57.01 (2CH_2_), 64.01 (CH_2_), 65.92 (CH_2_), 68.13 (CH_2_), 105.75 (CH), 115.74 (CH), 121.63 (CH), 133.16 (C), 142.97 (C), 155.81 (C), 156.25 (C), 167.06 (C), 194.32 (C) (Figure S62). HRMS (ESI) (*m*/*z*) [M + H]^+^ calcd. for C_20_H_28_N_4_O_3_S_3_: 469.1396, found: 469.1401 (Figure S63).

##### 2-Oxo-2-[(6-nitrobenzothiazol-2-yl)amino]ethyl 4-(2-ethoxyethyl)piperazine-1-carbodithioate (**22**)

Light yellow powder. Yield: 85%. M.p.: 90.0 °C. IR ν_max_ (cm^−1^): 3205.69, 3151.69, 3097.68, 3022.45, 2972.31, 2926.01, 2881.65, 2823.79, 1680.00, 1606.70, 1571.99, 1537.27, 1508.33, 1471.69, 1444.68, 1431.18, 1379.10, 1334.74, 1305.81, 1276.88, 1257.59, 1234.44, 1226.73, 1209.37, 1153.43, 1124.50, 1103.28, 1078.21, 1041.56, 1022.27, 999.13, 979.84, 916.19, 904.61, 887.26, 848.68, 831.32, 750.31, 734.88, 723.31, 682.80, 657.73. ^1^H NMR (300 MHz, DMSO-d_6_): 1.10 (t, *J* = 6.99, 7.02 Hz, 3H), 2.49–2.55 (m, 6H), 3.41 (q, *J* = 6.99, 7.02 Hz, 2H), 3.49 (t, *J* = 5.67, 5.79 Hz, 2H), 3.94 (brs, 2H), 4.18 (brs, 2H), 4.46 (s, 2H), 7.91 (d, *J* = 8.97 Hz, 1H), 8.28 (dd, *J* = 2.43, 2.46, 8.96 Hz, 1H), 9.06 (d, *J* = 2.40 Hz, 1H), 13.04 (brs, 1H) (Figure S64). ^13^C NMR (75 MHz, DMSO-d_6_) δ (ppm): 15.61 (CH_3_), 40.32 (CH_2_), 50.40 (CH_2_), 51.66 (CH_2_), 52.84 (CH_2_), 56.99 (2CH_2_), 65.93 (CH_2_), 68.10 (CH_2_), 119.58 (CH), 121.11 (CH), 122.28 (CH), 132.61 (C), 143.42 (C), 153.96 (C), 163.91 (C), 168.27 (C), 194.16 (C) (Figure S65). HRMS (ESI) (*m*/*z*) [M + H]^+^ calcd. for C_18_H_23_N_5_O_4_S_3_: 470.0985, found: 470.0987 (Figure S66).

##### 2-Oxo-2-[(thiazol-2-yl)amino]ethyl 4-[2-(2-hydroxyethoxy)ethyl]piperazine-1-carbodithioate (**23**)

White powder. Yield: 79%. M.p.: 168.1 °C. IR ν_max_ (cm^−1^): 3454.51, 3377.36, 3255.84, 3180.62, 3097.68, 3043.67, 2920.23, 2848.86, 2733.13, 1701.22, 1571.99, 1477.47, 1427.32, 1365.60, 1348.24, 1319.31, 1273.02, 1265.30, 1232.51, 1209.37, 1170.79, 1157.29, 1139.93, 1111.00, 1074.35, 1056.99, 1026.13, 1012.63, 991.41, 966.34, 939.33, 923.90, 881.47, 831.32, 806.25, 763.81, 711.73, 680.87, 617.22. ^1^H NMR (300 MHz, DMSO-d_6_): 2.49–2.55 (m, 6H), 3.40–3.42 (m, 2H), 3.47–3.49 (m, 2H), 3.53 (t, *J* = 5.76 Hz, 2H), 3.93 (brs, 2H), 4.18 (brs, 2H), 4.36 (s, 2H), 4.62 (brs, 1H), 7.21 (d, *J* = 3.57 Hz, 1H), 7.47 (d, *J* = 3.57 Hz, 1H), 12.40 (s, 1H) (Figure S67). ^13^C NMR (75 MHz, DMSO-d_6_) δ (ppm): 40.10 (CH_2_), 50.29 (CH_2_), 51.63 (CH_2_), 52.97 (CH_2_), 56.98 (2CH_2_), 60.66 (CH_2_), 68.70 (CH_2_), 72.69 (CH_2_), 113.97 (CH), 138.14 (CH), 158.44 (C), 166.25 (C), 194.40 (C) (Figure S68). HRMS (ESI) (*m*/*z*) [M + H]^+^ calcd. for C_14_H_22_N_4_O_3_S_3_: 391.0927, found: 391.0920 (Figure S69).

##### 2-Oxo-2-[(5-nitrothiazol-2-yl)amino]ethyl 4-[2-(2-hydroxyethoxy)ethyl]piperazine-1-carbodithioate (**24**)

Brown powder. Yield: 76%. M.p.: 143.1 °C. IR ν_max_ (cm^−1^): 3329.14, 3226.91, 3116.97, 3062.96, 3016.67, 2918.30, 2854.65, 2833.43, 2690.70, 1676.14, 1552.70, 1514.12, 1483.26, 1460.11, 1427.32, 1388.75, 1342.46, 1319.31, 1298.09, 1274.95, 1247.94, 1226.73, 1197.79, 1166.93, 1145.72, 1118.71, 1095.57, 1060.85, 1022.27, 991.41, 966.34, 925.83, 889.18, 823.60, 810.10, 771.53, 732.95, 717.52, 673.16, 634.58. ^1^H NMR (300 MHz, DMSO-d_6_): 2.57–2.61 (m, 6H), 3.41–3.43 (m, 2H), 3.47–3.49 (m, 2H), 3.55 (t, *J* = 5.64, 5.67 Hz, 2H), 3.95 (brs, 2H), 4.18 (brs, 2H), 4.42 (s, 2H), 4.62 (brs, 1H), 8.62 (s, 1H), 13.08 (s, 1H) (Figure S70). ^13^C NMR (75 MHz, DMSO-d_6_) δ (ppm): 40.37 (CH_2_), 50.18 (CH_2_), 51.43 (CH_2_), 52.78 (CH_2_), 56.86 (2CH_2_), 60.65 (CH_2_), 68.45 (CH_2_), 72.69 (CH_2_), 141.95 (C), 143.44 (CH), 162.83 (C), 168.80 (C), 194.22 (C) (Figure S71). HRMS (ESI) (*m*/*z*) [M + H]^+^ calcd. for C_14_H_21_N_5_O_5_S_3_: 436.0778, found: 436.0767 (Figure S72).

##### 2-Oxo-2-[(4-(ethoxycarbonylmethyl)thiazol-2-yl)amino]ethyl 4-[2-(2-hydroxyethoxy)ethyl]piperazine-1-carbodithioate (**25**)

White powder. Yield: 77%. M.p.: 189.4 °C. IR ν_max_ (cm^−1^): 3437.15, 3305.99, 3169.04, 3099.61, 2922.16, 2872.01, 2823.79, 1728.22, 1681.93, 1556.55, 1427.32, 1371.39, 1309.67, 1273.02, 1228.66, 1149.57, 1111.00, 1062.78, 1016.49, 995.27, 925.83, 837.11, 738.74, 667.37, 646.15. ^1^H NMR (300 MHz, DMSO-d_6_): 1.18 (t, *J* = 7.08, 7.11 Hz, 3H), 2.49–2.55 (m, 6H), 3.47–3.49 (m, 2H), 3.51–3.55 (m, 4H), 3.68 (s, 2H), 3.92 (brs, 2H), 4.08 (q, *J* = 7.11 Hz, 2H), 4.18 (brs, 2H), 4.33 (s, 2H), 4.62 (brs, 1H), 6.98 (s, 1H), 12.44 (s, 1H) (Figure S73). ^13^C NMR (75 MHz, DMSO-d_6_) δ (ppm): 14.57 (CH_3_), 37.09 (CH_2_), 40.04 (CH_2_), 50.30 (CH_2_), 51.62 (CH_2_), 52.89 (CH_2_), 56.98 (2CH_2_), 60.67 (CH_2_), 60.80 (CH_2_), 68.69 (CH_2_), 72.68 (CH_2_), 110.93 (CH), 144.14 (C), 158.05 (C), 166.32 (C), 170.52 (C), 194.38 (C) (Figure S74). HRMS (ESI) (*m*/*z*) [M + H]^+^ calcd. for C_18_H_28_N_4_O_5_S_3_: 477.1295, found: 477.1298 (Figure S75).

##### 2-Oxo-2-[(benzothiazol-2-yl)amino]ethyl 4-[2-(2-hydroxyethoxy)ethyl]piperazine-1-carbodithioate (**26**)

Off-white powder. Yield: 77%. M.p.: 169.6 °C. IR ν_max_ (cm^−1^): 3402.43, 3255.84, 3180.62, 3051.39, 2920.23, 2848.86, 1685.79, 1597.06, 1558.48, 1469.76, 1454.33, 1438.90, 1427.32, 1354.03, 1311.59, 1267.23, 1228.66, 1205.51, 1168.86, 1149.57, 1118.71, 1101.35, 1014.56, 995.27, 975.98, 927.76, 869.90, 806.25, 758.02, 731.02, 680.87. ^1^H NMR (300 MHz, DMSO-d_6_): 2.49–2.56 (m, 6H), 3.41–3.42 (m, 2H), 3.47–3.49 (m, 2H), 3.53 (t, *J* = 5.76, 5.79 Hz, 2H), 3.94 (brs, 2H), 4.18 (brs, 2H), 4.43 (s, 2H), 4.62 (brs, 1H), 7.30 (td, *J* = 0.75, 1.11, 7.23, 7.89 Hz, 1H), 7.44 (td, *J* = 1.08, 1.23, 1.29, 7.19, 8.15 Hz, 1H), 7.75 (d, *J* = 7.95 Hz, 1H), 7.97 (d, *J* = 7.29 Hz, 1H), 12.64 (brs, 1H) (Figure S76). ^13^C NMR (75 MHz, DMSO-d_6_) δ (ppm): 40.33 (CH_2_), 50.39 (CH_2_), 51.69 (CH_2_), 52.97 (CH_2_), 56.98 (2CH_2_), 60.67 (CH_2_), 68.71 (CH_2_), 72.69 (CH_2_), 121.03 (CH), 122.19 (CH), 124.04 (CH), 126.61 (CH), 131.87 (C), 148.99 (C), 158.36 (C), 167.42 (C), 194.32 (C) (Figure S77). HRMS (ESI) (*m*/*z*) [M + H]^+^ calcd. for C_18_H_24_N_4_O_3_S_3_: 441.1083, found: 441.1090 (Figure S78).

##### 2-Oxo-2-[(6-fluorobenzothiazol-2-yl)amino]ethyl 4-[2-(2-hydroxyethoxy)ethyl]piperazine-1-carbodithioate (**27**)

White powder. Yield: 79%. M.p.: 163.7 °C. IR ν_max_ (cm^−1^): 3250.05, 3172.90, 3138.18, 3062.96, 2970.38, 2916.37, 2854.65, 1689.64, 1608.63, 1556.55, 1454.33, 1419.61, 1379.10, 1303.88, 1273.02, 1249.87, 1232.51, 1201.65, 1139.93, 1111.00, 1066.64, 1049.28, 1018.41, 995.27, 983.70, 933.55, 912.33, 891.11, 848.68, 825.53, 804.32, 777.31, 746.45, 702.09, 661.58. ^1^H NMR (300 MHz, DMSO-d_6_): 2.50–2.56 (m, 6H), 3.39–3.42 (m, 2H), 3.47–3.49 (m, 2H), 3.53 (t, *J* = 5.73, 5.79 Hz, 2H), 3.94 (brs, 2H), 4.18 (brs, 2H), 4.42 (s, 2H), 4.62 (brs, 1H), 7.29 (td, *J* = 2.64, 2.67, 2.70, 9.06, 9.08 Hz, 1H), 7.76 (dd, *J* = 4.80, 4.83, 8.90 Hz, 1H), 7.89 (dd, *J* = 2.64, 2.67, 8.72 Hz, 1H), 12.69 (brs, 1H) (Figure S79). ^13^C NMR (75 MHz, DMSO-d_6_) δ (ppm): 40.23 (CH_2_), 50.35 (CH_2_), 51.68 (CH_2_), 52.96 (CH_2_), 56.96 (2CH_2_), 60.67 (CH_2_), 68.67 (CH_2_), 72.69 (CH_2_), 108.66 (d, *J* = 27.00 Hz, CH), 114.72 (d, *J* = 24.00 Hz, CH), 122.14 (d, *J* = 9.00 Hz, CH), 133.12 (d, *J* = 11.25 Hz, C), 145.73 (C), 157.51 and 158.34 (C), 160.69 (C), 167.52 (C), 194.30 (C) (Figure S80). HRMS (ESI) (*m*/*z*) [M + H]^+^ calcd. for C_18_H_23_FN_4_O_3_S_3_: 459.0989, found: 459.0994 (Figure S81).

##### 2-Oxo-2-[(6-chlorobenzothiazol-2-yl)amino]ethyl 4-[2-(2-hydroxyethoxy)ethyl]piperazine-1-carbodithioate (**28**)

Off-white powder. Yield: 84%. M.p.: 167.1 °C. IR ν_max_ (cm^−1^): 3402.43, 3182.55, 3066.82, 2983.88, 2933.73, 2862.36, 2806.43, 1689.64, 1602.85, 1564.27, 1469.76, 1444.68, 1433.11, 1352.10, 1321.24, 1261.45, 1246.02, 1234.44, 1190.08, 1165.00, 1149.57, 1118.71, 1111.00, 1097.50, 1074.35, 1051.20, 1041.56, 1008.77, 997.20, 981.77, 952.84, 902.69, 867.97, 858.32, 831.32, 813.96, 802.39, 761.88, 734.88, 688.59, 657.73, 648.08. ^1^H NMR (300 MHz, DMSO-d_6_): 2.50–2.54 (m, 6H), 3.41–3.42 (m, 2H), 3.49–3.53 (m, 4H), 3.94 (brs, 2H), 4.18 (brs, 2H), 4.43 (s, 2H), 4.61 (brs, 1H), 7.46 (d, *J* = 8.19 Hz, 1H), 7.75 (d, *J* = 8.55 Hz, 1H), 8.12 (s, 1H), 12.76 (brs, 1H) (Figure S82). ^13^C NMR (75 MHz, DMSO-d_6_) δ (ppm): 40.23 (CH_2_), 50.40 (CH_2_), 51.69 (CH_2_), 52.87 (CH_2_), 56.98 (2CH_2_), 60.67 (CH_2_), 68.70 (CH_2_), 72.69 (CH_2_), 121.94 (CH), 122.25 (CH), 126.96 (CH), 128.06 (C), 133.58 (C), 147.90 (C), 159.21 (C), 167.66 (C), 194.26 (C) (Figure S83). HRMS (ESI) (*m*/*z*) [M + H]^+^ calcd. for C_18_H_23_ClN_4_O_3_S_3_: 475.0694, found: 475.0693 (Figure S84).

##### 2-Oxo-2-[(6-bromobenzothiazol-2-yl)amino]ethyl 4-[2-(2-hydroxyethoxy)ethyl]piperazine-1-carbodithioate (**29**)

White powder. Yield: 85%. M.p.: 171.6 °C. IR ν_max_ (cm^−1^): 3402.43, 3174.83, 3064.89, 2964.59, 2935.66, 2904.80, 2858.51, 2806.43, 1687.71, 1598.99, 1562.34, 1469.76, 1442.75, 1435.04, 1319.31, 1301.95, 1259.52, 1246.02, 1234.44, 1190.08, 1165.00, 1149.57, 1111.00, 1083.99, 1074.35, 1051.20, 1035.77, 1008.77, 997.20, 983.70, 952.84, 904.61, 869.90, 858.32, 831.32, 813.96, 800.46, 763.81, 736.81, 682.80. ^1^H NMR (300 MHz, DMSO-d_6_): 2.49–2.56 (m, 6H), 3.40–3.42 (m, 2H), 3.47–3.49 (m, 2H), 3.53 (t, *J* = 5.76 Hz, 2H), 3.94 (brs, 2H), 4.18 (brs, 2H), 4.42 (s, 2H), 4.62 (brs, 1H), 7.57 (dd, *J* = 2.04, 8.61 Hz, 1H), 7.69 (d, *J* = 8.61 Hz, 1H), 8.25 (d, *J* = 1.98 Hz, 1H), 12.77 (brs, 1H) (Figure S85). ^13^C NMR (75 MHz, DMSO-d_6_) δ (ppm): 40.22 (CH_2_), 50.41 (CH_2_), 51.67 (CH_2_), 52.88 (CH_2_), 56.97 (2CH_2_), 60.66 (CH_2_), 68.70 (CH_2_), 72.69 (CH_2_), 115.96 (C), 122.65 (CH), 124.77 (CH), 129.64 (CH), 134.07 (C), 148.19 (C), 159.18 (C), 167.67 (C), 194.25 (C) (Figure S86). HRMS (ESI) (*m*/*z*) [M + H]^+^ calcd. for C_18_H_23_BrN_4_O_3_S_3_: 519.0188, found: 519.0197 (Figure S87).

##### 2-Oxo-2-[(6-methylbenzothiazol-2-yl)amino]ethyl 4-[2-(2-hydroxyethoxy)ethyl]piperazine-1-carbodithioate (**30**)

Beige powder. Yield: 84%. M.p.: 67.4 °C. IR ν_max_ (cm^−1^): 3404.36, 3194.12, 3053.32, 2916.37, 2854.65, 1683.86, 1608.63, 1568.13, 1556.55, 1460.11, 1435.04, 1373.32, 1352.10, 1317.38, 1261.45, 1234.44, 1224.80, 1205.51, 1161.15, 1141.86, 1111.00, 1053.13, 1041.56, 999.13, 981.77, 945.12, 904.61, 887.26, 869.90, 812.03, 804.32, 777.31, 761.88, 742.59, 704.02, 663.51. ^1^H NMR (300 MHz, DMSO-d_6_): 2.40 (s, 3H), 2.49–2.56 (m, 6H), 3.40–3.42 (m, 2H), 3.47–3.49 (m, 2H), 3.53 (t, *J* = 5.76 Hz, 2H), 3.94 (brs, 2H), 4.18 (brs, 2H), 4.41 (s, 2H), 4.62 (brs, 1H), 7.25 (dd, *J* = 1.17, 1.26, 8.27, 1H), 7.63 (d, *J* = 8.25 Hz, 1H), 7.75–7.76 (m, 1H), 12.58 (brs, 1H) (Figure S88). ^13^C NMR (75 MHz, DMSO-d_6_) δ (ppm): 21.45 (CH_3_), 40.30 (CH_2_), 50.36 (CH_2_), 51.67 (CH_2_), 52.99 (CH_2_), 56.98 (2CH_2_), 60.66 (CH_2_), 68.69 (CH_2_), 72.69 (CH_2_), 120.67 (CH), 121.79 (CH), 127.94 (CH), 132.00 (C), 133.49 (C), 146.95 (C), 157.47 (C), 167.25 (C), 194.33 (C) (Figure S89). HRMS (ESI) (*m*/*z*) [M + H]^+^ calcd. for C_19_H_26_N_4_O_3_S_3_: 455.1240, found: 455.1257 (Figure S90).

##### 2-Oxo-2-[(6-methoxybenzothiazol-2-yl)amino]ethyl 4-[2-(2-hydroxyethoxy)ethyl]piperazine-1-carbodithioate (**31**)

White powder. Yield: 74%. M.p.: 137.9 °C. IR ν_max_ (cm^−1^): 3404.36, 3282.84, 3064.89, 2966.52, 2885.51, 1672.28, 1602.85, 1556.55, 1465.90, 1429.25, 1392.61, 1363.67, 1317.38, 1286.52, 1257.59, 1224.80, 1151.50, 1107.14, 1058.92, 1035.77, 1012.63, 1001.06, 991.41, 975.98, 925.83, 902.69, 883.40, 873.75, 840.96, 819.75, 790.81, 736.81, 700.16, 663.51. ^1^H NMR (300 MHz, DMSO-d_6_): 2.49–2.56 (m, 6H), 3.41–3.42 (m, 2H), 3.47–3.48 (m, 2H), 3.53 (t, *J* = 5.73, 5.76 Hz, 2H), 3.80 (s, 3H), 3.93 (brs, 2H), 4.18 (brs, 2H), 4.40 (s, 2H), 4.62 (brs, 1H), 7.03 (dd, *J* = 2.61, 8.85 Hz, 1H), 7.57 (d, *J* = 2.58 Hz, 1H), 7.64 (d, *J* = 8.82 Hz, 1H), 12.53 (brs, 1H) (Figure S91). ^13^C NMR (75 MHz, DMSO-d_6_) δ (ppm): 40.22 (CH_2_), 50.35 (CH_2_), 51.68 (CH_2_), 53.02 (CH_2_), 56.05 (CH_3_), 56.98 (2CH_2_), 60.66 (CH_2_), 68.70 (CH_2_), 72.69 (CH_2_), 105.15 (CH), 115.42 (CH), 121.64 (CH), 133.18 (C), 143.06 (C), 150.12 (C), 156.29 (C), 167.08 (C), 194.34 (C) (Figure S92). HRMS (ESI) (*m*/*z*) [M + H]^+^ calcd. for C_19_H_26_N_4_O_4_S_3_: 471.1189, found: 471.1192 (Figure S93).

##### 2-Oxo-2-[(6-ethoxybenzothiazol-2-yl)amino]ethyl 4-[2-(2-hydroxyethoxy)ethyl]piperazine-1-carbodithioate (**32**)

White powder. Yield: 85%. M.p.: 149.4 °C. IR ν_max_ (cm^−1^): 3415.93, 3190.26, 3080.32, 2920.23, 2806.43, 1705.07, 1681.93, 1604.77, 1573.91, 1556.55, 1460.11, 1431.18, 1392.61, 1363.67, 1317.38, 1286.52, 1257.59, 1224.80, 1151.50, 1107.14, 1058.92, 1035.77, 1012.63, 1001.06, 981.77, 941.26, 925.83, 891.11, 869.90, 852.54, 810.10, 763.81, 736.81. ^1^H NMR (300 MHz, DMSO-d_6_): 1.34 (t, *J* = 6.90, 6.99 Hz, 3H), 2.49–2.56 (m, 6H), 3.41–3.42 (m, 2H), 3.47–3.49 (m, 2H), 3.53 (t, *J* = 5.76 Hz, 2H), 3.94 (brs, 2H), 4.06 (q, *J* = 6.93, 6.96 Hz, 2H), 4.18 (brs, 2H), 4.40 (s, 2H), 4.62 (brs, 1H), 7.01 (dd, *J* = 2.58, 8.82 Hz, 1H), 7.55 (d, *J* = 2.52 Hz, 1H), 7.63 (d, *J* = 8.82 Hz, 1H), 12.52 (brs, 1H) (Figure S94). ^13^C NMR (75 MHz, DMSO-d_6_) δ (ppm): 15.17 (CH_3_), 40.22 (CH_2_), 50.31 (CH_2_), 51.68 (CH_2_), 52.98 (CH_2_), 56.98 (2CH_2_), 60.66 (CH_2_), 64.03 (CH_2_), 68.70 (CH_2_), 72.69 (CH_2_), 105.78 (CH), 115.75 (CH), 121.63 (CH), 133.17 (C), 142.98 (C), 155.82 (C), 156.26 (C), 167.07 (C), 194.34 (C) (Figure S95). HRMS (ESI) (*m*/*z*) [M + H]^+^ calcd. for C_20_H_28_N_4_O_4_S_3_: 485.1345, found: 485.1355 (Figure S96).

##### 2-Oxo-2-[(6-nitrobenzothiazol-2-yl)amino]ethyl 4-[2-(2-hydroxyethoxy)ethyl]piperazine-1-carbodithioate (**33**)

Beige powder. Yield: 81%. M.p.: 155.1 °C. IR ν_max_ (cm^−1^): 3423.65, 3261.63, 3097.68, 2958.80, 2927.94, 2891.30, 2846.93, 1678.07, 1608.63, 1573.91, 1537.27, 1508.33, 1473.62, 1442.75, 1431.18, 1377.17, 1336.67, 1303.88, 1276.88, 1259.52, 1236.37, 1222.87, 1153.43, 1136.07, 1124.50, 1107.14, 1083.99, 1062.78, 1043.49, 1020.34, 997.20, 981.77, 923.90, 904.61, 889.18, 831.32, 752.24, 734.88, 723.31, 682.80. ^1^H NMR (300 MHz, DMSO-d_6_): 2.50–2.56 (m, 6H), 3.39–3.42 (m, 2H), 3.47–3.49 (m, 2H), 3.53 (t, *J* = 5.58, 5.79 Hz, 2H), 3.94 (brs, 2H), 4.18 (brs, 2H), 4.46 (s, 2H), 4.69 (brs, 1H), 7.90 (d, *J* = 8.94 Hz, 1H), 8.28 (dd, *J* = 2.31, 8.94 Hz, 1H), 9.05 (d, *J* = 2.22 Hz, 1H), 13.09 (brs, 1H) (Figure S97). ^13^C NMR (75 MHz, DMSO-d_6_) δ (ppm): 40.33 (CH_2_), 50.39 (CH_2_), 51.68 (CH_2_), 52.99 (CH_2_), 56.95 (2CH_2_), 60.66 (CH_2_), 68.65 (CH_2_), 72.69 (CH_2_), 119.56 (CH), 121.10 (CH), 122.27 (CH), 132.62 (C), 143.43 (C), 153.96 (C), 163.91 (C), 168.26 (C), 194.19 (C) (Figure S98). HRMS (ESI) (*m*/*z*) [M + H]^+^ calcd. for C_18_H_23_N_5_O_5_S_3_: 486.0934, found: 486.0936 (Figure S99).

##### 2-Oxo-2-[(thiazol-2-yl)amino]ethyl 4-(2-morpholinoethyl)piperazine-1-carbodithioate (**34**)

White powder. Yield: 76%. M.p.: 180.7 °C. IR ν_max_ (cm^−1^): 3257.77, 3174.83, 3072.60, 2951.09, 2922.16, 2889.37, 2852.72, 2818.00, 1674.21, 1573.91, 1475.54, 1431.18, 1390.68, 1355.96, 1319.31, 1311.59, 1300.02, 1265.30, 1244.09, 1219.01, 1182.36, 1168.86, 1114.86, 1062.78, 1033.85, 1002.98, 993.34, 966.34, 929.69, 912.33, 885.33, 867.97, 812.03, 771.53, 717.52, 686.66. ^1^H NMR (300 MHz, DMSO-d_6_): 2.37–2.50 (m, 12H), 3.55 (t, *J* = 4.53 Hz, 4H), 3.92 (brs, 2H), 4.17 (brs, 2H), 4.35 (s, 2H), 7.21 (d, *J* = 3.54 Hz, 1H), 7.47 (d, *J* = 3.54 Hz, 1H), 12.40 (brs, 1H) (Figure S100). ^13^C NMR (75 MHz, DMSO-d_6_) δ (ppm): 40.09 (CH_2_), 50.31 (CH_2_), 51.64 (CH_2_), 52.96 (CH_2_), 54.10 (2CH_2_), 54.68 (CH_2_), 56.22 (2CH_2_), 66.65 (2CH_2_), 113.98 (CH), 138.15 (CH), 158.44 (C), 166.25 (C), 194.37 (C) (Figure S101). HRMS (ESI) (*m*/*z*) [M + H]^+^ calcd. for C_16_H_25_N_5_O_2_S_3_: 416.1243, found: 416.1261 (Figure S102).

##### 2-Oxo-2-[(5-nitrothiazol-2-yl)amino]ethyl 4-(2-morpholinoethyl)piperazine-1-carbodithioate (**35**)

Mustard-colored powder. Yield: 75%. M.p.: 193.2 °C. IR ν_max_ (cm^−1^): 3068.75, 3008.95, 2926.01, 2821.86, 1705.07, 1583.56, 1492.90, 1462.04, 1409.96, 1369.46, 1344.38, 1276.88, 1255.66, 1222.87, 1153.43, 1136.07, 1087.85, 1066.64, 1053.13, 1028.06, 995.27, 966.34, 939.33, 927.76, 914.26, 860.25, 825.53, 804.32, 756.10, 740.67, 725.23, 665.44. ^1^H NMR (300 MHz, DMSO-d_6_): 2.50–2.62 (m, 12H), 3.61 (t, *J* = 4.59, 4.65 Hz, 4H), 3.94 (brs, 2H), 4.18 (brs, 2H), 4.37 (s, 2H), 8.58 (s, 1H), 13.04 (brs, 1H) (Figure S103). ^13^C NMR (75 MHz, DMSO-d_6_) δ (ppm): 41.27 (CH_2_), 50.18 (CH_2_), 51.47 (CH_2_), 52.68 (CH_2_), 53.61 (2CH_2_), 53.84 (CH_2_), 55.39 (2CH_2_), 65.99 (2CH_2_), 140.90 (C), 144.07 (CH), 164.92 (C), 170.01 (C), 194.49 (C) (Figure S104). HRMS (ESI) (*m*/*z*) [M + H]^+^ calcd. for C_16_H_24_N_6_O_4_S_3_: 461.1094, found: 461.1091 (Figure S105).

##### 2-Oxo-2-[(4-(ethoxycarbonylmethyl)thiazol-2-yl)amino]ethyl 4-(2-morpholinoethyl)piperazine-1-carbodithioate (**36**)

White powder. Yield: 80%. M.p.: 112.1 °C. IR ν_max_ (cm^−1^): 3250.05, 3180.62, 3064.89, 2980.02, 2949.16, 2895.15, 2810.28, 1730.15, 1674.21, 1564.27, 1467.83, 1431.18, 1388.75, 1361.74, 1317.38, 1296.16, 1263.37, 1222.87, 1141.86, 1124.50, 1107.14, 1066.64, 1033.85, 997.20, 956.69, 914.26, 862.18, 802.39, 769.60, 736.81, 663.51. ^1^H NMR (300 MHz, DMSO-d_6_): 1.18 (t, *J* = 7.11 Hz, 3H), 2.36–2.51 (m, 12H), 3.55 (t, *J* = 4.59, 4.62 Hz, 4H), 3.68 (s, 2H), 3.92 (brs, 2H), 4.08 (q, *J* = 7.08, 7.11 Hz, 2H), 4.17 (brs, 2H), 4.32 (s, 2H), 6.98 (s, 1H), 12.45 (s, 1H) (Figure S106). ^13^C NMR (75 MHz, DMSO-d_6_) δ (ppm): 14.57 (CH_3_), 37.08 (CH_2_), 40.03 (CH_2_), 50.31 (CH_2_), 51.63 (CH_2_), 52.83 (CH_2_), 54.10 (2CH_2_), 54.67 (CH_2_), 56.22 (2CH_2_), 60.80 (CH_2_), 66.65 (2CH_2_), 110.94 (CH), 144.13 (C), 158.06 (C), 166.32 (C), 170.52 (C), 194.35 (C) (Figure S107). HRMS (ESI) (*m*/*z*) [M + H]^+^ calcd. for C_20_H_31_N_5_O_4_S_3_: 502.1611, found: 502.1621 (Figure S108).

##### 2-Oxo-2-[(benzothiazol-2-yl)amino]ethyl 4-(2-morpholinoethyl)piperazine-1-carbodithioate (**37**)

Off-white powder. Yield: 80%. M.p.: 186.7 °C. IR ν_max_ (cm^−1^): 3250.05, 3219.19, 3066.82, 2989.66, 2945.30, 2910.58, 2819.93, 1691.57, 1595.13, 1531.48, 1490.97, 1471.69, 1448.54, 1436.97, 1390.68, 1363.67, 1334.74, 1294.24, 1271.09, 1259.52, 1226.73, 1176.58, 1163.08, 1139.93, 1114.86, 1068.56, 1029.99, 1012.63, 989.48, 979.84, 927.76, 912.33, 866.04, 844.82, 806.25, 765.74, 729.09, 704.02, 669.30, 661.58. ^1^H NMR (300 MHz, DMSO-d_6_): 2.37–2.51 (m, 12H), 3.55 (t, *J* = 4.59 Hz, 4H), 3.93 (brs, 2H), 4.18 (brs, 2H), 4.43 (s, 2H), 7.30 (td, *J* = 1.05, 1.08, 7.55, 7.59 Hz, 1H), 7.44 (td, *J* = 1.20, 1.26, 7.59, 7.71 Hz, 1H), 7.75 (d, *J* = 7.95 Hz, 1H), 7.97 (d, *J* = 7.41 Hz, 1H), 12.63 (brs, 1H) (Figure S109). ^13^C NMR (75 MHz, DMSO-d_6_) δ (ppm): 40.33 (CH_2_), 50.35 (CH_2_), 51.69 (CH_2_), 52.91 (CH_2_), 54.09 (2CH_2_), 54.66 (CH_2_), 56.21 (2CH_2_), 66.64 (2CH_2_), 121.03 (CH), 122.19 (CH), 124.04 (CH), 126.61 (CH), 131.86 (C), 148.99 (C), 158.37 (C), 167.42 (C), 194.29 (C) (Figure S110). HRMS (ESI) (*m*/*z*) [M + H]^+^ calcd. for C_20_H_27_N_5_O_2_S_3_: 466.1400, found: 466.1408 (Figure S111).

##### 2-Oxo-2-[(6-fluorobenzothiazol-2-yl)amino]ethyl 4-(2-morpholinoethyl)piperazine-1-carbodithioate (**38**)

White powder. Yield: 79%. M.p.: 192.4 °C. IR ν_max_ (cm^−1^): 3201.83, 3072.60, 2995.45, 2954.95, 2933.73, 2839.22, 2816.07, 1685.79, 1604.77, 1544.98, 1494.83, 1471.69, 1450.47, 1442.75, 1386.82, 1359.82, 1332.81, 1292.31, 1271.09, 1259.52, 1242.16, 1224.80, 1197.79, 1174.65, 1163.08, 1147.65, 1120.64, 1103.28, 1070.49, 1045.42, 1026.13, 1010.70, 987.55, 977.91, 927.76, 912.33, 883.40, 867.97, 846.75, 825.53, 813.96, 802.39, 767.67, 736.81, 682.80. ^1^H NMR (300 MHz, DMSO-d_6_): 2.36–2.51 (m, 12H), 3.55 (t, *J* = 4.59, 4.62 Hz, 4H), 3.93 (brs, 2H), 4.17 (brs, 2H), 4.42 (s, 2H), 7.29 (td, *J* = 2.67, 2.73, 9.06, 9.09 Hz, 1H), 7.76 (dd, *J* = 4.80, 4.83, 8.90 Hz, 1H), 7.89 (dd, *J* = 2.67, 8.70 Hz, 1H), 12.69 (brs, 1H) (Figure S112). ^13^C NMR (75 MHz, DMSO-d_6_) δ (ppm): 40.28 (CH_2_), 50.35 (CH_2_), 51.69 (CH_2_), 52.96 (CH_2_), 54.09 (2CH_2_), 54.65 (CH_2_), 56.21 (2CH_2_), 66.63 (2CH_2_), 108.66 (d, *J* = 27.00 Hz, CH), 114.71 (d, *J* = 24.00 Hz, CH), 122.14 (d, *J* = 9.00 Hz, CH), 133.12 (d, *J* = 11.25 Hz, C), 145.73 (C), 157.50 and 158.40 (2s, C), 160.68 (C), 167.53 (C), 194.27 (C) (Figure S113). HRMS (ESI) (*m*/*z*) [M + H]^+^ calcd. for C_20_H_26_FN_5_O_2_S_3_: 484.1305, found: 484.1314 (Figure S114).

##### 2-Oxo-2-[(6-chlorobenzothiazol-2-yl)amino]ethyl 4-(2-morpholinoethyl)piperazine-1-carbodithioate (**39**)

White powder. Yield: 81%. M.p.: 203.2 °C. IR ν_max_ (cm^−1^): 3201.83, 3068.75, 2953.02, 2906.73, 2856.58, 2816.07, 1687.71, 1597.06, 1539.20, 1489.05, 1471.69, 1448.54, 1435.04, 1398.39, 1386.82, 1357.89, 1332.81, 1292.31, 1271.09, 1259.52, 1224.80, 1178.51, 1163.08, 1145.72, 1116.78, 1099.43, 1064.71, 1049.28, 1029.99, 1010.70, 987.55, 979.84, 927.76, 912.33, 893.04, 867.97, 813.96, 804.32, 759.95, 736.81, 684.73. ^1^H NMR (300 MHz, DMSO-d_6_): 2.38–2.50 (m, 12H), 3.55 (t, *J* = 4.14, 4.17 Hz, 4H), 3.93 (brs, 2H), 4.17 (brs, 2H), 4.42 (s, 2H), 7.45 (dd, *J* = 1.92, 1.98, 8.61 Hz, 1H), 7.74 (d, *J* = 8.64 Hz, 1H), 8.12 (d, *J* = 1.80 Hz, 1H), 12.73 (brs, 1H) (Figure S115). ^13^C NMR (75 MHz, DMSO-d_6_) δ (ppm): 40.32 (CH_2_), 50.37 (CH_2_), 51.67 (CH_2_), 52.85 (CH_2_), 54.09 (2CH_2_), 54.64 (CH_2_), 56.20 (2CH_2_), 66.63 (2CH_2_), 121.93 (CH), 122.23 (CH), 126.95 (CH), 128.05 (C), 133.58 (C), 147.91 (C), 159.24 (C), 167.66 (C), 194.24 (C) (Figure S116). HRMS (ESI) (*m*/*z*) [M + H]^+^ calcd. for C_20_H_26_ClN_5_O_2_S_3_: 500.1010, found: 500.0997 (Figure S117).

##### 2-Oxo-2-[(6-bromobenzothiazol-2-yl)amino]ethyl 4-(2-morpholinoethyl)piperazine-1-carbodithioate (**40**)

White powder. Yield: 82%. M.p.: 199.6 °C. IR ν_max_ (cm^−1^): 3201.83, 3066.82, 2951.09, 2816.07, 1687.71, 1593.20, 1537.27, 1489.05, 1473.62, 1448.54, 1433.11, 1386.82, 1355.96, 1332.81, 1292.31, 1273.02, 1257.59, 1224.80, 1182.36, 1163.08, 1143.79, 1116.78, 1083.99, 1070.49, 1047.35, 1029.99, 1010.70, 987.55, 979.84, 927.76, 912.33, 893.04, 867.97, 812.03, 769.60, 736.81, 678.94. ^1^H NMR (300 MHz, DMSO-d_6_): 2.37–2.51 (m, 12H), 3.54 (t, *J* = 4.56 Hz, 4.59 Hz, 4H), 3.93 (brs, 2H), 4.17 (brs, 2H), 4.42 (s, 2H), 7.55 (dd, *J* = 2.04, 8.61 Hz, 1H), 7.68 (d, *J* = 8.61 Hz, 1H), 8.24 (d, *J* = 2.01 Hz, 1H), 12.74 (brs, 1H) (Figure S118). ^13^C NMR (75 MHz, DMSO-d_6_) δ (ppm): 40.33 (CH_2_), 50.36 (CH_2_), 51.68 (CH_2_), 52.94 (CH_2_), 54.07 (2CH_2_), 54.63 (CH_2_), 56.18 (2CH_2_), 66.62 (2CH_2_), 115.96 (C), 122.64 (CH), 124.75 (CH), 129.63 (CH), 134.08 (C), 148.20 (C), 159.20 (C), 167.67 (C), 194.24 (C) (Figure S119). HRMS (ESI) (*m*/*z*) [M + H]^+^ calcd. for C_20_H_26_BrN_5_O_2_S_3_: 544.0505, found: 544.0509 (Figure S120).

##### 2-Oxo-2-[(6-methylbenzothiazol-2-yl)amino]ethyl 4-(2-morpholinoethyl)piperazine-1-carbodithioate (**41**)

Beige powder. Yield: 79%. M.p.: 193.4 °C. IR ν_max_ (cm^−1^): 3223.05, 3008.95, 2949.16, 2908.65, 2883.58, 2819.93, 1687.71, 1606.70, 1564.27, 1543.05, 1489.05, 1471.69, 1448.54, 1388.75, 1355.96, 1332.81, 1292.31, 1273.02, 1259.52, 1226.43, 1211.30, 1163.08, 1141.86, 1126.43, 1114.86, 1062.78, 1029.99, 1010.70, 987.55, 979.84, 927.76, 912.33, 896.90, 867.97, 812.03, 767.67, 736.81, 663.51. ^1^H NMR (300 MHz, DMSO-d_6_): 2.37–2.51 (m, 15H), 3.55 (t, *J* = 4.53, 4.59 Hz, 4H), 3.93 (brs, 2H), 4.17 (brs, 2H), 4.41 (s, 2H), 7.24 (dd, *J* = 1.26, 1.29, 8.30 Hz, 1H), 7.63 (d, *J* = 8.22 Hz, 1H), 7.75 (s, 1H), 12.55 (brs, 1H) (Figure S121). ^13^C NMR (75 MHz, DMSO-d_6_) δ (ppm): 21.46 (CH_3_), 40.32 (CH_2_), 50.33 (CH_2_), 51.67 (CH_2_), 52.94 (CH_2_), 54.07 (2CH_2_), 54.63 (CH_2_), 56.18 (2CH_2_), 66.61 (2CH_2_), 120.67 (CH), 121.78 (CH), 127.92 (CH), 132.00 (C), 133.48 (C), 146.96 (C), 157.47 (C), 167.23 (C), 194.30 (C) (Figure S122). HRMS (ESI) (*m*/*z*) [M + H]^+^ calcd. for C_21_H_29_N_5_O_2_S_3_: 480.1556, found: 480.1554 (Figure S123).

##### 2-Oxo-2-[(6-methoxybenzothiazol-2-yl)amino]ethyl 4-(2-morpholinoethyl)piperazine-1-carbodithioate (**42**)

Pale yellow powder. Yield: 81%. M.p.: 132.9 °C. IR ν_max_ (cm^−1^): 3250.05, 3142.04, 3061.03, 2989.66, 2960.73, 2931.80, 2860.43, 2825.72, 2804.50, 1685.79, 1602.85, 1568.13, 1554.63, 1463.97, 1427.32, 1381.03, 1334.74, 1305.81, 1294.24, 1257.59, 1224.80, 1161.15, 1138.00, 1118.71, 1105.21, 1058.92, 1029.99, 1012.63, 981.77, 923.90, 908.47, 885.33, 850.61, 815.89, 806.25, 783.10, 750.31, 700.16, 665.44. ^1^H NMR (300 MHz, DMSO-d_6_): 2.37–2.51 (m, 12H), 3.55 (t, *J* = 4.59 Hz, 4H), 3.80 (s, 3H), 3.93 (brs, 2H), 4.18 (brs, 2H), 4.40 (s, 2H), 7.02 (dd, *J* = 8.85 Hz, 1H), 7.57 (d, *J* = 2.55 Hz, 1H), 7.64 (d, *J* = 8.85 Hz, 1H), 12.53 (brs, 1H) (Figure S124). ^13^C NMR (75 MHz, DMSO-d_6_) δ (ppm): 40.28 (CH_2_), 50.34 (CH_2_), 51.66 (CH_2_), 52.95 (CH_2_), 54.09 (2CH_2_), 54.65 (CH_2_), 56.07 (CH_3_), 56.20 (2CH_2_), 66.63 (2CH_2_), 105.14 (CH), 115.41 (CH), 121.64 (CH), 133.18 (C), 143.05 (C), 156.29 (C), 156.58 (C), 167.08 (C), 194.31 (C) (Figure S125). HRMS (ESI) (*m*/*z*) [M + H]^+^ calcd. for C_21_H_29_N_5_O_3_S_3_: 496.1505, found: 496.1502 (Figure S126).

##### 2-Oxo-2-[(6-ethoxybenzothiazol-2-yl)amino]ethyl 4-(2-morpholinoethyl)piperazine-1-carbodithioate (**43**)

White powder. Yield: 90%. M.p.: 93.0 °C. IR ν_max_ (cm^−1^): 3176.76, 3076.46, 2954.95, 2914.44, 2864.29, 2829.57, 1683.86, 1606.70, 1571.99, 1556.55, 1483.26, 1458.18, 1435.04, 1392.61, 1375.25, 1350.17, 1325.10, 1311.59, 1278.81, 1249.87, 1224.80, 1159.22, 1114.86, 1058.92, 1031.92, 995.27, 941.26, 891.11, 858.32, 825.53, 783.10, 754.17, 704.02, 659.66. ^1^H NMR (300 MHz, DMSO-d_6_): 1.34 (t, *J* = 6.90, 6.99 Hz, 3H), 2.37–2.47 (m, 12H), 3.55 (t, *J* = 4.59, 4.62 Hz, 4H), 3.93 (brs, 2H), 4.06 (q, *J* = 6.93, 6.96, 6.99 Hz, 2H), 4.18 (brs, 2H), 4.40 (s, 2H), 7.01 (dd, *J* = 2.58, 8.82 Hz, 1H), 7.55 (d, *J* = 2.55 Hz, 1H), 7.63 (d, *J* = 8.85 Hz, 1H), 12.52 (brs, 1H) (Figure S127). ^13^C NMR (75 MHz, DMSO-d_6_) δ (ppm): 15.17 (CH_3_), 40.28 (CH_2_), 50.34 (CH_2_), 51.69 (CH_2_), 52.94 (CH_2_), 54.10 (2CH_2_), 54.67 (CH_2_), 56.22 (2CH_2_), 64.02 (CH_2_), 66.64 (2CH_2_), 105.77 (CH), 115.75 (CH), 121.64 (CH), 133.16 (C), 142.98 (C), 155.82 (C), 156.26 (C), 167.07 (C), 194.32 (C) (Figure S128). HRMS (ESI) (*m*/*z*) [M + H]^+^ calcd. for C_22_H_31_N_5_O_3_S_3_: 510.1662, found: 510.1677 (Figure S129).

##### 2-Oxo-2-[(6-nitrobenzothiazol-2-yl)amino]ethyl 4-(2-morpholinoethyl)piperazine-1-carbodithioate (**44**)

Beige powder. Yield: 81%. M.p.: 194.0 °C. IR ν_max_ (cm^−1^): 3209.55, 3076.46, 3010.88, 2949.16, 2900.94, 2816.07, 1695.43, 1573.91, 1525.69, 1510.26, 1463.97, 1444.68, 1435.04, 1375.25, 1334.74, 1301.95, 1292.31, 1276.88, 1247.94, 1230.58, 1207.44, 1166.93, 1126.43, 1112.93, 1064.71, 1043.49, 1012.63, 993.34, 931.62, 914.26, 902.69, 864.11, 840.96, 821.68, 744.52, 719.45, 682.80. ^1^H NMR (300 MHz, DMSO-d_6_): 2.40–2.50 (m, 12H), 3.56 (t, *J* = 4.41, 4.44 Hz, 4H), 3.94 (brs, 2H), 4.17 (brs, 2H), 4.45 (s, 2H), 7.90 (d, *J* = 8.94 Hz, 1H), 8.28 (dd, *J* = 2.40, 8.94 Hz, 1H), 9.05 (d, *J* = 2.31 Hz, 1H), 13.01 (brs, 1H) (Figure S130). ^13^C NMR (75 MHz, DMSO-d_6_) δ (ppm): 40.43 (CH_2_), 50.37 (CH_2_), 51.67 (CH_2_), 52.81 (CH_2_), 54.05 (2CH_2_), 54.57 (CH_2_), 56.13 (2CH_2_), 66.57 (2CH_2_), 119.55 (CH), 121.06 (CH), 122.26 (CH), 132.64 (C), 143.38 (C), 154.02 (C), 164.09 (C), 168.37 (C), 194.19 (C) (Figure S131). HRMS (ESI) (*m*/*z*) [M + H]^+^ calcd. for C_20_H_26_N_6_O_4_S_3_: 511.1250, found: 511.1252 (Figure S132).

### 4.2. hCA Isoform Inhibition Studies

#### 4.2.1. Hydratase Activity

The hydratase activity of hCA was evaluated using a modified version of the Wilbur–Anderson assay, following the procedure reported by Rickli et al. [46,47]. Briefly, the catalytic conversion of CO_2_ was followed by monitoring the decrease in pH associated with the formation of H^+^ during the hydration reaction. Bromothymol blue was employed as a pH-sensitive indicator to visualize and monitor the reaction progress. The enzymatic activity was quantified in Enzyme Units (EU) based on the time required to reach the predetermined pH endpoint in the presence and absence of the enzyme. The activity was calculated using the following equation:

EU = (t_0_ − t_*c*_)/t_*c*_

where t_0_ denotes the time required to reach the specified pH change in the absence of hCA, whereas t_*c*_ corresponds to the time measured under enzyme-catalyzed conditions.

#### 4.2.2. Inhibition Assay and IC_50_/*K*_i_ Determination

The inhibitory activities of compounds **1–44** against the hCA I and II isoforms were investigated using the hydratase activity assay. For IC_50_ determination, a series of inhibitor concentrations was tested under constant substrate and enzyme conditions. The activity measured in the absence of an inhibitor was taken as the control value (100%), and the remaining enzymatic activity at each inhibitor concentration was subsequently calculated. IC_50_ values were derived from the corresponding inhibition profiles obtained by plotting the percentage of residual enzyme activity against inhibitor concentration. Each measurement was conducted in triplicate to ensure the reliability of the results.

The inhibition constant (*K*_i_) was subsequently estimated using the Cheng–Prusoff relationship in conjunction with a nonlinear least-squares fitting procedure [48,49]. AAZ was used as a reference inhibitor, and the inhibitory effects of compounds **1–44** were assessed relative to this standard [28]. All experiments were independently repeated three times, and the obtained results were used to confirm the reproducibility of the inhibition measurements.

#### 4.2.3. hCA IX and XII Activity and Inhibition Assay

The enzymatic activities of hCA IX (Novus Biologicals, Centennial, CO, USA) and XII (R&D Systems, Minneapolis, MN, USA) were determined using a colorimetric CA activity assay based on the esterase activity of the enzymes. The assay was performed according to the manufacturer’s instructions with minor modifications. The assay was performed in 96-well plates, and the formation of the chromophoric product was monitored at 405 nm using a microplate reader. Briefly, purified hCA IX and XII were diluted in CA assay buffer, and the enzyme solutions were preincubated at room temperature for 15 min. The reaction was initiated by adding 5 μL of CA substrate, and absorbance was monitored kinetically for 60 min. Enzyme activity was determined from the initial linear portion of the reaction curves using a nitrophenol standard curve.

For the inhibition studies, hCA IX and XII were preincubated with the synthesized compounds at different concentrations under the same assay conditions, followed by addition of the substrate. AAZ was used as a reference inhibitor. The inhibitory activity was determined by comparing the residual enzyme activity in the presence of each compound with that of the uninhibited enzyme control. All measurements were performed in triplicate, and the results were expressed as mean ± standard deviation (SD).

### 4.3. Anticancer Activity

#### 4.3.1. Preparation of Primary Cortical Neuron Culture

The experiments were conducted in accordance with the Guide for the Care and Use of Laboratory Animals. The study was approved by the Atatürk University Local Ethics Council of Animal Experiments (HADYEK; approval code: 41 and date of approval: 10 March 2022). Accordingly, newborn Sprague–Dawley rat pups less than 24 h old were used for the isolation of primary neurons from the cerebral cortex, and cerebellum tissues. The rat pups were rapidly decapitated, and the relevant tissues were carefully dissected. The isolated tissues were transferred into 5 mL of Hanks’ balanced salt solution (HBSS). Subsequently, the tissues were mechanically minced using a scalpel, followed by enzymatic dissociation with trypsin–ethylenediaminetetraacetic acid (EDTA) solution (0.25% trypsin–0.02% EDTA). After dissociation, the cell suspension was centrifuged at 1200 rpm for 5 min. The resulting cell pellet was resuspended in culture medium consisting of 88% neurobasal medium (NBM), 10% fetal bovine serum (FBS), 2% B-27 Supplement, and 0.1% penicillin–streptomycin and amphotericin B. The cells were cultured in plates and incubated at 37 °C in a humidified atmosphere containing 5% CO_2_ for 14 days. The culture medium was replaced every 5 days throughout the incubation period [50].

#### 4.3.2. Cell Culture and Treatment of Compounds

The U87 (HBT-14™) and T98 (CRL-1690™) human glioblastoma cell lines were obtained from the American Type Culture Collection (ATCC), expanded under appropriate culture conditions, and cryopreserved until use. The cells were cultured in DMEM/F12 medium for U87 cells and DMEM high-glucose medium for T98 cells, both supplemented with 10% fetal bovine serum (FBS), 100 IU/mL penicillin, and 10 mg/mL streptomycin. The cells were incubated at 37 °C in a humidified atmosphere containing 5% CO_2_ to allow cell proliferation [51,52].

When the cells reached sufficient confluency in the culture flasks, they were seeded into 96-well culture plates. Subsequently, cells that reached 80% confluency in the culture plates were treated with the test compounds alone at concentrations of 0.1, 1, 10, 100, and 1000 µM. The test compounds were initially dissolved in dimethyl sulfoxide (DMSO). To minimize any solvent-related cytotoxicity, the final concentration of DMSO in the culture medium was strictly maintained below 0.1%. The same DMSO limit (0.1%) was applied to the negative control groups to exclude potential solvent-related experimental effects.

#### 4.3.3. MTT Assay

The in vitro cytotoxic effects of the compounds exhibiting inhibitory activity against hCA IX and/or XII were determined using the MTT assay at 24, 48, and 72 h. This assay is based on a colorimetric quantification method in which 3-(4,5-dimethylthiazol-2-yl)-2,5-diphenyltetrazolium bromide (MTT), a water-soluble tetrazolium salt, is utilized during the measurements. Viable cells reduce MTT into insoluble purple formazan crystals through mitochondrial enzymatic activity.

Upon completion of the predetermined incubation periods, 10 µL of MTT solution was added to each well, followed by incubation for 4 h. Subsequently, 100 µL of DMSO was added to each well to dissolve the formed formazan crystals. The absorbance was then measured spectrophotometrically at a wavelength of 570 nm [50].

### 4.4. Biochemical Analyses

#### 4.4.1. TAS and TOS Analyses

To elucidate the effects of the compounds on cancer-associated oxidative stress, the TAS and TOS assay kits from Rel Assay Diagnostics^®^ (Gaziantep, Türkiye). The principle of the TAS assay is based on the reduction of the blue-green colored ABTS radical to its colorless form by antioxidants present in the tested sample. The principle of the TOS assay relies on the oxidation of the ferrous ion–chelator complex to ferric ions by oxidants present in the sample. The generated ferric ions react with the chromogen under acidic conditions, resulting in an increase in absorbance [53,54].

#### 4.4.2. Enzyme-Linked Immunosorbent Assays (ELISA)

In the ELISA studies, the plasma samples obtained from the cell culture media in which the cells were maintained were utilized [49]. To elucidate the effects of the compounds on cancer-associated inflammation, and cell death mechanisms, the TNF-α, IL-1β, IL-10, and TGF-β ELISA kits from Elabscience^®^ (Houston, TX, USA) were used according to the manufacturers’ protocols.

The Elabscience^®^ ELISA kits are based on the sandwich ELISA principle. According to the kit protocol, the micro-ELISA plate is pre-coated with an antibody specific to the target factor. The optical density (OD) was measured spectrophotometrically at 450 nm following the formation of the enzyme–substrate complex [55].

### 4.5. Statistical Analyses

Data obtained from the MTT and biochemical analyses were statistically analyzed using GraphPad Prism version 8.3.0 statistical software. Quantitative data are expressed as the mean ± SD. Statistical significance among multiple groups was assessed using one-way analysis of variance (ANOVA), followed by Tukey’s post-hoc test for multiple comparisons. For all analyses, statistical significance was set at * *p* < 0.05 and ** *p* < 0.001.

### 4.6. Molecular Docking Studies

Molecular docking studies were performed for the compounds exhibiting hCA IX and/or hCA XII inhibitory activity to investigate their potential binding modes within the active sites of hCA IX and XII. The X-ray crystal structures of hCA IX and hCA XII were obtained from the Protein Data Bank (PDB) using PDB IDs 3IAI and 1JD0, respectively. Prior to docking, the protein structures were prepared using the Protein Preparation Wizard implemented in Schrödinger Release 2023-2 (Schrödinger, LLC, New York, NY, USA). The protein structures were processed by assigning appropriate bond orders and protonation states, optimizing hydrogen-bonding networks, and performing restrained energy minimization using the OPLS force field. The compounds were prepared using the LigPrep module of Schrödinger Release 2023-2. The ligand structures were subjected to energy minimization using the OPLS force field, and their appropriate ionization states at physiological pH were considered during ligand preparation. For the docking calculations, receptor grids were generated at the active sites of hCA IX and hCA XII based on the binding sites observed in the corresponding X-ray crystal structures. The prepared ligands were subsequently docked into the active sites using the Glide docking protocol. The predicted binding modes were analyzed with respect to the interactions established between the docked compounds and the amino acid residues forming the catalytic active sites of hCA IX and hCA XII. The docking scores obtained for the compounds were recorded and used for comparative evaluation of their predicted binding modes.

### 4.7. In Silico ADME Studies

The in silico pharmacokinetic properties of compounds **4**, **9**, and **30** were evaluated using QikProp (Schrödinger Release 2023-2, LLC, New York, NY, USA). The SASA, QPlogPo/w, QPlogS, QPlogKhsa, and QPlogBB were calculated. Additionally, compliance with Lipinski’s Ro5 and Jorgensen’s Ro3 was assessed.

## 5. Conclusions

GBM remains the most aggressive primary brain tumor, with limited therapeutic options and poor patient survival. In the pursuit of hCA IX/XII-targeted therapeutic agents for GBM, a series of 44 novel DTC-based compounds were synthesized and evaluated for their inhibitory activity against hCA I, II, IX, and XII isoforms. The most potent hCA IX/XII inhibitors were further investigated for their anti-glioblastoma activity, selectivity toward healthy neuronal cells, and their effects on oxidative stress, inflammation, and cell death-related pathways.

In conclusion, compounds **4**, **9** and **30** exhibited promising cytotoxic activity in GBM cells by modulating oxidative stress, inflammatory mediators, and tumor-associated hCA isoforms. These compounds acted as dual hCA IX/XII inhibitors, exhibiting more potent inhibitory activity against hCA XII, suggesting that hCA XII may represent their primary molecular target.

In T98 cells, compound **9** increased TAS levels and significantly reduced IL-1β, IL-10, TNF-α, and TGF-β levels, whereas compound **30** improved the cellular redox balance by decreasing TOS and increasing TAS while also suppressing IL-1β, IL-10, TNF-α, and TGF-β. In U87 cells, both compounds enhanced antioxidant capacity by increasing TAS levels and significantly reduced IL-1β and IL-10 production in a time- and concentration-dependent manner. Moreover, compounds **9** and **30** exhibited a time-dependent modulation of TNF-α, characterized by an initial increase after 24 h followed by a marked reduction at later time points, indicating a progressive shift toward an anti-inflammatory response. Compound **30** also demonstrated a more sustained anti-inflammatory profile during prolonged exposure and predominantly suppressed TGF-β production, whereas compound **9** displayed a biphasic modulation of TGF-β, with an initial reduction followed by increased levels at intermediate exposure and higher concentrations. Collectively, these findings suggest that the antiglioblastoma effects of compounds **9** and **30** may be mediated, at least in part, through preferential inhibition of hCA XII, together with the modulation of oxidative stress and inflammatory signaling pathways, supporting their further development as promising lead candidates for GBM therapy. Similarly, compound **4**, a dual hCA IX/XII inhibitor with predominant hCA XII inhibitory activity, exerted antiglioblastoma effects in U87 cells by reducing oxidative stress, suppressing pro-inflammatory signaling, decreasing IL-10 production, and progressively inhibiting TGF-β signaling following prolonged exposure, thereby disrupting the oxidative stress–inflammation axis that supports glioblastoma cell survival and progression.

Molecular docking studies further supported these findings by confirming favorable binding interactions within the hCA IX/XII active sites, providing a structural basis for their inhibitory activities. Overall, the in silico ADME findings suggest that compounds **4**, **9**, and **30** exhibit favorable drug-like properties and BBB permeability, highlighting their potential as orally bioavailable candidates capable of reaching the target tumor and warranting further in vitro and in vivo evaluation.

Collectively, these findings indicate that the antiglioblastoma activities of compounds **4**, **9**, and **30** are mediated, at least in part, through preferential inhibition of hCA XII together with the modulation of oxidative stress and inflammatory signaling pathways, supporting their further development as promising hCA XII-targeted lead candidates for GBM therapy. These findings provide a strong rationale for future studies aimed at further elucidating the molecular mechanisms underlying their anti-GBM activity and evaluating their therapeutic potential in relevant preclinical models.

## Figures and Tables

**Figure 2:**
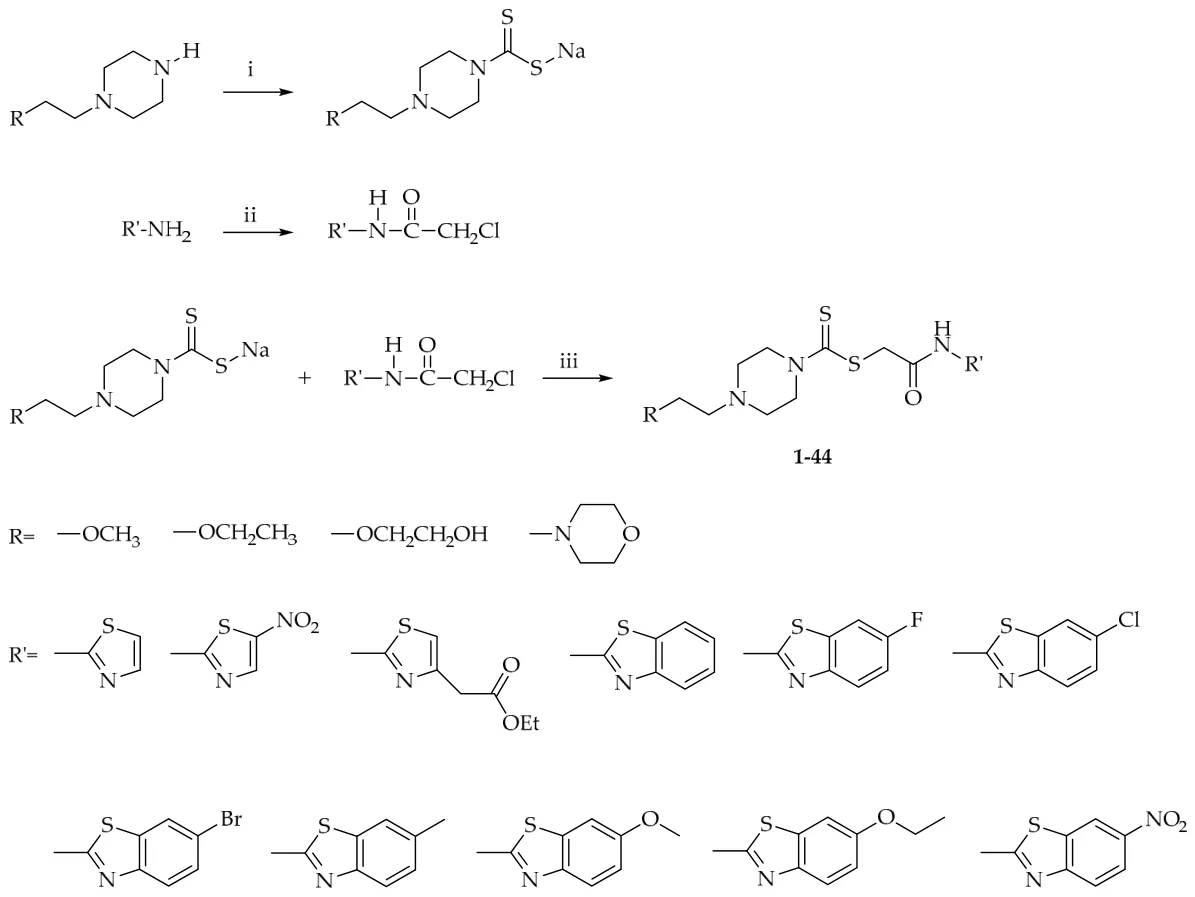
The synthetic route for the preparation of new DTCs (**1–44**). Reagents and conditions: (i) CS_2_, NaOH, EtOH, ice bath, 2 h; (ii) ClCOCH_2_Cl, TEA, toluene, ice bath; (iii) Acetone, room temperature, 8 h.

**Table 1:** Inhibitory effects of compounds **1–44** on hCA I and II.

Compound	hCA I				hCA II			
	IC_50_ (µM)	r^2^	*K*_i_ (µM)	Inhibition Type	IC_50_ (µM)	r^2^	*K*_i_ (µM)	Inhibition Type
**1**	7.91 ± 0.24	0.9919	4.21 ± 1.36	Competitive	9.64 ± 2.89	0.9651	7.21 ± 2.33	Competitive
**2**	25.73 ± 0.77	0.9976	20.66 ± 5.21	Competitive	11.22 ± 1.33	0.9883	9.08 ± 2.55	Competitive
**3**	20.48 ± 1.61	0.9946	13.33 ± 2.45	Noncompetitive	5.23 ± 0.15	0.9978	3.11 ± 0.33	Competitive
**4**	7.82 ± 0.23	0.9865	5.22 ± 1.03	Competitive	5.76 ± 0.17	0.9950	3.55 ± 1.32	Competitive
**5**	13.45 ± 0.45	0.9985	10.21 ± 3.63	Competitive	15.01 ± 4.52	0.9922	10.77 ± 3.18	Competitive
**6**	6.04 ± 0.28	0.9909	4.08 ± 1.78	Competitive	18.19 ± 2.54	0.9756	16.36 ± 3.45	Noncompetitive
**7**	4.83 ± 0.15	0.9966	2.77 ± 0.22	Competitive	7.03 ± 0.21	0.9917	5.44 ± 1.63	Competitive
**8**	3.96 ± 0.12	0.9929	2.14 ± 1.14	Noncompetitive	6.55 ± 0.19	0.9937	4.21 ± 1.09	Competitive
**9**	14.68 ± 0.55	0.9949	11.55 ± 3.12	Competitive	6.33 ± 0.18	0.9949	4.36 ± 1.65	Competitive
**10**	9.64 ± 1.29	0.9831	6.41 ± 2.41	Competitive	5.18 ± 1.55	0.9909	3.17 ± 1.23	Competitive
**11**	11.21 ± 0.33	0.9970	9.23 ± 1.13	Competitive	6.37 ± 0.19	0.9954	4.18 ± 0.44	Competitive
**12**	3.26 ± 0.10	0.9960	1.63 ± 0.03	Competitive	5.57 ± 0.17	0.9983	3.22 ± 1.06	Competitive
**13**	6.71 ± 0.20	0.9944	7.89 ± 2.65	Competitive	4.92 ± 0.76	0.9891	4.52 ± 2.45	Competitive
**14**	3.67 ± 0.12	0.9804	1.89 ± 0.36	Competitive	7.27 ± 1.28	0.9963	2.36 ± 1.36	Competitive
**15**	5.99 ± 0.18	0.9943	4.78 ± 2.12	Competitive	7.23 ± 2.16	0.9843	4.08 ± 2.55	Competitive
**16**	5.57 ± 0.18	0.9977	3.63 ± 1.56	Competitive	6.81 ± 0.43	0.9924	4.55 ± 1.47	Competitive
**17**	5.42 ± 0.17	0.9816	4.22 ± 2.11	Noncompetitive	7.56 ± 0.68	0.9842	5.11 ± 2.12	Noncompetitive
**18**	6.89 ± 0.22	0.9941	4.56 ± 2.09	Competitive	6.73 ± 0.20	0.9963	4.35 ± 1.33	Competitive
**19**	5.23 ± 0.15	0.9915	3.78 ± 1.32	Competitive	8.05 ± 0.24	0.9952	6.77 ± 2.66	Noncompetitive
**20**	6.68 ± 0.20	0.9906	4.08 ± 2.31	Competitive	6.49 ± 1.47	0.9985	5.22 ± 2.16	Competitive
**21**	6.97 ± 0.25	0.9922	3.89 ± 1.87	Competitive	5.87 ± 1.76	0.9928	3.11 ± 0.56	Competitive
**22**	6.20 ± 0.17	0.9959	5.11 ± 2.14	Competitive	3.52 ± 0.06	0.9929	1.75 ± 0.32	Competitive
**23**	6.02 ± 0.15	0.9526	4.25 ± 1.21	Competitive	7.86 ± 0.23	0.9942	5.23 ± 0.32	Competitive
**24**	15.92 ± 0.65	0.9974	7.86 ± 2.16	Competitive	7.52 ± 0.56	0.9901	5.12 ± 1.23	Competitive
**25**	20.97 ± 5.62	0.9984	15.87 ± 3.14	Competitive	7.41 ± 0.32	0.9953	5.03 ± 2.14	Competitive
**26**	17.81 ± 2.43	0.9914	10.01 ± 2.16	Competitive	8.12 ± 0.63	0.9907	6.12 ± 1.41	Competitive
**27**	9.29 ± 0.27	0.9897	7.21 ± 2.10	Competitive	7.16 ± 0.48	0.9933	4.05 ± 2.06	Competitive
**28**	14.02 ± 1.42	0.9927	12.32 ± 1.14	Noncompetitive	7.15 ± 0.21	0.9951	5.36 ± 1.01	Noncompetitive
**29**	10.59 ± 2.31	0.9714	6.56 ± 0.23	Competitive	7.65 ± 0.23	0.9914	4.63 ± 1.56	Competitive
**30**	27.38 ± 3.82	0.9825	20.41 ± 3.14	Competitive	17.81 ± 2.53	0.9903	12.19 ± 3.78	Competitive
**31**	8.99 ± 0.27	0.9954	12.02 ± 3.21	Competitive	7.74 ± 2.22	0.9917	10.32 ± 2.74	Competitive
**32**	29.64 ± 2.89	0.9840	12.56 ± 2.14	Noncompetitive	5.74 ± 0.17	0.9968	3.21 ± 0.12	Noncompetitive
**33**	27.34 ± 2.82	0.9870	20.13 ± 3.41	Competitive	5.59 ± 0.77	0.992	2.32 ± 0.36	Competitive
**34**	7.15 ± 1.22	0.9973	5.32 ± 2.02	Noncompetitive	3.85 ± 0.05	0.9900	2.31 ± 0.22	Noncompetitive
**35**	7.14 ± 1.21	0.9944	5.12 ± 1.32	Competitive	3.27 ± 0.81	0.9955	1.52 ± 0.63	Competitive
**36**	8.04 ± 1.24	0.9947	6.38 ± 0.8	Competitive	6.47 ± 0.41	0.9951	7.32 ± 2.56	Competitive
**37**	7.39 ± 1.22	0.9938	5.23 ± 1.06	Competitive	2.76 ± 0.08	0.9934	1.14 ± 0.33	Competitive
**38**	5.30 ± 1.59	0.9900	4.12 ± 1.56	Competitive	6.87 ± 2.06	0.9961	4.85 ± 1.85	Competitive
**39**	7.69 ± 0.23	0.9878	5.12 ± 2.06	Noncompetitive	5.71 ± 0.17	0.9911	3.66 ± 1.45	Noncompetitive
**40**	4.94 ± 0.15	0.9894	2.11 ± 0.11	Competitive	2.35 ± 0.07	0.9969	1.11 ± 0.05	Competitive
**41**	5.07 ± 0.15	0.9936	3.56 ± 2.00	Competitive	6.69 ± 0.20	0.9952	4.78 ± 1.03	Competitive
**42**	3.27 ± 0.09	0.9816	1.25 ± 0.23	Noncompetitive	4.83 ± 0.14	0.9892	3.87 ± 1.63	Noncompetitive
**43**	4.82 ± 1.46	0.9905	2.69 ± 1.06	Competitive	3.91 ± 0.11	0.9951	1.14 ± 0.56	Competitive
**44**	8.72 ± 1.26	0.9909	10.21 ± 2.85	Competitive	5.83 ± 0.17	0.9900	7.68 ± 2.47	Competitive
**AAZ**	0.16 ± 0.05	0.9965	0.05 ± 0.12	Competitive	0.12 ± 0.04	0.9856	0.011 ± 0.01	Competitive

**Table 2:** Inhibitory effects of compounds **1–44** on hCA IX and XII.

Compound	hCA IX				hCA XII			
	IC_50_ (nM)	r^2^	*K*_i_ (nM)	Inhibition Type	IC_50_ (nM)	r^2^	*K*_i_ (nM)	Inhibition Type
**1**	40.32 ± 6.32	0.9964	39.24 ± 5.14	Competitive	83.13 ± 15.41	0.9866	81.54 ± 0.63	Competitive
**2**	Enzyme activation was observed.				Enzyme activation was observed.			
**3**	55.03 ± 7.51	0.9906	53.95 ± 11.06	Competitive	51.02 ± 15.52	0.9933	35.99 ± 0.45	Competitive
**4**	57.49 ± 5.87	0.9925	55.91 ± 16.21	Competitive	21.41 ± 0.71	0.9847	15.12 ± 0.01	Competitive
**5**	49.75 ± 4.24	0.9833	48.06 ± 15.11	Competitive	55.04 ± 12.75	0.9911	38.85 ± 2.66	Competitive
**6**	Enzyme activation was observed.				84.93 ± 17.25	0.9922	59.95 ± 4.33	Competitive
**7**	Enzyme activation was observed.				59.61 ± 8.99	0.9814	42.08 ± 0.63	Competitive
**8**	61.21 ± 13.06	0.9745	59.84 ± 8.06	Competitive	51.78 ± 15.28	0.9714	35.99 ± 0.23	Competitive
**9**	40.72 ± 6.30	0.9903	39.24 ± 7.21	Competitive	20.95 ± 1.47	0.9643	14.11 ± 0.02	Competitive
**10**	59.90 ± 5.99	0.9863	57.87 ± 5.66	Competitive	56.22 ± 1.28	0.9887	39.53 ± 4.03	Competitive
**11**	Enzyme activation was observed.				Enzyme activation was observed.			
**12**	44.86 ± 12.43	0.9875	44.00 ± 5.36	Noncompetitive	49.78 ± 8.95	0.9922	34.59 ± 2.03	Noncompetitive
**13**	Enzyme activation was observed.				Enzyme activation was observed.			
**14**	34.06 ± 7.31	0.9924	33.41 ± 4.46	Competitive	50.60 ± 12.35	0.9916	35.29 ± 0.63	Competitive
**15**	39.70 ± 3.57	0.9873	38.94 ± 12.34	Competitive	16.29 ± 1.48	0.9844	11.29 ± 0.04	Competitive
**16**	47.90 ± 5.98	0.9705	46.10 ± 6.08	Competitive	29.84 ± 1.49	0.9912	20.47 ± 0.05	Competitive
**17**	40.23 ± 9.53	0.9947	39.24 ± 6.67	Competitive	12.01 ± 0.60	0.9856	8.47 ± 0.01	Competitive
**18**	51.44 ± 11.52	0.9688	50.03 ± 9.22	Competitive	20.85 ± 1.42	0.9919	14.12 ± 0.03	Competitive
**19**	44.19 ± 7.25	0.9923	43.16 ± 10.71	Competitive	38.62 ± 1.93	0.9921	26.82 ± 0.10	Competitive
**20**	14.72 ± 9.22	0.9767	13.73 ± 0.22	Competitive	17.61 ± 0.88	0.9833	11.99 ± 0.04	Competitive
**21**	14.70 ± 3.76	0.9922	15.72 ± 5.61	Competitive	Enzyme activation was observed.			
**22**	Enzyme activation was observed.				Enzyme activation was observed.			
**23**	36.53 ± 8.65	0.9933	38.63 ± 0.03	Noncompetitive	50.59 ± 2.59	0.9917	35.29 ± 0.07	Noncompetitive
**24**	Enzyme activation was observed.				Enzyme activation was observed.			
**25**	41.45 ± 7.52	0.9898	40.22 ± 0.27	Competitive	50.80 ± 12.45	0.9965	35.29 ± 0.65	Competitive
**26**	39.91 ± 7.25	0.9983	38.26 ± 2.17	Competitive	86.42 ± 13.21	0.9806	60.71 ± 3.06	Competitive
**27**	42.01 ± 19.95	0.9922	40.19 ± 3.44	Competitive	41.23 ± 6.21	0.9873	28.94 ± 2.33	Competitive
**28**	62.98 ± 21.05	0.9875	60.82 ± 11.61	Competitive	58.23 ± 1.92	0.9922	40.94 ± 4.01	Competitive
**29**	48.95 ± 13.49	0.9948	47.08 ± 4.78	Competitive	30.04 ± 1.55	0.9918	21.18 ± 0.08	Competitive
**30**	45.67 ± 7.45	0.9866	44.14 ± 0.66	Competitive	29.83 ± 1.49	0.9854	20.47 ± 0.65	Competitive
**31**	42.60 ± 12.83	0.9862	40.37 ± 0.03	Competitive	55.48 ± 2.77	0.9706	54.99 ± 2.63	Competitive
**32**	49.35 ± 10.30	0.9732	46.00 ± 0.13	Competitive	Enzyme activation was observed.			
**33**	Enzyme activation was observed.				Enzyme activation was observed.			
**34**	51.51 ± 15.76	0.9935	50.03 ± 1.22	Noncompetitive	61.07 ± 13.35	0.9622	43.06 ± 1.83	Noncompetitive
**35**	Enzyme activation was observed.				Enzyme activation was observed.			
**36**	32.17 ± 1.63	0.9909	31.19 ± 0.14	Competitive	50.31 ± 15.58	0.9892	35.29 ± 3.88	Competitive
**37**	44.40 ± 1.68	0.9947	43.57 ± 0.21	Competitive	47.76 ± 8.32	0.9825	35.18 ± 5.08	Competitive
**38**	48.95 ± 12.20	0.9145	47.99 ± 1.03	Competitive	48.84 ± 14.22	0.9988	33.88 ± 5.11	Competitive
**39**	28.15 ± 2.44	0.9957	27.59 ± 2.06	Competitive	55.81 ± 10.97	0.9870	39.39 ± 1.13	Competitive
**40**	53.79 ± 7.14	0.9833	52.79 ± 3.16	Noncompetitive	55.51 ± 13.75	0.9933	50.51 ± 0.16	Noncompetitive
**41**	35.62 ± 5.68	0.9866	34.98 ± 0.26	Competitive	76.60 ± 13.80	0.9751	54.07 ± 4.14	Competitive
**42**	61.70 ± 8.17	0.9918	60.51 ± 0.09	Competitive	54.15 ± 12.77	0.9947	38.22 ± 0.77	Competitive
**43**	62.80 ± 13.85	0.9877	61.65 ± 0.13	Competitive	42.31 ± 5.12	0.9884	35.31 ± 3.61	Competitive
**44**	Enzyme activation was observed.				Enzyme activation was observed.			
**AAZ**	35.26 ± 3.14	0.9832	44.57 ± 3.17	Competitive	30.13 ± 1.65	0.9907	13.22 ± 0.33	Competitive

**Table 3:** Cytotoxic effects of hCA IX and/or hCA XII inhibitors on T98 and U87 human glioblastoma cells at 24, 48, and 72 h.

Compound	IC_50_ Values for the T98 Cell Line (µM)			IC_50_ Values for the U87 Cell Line (µM)		
	24 h	48 h	72 h	24 h	48 h	72 h
**4**	-	-	270.40	33.85	35.56	3.86
**9**	-	875.70	69.69	74.26	35.64	120.80
**14**	-	889.20	185.30	952.10	490.30	41.73
**15**	-	332.70	104.10	212.40	73.19	4.79
**17**	753.30	-	96.62	102.70	113.80	87.39
**20**	539.70	261.10	548.00	207.30	493.50	532.40
**21**	464.20	604.20	316.80	121.40	371.50	189.30
**23**	-	435.20	756.70	-	218.00	20.33
**29**	832.70	480.20	228.80	106.40	138.50	120.30
**30**	-	642.80	57.71	61.28	17.76	19.51
**36**	581.10	622.20	-	-	875.70	134.90
**41**	404.90	767.10	-	428.50	187.80	179.10
TMZ *	-	-	438.30	123.90	223.10	230.00

* IC_50_ values for TMZ obtained from a previous study [31] and used for comparison. -: IC_50_ could not be determined within the tested concentration range (up to 1000 µM).

**Table 4:** SI data of compounds **4**, **9** and **30** for T98 and U87 cells.

Compound	IC_50_ for Normal Cells (µM)	IC_50_ for T98 Cells (µM)	SI for T98 Cells	IC_50_ for U87 Cells (µM)	SI for U87 Cells
**4**	>1000	270.40	>3.70	3.86	>259.07
**9**	>1000	69.69	>14.35	120.80	>8.28
**30**	>1000	57.71	>17.33	19.51	>51.26

**Table 5:** Predicted ADME profiles of compounds **4**, **9** and **30**.

Compound	SASA *	QPlogPo/w *	QPlogS *	QPlogKhsa *	QPlogBB *	Ro5 **	Ro3 **
**4**	727.779	2.587	−3.969	−0.222	−0.169	0	0
**9**	764.921	2.660	−4.151	−0.233	−0.252	0	0
**30**	818.888	2.314	−4.551	−0.258	−1.100	0	0

* Recommended values for SASA: 300–1000 Å2, QPlogPo/w: −2–6.5, QPlogS: −6.5–0.5, QPlogKhsa: −1.5–1.5, QPlogBB: –3.0–1.2. ** Ro5: Number of violations of Lipinski’s rule of five. The rules are: molecular weight of the molecule < 500, QPlogPo/w < 5, hydrogen-bond donor atoms ≤ 5, hydrogen-bond acceptor atoms ≤ 10. Compounds that provide these rules are considered as drug-like molecules. Ro3: Number of violations of Jorgensen’s rule of three. The three rules are: QPlogS > −5.7, Predicted apparent Caco-2 cell permeability (QPPCaco) > 22 nm/s, # Primary Metabolites < 7. Compounds with fewer (and preferably no) violations of these rules are more likely to be orally available agents (Schrödinger Release 2023-2, LLC, New York, NY, USA).

**Table 6:** Structures of the novel DTC-based compounds **1–44**.

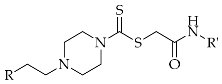					
Cmpd.	R	R’	Cmpd.	R	R’
**1**	OCH_3_	Thiazol-2-yl	**23**	OCH_2_CH_2_OH	Thiazol-2-yl
**2**	OCH_3_	5-Nitrothiazol-2-yl	**24**	OCH_2_CH_2_OH	5-Nitrothiazol-2-yl
**3**	OCH_3_	4-(Ethoxycarbonylmethyl)thiazol-2-yl	**25**	OCH_2_CH_2_OH	4-(Ethoxycarbonylmethyl)thiazol-2-yl
**4**	OCH_3_	Benzothiazol-2-yl	**26**	OCH_2_CH_2_OH	Benzothiazol-2-yl
**5**	OCH_3_	6-Fluorobenzothiazol-2-yl	**27**	OCH_2_CH_2_OH	6-Fluorobenzothiazol-2-yl
**6**	OCH_3_	6-Chlorobenzothiazol-2-yl	**28**	OCH_2_CH_2_OH	6-Chlorobenzothiazol-2-yl
**7**	OCH_3_	6-Bromobenzothiazol-2-yl	**29**	OCH_2_CH_2_OH	6-Bromobenzothiazol-2-yl
**8**	OCH_3_	6-Methylbenzothiazol-2-yl	**30**	OCH_2_CH_2_OH	6-Methylbenzothiazol-2-yl
**9**	OCH_3_	6-Methoxybenzothiazol-2-yl	**31**	OCH_2_CH_2_OH	6-Methoxybenzothiazol-2-yl
**10**	OCH_3_	6-Ethoxybenzothiazol-2-yl	**32**	OCH_2_CH_2_OH	6-Ethoxybenzothiazol-2-yl
**11**	OCH_3_	6-Nitrobenzothiazol-2-yl	**33**	OCH_2_CH_2_OH	6-Nitrobenzothiazol-2-yl
**12**	OC_2_H_5_	Thiazol-2-yl	**34**	Morpholin-4-yl	Thiazol-2-yl
**13**	OC_2_H_5_	5-Nitrothiazol-2-yl	**35**	Morpholin-4-yl	5-Nitrothiazol-2-yl
**14**	OC_2_H_5_	4-(Ethoxycarbonylmethyl)thiazol-2-yl	**36**	Morpholin-4-yl	4-(Ethoxycarbonylmethyl)thiazol-2-yl
**15**	OC_2_H_5_	Benzothiazol-2-yl	**37**	Morpholin-4-yl	Benzothiazol-2-yl
**16**	OC_2_H_5_	6-Fluorobenzothiazol-2-yl	**38**	Morpholin-4-yl	6-Fluorobenzothiazol-2-yl
**17**	OC_2_H_5_	6-Chlorobenzothiazol-2-yl	**39**	Morpholin-4-yl	6-Chlorobenzothiazol-2-yl
**18**	OC_2_H_5_	6-Bromobenzothiazol-2-yl	**40**	Morpholin-4-yl	6-Bromobenzothiazol-2-yl
**19**	OC_2_H_5_	6-Methylbenzothiazol-2-yl	**41**	Morpholin-4-yl	6-Methylbenzothiazol-2-yl
**20**	OC_2_H_5_	6-Methoxybenzothiazol-2-yl	**42**	Morpholin-4-yl	6-Methoxybenzothiazol-2-yl
**21**	OC_2_H_5_	6-Ethoxybenzothiazol-2-yl	**43**	Morpholin-4-yl	6-Ethoxybenzothiazol-2-yl
**22**	OC_2_H_5_	6-Nitrobenzothiazol-2-yl	**44**	Morpholin-4-yl	6-Nitrobenzothiazol-2-yl

## Data Availability

The data presented in this study are available from the corresponding author upon reasonable request.

## Supplementary Materials

The following supporting information can be downloaded at https://www.mdpi.com/article/10.3390/molecules31203593/s1, Figure S1. ^1^H NMR spectrum of compound **1**; Figure S2. ^13^C NMR spectrum of compound **1**; Figure S3. HRMS spectrum of compound **1**; Figure S4. ^1^H NMR spectrum of compound **2**; Figure S5. ^13^C NMR spectrum of compound **2**; Figure S6. HRMS spectrum of compound **2**; Figure S7. ^1^H NMR spectrum of compound **3**; Figure S8. ^13^C NMR spectrum of compound **3**; Figure S9. HRMS spectrum of compound **3**; Figure S10. ^1^H NMR spectrum of compound **4**; Figure S11. ^13^C NMR spectrum of compound **4**; Figure S12. HRMS spectrum of compound **4**; Figure S13. ^1^H NMR spectrum of compound **5**; Figure S14. ^13^C NMR spectrum of compound **5**; Figure S15. HRMS spectrum of compound **5**; Figure S16. ^1^H NMR spectrum of compound **6**; Figure S17. ^13^C NMR spectrum of compound **6**; Figure S18. HRMS spectrum of compound **6**; Figure S19. ^1^H NMR spectrum of compound **7**; Figure S20. ^13^C NMR spectrum of compound **7**; Figure S21. HRMS spectrum of compound **7**; Figure S22. ^1^H NMR spectrum of compound **8**; Figure S23. ^13^C NMR spectrum of compound **8**; Figure S24. HRMS spectrum of compound **8**; Figure S25. ^1^H NMR spectrum of compound **9**; Figure S26. ^13^C NMR spectrum of compound **9**; Figure S27. HRMS spectrum of compound **9**; Figure S28. ^1^H NMR spectrum of compound **10**; Figure S29. ^13^C NMR spectrum of compound **10**; Figure S30. HRMS spectrum of compound **10**; Figure S31. ^1^H NMR spectrum of compound **11**; Figure S32. ^13^C NMR spectrum of compound **11**; Figure S33. HRMS spectrum of compound **11**; Figure S34. ^1^H NMR spectrum of compound **12**; Figure S35. ^13^C NMR spectrum of compound **12**; Figure S36. HRMS spectrum of compound **12**; Figure S37. ^1^H NMR spectrum of compound **13**; Figure S38. ^13^C NMR spectrum of compound **13**; Figure S39. HRMS spectrum of compound **13**; Figure S40. ^1^H NMR spectrum of compound **14**; Figure S41. ^13^C NMR spectrum of compound **14**; Figure S42. HRMS spectrum of compound **14**; Figure S43. ^1^H NMR spectrum of compound **15**; Figure S44. ^13^C NMR spectrum of compound **15**; Figure S45. HRMS spectrum of compound **15**; Figure S46. ^1^H NMR spectrum of compound **16**; Figure S47. ^13^C NMR spectrum of compound **16**; Figure S48. HRMS spectrum of compound **16**; Figure S49. ^1^H NMR spectrum of compound **17**; Figure S50. ^13^C NMR spectrum of compound **17**; Figure S51. HRMS spectrum of compound **17**; Figure S52. ^1^H NMR spectrum of compound **18**; Figure S53. ^13^C NMR spectrum of compound **18**; Figure S54. HRMS spectrum of compound **18**; Figure S55. ^1^H NMR spectrum of compound **19**; Figure S56. ^13^C NMR spectrum of compound **19**; Figure S57. HRMS spectrum of compound **19**; Figure S58. ^1^H NMR spectrum of compound **20**; Figure S59. ^13^C NMR spectrum of compound **20**; Figure S60. HRMS spectrum of compound **20**; Figure S61. ^1^H NMR spectrum of compound **21**; Figure S62. ^13^C NMR spectrum of compound **21**; Figure S63. HRMS spectrum of compound **21**; Figure S64. ^1^H NMR spectrum of compound **22**; Figure S65. ^13^C NMR spectrum of compound **22**; Figure S66. HRMS spectrum of compound **22**; Figure S67. ^1^H NMR spectrum of compound **23**; Figure S68. ^13^C NMR spectrum of compound **23**; Figure S69. HRMS spectrum of compound **23**; Figure S70. ^1^H NMR spectrum of compound **24**; Figure S71. ^13^C NMR spectrum of compound **24**; Figure S72. HRMS spectrum of compound **24**; Figure S73. ^1^H NMR spectrum of compound **25**; Figure S74. ^13^C NMR spectrum of compound **25**; Figure S75. HRMS spectrum of compound **25**; Figure S76. ^1^H NMR spectrum of compound **26**; Figure S77. ^13^C NMR spectrum of compound **26**; Figure S78. HRMS spectrum of compound **26**; Figure S79. ^1^H NMR spectrum of compound **27**; Figure S80. ^13^C NMR spectrum of compound **27**; Figure S81. HRMS spectrum of compound **27**; Figure S82. ^1^H NMR spectrum of compound **28**; Figure S83. ^13^C NMR spectrum of compound **28**; Figure S84. HRMS spectrum of compound **28**; Figure S85. ^1^H NMR spectrum of compound **29**; Figure S86. ^13^C NMR spectrum of compound **29**; Figure S87. HRMS spectrum of compound **29**; Figure S88. ^1^H NMR spectrum of compound **30**; Figure S89. ^13^C NMR spectrum of compound **30**; Figure S90. HRMS spectrum of compound **30**; Figure S91. ^1^H NMR spectrum of compound **31**; Figure S92. ^13^C NMR spectrum of compound **31**; Figure S93. HRMS spectrum of compound **31**; Figure S94. ^1^H NMR spectrum of compound **32**; Figure S95. ^13^C NMR spectrum of compound **32**; Figure S96. HRMS spectrum of compound **32**; Figure S97. ^1^H NMR spectrum of compound **33**; Figure S98. ^13^C NMR spectrum of compound **33**; Figure S99. HRMS spectrum of compound **33**; Figure S100. ^1^H NMR spectrum of compound **34**; Figure S101. ^13^C NMR spectrum of compound **34**; Figure S102. HRMS spectrum of compound **34**; Figure S103. ^1^H NMR spectrum of compound **35**; Figure S104. ^13^C NMR spectrum of compound **35**; Figure S105. HRMS spectrum of compound **35**; Figure S106. ^1^H NMR spectrum of compound **36**; Figure S107. ^13^C NMR spectrum of compound **36**; Figure S108. HRMS spectrum of compound **36**; Figure S109. ^1^H NMR spectrum of compound **37**; Figure S110. ^13^C NMR spectrum of compound **37**; Figure S111. HRMS spectrum of compound **37**; Figure S112. ^1^H NMR spectrum of compound **38**; Figure S113. ^13^C NMR spectrum of compound **38**; Figure S114. HRMS spectrum of compound **38**; Figure S115. ^1^H NMR spectrum of compound **39**; Figure S116. ^13^C NMR spectrum of compound **39**; Figure S117. HRMS spectrum of compound **39**; Figure S118. ^1^H NMR spectrum of compound **40**; Figure S119. ^13^C NMR spectrum of compound **40**; Figure S120. HRMS spectrum of compound **40**; Figure S121. ^1^H NMR spectrum of compound **41**; Figure S122. ^13^C NMR spectrum of compound **41**; Figure S123. HRMS spectrum of compound **41**; Figure S124. ^1^H NMR spectrum of compound **42**; Figure S125. ^13^C NMR spectrum of compound **42**; Figure S126. HRMS spectrum of compound **42**; Figure S127. ^1^H NMR spectrum of compound **43**; Figure S128. ^13^C NMR spectrum of compound **43**; Figure S129. HRMS spectrum of compound **43**; Figure S130. ^1^H NMR spectrum of compound **44**; Figure S131. ^13^C NMR spectrum of compound **44**; Figure S132. HRMS spectrum of compound **44**; Figure S133. Docking poses of compound **14** (**A**), compound **20** (**B**), compound **21** (**C**), compound **36** (**D**), compound **39** (**E**), and AAZ (**F**) within the catalytic domain of hCA IX (PDB ID: 3IAI); Figure S134. Docking poses of compound **4** (**A**), compound **9** (**B**), compound **15** (**C**), compound **16** (**D**), compound **17** (**E**), compound **18** (**F**), compound **20** (**G**), compound **29** (**H**), compound **30** (**I**), and AAZ (**J**) in the catalytic domain of hCA XII (PDB ID: 1JD0).

## Abbreviations

The following abbreviations are used in this manuscript:

AAZ	Acetazolamide
ADME	Absorption, Distribution, Metabolism, and Excretion
BBB	Blood–brain barrier
BBTB	Blood–brain tumor barrier
DTC	Dithiocarbamate
EDTA	Ethylenediaminetetraacetic acid
FBS	Fetal bovine serum
FT	Fourier Transform
HBSS	Hanks’ balanced salt solution
hCA	Human carbonic anhydrase
HRMS	High Resolution Mass Spectrometry
IL-10	Interleukin-10
IL-1β	Interleukin-1 beta
IR	Infrared
MGMT	O^6^-methylguanine-DNA methyltransferase
M.p.	Melting point
MTT	3-(4,5-Dimethylthiazol-2-yl)-2,5-diphenyltetrazolium bromide
NBM	Neurobasal medium
NMR	Nuclear Magnetic Resonance
PBS	Phosphate-buffered saline
QPlogBB	Predicted brain/blood partition coefficient
QPlogKhsa	Predicted human serum albumin binding
QPlogPo/w	Predicted octanol/water partition coefficient
QPlogS	Predicted aqueous solubility
Ro3	Rule of Three
Ro5	Rule of Five
SASA	Total solvent accessible surface area
SI	Selectivity index
TAS	Total antioxidant status
TEA	Triethylamine
TGF-β	Transforming growth factor-beta
TLC	Thin Layer Chromatography
TME	Tumor microenvironment
TMZ	Temozolomide
TNF-α	Tumor necrosis factor-alpha
TOS	Total oxidative status
