# Supplementary material for: Design, Synthesis, and Biological Evaluation of Novel Dithiocarbamate-Based Human Carbonic Anhydrase IX/XII Inhibitors as Potential Anti-Glioblastoma Agents
Source: Molecules. 2026 Oct 9;31(20):3593. doi: 10.3390/molecules31203593 (PMC13707839; doi:10.3390/molecules31203593)
Supplement: Supplementary file 1 [file molecules-31-03593-s001.zip › molecules-4547826-supplementary.pdf]

Supplementary Material

Design, Synthesis, and Biological Evaluation of Novel Dithiocarbamate-Based Human Carbonic Anhydrase IX/XII Inhibitors as Potential Anti-Glioblastoma Agents

Mehlika Dilek Altıntop [1,*], Hayrunnisa Nadaroglu [2,3], Fatma Yesilyurt [4,5], Nalan Yılmaz Savaş [6], Ruken Avsaroglu [7], Azize Alaylı [8], Kaan Küçükoğlu [9], Ahmet Hacimuftuoglu [7], and Ahmet Özdemir [1]

[1] Department of Pharmaceutical Chemistry, Faculty of Pharmacy, Anadolu University, 26470 Eskişehir, Türkiye; ahmeto@anadolu.edu.tr
[2] Department of Food Technology, Vocational College of Technical Science, Ataturk University, 25240 Erzurum, Türkiye; hnisa25@atauni.edu.tr
[3] Department of Nanoscience and Nanoengineering, Institute of Natural and Applied Sciences, Ataturk University, 25240 Erzurum, Türkiye
[4] Department of Pharmacology, Faculty of Dentistry, Atatürk University, 25240 Erzurum, Türkiye; fatmayesilyurt@atauni.edu.tr
[5] Vocational School of Health Services, Department of Pharmacy Services, Ataturk University, 25240 Erzurum, Türkiye
[6] Department of Pharmaceutical Chemistry, Graduate School, Anadolu University, 26470 Eskişehir, Türkiye; nalan_y@anadolu.edu.tr
[7] Department of Medical Pharmacology, Faculty of Medicine, Ataturk University, 25240 Erzurum, Türkiye; rukenavsaroglu@gmail.com; ahmeth@atauni.edu.tr
[8] Department of Nursing, Faculty of Health Sciences, Sakarya University of Applied Sciences, 54187 Sakarya, Türkiye; aalayli@subu.edu.tr
[9] Department of Pharmaceutical Chemistry, Faculty of Pharmacy, Selçuk University, 42130 Konya, Türkiye; kaan.kucukoglu@selcuk.edu.tr

* Correspondence: mdaltintop@anadolu.edu.tr; Tel.: +90-222-335-0580 (ext. 3807) (M.D.A.)

List of Contents

Figure	Page
**Figure S1.** ^{1}H NMR spectrum of compound **1**	S7
**Figure S2.** ^{13}C NMR spectrum of compound **1**	S8
**Figure S3.** HRMS spectrum of compound **1**	S9
**Figure S4.** ^{1}H NMR spectrum of compound **2**	S10
**Figure S5.** ^{13}C NMR spectrum of compound **2**	S11
**Figure S6.** HRMS spectrum of compound **2**	S12
**Figure S7.** ^{1}H NMR spectrum of compound **3**	S13
**Figure S8.** ^{13}C NMR spectrum of compound **3**	S14
**Figure S9.** HRMS spectrum of compound **3**	S15
**Figure S10.** ^{1}H NMR spectrum of compound **4**	S16
**Figure S11.** ^{13}C NMR spectrum of compound **4**	S17
**Figure S12.** HRMS spectrum of compound **4**	S18
**Figure S13.** ^{1}H NMR spectrum of compound **5**	S19
**Figure S14.** ^{13}C NMR spectrum of compound **5**	S20
**Figure S15.** HRMS spectrum of compound **5**	S21
**Figure S16.** ^{1}H NMR spectrum of compound **6**	S22
**Figure S17.** ^{13}C NMR spectrum of compound **6**	S23
**Figure S18.** HRMS spectrum of compound **6**	S24
**Figure S19.** ^{1}H NMR spectrum of compound **7**	S25
**Figure S20.** ^{13}C NMR spectrum of compound **7**	S26
**Figure S21.** HRMS spectrum of compound **7**	S27
**Figure S22.** ^{1}H NMR spectrum of compound **8**	S28
**Figure S23.** ^{13}C NMR spectrum of compound **8**	S29
**Figure S24.** HRMS spectrum of compound **8**	S30
**Figure S25.** ^{1}H NMR spectrum of compound **9**	S31
**Figure S26.** ^{13}C NMR spectrum of compound **9**	S32
**Figure S27.** HRMS spectrum of compound **9**	S33
**Figure S28.** ^{1}H NMR spectrum of compound **10**	S34
**Figure S29.** ^{13}C NMR spectrum of compound **10**	S35

Figure S30. HRMS spectrum of compound **10** S36

Figure S31. ^{1}H NMR spectrum of compound **11** S37

Figure S32. ^{13}C NMR spectrum of compound **11** S38

Figure S33. HRMS spectrum of compound **11** S39

Figure S34. ^{1}H NMR spectrum of compound **12** S40

Figure S35. ^{13}C NMR spectrum of compound **12** S41

Figure S36. HRMS spectrum of compound **12** S42

Figure S37. ^{1}H NMR spectrum of compound **13** S43

Figure S38. ^{13}C NMR spectrum of compound **13** S44

Figure S39. HRMS spectrum of compound **13** S45

Figure S40. ^{1}H NMR spectrum of compound **14** S46

Figure S41. ^{13}C NMR spectrum of compound **14** S47

Figure S42. HRMS spectrum of compound **14** S48

Figure S43. ^{1}H NMR spectrum of compound **15** S49

Figure S44. ^{13}C NMR spectrum of compound **15** S50

Figure S45. HRMS spectrum of compound **15** S51

Figure S46. ^{1}H NMR spectrum of compound **16** S52

Figure S47. ^{13}C NMR spectrum of compound **16** S53

Figure S48. HRMS spectrum of compound **16** S54

Figure S49. ^{1}H NMR spectrum of compound **17** S55

Figure S50. ^{13}C NMR spectrum of compound **17** S56

Figure S51. HRMS spectrum of compound **17** S57

Figure S52. ^{1}H NMR spectrum of compound **18** S58

Figure S53. ^{13}C NMR spectrum of compound **18** S59

Figure S54. HRMS spectrum of compound **18** S60

Figure S55. ^{1}H NMR spectrum of compound **19** S61

Figure S56. ^{13}C NMR spectrum of compound **19** S62

Figure S57. HRMS spectrum of compound **19** S63

Figure S58. ^{1}H NMR spectrum of compound **20** S64

Figure S59. ^{13}C NMR spectrum of compound **20** S65

Figure S60. HRMS spectrum of compound **20** S66

Figure S61. ^{1}H NMR spectrum of compound **21** S67
Figure S62. ^{13}C NMR spectrum of compound **21** S68
Figure S63. HRMS spectrum of compound **21** S69
Figure S64. ^{1}H NMR spectrum of compound **22** S70
Figure S65. ^{13}C NMR spectrum of compound **22** S71
Figure S66. HRMS spectrum of compound **22** S72
Figure S67. ^{1}H NMR spectrum of compound **23** S73
Figure S68. ^{13}C NMR spectrum of compound **23** S74
Figure S69. HRMS spectrum of compound **23** S75
Figure S70. ^{1}H NMR spectrum of compound **24** S76
Figure S71. ^{13}C NMR spectrum of compound **24** S77
Figure S72. HRMS spectrum of compound **24** S78
Figure S73. ^{1}H NMR spectrum of compound **25** S79
Figure S74. ^{13}C NMR spectrum of compound **25** S80
Figure S75. HRMS spectrum of compound **25** S81
Figure S76. ^{1}H NMR spectrum of compound **26** S82
Figure S77. ^{13}C NMR spectrum of compound **26** S83
Figure S78. HRMS spectrum of compound **26** S84
Figure S79. ^{1}H NMR spectrum of compound **27** S85
Figure S80. ^{13}C NMR spectrum of compound **27** S86
Figure S81. HRMS spectrum of compound **27** S87
Figure S82. ^{1}H NMR spectrum of compound **28** S88
Figure S83. ^{13}C NMR spectrum of compound **28** S89
Figure S84. HRMS spectrum of compound **28** S90
Figure S85. ^{1}H NMR spectrum of compound **29** S91
Figure S86. ^{13}C NMR spectrum of compound **29** S92
Figure S87. HRMS spectrum of compound **29** S93
Figure S88. ^{1}H NMR spectrum of compound **30** S94
Figure S89. ^{13}C NMR spectrum of compound **30** S95
Figure S90. HRMS spectrum of compound **30** S96
Figure S91. ^{1}H NMR spectrum of compound **31** S97

Figure S92. ^{13}C NMR spectrum of compound **31** S98

Figure S93. HRMS spectrum of compound **31** S99

Figure S94. ^{1}H NMR spectrum of compound **32** S100

Figure S95. ^{13}C NMR spectrum of compound **32** S101

Figure S96. HRMS spectrum of compound **32** S102

Figure S97. ^{1}H NMR spectrum of compound **33** S103

Figure S98. ^{13}C NMR spectrum of compound **33** S104

Figure S99. HRMS spectrum of compound **33** S105

Figure S100. ^{1}H NMR spectrum of compound **34** S106

Figure S101. ^{13}C NMR spectrum of compound **34** S107

Figure S102. HRMS spectrum of compound **34** S108

Figure S103. ^{1}H NMR spectrum of compound **35** S109

Figure S104. ^{13}C NMR spectrum of compound **35** S110

Figure S105. HRMS spectrum of compound **35** S111

Figure S106. ^{1}H NMR spectrum of compound **36** S112

Figure S107. ^{13}C NMR spectrum of compound **36** S113

Figure S108. HRMS spectrum of compound **36** S114

Figure S109. ^{1}H NMR spectrum of compound **37** S115

Figure S110. ^{13}C NMR spectrum of compound **37** S116

Figure S111. HRMS spectrum of compound **37** S117

Figure S112. ^{1}H NMR spectrum of compound **38** S118

Figure S113. ^{13}C NMR spectrum of compound **38** S119

Figure S114. HRMS spectrum of compound **38** S120

Figure S115. ^{1}H NMR spectrum of compound **39** S121

Figure S116. ^{13}C NMR spectrum of compound **39** S122

Figure S117. HRMS spectrum of compound **39** S123

Figure S118. ^{1}H NMR spectrum of compound **40** S124

Figure S119. ^{13}C NMR spectrum of compound **40** S125

Figure S120. HRMS spectrum of compound **40** S126

Figure S121. ^{1}H NMR spectrum of compound **41** S127

Figure S122. ^{13}C NMR spectrum of compound **41** S128

Figure S123. HRMS spectrum of compound **41** S129

Figure S124. ^{1}H NMR spectrum of compound **42** S130

Figure S125. ^{13}C NMR spectrum of compound **42** S131

Figure S126. HRMS spectrum of compound **42** S132

Figure S127. ^{1}H NMR spectrum of compound **43** S133

Figure S128. ^{13}C NMR spectrum of compound **43** S134

Figure S129. HRMS spectrum of compound **43** S135

Figure S130. ^{1}H NMR spectrum of compound **44** S136

Figure S131. ^{13}C NMR spectrum of compound **44** S137

Figure S132. HRMS spectrum of compound **44** S138

Figure S133. Docking poses of compound **14** (**A**), compound **20** (**B**), compound **21** (**C**), compound **36** (**D**), compound **39** (**E**), and AAZ (**F**) within the catalytic domain of hCA IX (PDB ID: 3IAI) S139

Figure S134. Docking poses of compound **4** (**A**), compound **9** (**B**), compound **15** (**C**), compound **16** (**D**), compound **17** (**E**), compound **18** (**F**), compound **20** (**G**), compound **29** (**H**), compound **30** (**I**), and AAZ (**J**) in the catalytic domain of hCA XII (PDB ID: 1JD0) S140

Figure S1. ^{1}H NMR spectrum of compound **1**

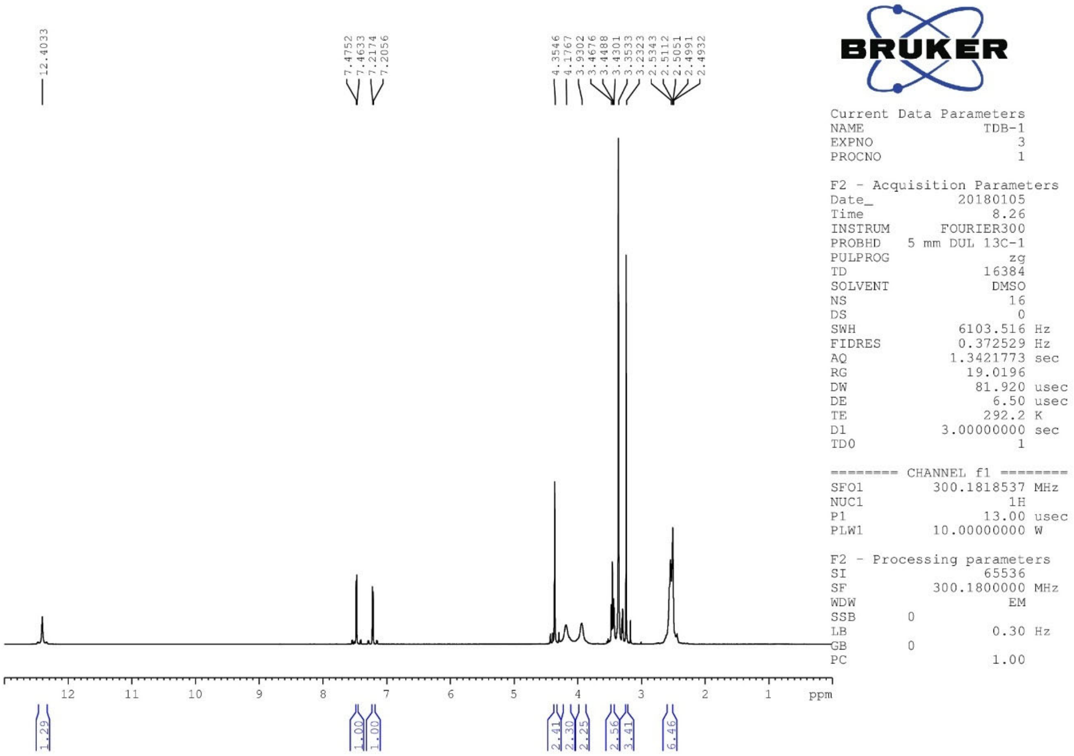

Figure S2. ^{13}C NMR spectrum of compound **1**

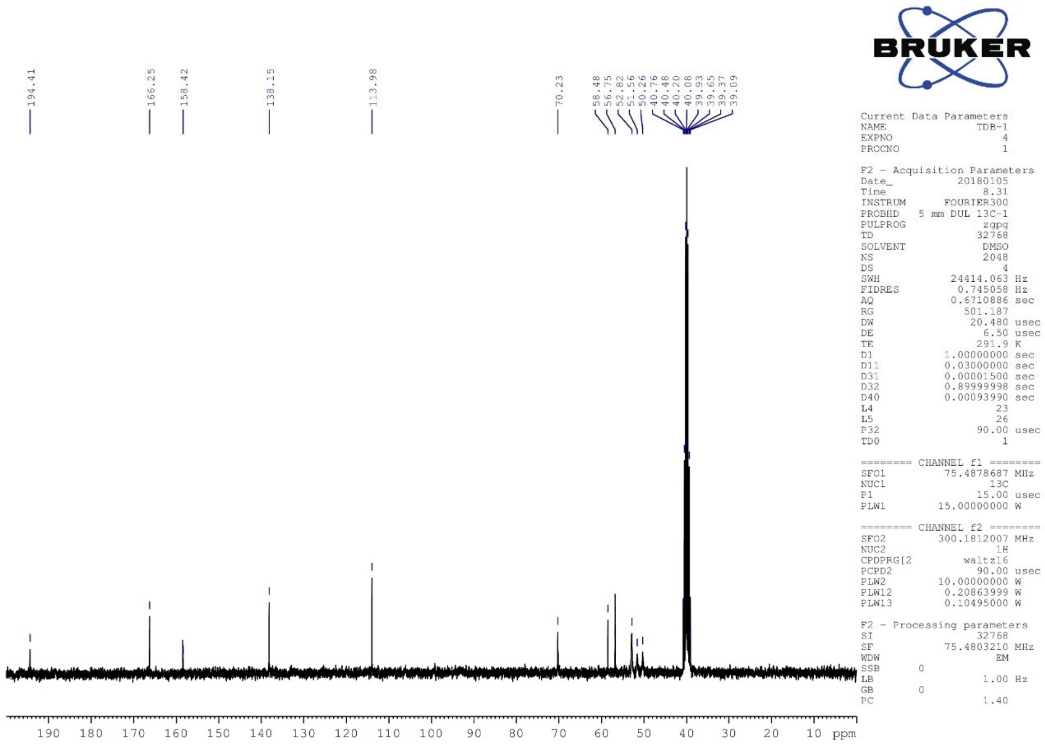

Figure S3. HRMS spectrum of compound **1**

Formula Predictor Report - TDB-1_23.lcd

Data File: C:\LabSolutions\Data\Analiz\mdaltintop\TDB-1_23.lcd

Elmt	Val.	Min	Max	Elmt	Val.	Min	Max	Elmt	Val.	Min	Max	Elmt	Val.	Min	Max	Use Adduct
H	1	5	40	O	2	0	4	S	2	0	3	Ru	2	0	0	H
C	4	0	35	F	1	0	0	Cl	1	0	0	I	3	0	0	
N	3	3	5	P	3	0	0	Br	1	0	0					

Error Margin (ppm): 5
HC Ratio: unlimited
Max Isotopes: 3
MSn Iso RI (%): 10.00

DBE Range: 5.0 - 15.0
Apply N Rule: yes
Isotope RI (%): 1.00
MSn Logic Mode: AND

Electron Ions: both
Use MSn Info: yes
Isotope Res: 9000
Max Results: 500

Event#: 1 MS(E+) Ret. Time : 3.467 -> 4.387 Scan# : 521 -> 659

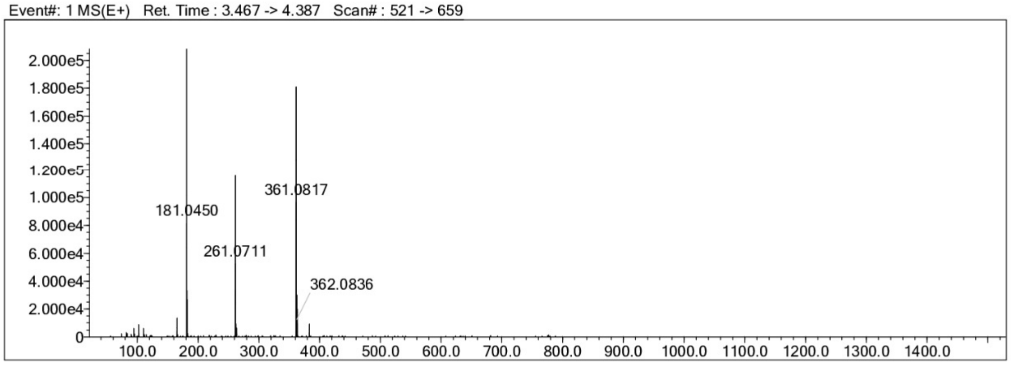

Measured region for 361.0817 m/z

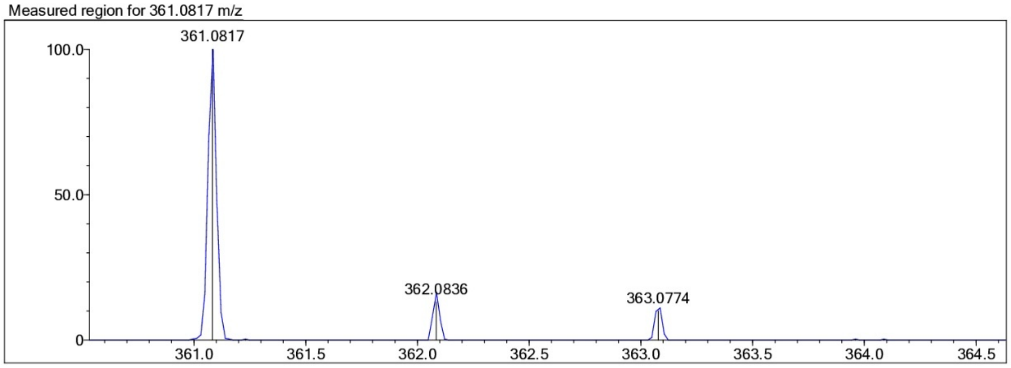

C13 H20 N4 O2 S3 [M+H]+ : Predicted region for 361.0821 m/z

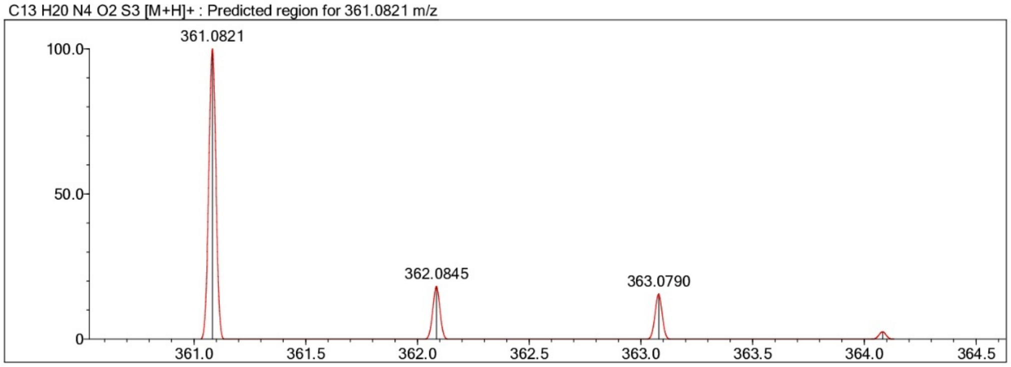

Rank	Score	Formula (M)	Ion	Meas. m/z	Pred. m/z	Df. (mDa)	Df. (ppm)	Iso	DBE
1	70.85	C13 H20 N4 O2 S3	[M+H]+	361.0817	361.0821	-0.4	-1.11	71.04	6.0

Figure S4. ^{1}H NMR spectrum of compound **2**

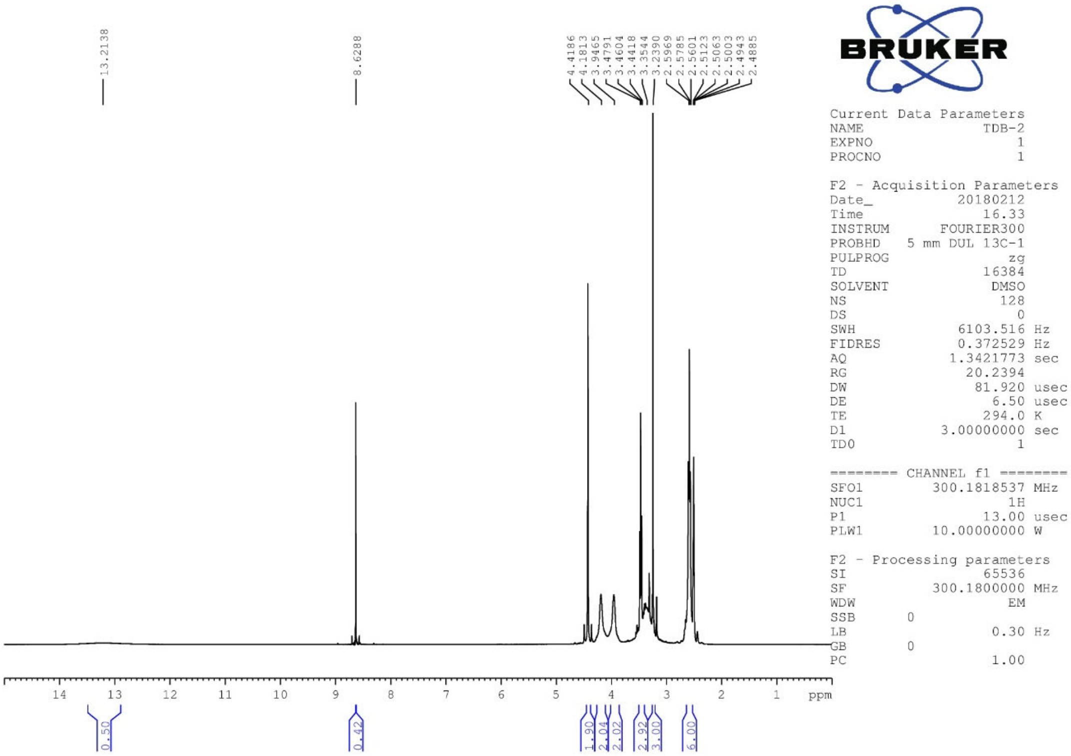

Figure S5. ^{13}C NMR spectrum of compound **2**

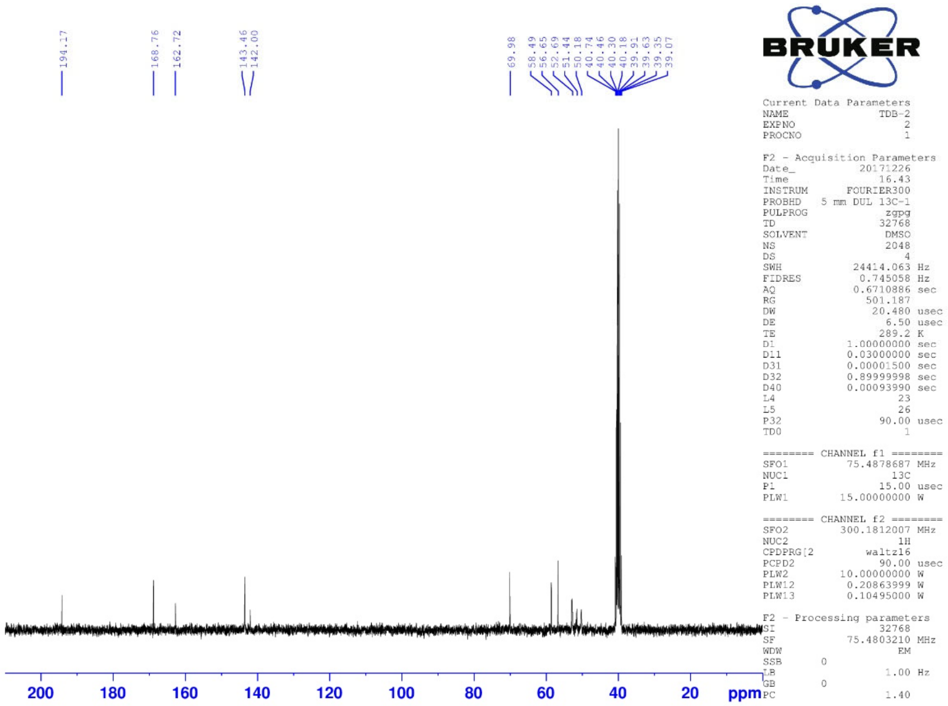

Figure S6. HRMS spectrum of compound **2**

Formula Predictor Report - TDB-2_24.lcd

Data File: C:\LabSolutions\Data\Analiz\mdaltintop\TDB-2_24.lcd

Elmt	Val.	Min	Max	Elmt	Val.	Min	Max	Elmt	Val.	Min	Max	Elmt	Val.	Min	Max	Use Adduct
H	1	5	40	O	2	0	5	S	2	0	3	Ru	2	0	0	H
C	4	0	35	F	1	0	0	Cl	1	0	0	I	3	0	0	
N	3	3	6	P	3	0	0	Br	1	0	0					

Error Margin (ppm): 5
HC Ratio: unlimited
Max Isotopes: 3
MSn Iso RI (%): 10.00

DBE Range: 5.0 - 15.0
Apply N Rule: yes
Isotope RI (%): 1.00
MSn Logic Mode: AND

Electron Ions: both
Use MSn Info: yes
Isotope Res: 9000
Max Results: 500

Event#: 1 MS(E+) Ret. Time : 4.720 -> 5.187 Scan# : 709 -> 779

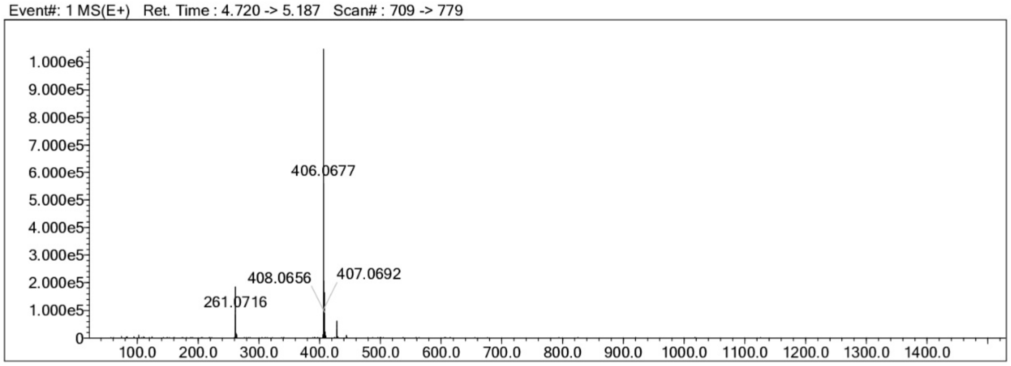

Measured region for 406.0677 m/z

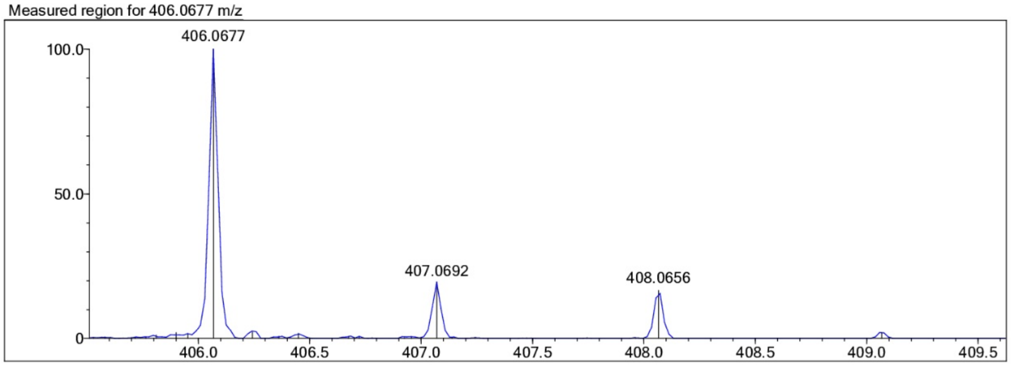

C13 H19 N5 O4 S3 [M+H]+ : Predicted region for 406.0672 m/z

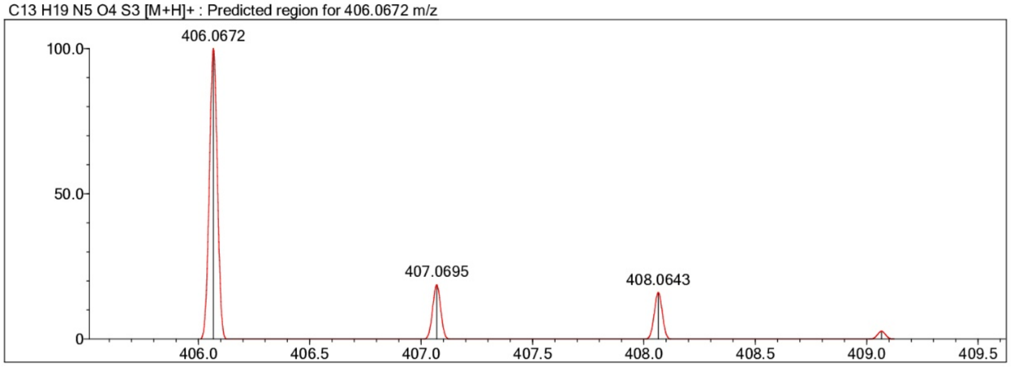

Rank	Score	Formula (M)	Ion	Meas. m/z	Pred. m/z	Df. (mDa)	Df. (ppm)	Iso	DBE
1	99.42	C13 H19 N5 O4 S3	[M+H]+	406.0677	406.0672	0.5	1.23	100.00	7.0

Figure S7. ^{1}H NMR spectrum of compound **3**

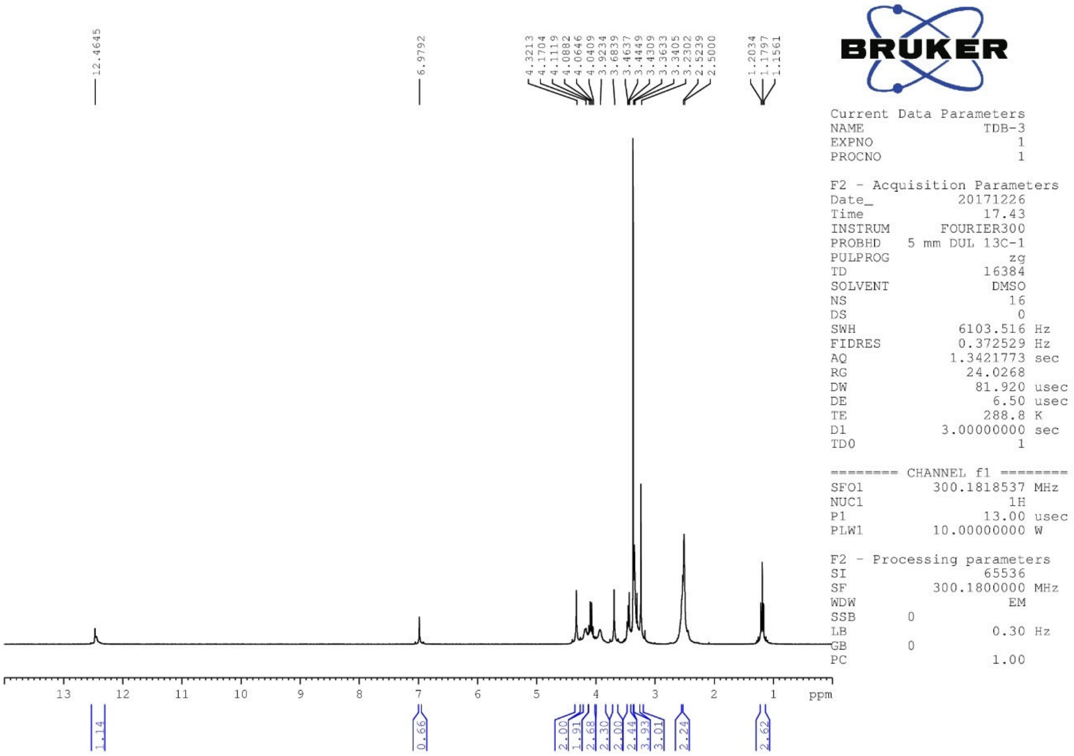

Figure S8. ^{13}C NMR spectrum of compound **3**

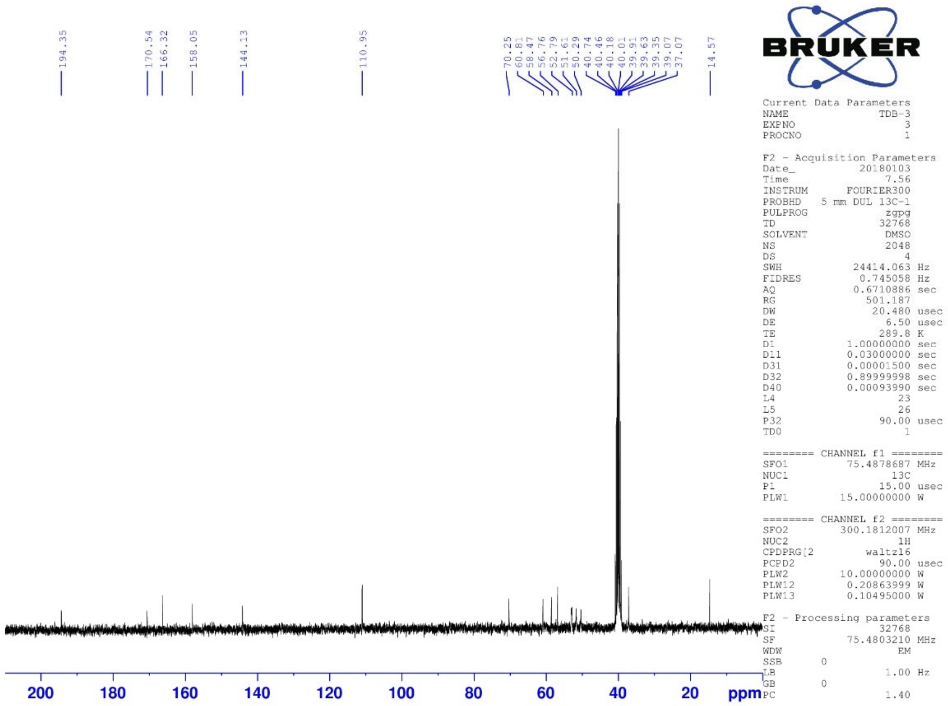

Figure S9. HRMS spectrum of compound **3**

Formula Predictor Report - TDB-3_24.lcd | Page 1 of 1

Data File: C:\LabSolutions\Data\Analiz\mdaltintop\TDB-3_24.lcd

Elmt	Val.	Min	Max	Elmt	Val.	Min	Max	Elmt	Val.	Min	Max	Elmt	Val.	Min	Max	Use Adduct
H	1	5	40	O	2	0	4	S	2	0	3	Ru	2	0	0	H
C	4	0	35	F	1	0	0	Cl	1	0	0	I	3	0	0	
N	3	3	5	P	3	0	0	Br	1	0	0					

Error Margin (ppm):	5	DBE Range:	5.0 - 15.0	Electron Ions:	both
HC Ratio:	unlimited	Apply N Rule:	yes	Use MSn Info:	yes
Max Isotopes:	3	Isotope RI (%):	1.00	Isotope Res:	9000
MSn Iso RI (%):	10.00	MSn Logic Mode:	AND	Max Results:	500

Event#: 1 MS(E+) Ret. Time : 4.707 -> 5.400 Scan# : 707 -> 811

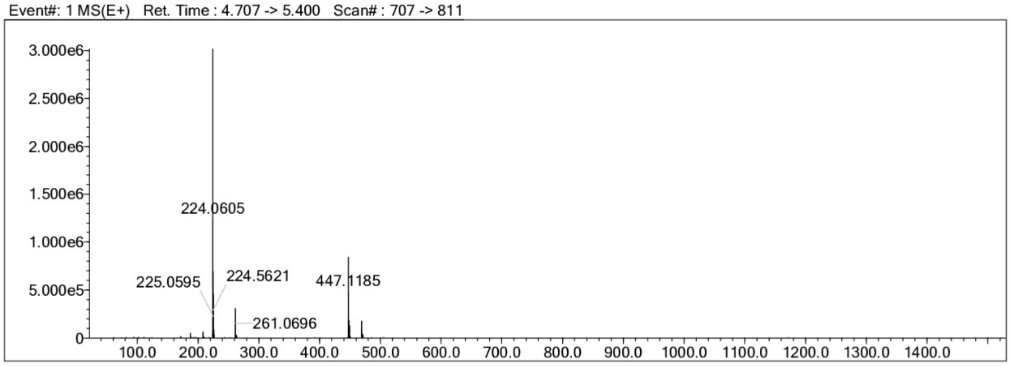

Measured region for 447.1185 m/z

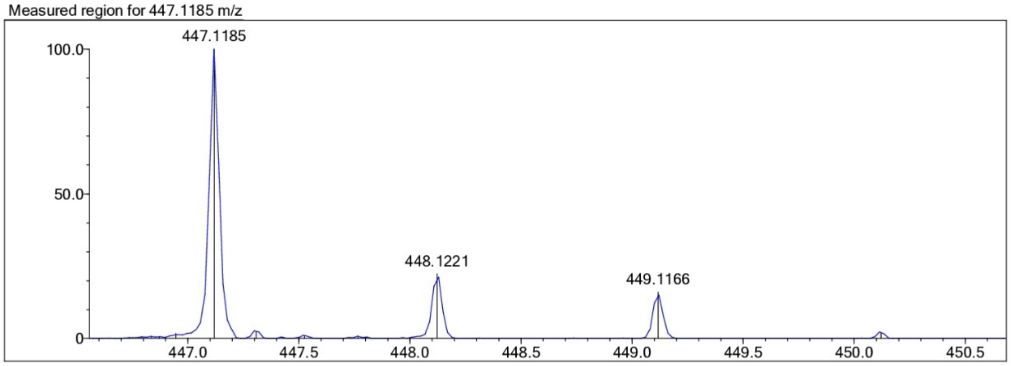

C17 H26 N4 O4 S3 [M+H]+ : Predicted region for 447.1189 m/z

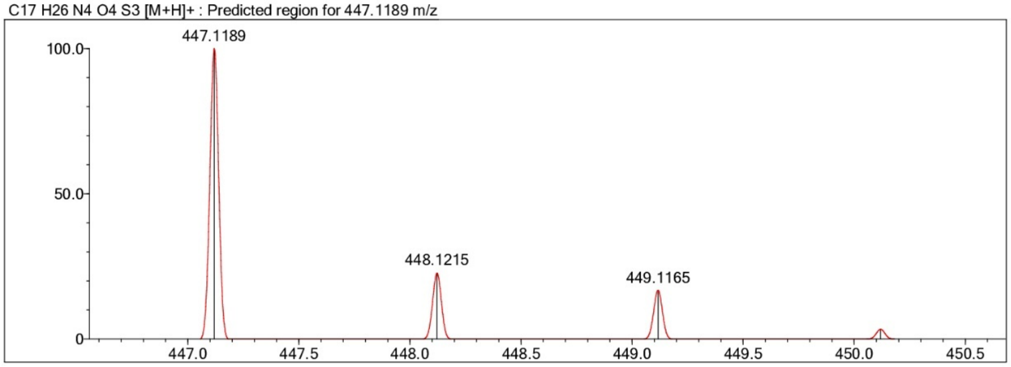

Rank	Score	Formula (M)	Ion	Meas. m/z	Pred. m/z	Df. (mDa)	Df. (ppm)	Iso	DBE
1	94.71	C17 H26 N4 O4 S3	[M+H]+	447.1185	447.1189	-0.4	-0.89	94.71	7.0

Figure S10. ^{1}H NMR spectrum of compound **4**

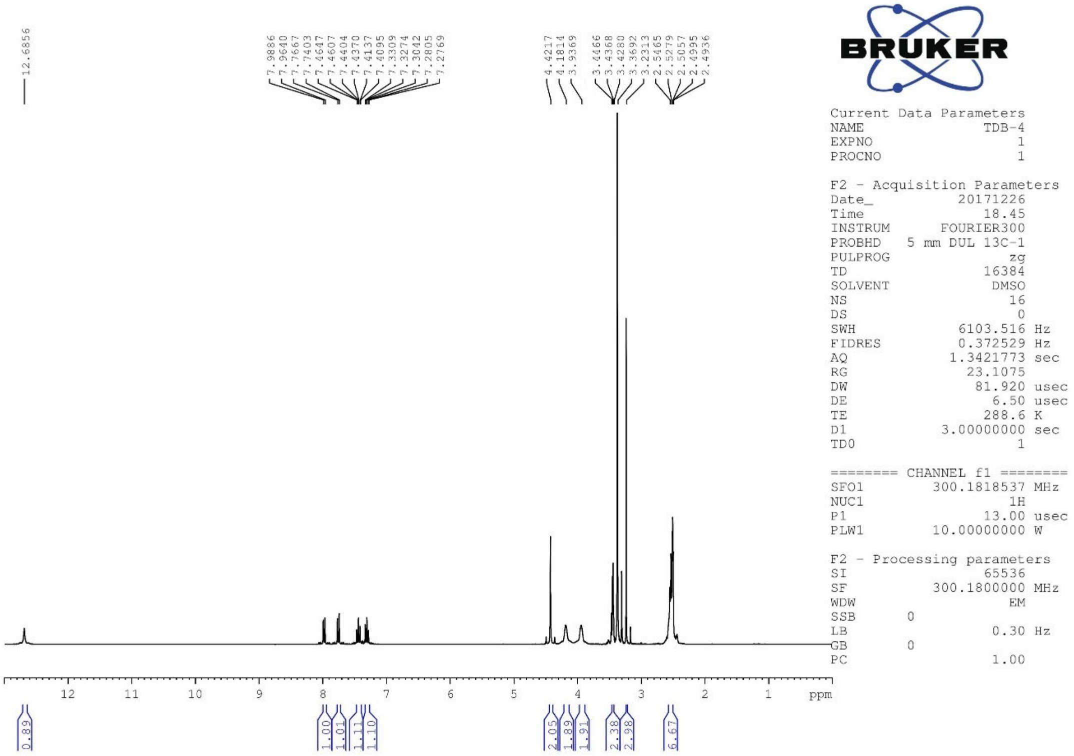

Figure S11. ^{13}C NMR spectrum of compound **4**

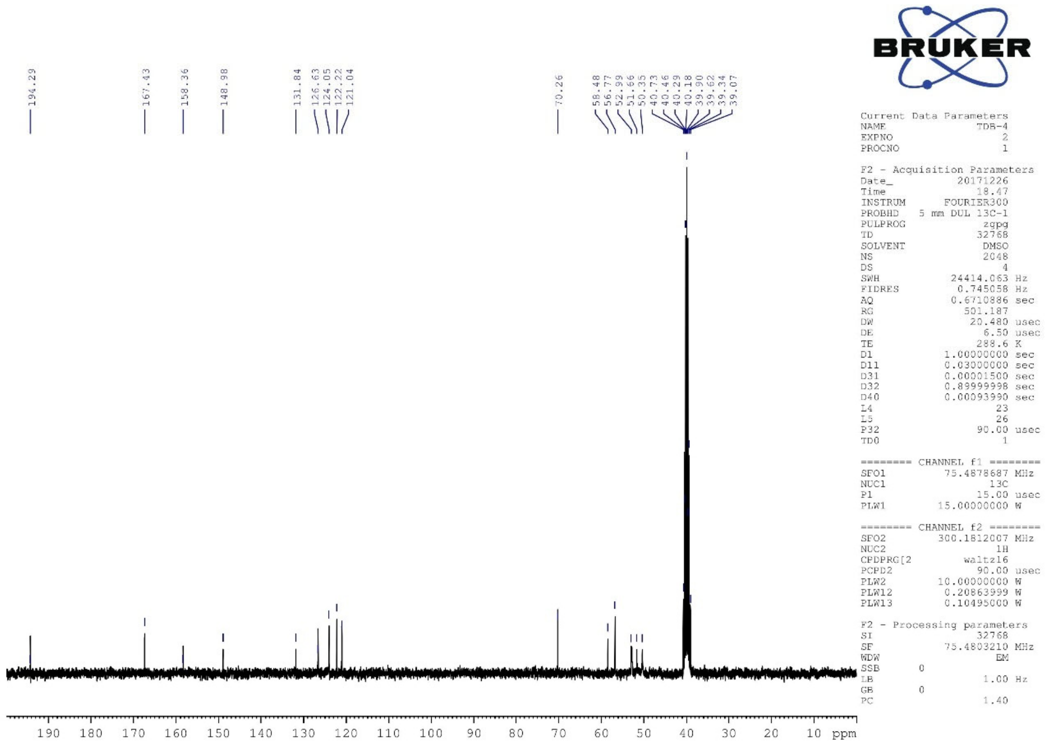

Figure S12. HRMS spectrum of compound **4**

Formula Predictor Report - TDB-4_25.lcd | Page 1 of 1

Data File: C:\LabSolutions\Data\Analiz\mdaltintop\TDB-4_25.lcd

Elmt	Val.	Min	Max	Elmt	Val.	Min	Max	Elmt	Val.	Min	Max	Elmt	Val.	Min	Max	Use Adduct
H	1	5	40	O	2	0	4	S	2	0	3	Ru	2	0	0	H
C	4	0	35	F	1	0	0	Cl	1	0	0	I	3	0	0	
N	3	3	5	P	3	0	0	Br	1	0	0					

Error Margin (ppm):	5	DBE Range:	5.0 - 15.0	Electron Ions:	both
HC Ratio:	unlimited	Apply N Rule:	yes	Use MSn Info:	yes
Max Isotopes:	3	Isotope RI (%):	1.00	Isotope Res:	9000
MSn Iso RI (%):	10.00	MSn Logic Mode:	AND	Max Results:	500

Event#: 1 MS(E+) Ret. Time : 5.040 -> 5.547 Scan# : 757 -> 833

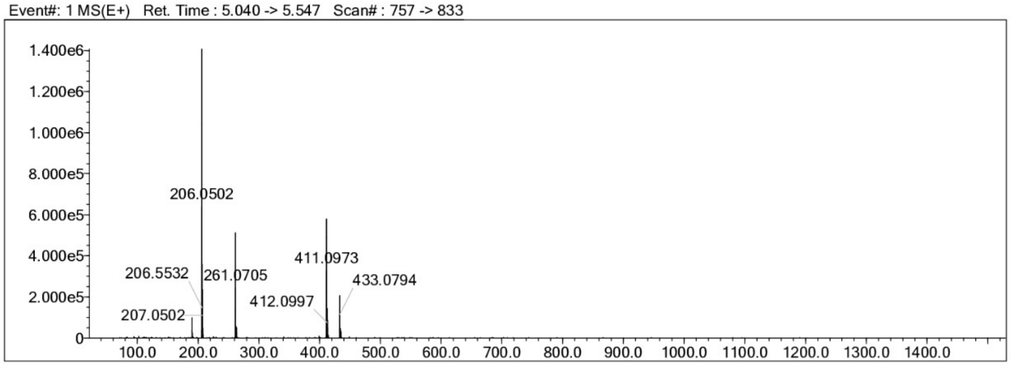

Measured region for 411.0973 m/z

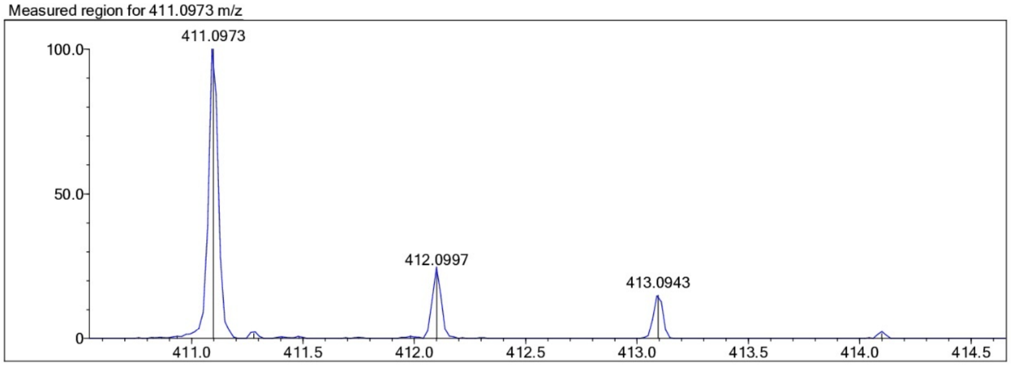

C17 H22 N4 O2 S3 [M+H]+ : Predicted region for 411.0978 m/z

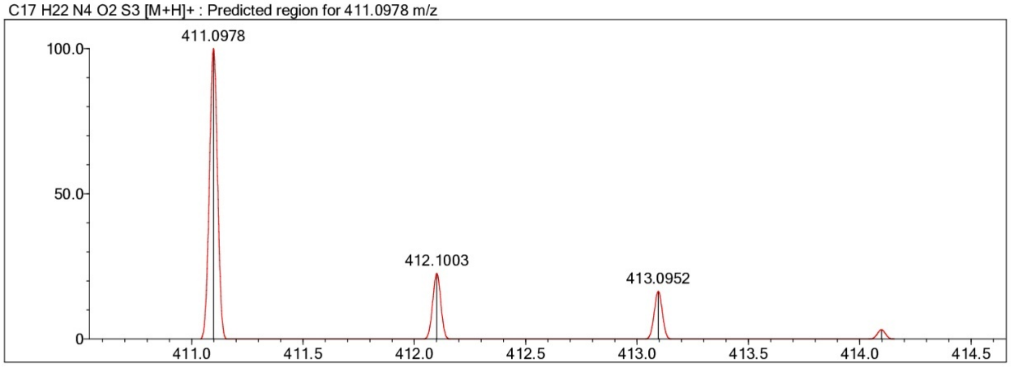

Rank	Score	Formula (M)	Ion	Meas. m/z	Pred. m/z	Df. (mDa)	Df. (ppm)	Iso	DBE
1	88.91	C17 H22 N4 O2 S3	[M+H]+	411.0973	411.0978	-0.5	-1.22	89.40	9.0

Figure S13. ^{1}H NMR spectrum of compound **5**

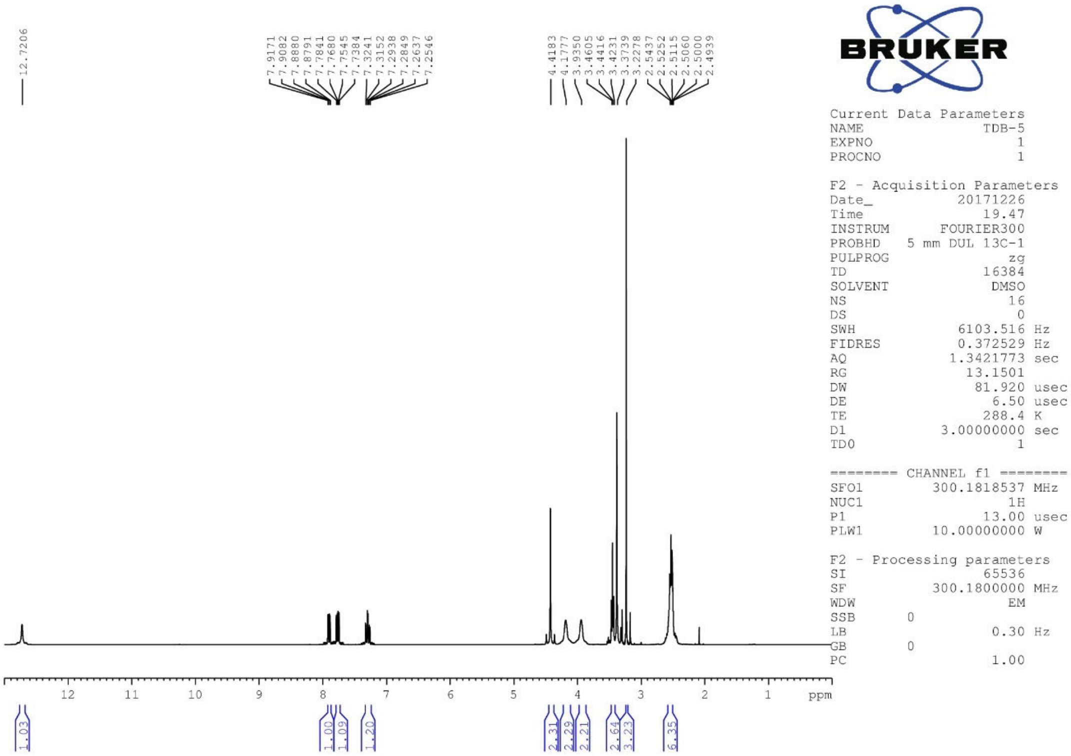

Figure S14. ^{13}C NMR spectrum of compound **5**

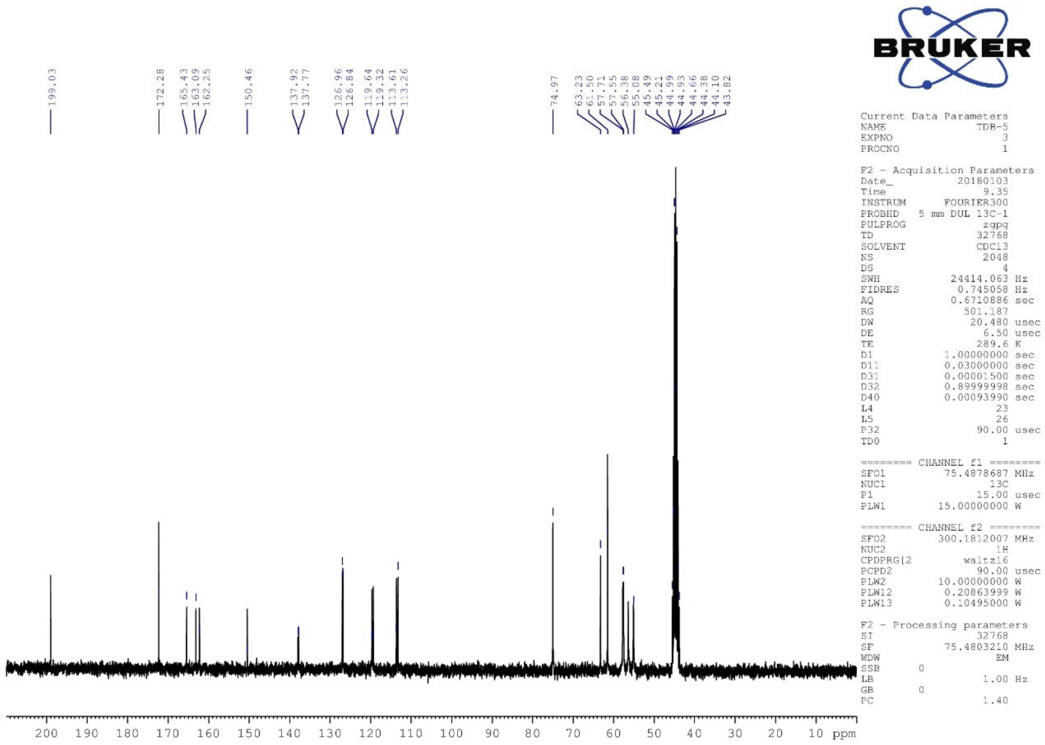

Figure S15. HRMS spectrum of compound **5**

Formula Predictor Report - TDB-5_26.lcd | Page 1 of 1

Data File: C:\LabSolutions\Data\Analiz\mdaltintop\TDB-5_26.lcd

Elmt	Val.	Min	Max	Elmt	Val.	Min	Max	Elmt	Val.	Min	Max	Elmt	Val.	Min	Max	Use Adduct
H	1	5	40	O	2	0	4	S	2	0	3	Ru	2	0	0	H
C	4	0	35	F	1	0	1	Cl	1	0	0	I	3	0	0	
N	3	3	5	P	3	0	0	Br	1	0	0					

Error Margin (ppm): 5
HC Ratio: unlimited
Max Isotopes: 3
MSn Iso RI (%): 10.00

DBE Range: 5.0 - 10.0
Apply N Rule: yes
Isotope RI (%): 1.00
MSn Logic Mode: AND

Electron Ions: both
Use MSn Info: yes
Isotope Res: 9000
Max Results: 500

Event#: 1 MS(E+) Ret. Time : 5.173 -> 5.840 Scan# : 777 -> 877

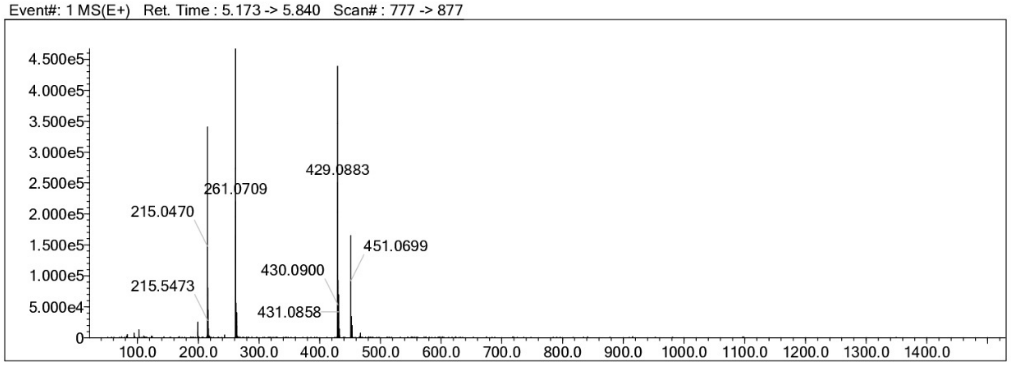

Measured region for 429.0883 m/z

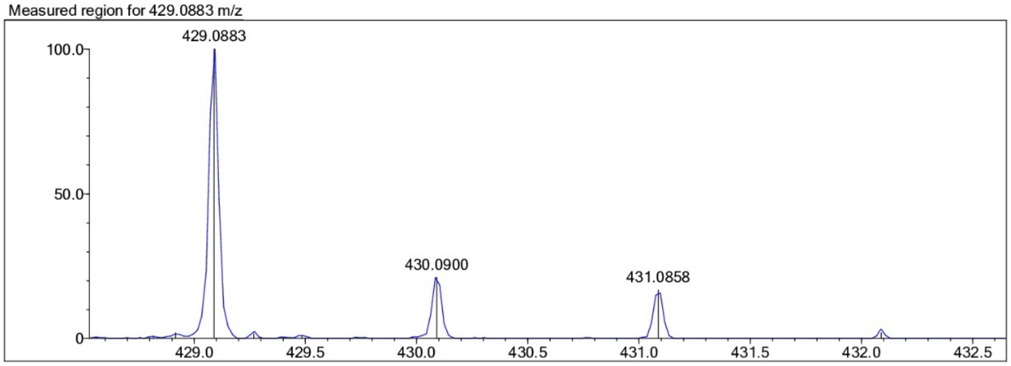

C17 H21 N4 O2 F S3 [M+H]+ : Predicted region for 429.0883 m/z

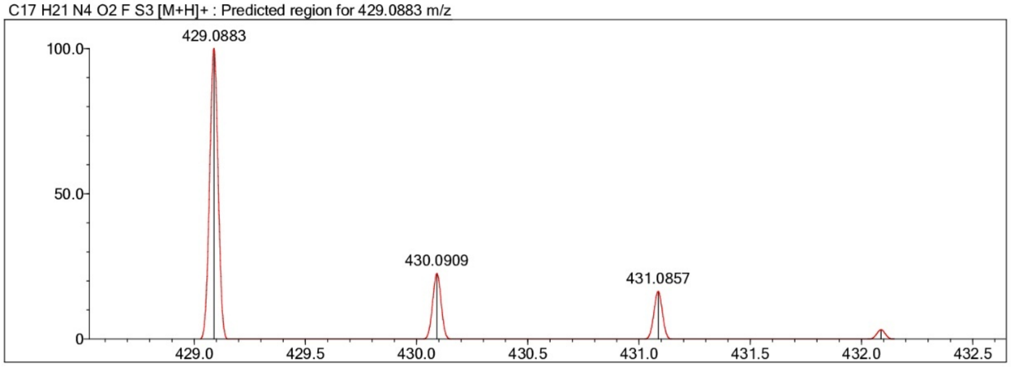

Rank	Score	Formula (M)	Ion	Meas. m/z	Pred. m/z	Df. (mDa)	Df. (ppm)	Iso	DBE
1	94.03	C17 H21 N4 O2 F S3	[M+H]+	429.0883	429.0883	-0.0	0.00	94.03	9.0

Figure S16. ^{1}H NMR spectrum of compound **6**

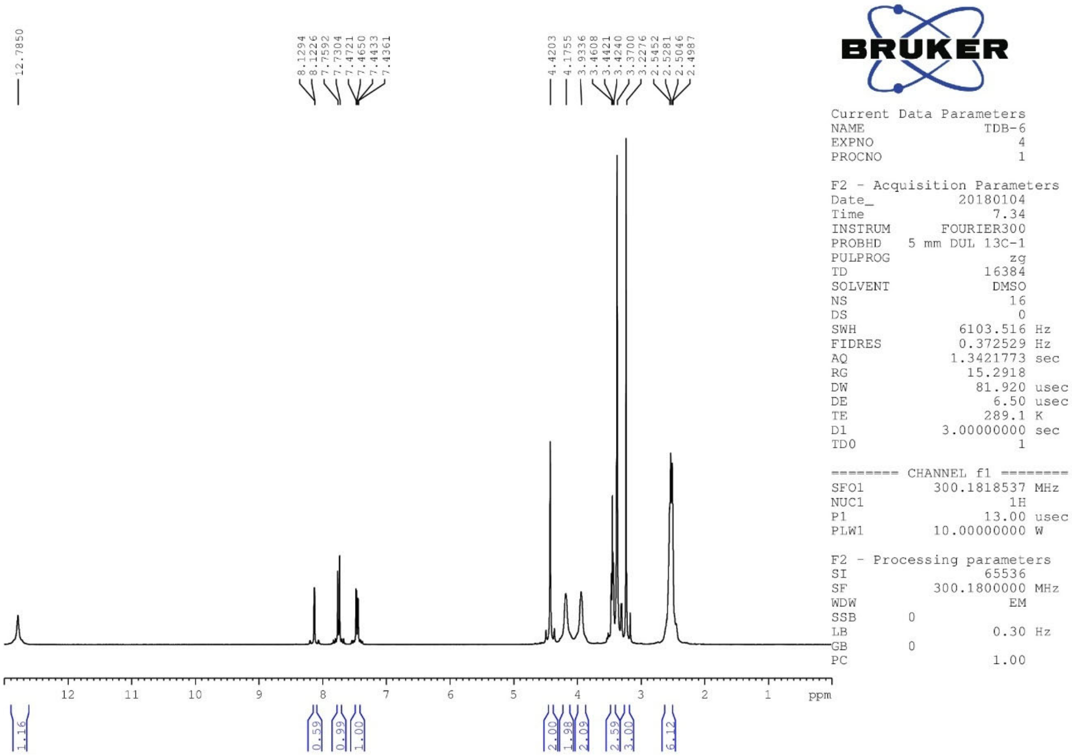

Figure S17. ^{13}C NMR spectrum of compound **6**

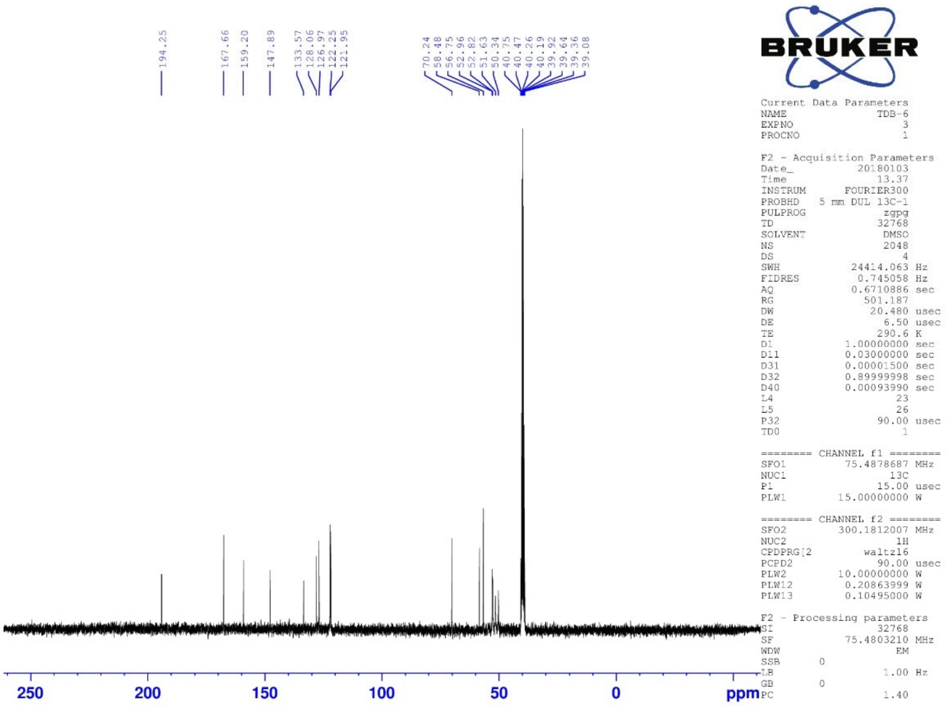

Figure S18. HRMS spectrum of compound **6**

Formula Predictor Report - TDB-6_27.lcd Page 1 of 1

Data File: C:\LabSolutions\Data\Analiz\mdaltintop\TDB-6_27.lcd

Elmt	Val.	Min	Max	Elmt	Val.	Min	Max	Elmt	Val.	Min	Max	Elmt	Val.	Min	Max	Use Adduct
H	1	5	40	O	2	0	4	S	2	0	3	Ru	2	0	0	H
C	4	0	35	F	1	0	0	Cl	1	0	1	I	3	0	0	
N	3	3	5	P	3	0	0	Br	1	0	0					

Error Margin (ppm):	5	DBE Range:	5.0 - 10.0	Electron Ions:	both
HC Ratio:	unlimited	Apply N Rule:	yes	Use MSn Info:	yes
Max Isotopes:	3	Isotope RI (%):	1.00	Isotope Res:	9000
MSn Iso RI (%):	10.00	MSn Logic Mode:	AND	Max Results:	500

Event#: 1 MS(E+) Ret. Time : 5.573 -> 6.013 Scan# : 837 -> 903

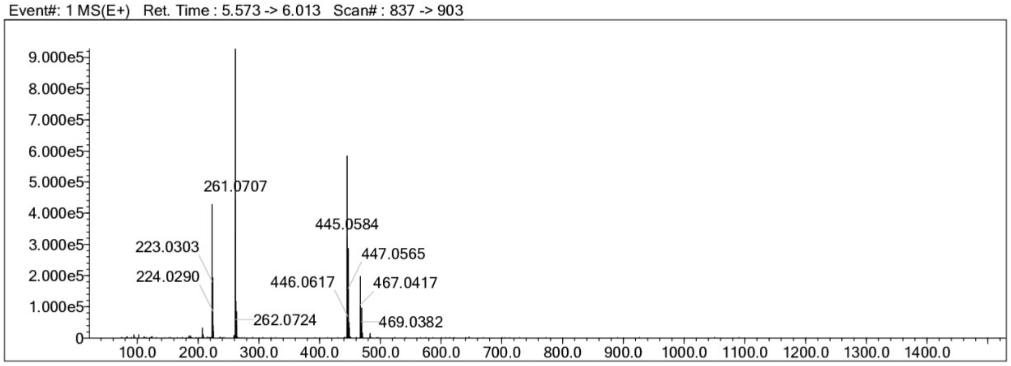

Measured region for 445.0584 m/z

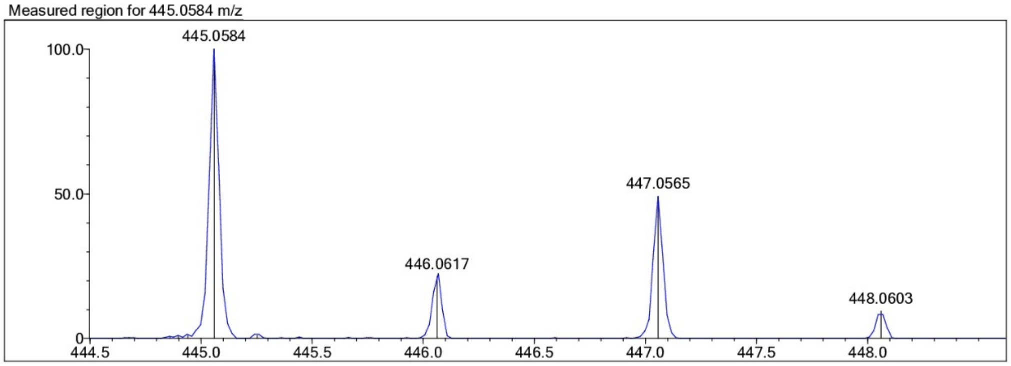

C17 H21 N4 O2 S3 Cl [M+H]+ : Predicted region for 445.0588 m/z

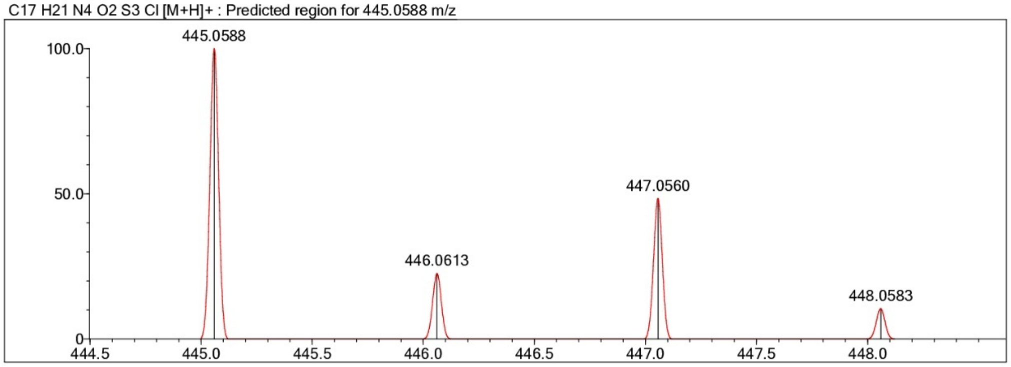

Rank	Score	Formula (M)	Ion	Meas. m/z	Pred. m/z	Df. (mDa)	Df. (ppm)	Iso	DBE
1	97.76	C17 H21 N4 O2 S3 Cl	[M+H]+	445.0584	445.0588	-0.4	-0.90	97.76	9.0

Figure S19. ^{1}H NMR spectrum of compound **7**

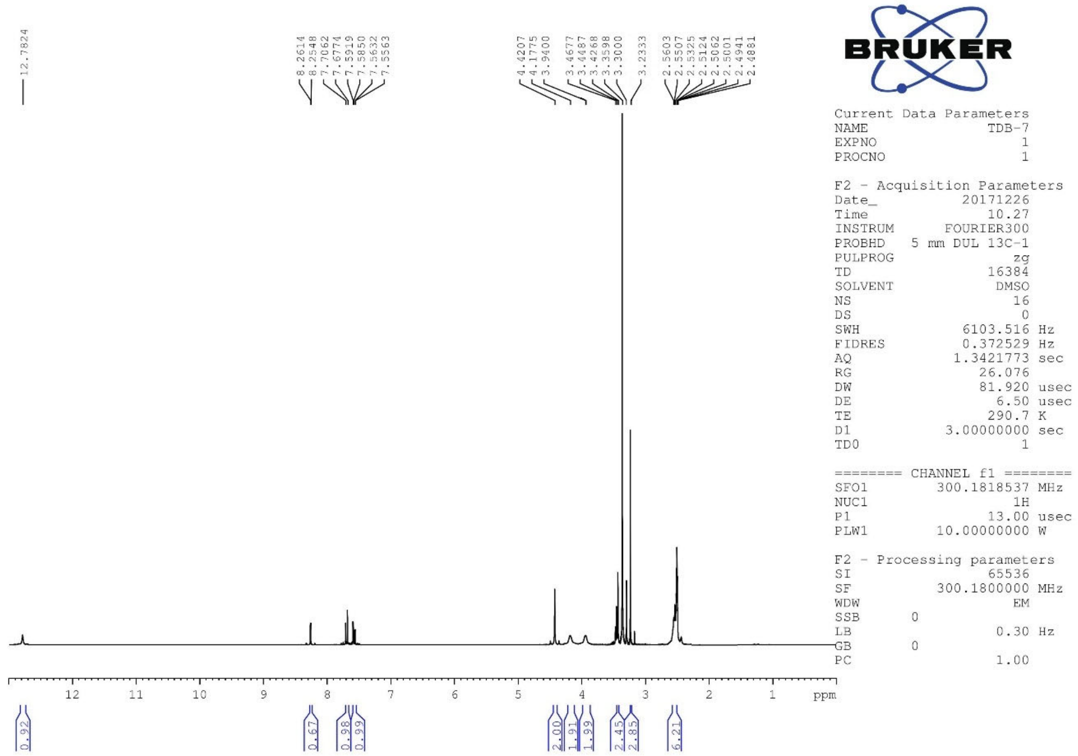

Figure S20. ^{13}C NMR spectrum of compound **7**

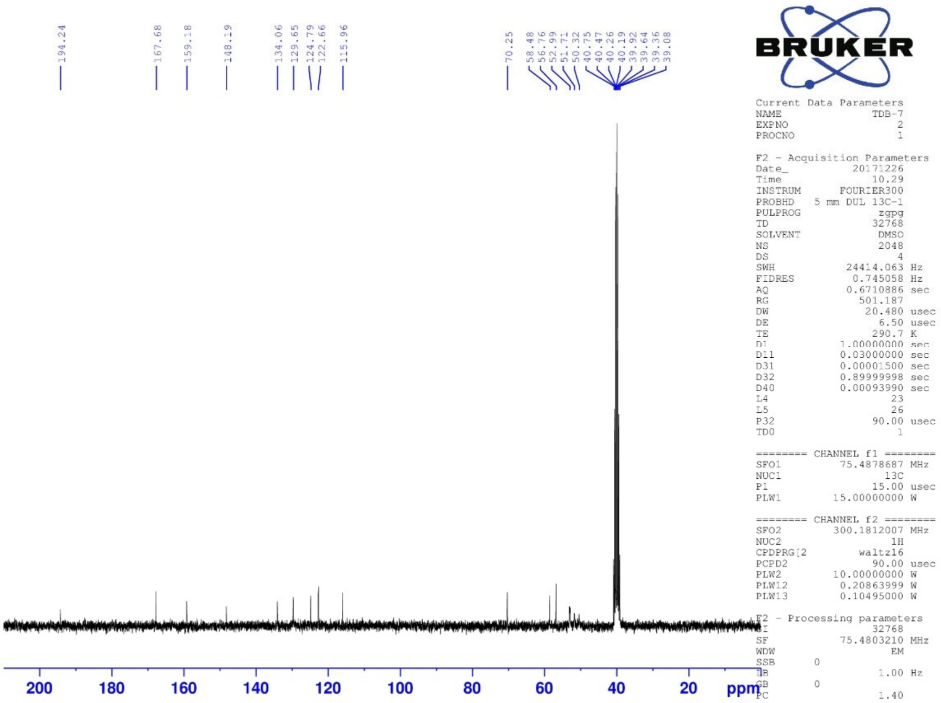

Figure S21. HRMS spectrum of compound **7**

Data File: C:\LabSolutions\Data\Analiz\mdaltintop\TDB-7_25.lcd

Elmt	Val.	Min	Max	Elmt	Val.	Min	Max	Elmt	Val.	Min	Max	Elmt	Val.	Min	Max	Use Adduct
H	1	5	40	O	2	0	5	S	2	0	3	Ru	2	0	0	H
C	4	0	35	F	1	0	0	Cl	1	0	0	I	3	0	0	
N	3	3	6	P	3	0	0	Br	1	0	1					

Error Margin (ppm):	5	DBE Range:	5.0 - 15.0	Electron Ions:	both
HC Ratio:	unlimited	Apply N Rule:	yes	Use MSn Info:	yes
Max Isotopes:	3	Isotope RI (%):	1.00	Isotope Res:	9000
MSn Iso RI (%):	10.00	MSn Logic Mode:	AND	Max Results:	500

Event#: 1 MS(E+) Ret. Time : 5.813 Scan# : 873

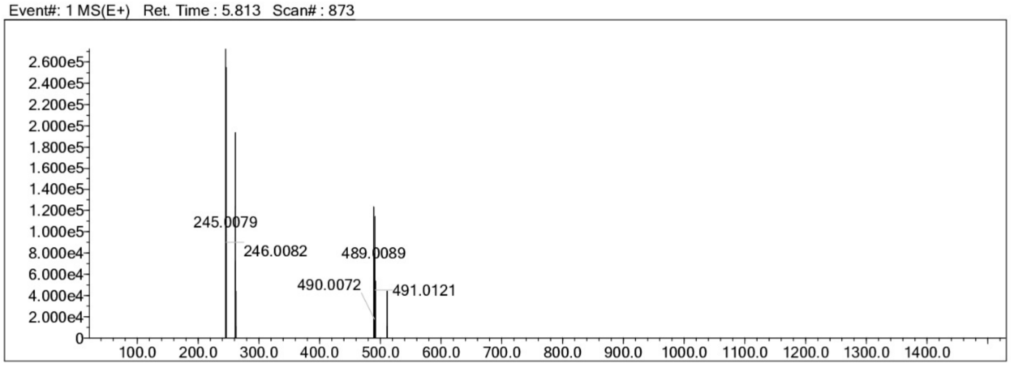

Measured region for 489.0089 m/z

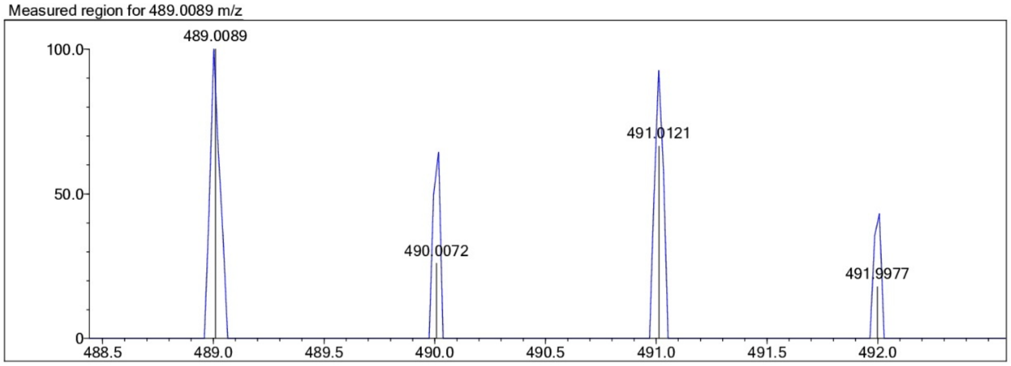

C17 H21 N4 O2 S3 Br [M+H]+ : Predicted region for 489.0083 m/z

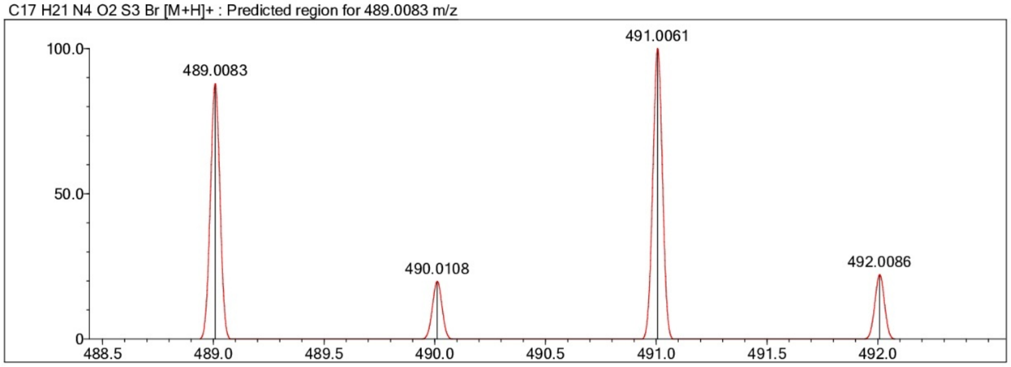

Rank	Score	Formula (M)	Ion	Meas. m/z	Pred. m/z	Df. (mDa)	Df. (ppm)	Iso	DBE
1	30.04	C17 H21 N4 O2 S3 Br	[M+H]+	489.0089	489.0083	0.6	1.23	30.22	9.0

Figure S22. ^1H NMR spectrum of compound **8**

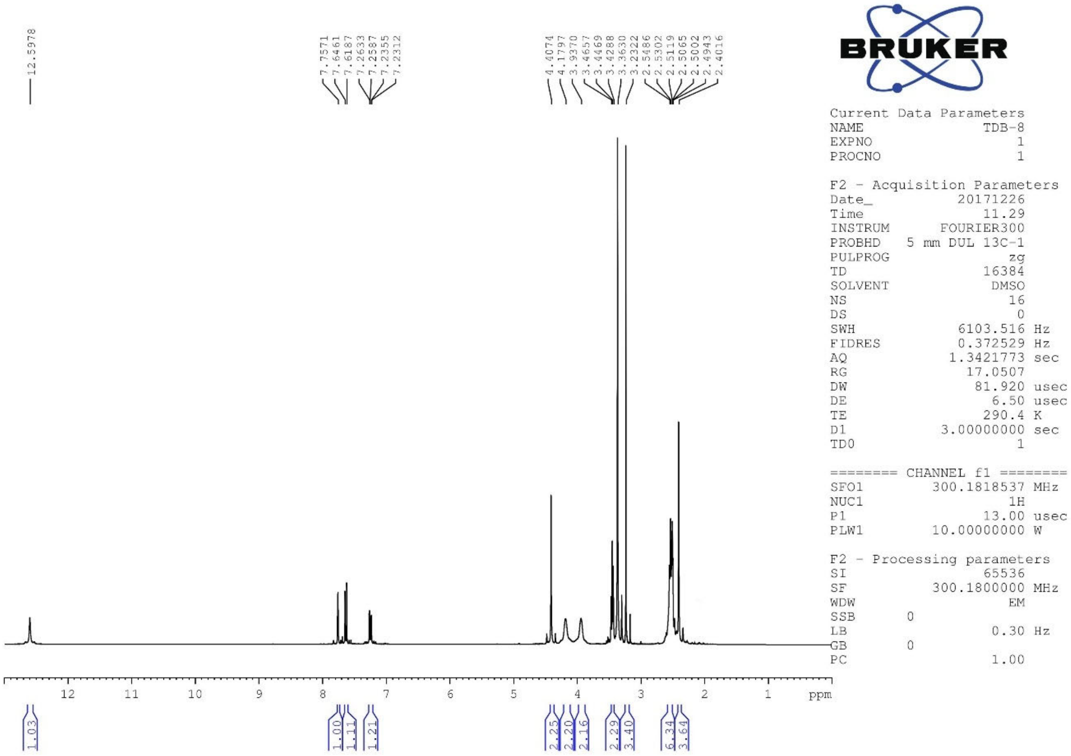

Figure S23. ^{13}C NMR spectrum of compound **8**

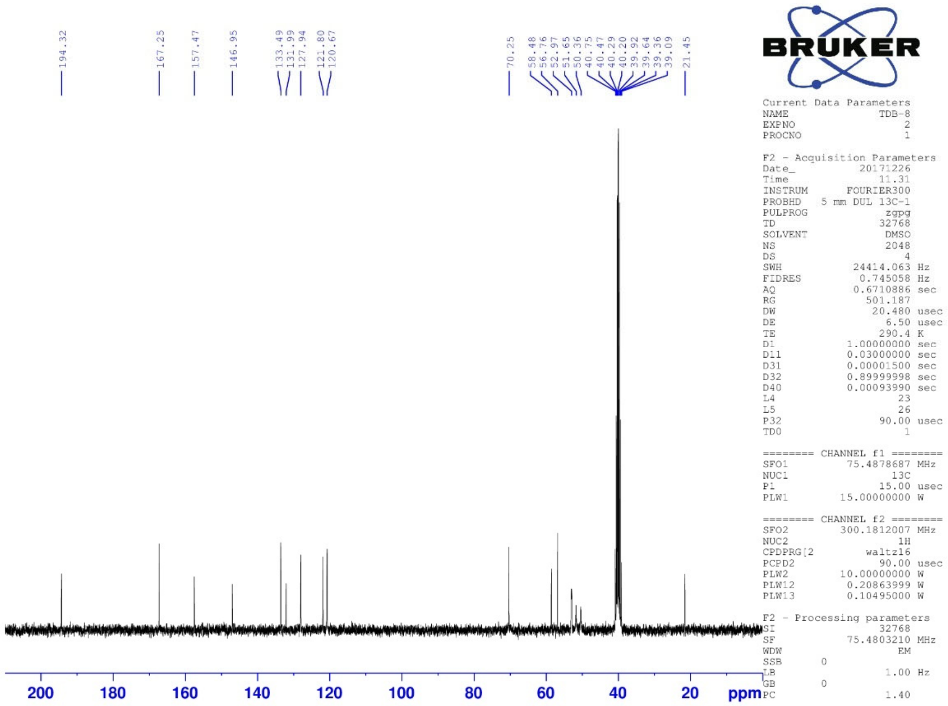

Figure S24. HRMS spectrum of compound **8**

Formula Predictor Report - TDB-8_26.lcd   Page 1 of 1

Data File: C:\LabSolutions\Data\Analiz\mdaltintop\TDB-8_26.lcd

Elmt	Val.	Min	Max	Elmt	Val.	Min	Max	Elmt	Val.	Min	Max	Elmt	Val.	Min	Max	Use Adduct
H	1	5	40	O	2	0	5	S	2	0	3	Ru	2	0	0	H
C	4	0	35	F	1	0	0	Cl	1	0	0	I	3	0	0	
N	3	3	6	P	3	0	0	Br	1	0	1					

Error Margin (ppm):	5	DBE Range:	5.0 - 15.0	Electron Ions:	both
HC Ratio:	unlimited	Apply N Rule:	yes	Use MSn Info:	yes
Max Isotopes:	3	Isotope RI (%):	1.00	Isotope Res:	9000
MSn Iso RI (%):	10.00	MSn Logic Mode:	AND	Max Results:	500

Event#: 1 MS(E+)   Ret. Time : 5.400 -> 5.827   Scan# : 811 -> 875

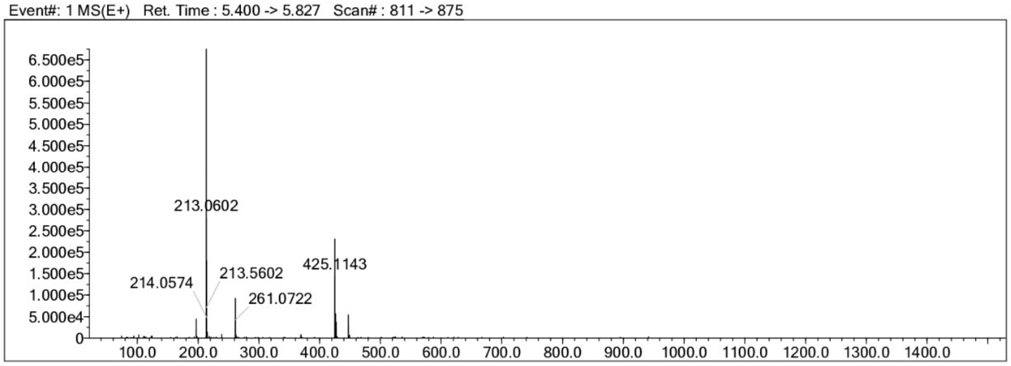

Measured region for 425.1143 m/z

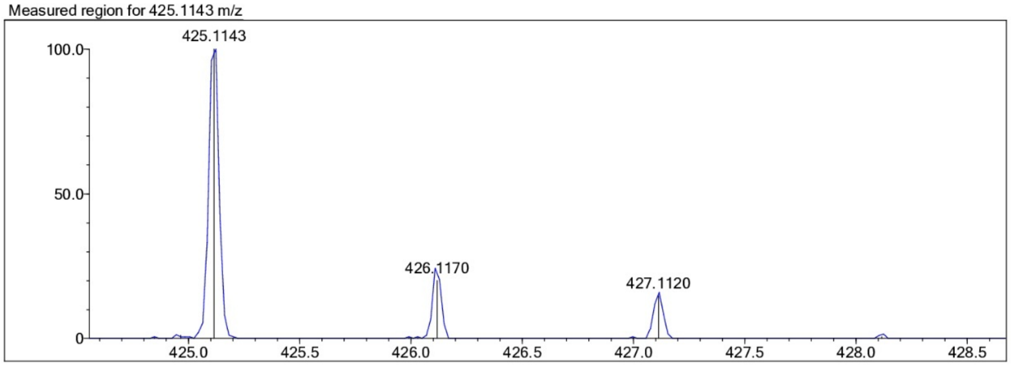

C18 H24 N4 O2 S3 [M+H]+ : Predicted region for 425.1134 m/z

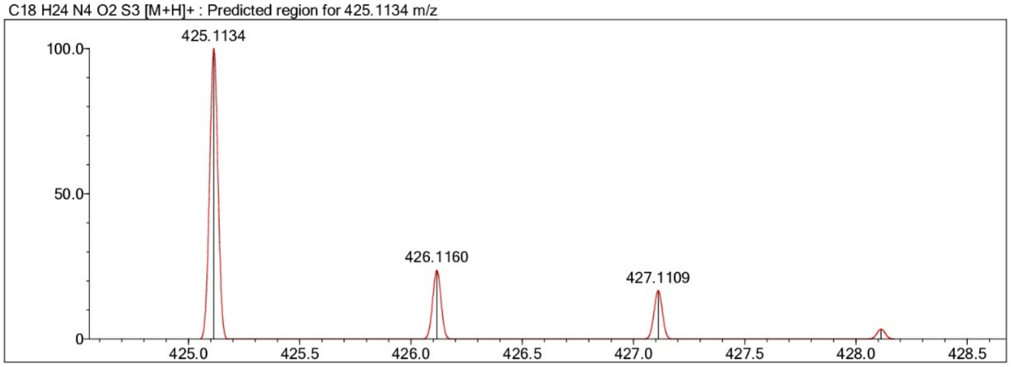

Rank	Score	Formula (M)	Ion	Meas. m/z	Pred. m/z	Df. (mDa)	Df. (ppm)	Iso	DBE
1	87.49	C18 H24 N4 O2 S3	[M+H]+	425.1143	425.1134	0.9	2.12	90.01	9.0

Figure S25. ^{1}H NMR spectrum of compound **9**

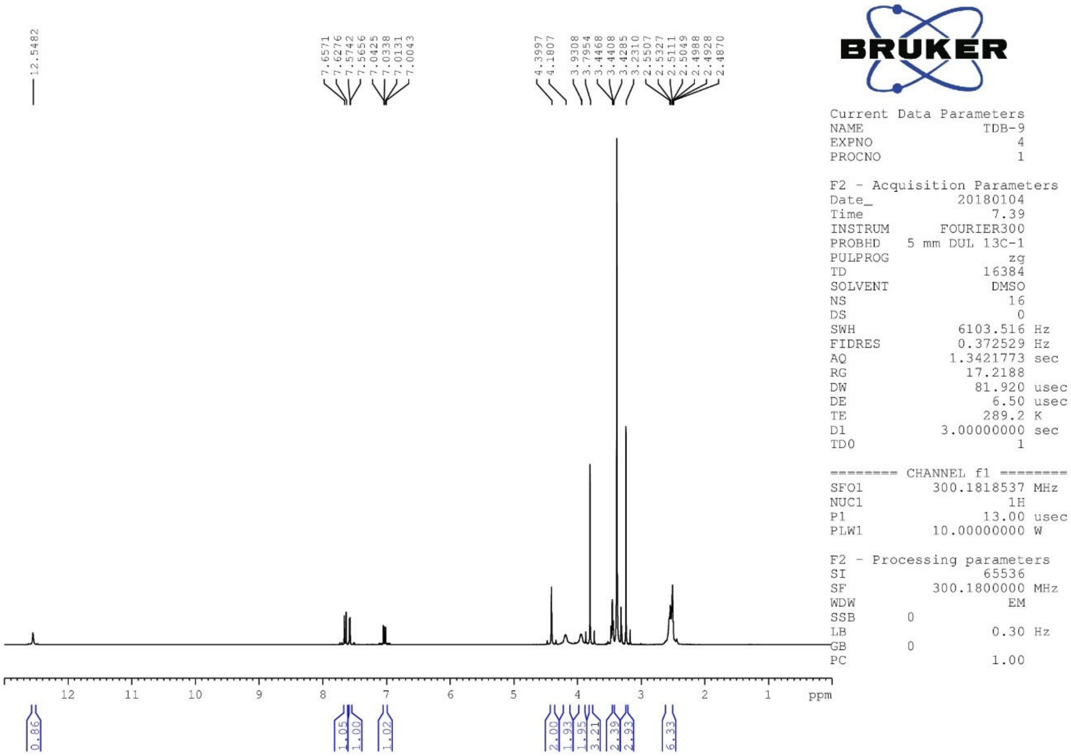

Figure S26. ^{13}C NMR spectrum of compound **9**

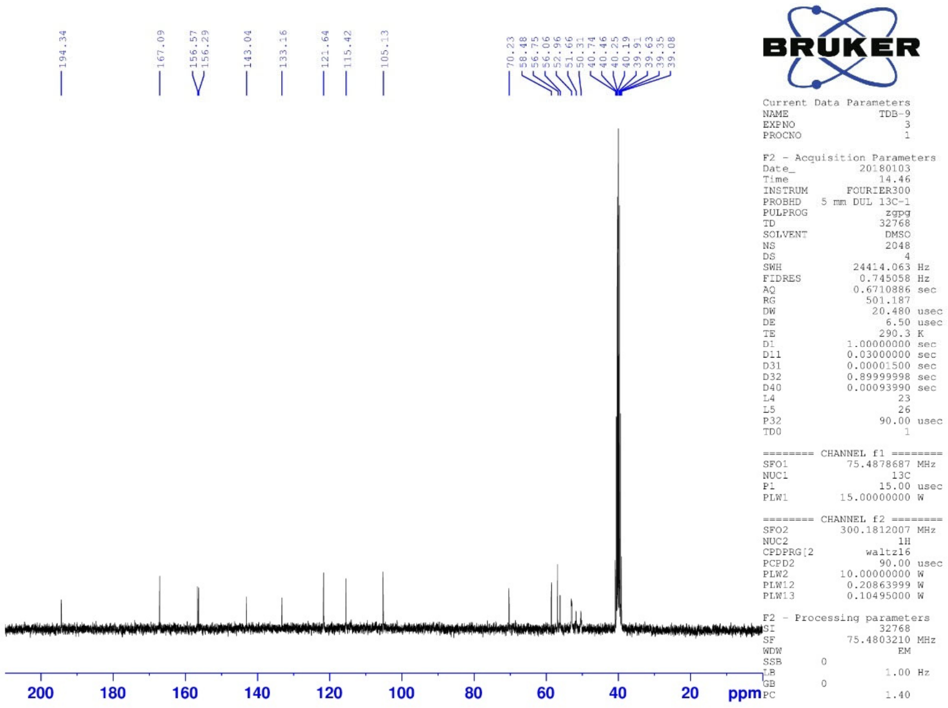

Figure S27. HRMS spectrum of compound **9**

Formula Predictor Report - TDB-9_27.lcd   Page 1 of 1

Data File: C:\LabSolutions\Data\Analiz\mdaltintop\TDB-9_27.lcd

Elmt	Val.	Min	Max	Elmt	Val.	Min	Max	Elmt	Val.	Min	Max	Elmt	Val.	Min	Max	Use Adduct
H	1	5	40	O	2	0	5	S	2	0	3	Ru	2	0	0	H
C	4	0	35	F	1	0	0	Cl	1	0	0	I	3	0	0	
N	3	3	6	P	3	0	0	Br	1	0	1					

Error Margin (ppm): 5
HC Ratio: unlimited
Max Isotopes: 3
MSn Iso RI (%): 10.00

DBE Range: 5.0 - 15.0
Apply N Rule: yes
Isotope RI (%): 1.00
MSn Logic Mode: AND

Electron Ions: both
Use MSn Info: yes
Isotope Res: 9000
Max Results: 500

Event#: 1 MS(E+) Ret. Time : 5.040 -> 6.133 Scan# : 757 -> 921

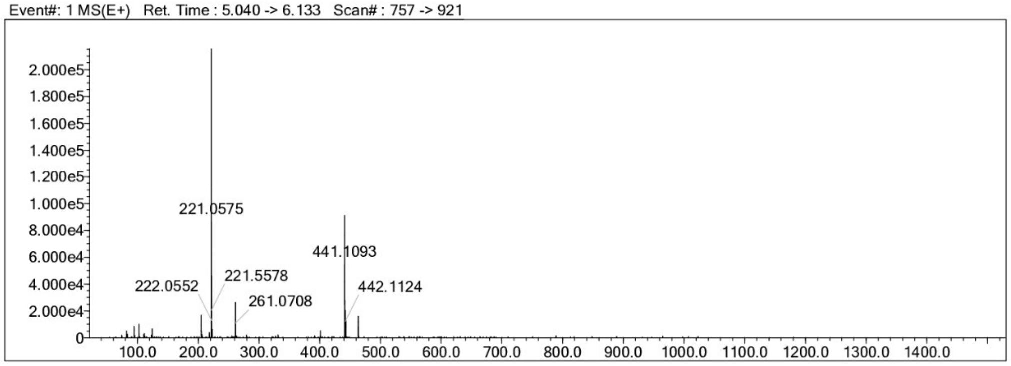

Measured region for 441.1093 m/z

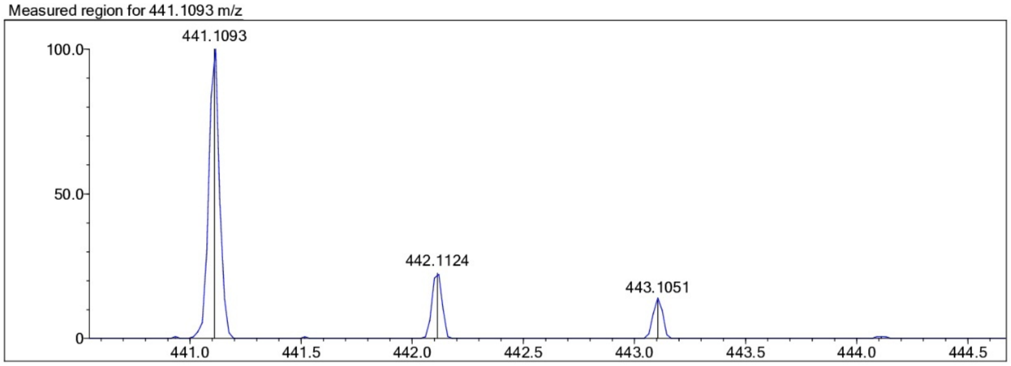

C18 H24 N4 O3 S3 [M+H]+ : Predicted region for 441.1083 m/z

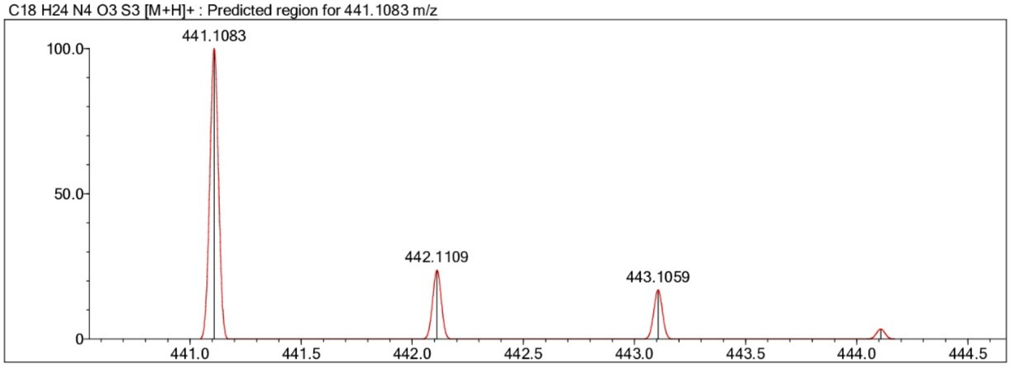

Rank	Score	Formula (M)	Ion	Meas. m/z	Pred. m/z	Df. (mDa)	Df. (ppm)	Iso	DBE
1	81.74	C18 H24 N4 O3 S3	[M+H]+	441.1093	441.1083	1.0	2.27	84.42	9.0

Figure S28. ^{1}H NMR spectrum of compound **10**

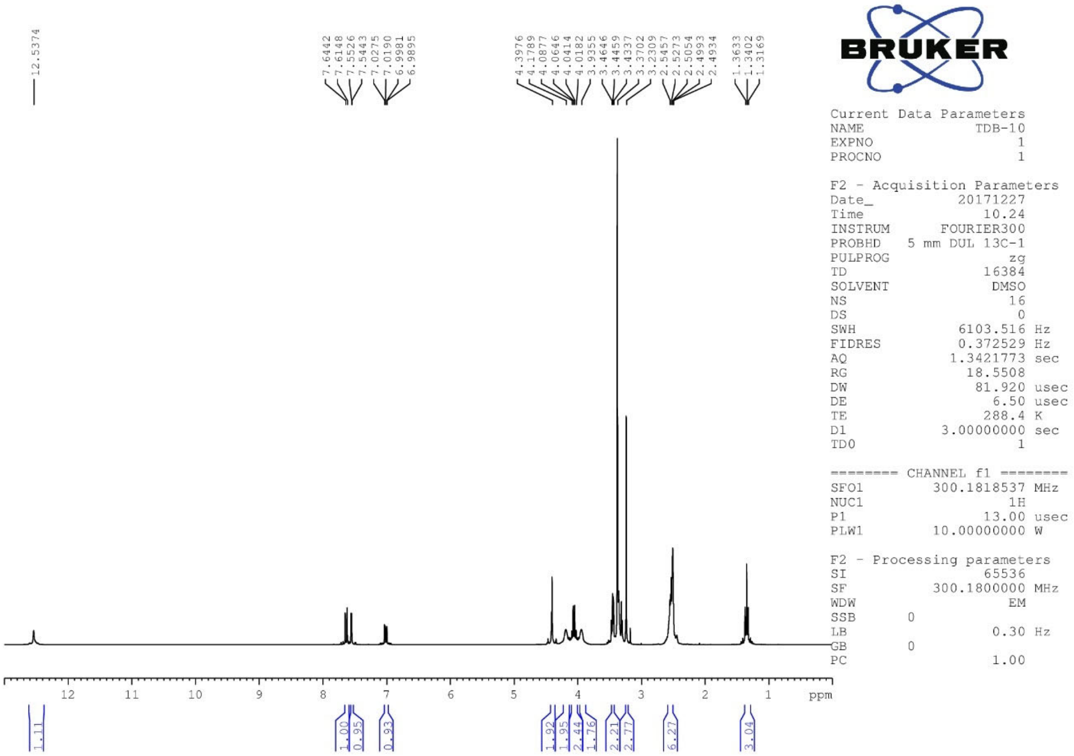

Figure S29. ^{13}C NMR spectrum of compound **10**

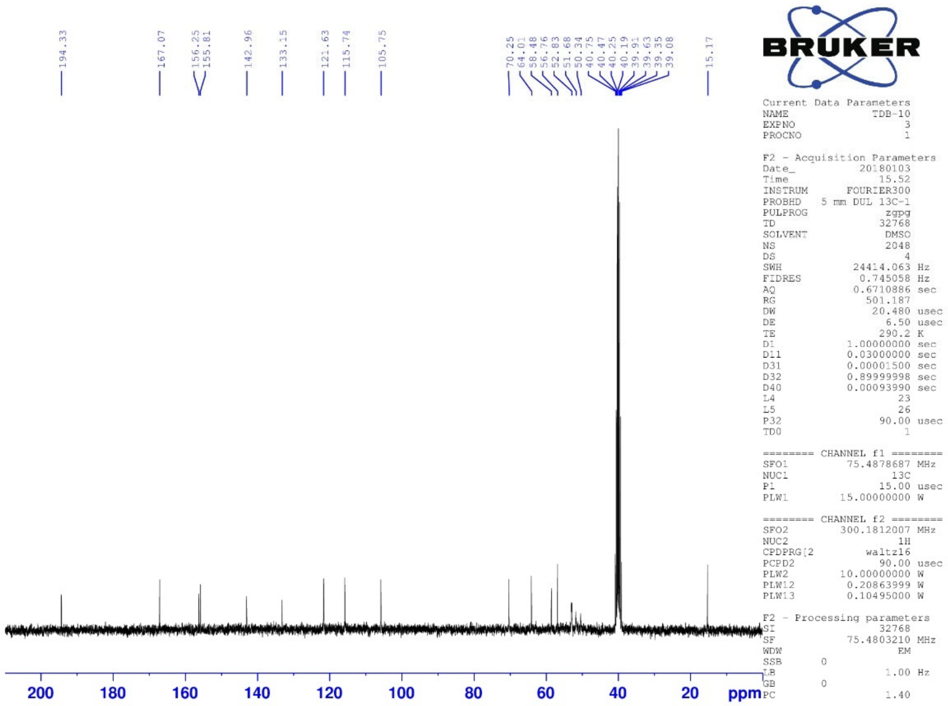

Figure S30. HRMS spectrum of compound **10**

Data File: C:\LabSolutions\Data\Analiz\mdaltintop\TDB-10_28.lcd

Elmt	Val.	Min	Max	Elmt	Val.	Min	Max	Elmt	Val.	Min	Max	Elmt	Val.	Min	Max	Use Adduct
H	1	5	40	O	2	0	4	S	2	0	3	Ru	2	0	0	H
C	4	0	35	F	1	0	0	Cl	1	0	1	I	3	0	0	
N	3	3	5	P	3	0	0	Br	1	0	0					

Error Margin (ppm):	5	DBE Range:	5.0 - 10.0	Electron Ions:	both
HC Ratio:	unlimited	Apply N Rule:	yes	Use MSn Info:	yes
Max Isotopes:	3	Isotope RI (%):	1.00	Isotope Res:	9000
MSn Iso RI (%):	10.00	MSn Logic Mode:	AND	Max Results:	500

Event#: 1 MS(E+) Ret. Time : 5.280 -> 5.920 Scan# : 793 -> 889

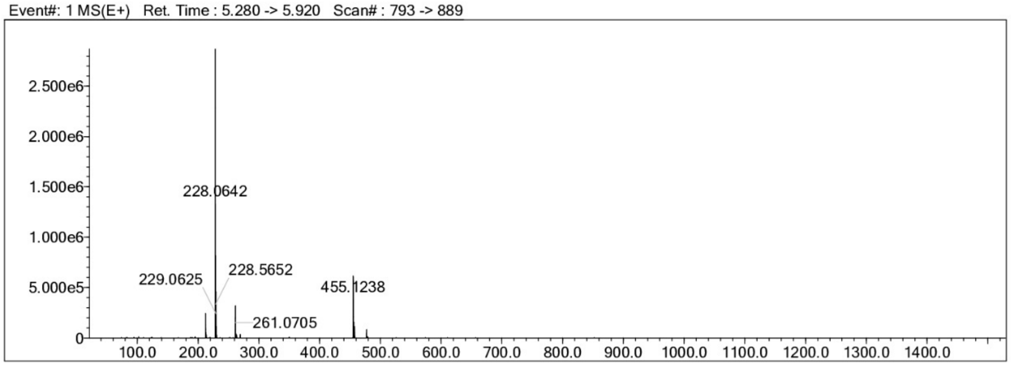

Measured region for 455.1238 m/z

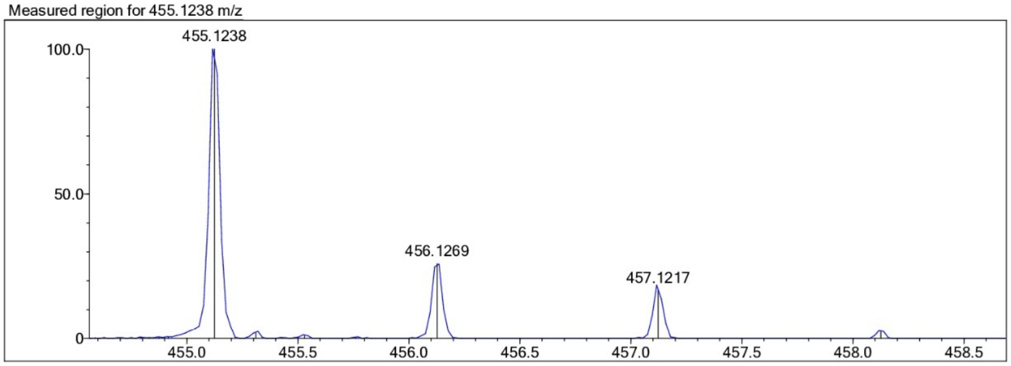

C19 H26 N4 O3 S3 [M+H]+ : Predicted region for 455.1240 m/z

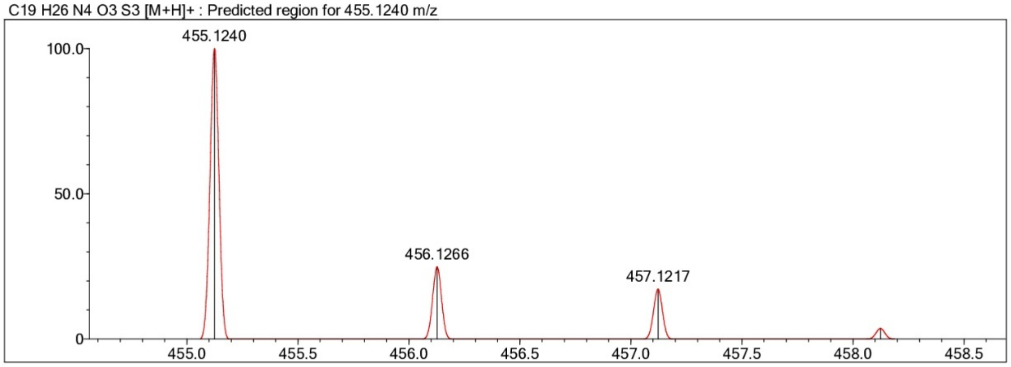

Rank	Score	Formula (M)	Ion	Meas. m/z	Pred. m/z	Df. (mDa)	Df. (ppm)	Iso	DBE
1	100.00	C19 H26 N4 O3 S3	[M+H]+	455.1238	455.1240	-0.2	-0.44	100.00	9.0

Figure S31. ^{1}H NMR spectrum of compound **11**

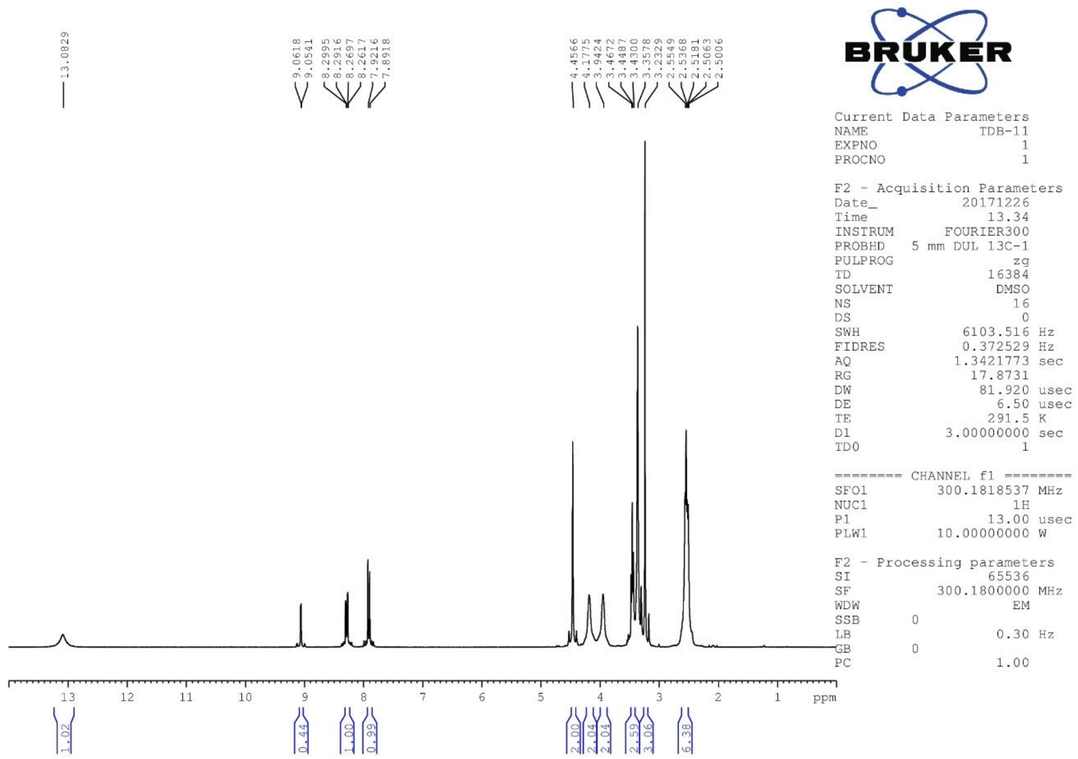

Figure S32. ^{13}C NMR spectrum of compound **11**

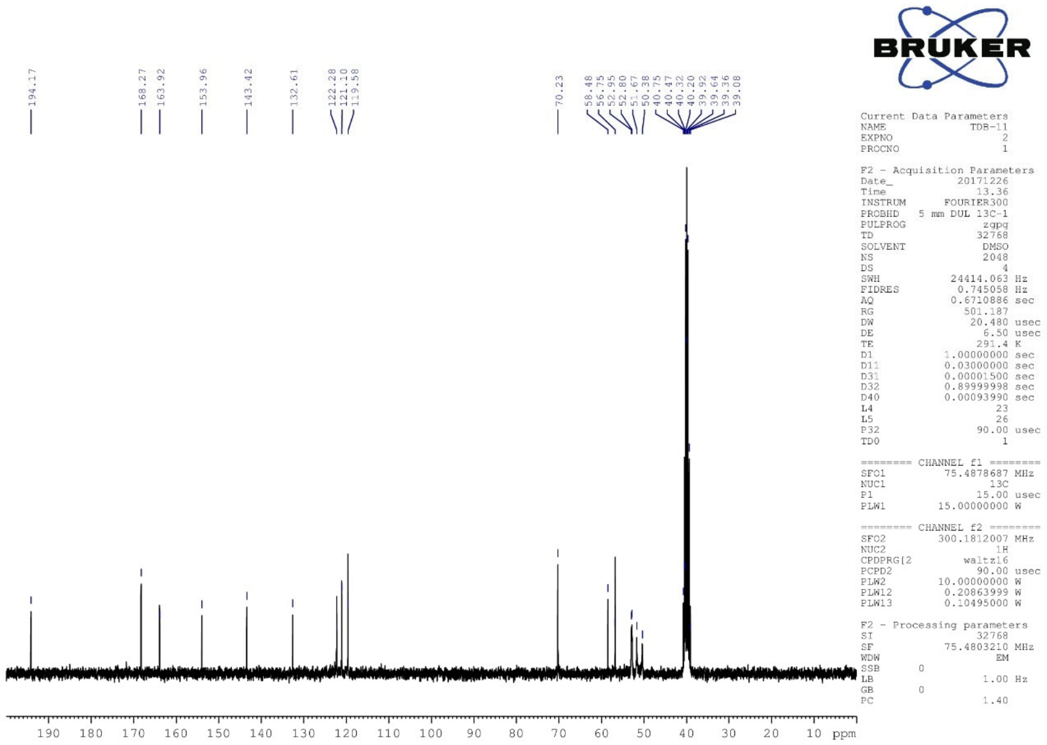

Figure S33. HRMS spectrum of compound **11**

Formula Predictor Report - TDB-11_29.lcd

Data File: C:\LabSolutions\Data\Analiz\mdaltintop\TDB-11_29.lcd

Elmt	Val.	Min	Max	Elmt	Val.	Min	Max	Elmt	Val.	Min	Max	Elmt	Val.	Min	Max	Use Adduct
H	1	5	40	O	2	0	4	S	2	0	3	Ru	2	0	0	H
C	4	0	35	F	1	0	0	Cl	1	0	0	I	3	0	0	
N	3	3	5	P	3	0	0	Br	1	0	0					

Error Margin (ppm): 5
HC Ratio: unlimited
Max Isotopes: 3
MSn Iso RI (%): 10.00

DBE Range: 5.0 - 10.0
Apply N Rule: yes
Isotope RI (%): 1.00
MSn Logic Mode: AND

Electron Ions: both
Use MSn Info: yes
Isotope Res: 9000
Max Results: 500

Event#: 1 MS(E+) Ret. Time : 5.307 -> 5.760 Scan# : 797 -> 865

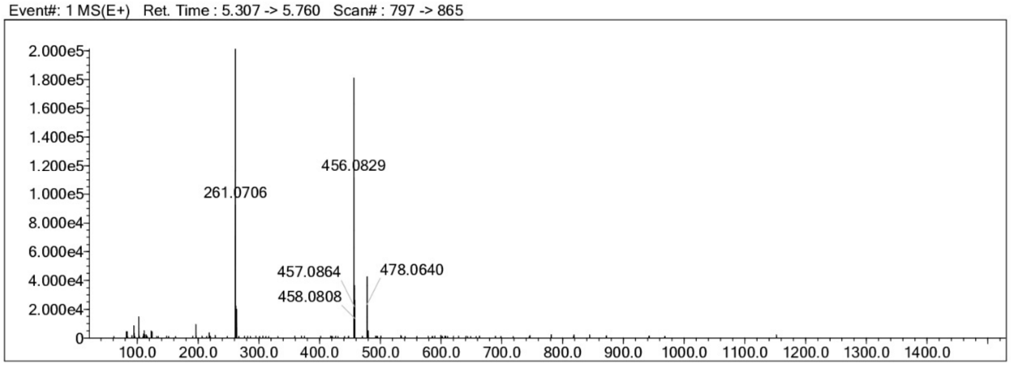

Measured region for 456.0829 m/z

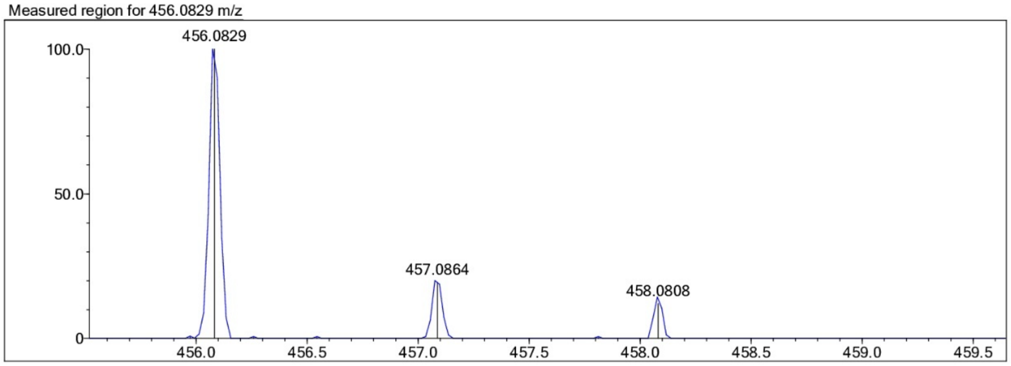

C17 H21 N5 O4 S3 [M+H]+ : Predicted region for 456.0828 m/z

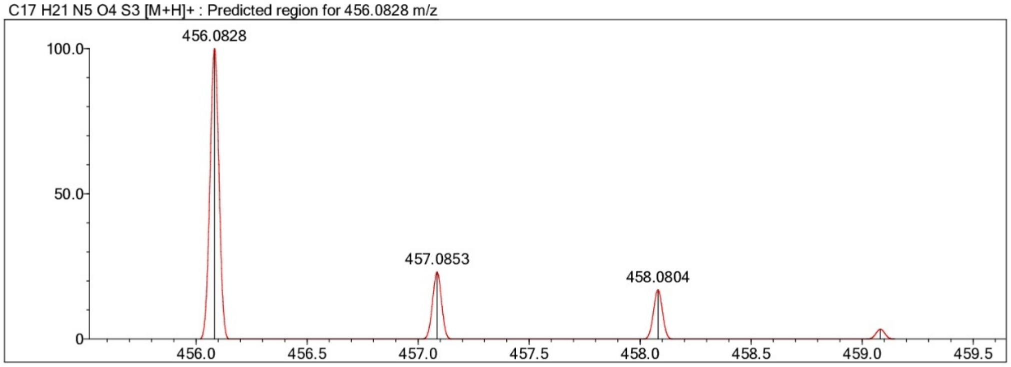

Rank	Score	Formula (M)	Ion	Meas. m/z	Pred. m/z	Df. (mDa)	Df. (ppm)	Iso	DBE
1	82.10	C17 H21 N5 O4 S3	[M+H]+	456.0829	456.0828	0.1	0.22	82.10	10.0

Figure S34. ^{1}H NMR spectrum of compound **12**

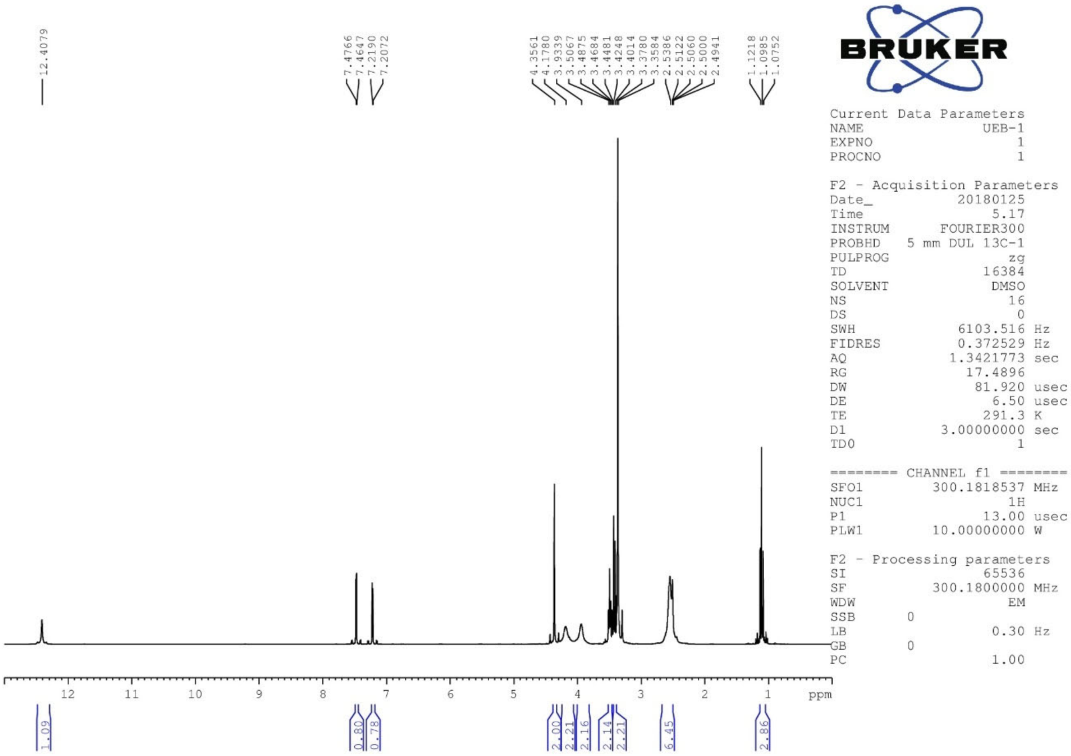

Figure S35. ^{13}C NMR spectrum of compound **12**

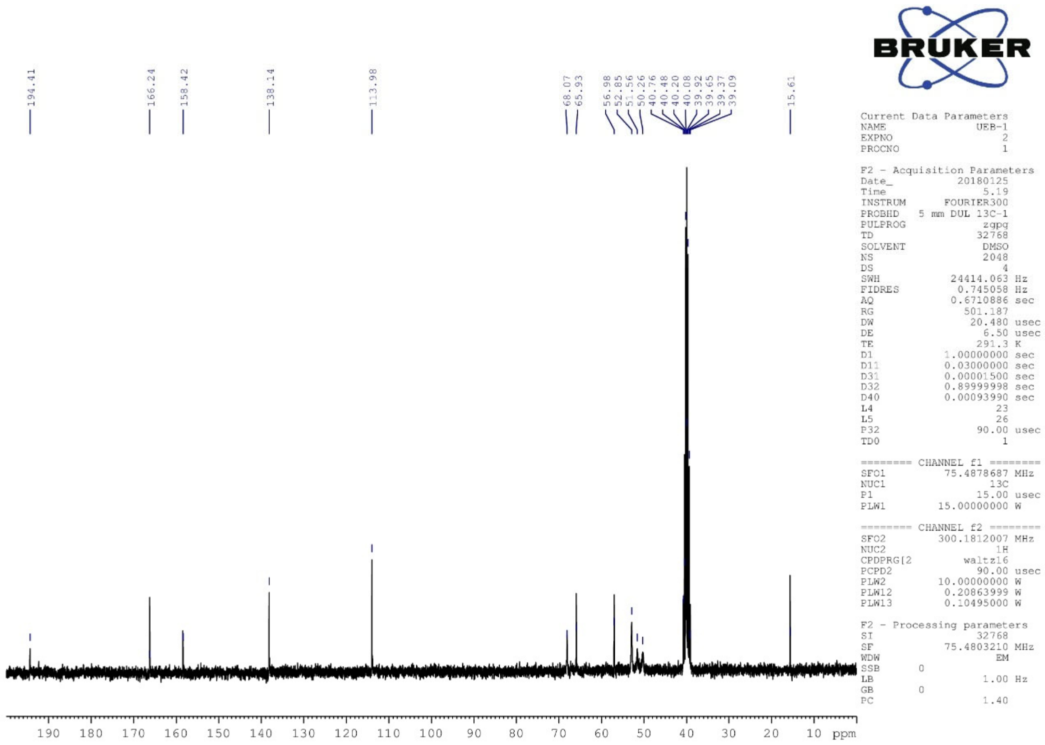

Figure S36. HRMS spectrum of compound **12**

Data File: C:\LabSolutions\Data\Analiz\mdaltintop\UEB-1_15.lcd

Elmt	Val.	Min	Max	Elmt	Val.	Min	Max	Elmt	Val.	Min	Max	Elmt	Val.	Min	Max	Use Adduct
H	1	5	40	O	2	0	4	S	2	0	3	Ru	2	0	0	H
C	4	0	35	F	1	0	0	Cl	1	0	0	I	3	0	0	
N	3	3	4	P	3	0	0	Br	1	0	0					

Error Margin (ppm): 5
HC Ratio: unlimited
Max Isotopes: 3
MSn Iso RI (%): 10.00

DBE Range: 5.0 - 10.0
Apply N Rule: yes
Isotope RI (%): 1.00
MSn Logic Mode: AND

Electron Ions: both
Use MSn Info: yes
Isotope Res: 9000
Max Results: 500

Event#: 1 MS(E+) Ret. Time : 4.293 -> 5.080 Scan# : 645 -> 763

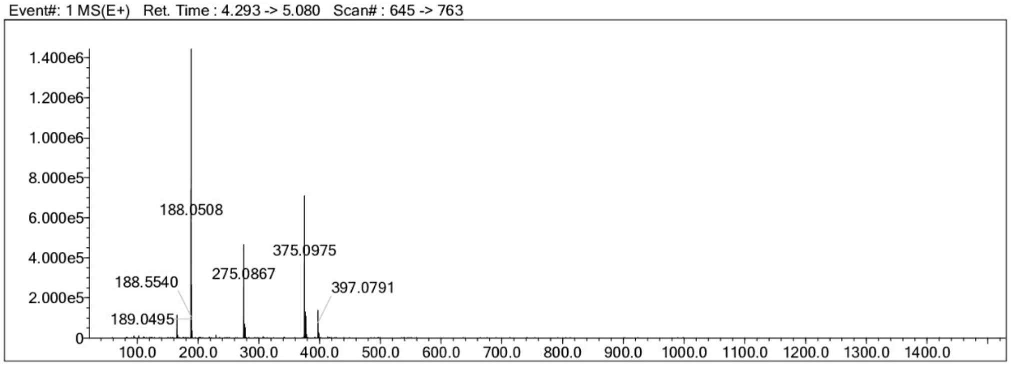

Measured region for 375.0975 m/z

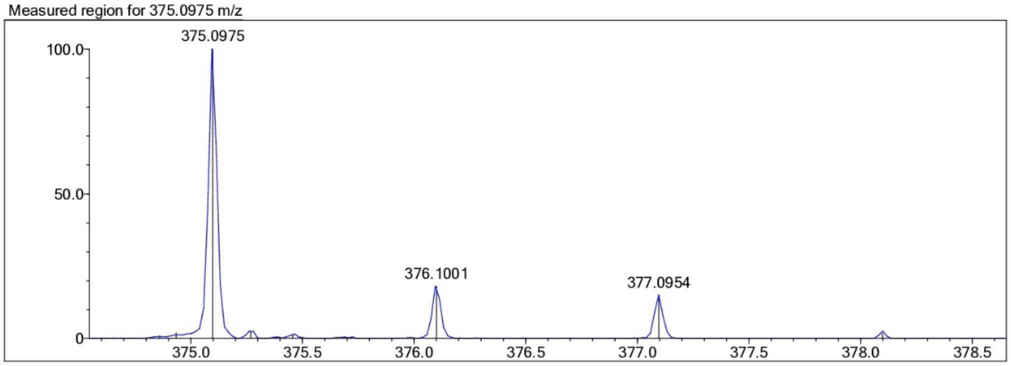

C14 H22 N4 O2 S3 [M+H]+ : Predicted region for 375.0978 m/z

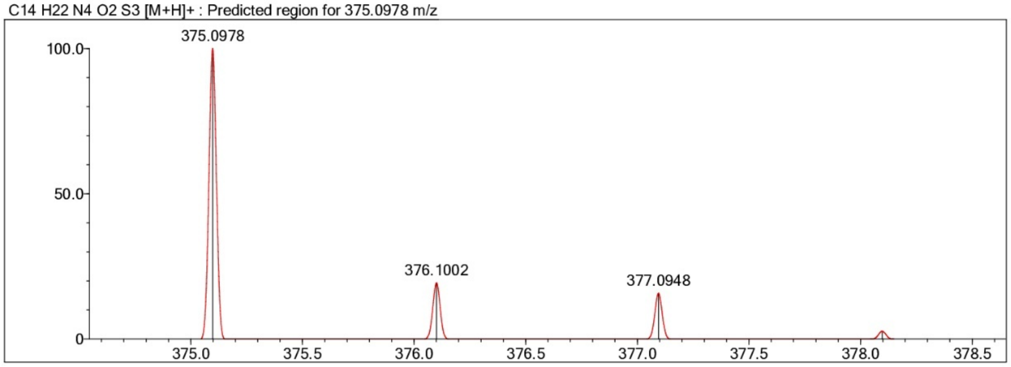

Rank	Score	Formula (M)	Ion	Meas. m/z	Pred. m/z	Df. (mDa)	Df. (ppm)	Iso	DBE
1	89.57	C14 H22 N4 O2 S3	[M+H]+	375.0975	375.0978	-0.3	-0.80	89.57	6.0

Figure S37. ^{1}H NMR spectrum of compound **13**

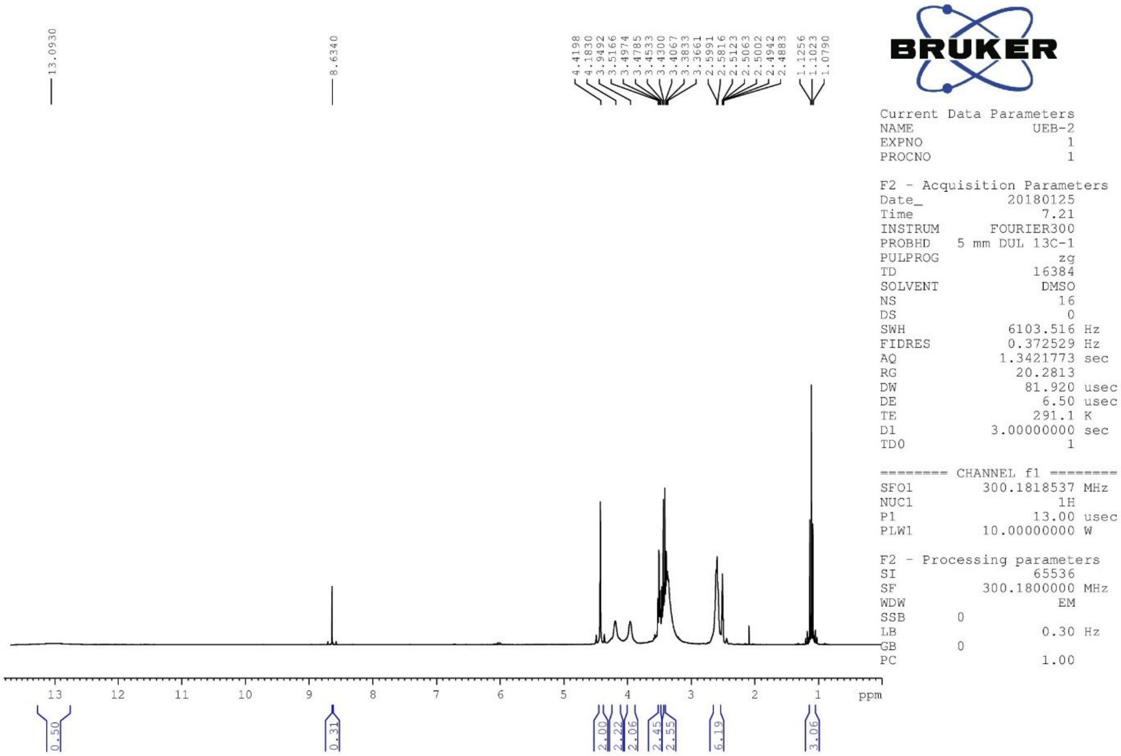

Figure S38. ^{13}C NMR spectrum of compound **13**

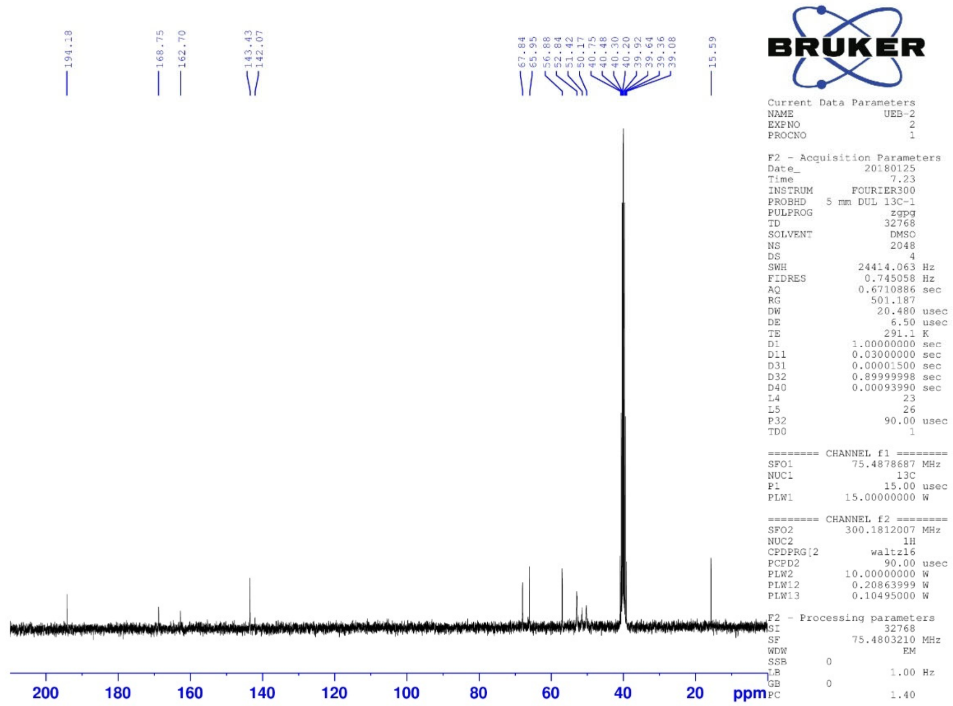

Figure S39. HRMS spectrum of compound **13**

Formula Predictor Report - UEB-2_16.lcd Page 1 of 1

Data File: C:\LabSolutions\Data\Analiz\mdaltintop\UEB-2_16.lcd

Elmt	Val.	Min	Max	Elmt	Val.	Min	Max	Elmt	Val.	Min	Max	Elmt	Val.	Min	Max	Use Adduct
H	1	5	40	O	2	0	5	S	2	0	3	Ru	2	0	0	H
C	4	0	35	F	1	0	0	Cl	1	0	0	I	3	0	0	
N	3	3	6	P	3	0	0	Br	1	0	0					

Error Margin (ppm):	5	DBE Range:	5.0 - 15.0	Electron Ions:	both
HC Ratio:	unlimited	Apply N Rule:	yes	Use MSn Info:	yes
Max Isotopes:	3	Isotope RI (%):	1.00	Isotope Res:	9000
MSn Iso RI (%):	10.00	MSn Logic Mode:	AND	Max Results:	500

Event#: 1 MS(E+) Ret. Time : 3.827 -> 5.627 Scan# : 575 -> 845

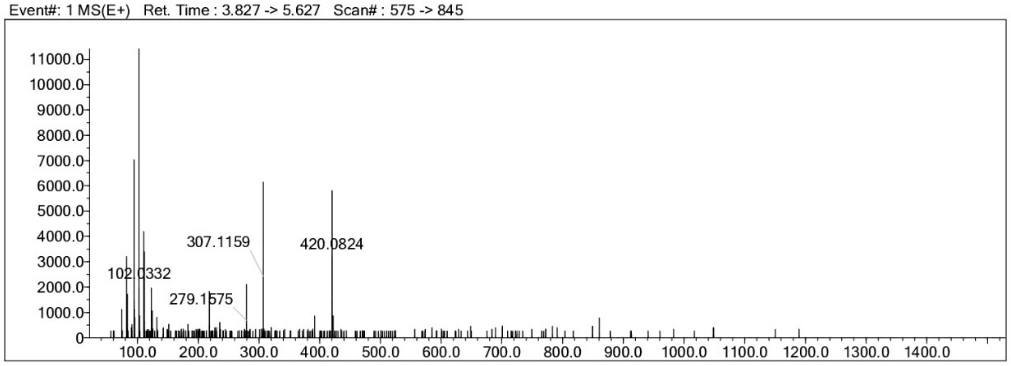

Measured region for 420.0824 m/z

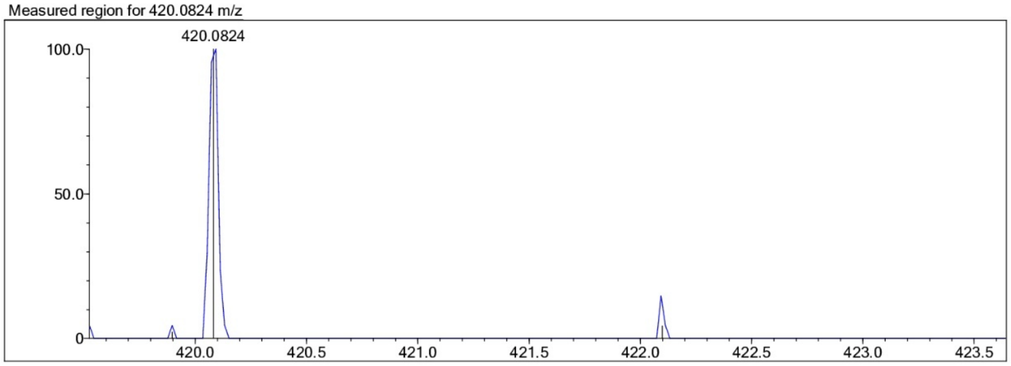

C14 H21 N5 O4 S3 [M+H]+ : Predicted region for 420.0828 m/z

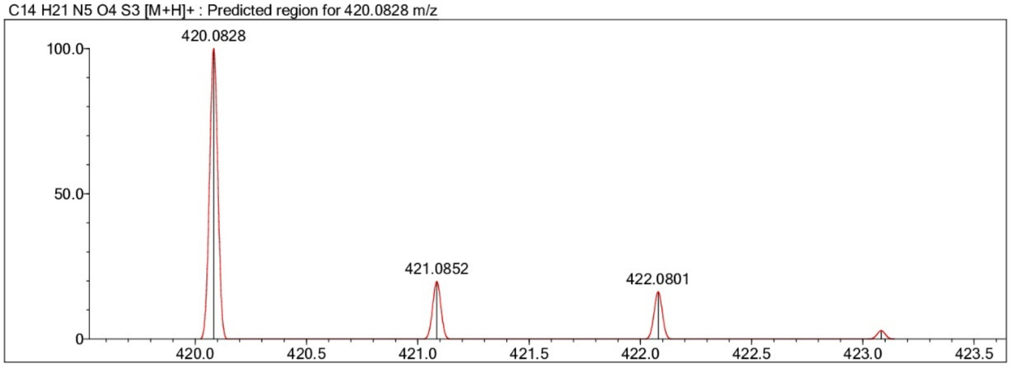

Rank	Score	Formula (M)	Ion	Meas. m/z	Pred. m/z	Df. (mDa)	Df. (ppm)	Iso	DBE
1	27.88	C14 H21 N5 O4 S3	[M+H]+	420.0824	420.0828	-0.4	-0.95	27.88	7.0

Figure S40. ^{1}H NMR spectrum of compound **14**

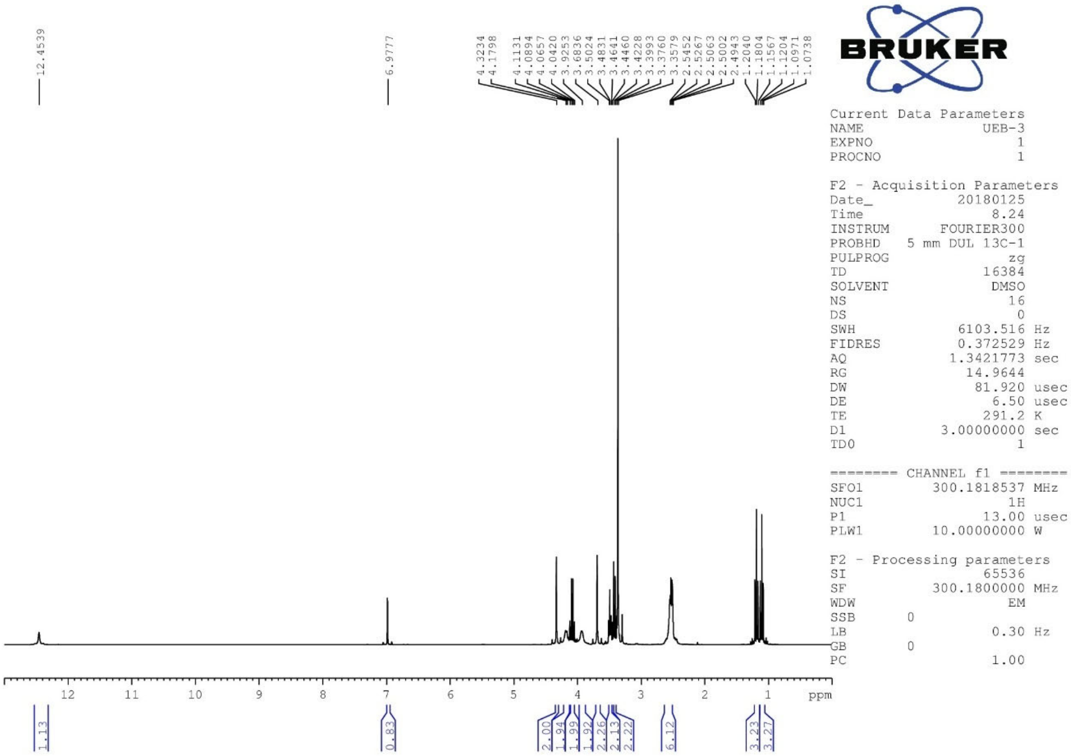

Figure S41. ^{13}C NMR spectrum of compound **14**

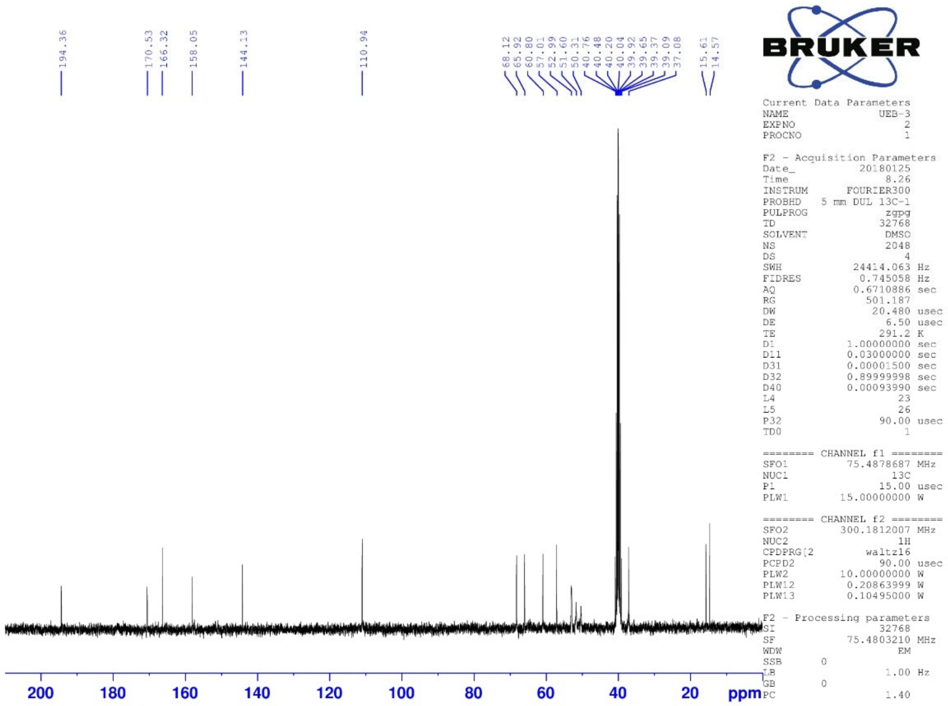

Figure S42. HRMS spectrum of compound **14**

Formula Predictor Report - UEB-3_16.lcd

Page 1 of 1

Data File: C:\LabSolutions\Data\Analiz\mdaltintop\UEB-3_16.lcd

Elmt	Val.	Min	Max	Elmt	Val.	Min	Max	Elmt	Val.	Min	Max	Elmt	Val.	Min	Max	Use Adduct
H	1	5	40	O	2	0	4	S	2	0	3	Ru	2	0	0	H
C	4	0	35	F	1	0	0	Cl	1	0	0	I	3	0	0	
N	3	3	4	P	3	0	0	Br	1	0	0					

Error Margin (ppm): 5
HC Ratio: unlimited
Max Isotopes: 3
MSn Iso RI (%): 10.00

DBE Range: 5.0 - 10.0
Apply N Rule: yes
Isotope RI (%): 1.00
MSn Logic Mode: AND

Electron Ions: both
Use MSn Info: yes
Isotope Res: 9000
Max Results: 500

Event#: 1 MS(E+) Ret. Time : 4.880 -> 5.587 Scan# : 733 -> 839

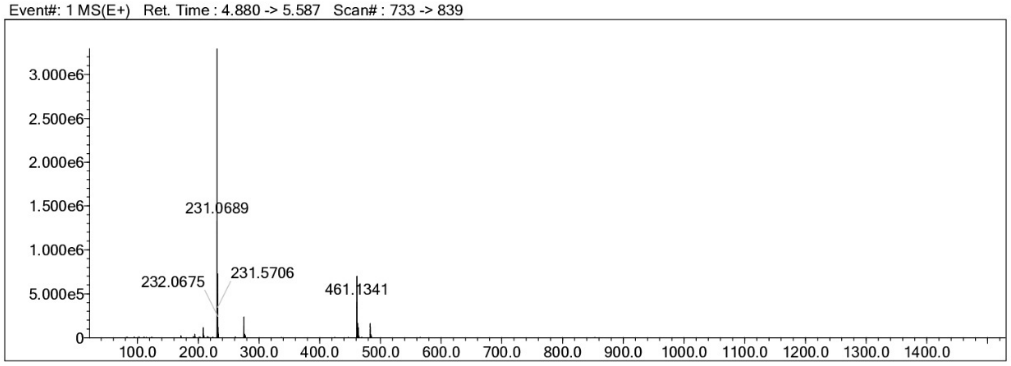

Measured region for 461.1341 m/z

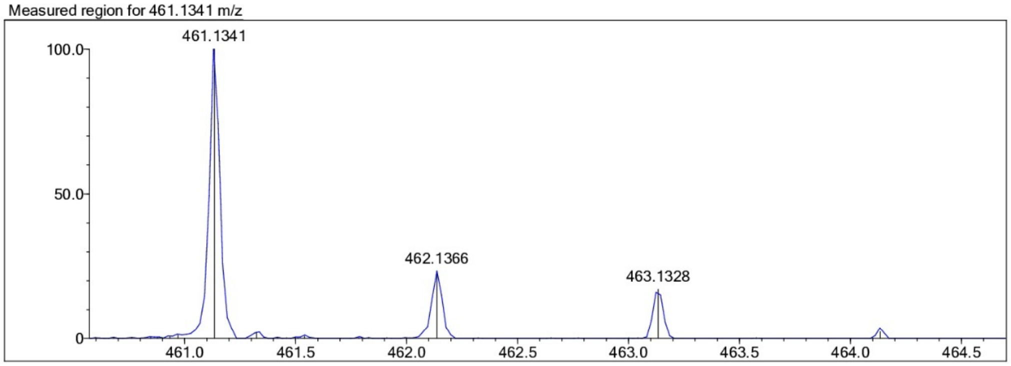

C18 H28 N4 O4 S3 [M+H]+ : Predicted region for 461.1345 m/z

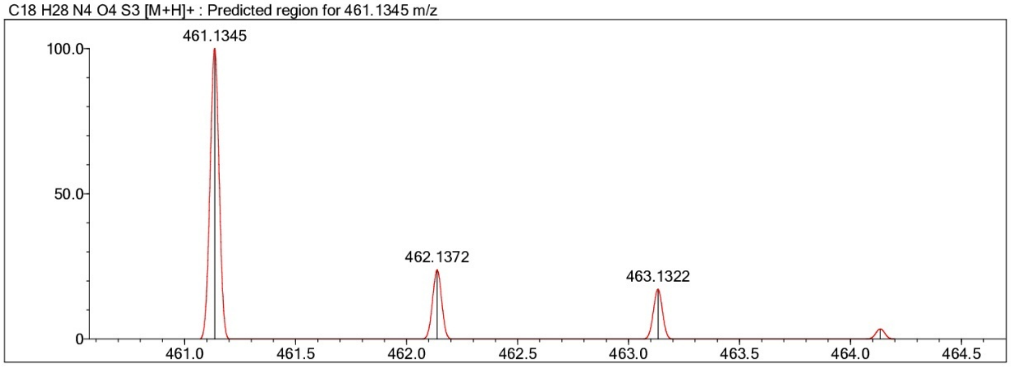

Rank	Score	Formula (M)	Ion	Meas. m/z	Pred. m/z	Df. (mDa)	Df. (ppm)	Iso	DBE
1	93.41	C18 H28 N4 O4 S3	[M+H]+	461.1341	461.1345	-0.4	-0.87	93.41	7.0

Figure S43. ^{1}H NMR spectrum of compound **15**

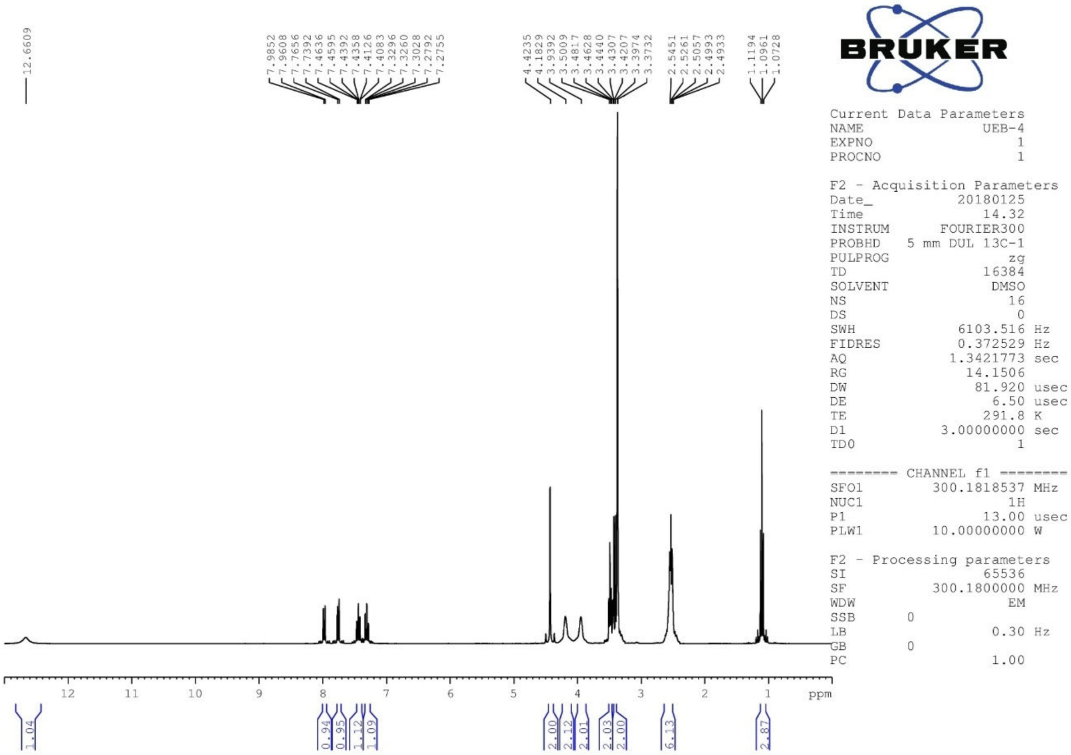

Figure S44. ^{13}C NMR spectrum of compound **15**

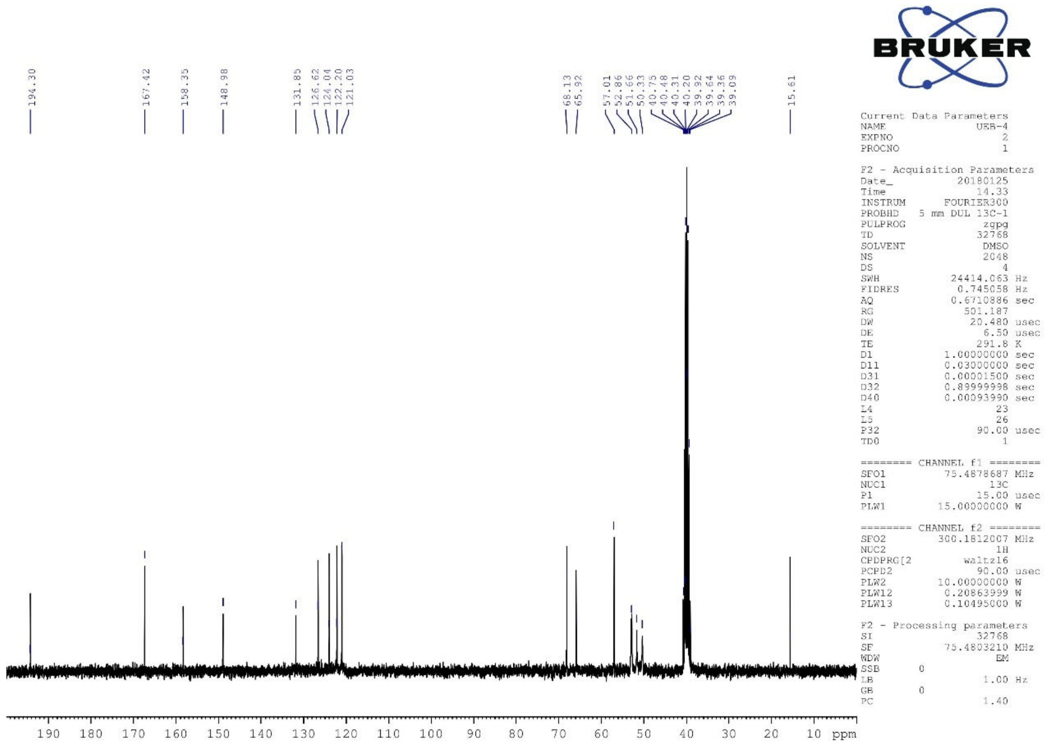

Figure S45. HRMS spectrum of compound **15**

Formula Predictor Report - UEB-4_17.lcd Page 1 of 1

Data File: C:\LabSolutions\Data\Analiz\mdaltintop\UEB-4_17.lcd

Elmt	Val.	Min	Max	Elmt	Val.	Min	Max	Elmt	Val.	Min	Max	Elmt	Val.	Min	Max	Use Adduct
H	1	5	40	O	2	0	4	S	2	0	3	Ru	2	0	0	H
C	4	0	35	F	1	0	0	Cl	1	0	0	I	3	0	0	
N	3	3	4	P	3	0	0	Br	1	0	0					

Error Margin (ppm):	5	DBE Range:	5.0 - 10.0	Electron Ions:	both
HC Ratio:	unlimited	Apply N Rule:	yes	Use MSn Info:	yes
Max Isotopes:	3	Isotope RI (%):	1.00	Isotope Res:	9000
MSn Iso RI (%):	10.00	MSn Logic Mode:	AND	Max Results:	500

Event#: 1 MS(E+) Ret. Time : 5.173 -> 5.787 Scan# : 777 -> 869

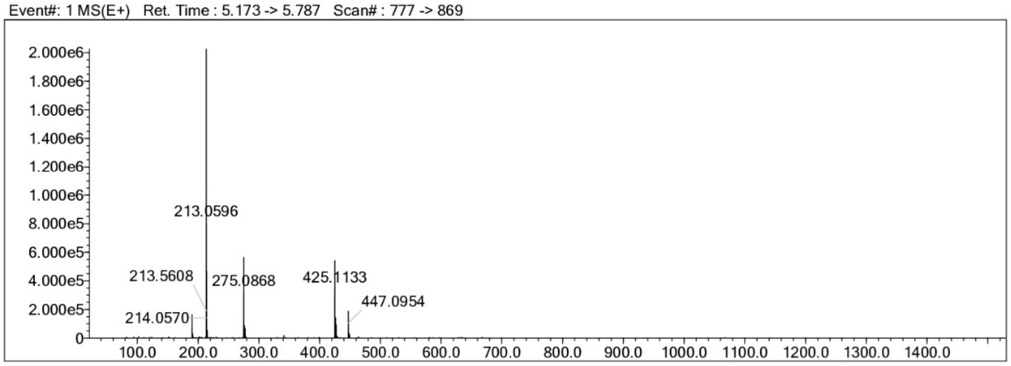

Measured region for 425.1133 m/z

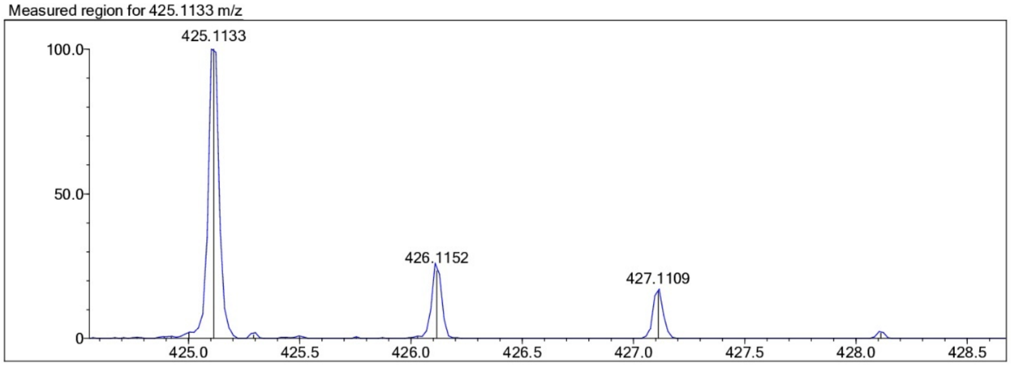

C18 H24 N4 O2 S3 [M+H]+ : Predicted region for 425.1134 m/z

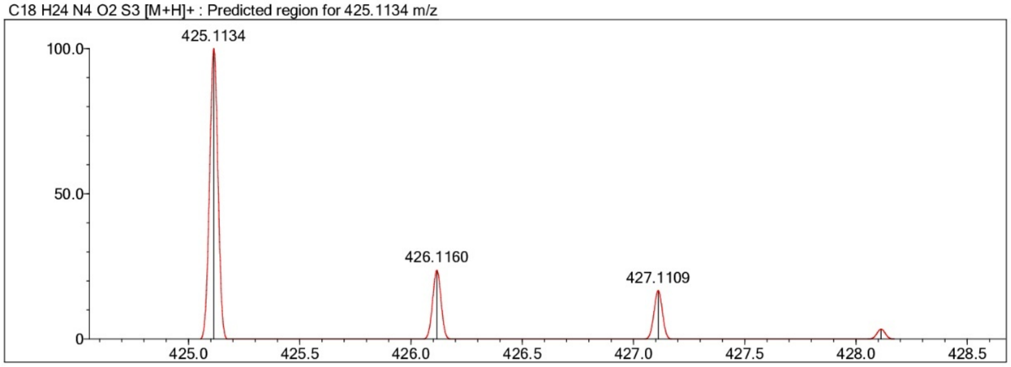

Rank	Score	Formula (M)	Ion	Meas. m/z	Pred. m/z	Df. (mDa)	Df. (ppm)	Iso	DBE
1	100.00	C18 H24 N4 O2 S3	[M+H]+	425.1133	425.1134	-0.1	-0.24	100.00	9.0

Figure S46. ^{1}H NMR spectrum of compound **16**

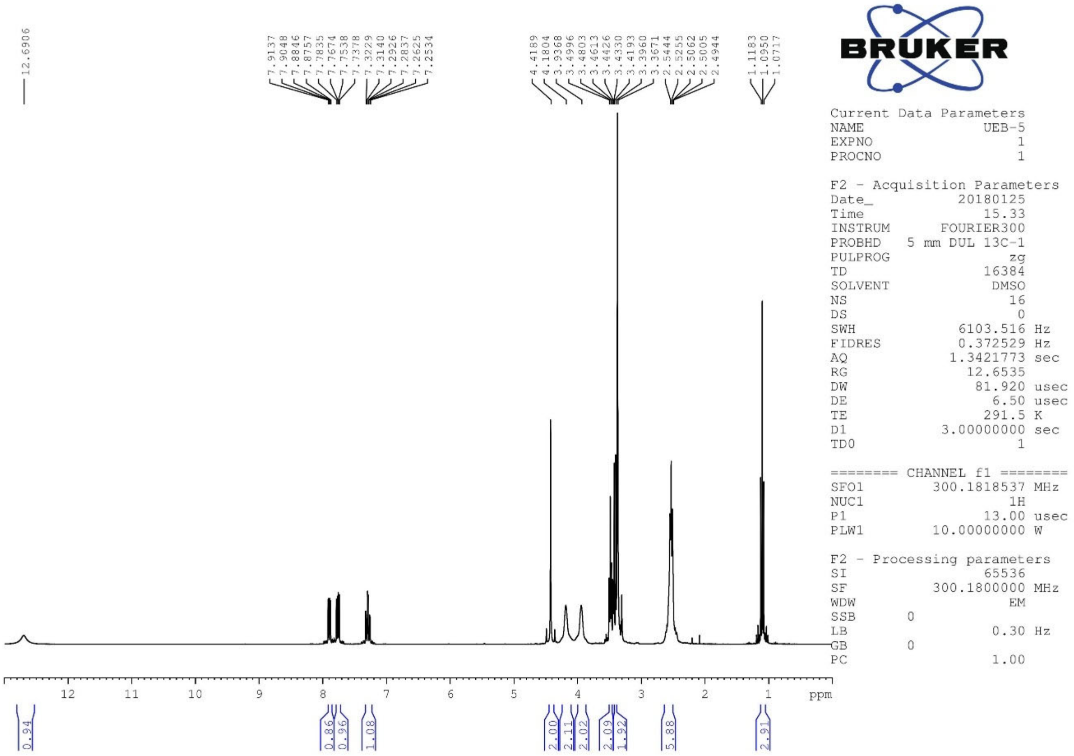

Figure S47. ^{13}C NMR spectrum of compound **16**

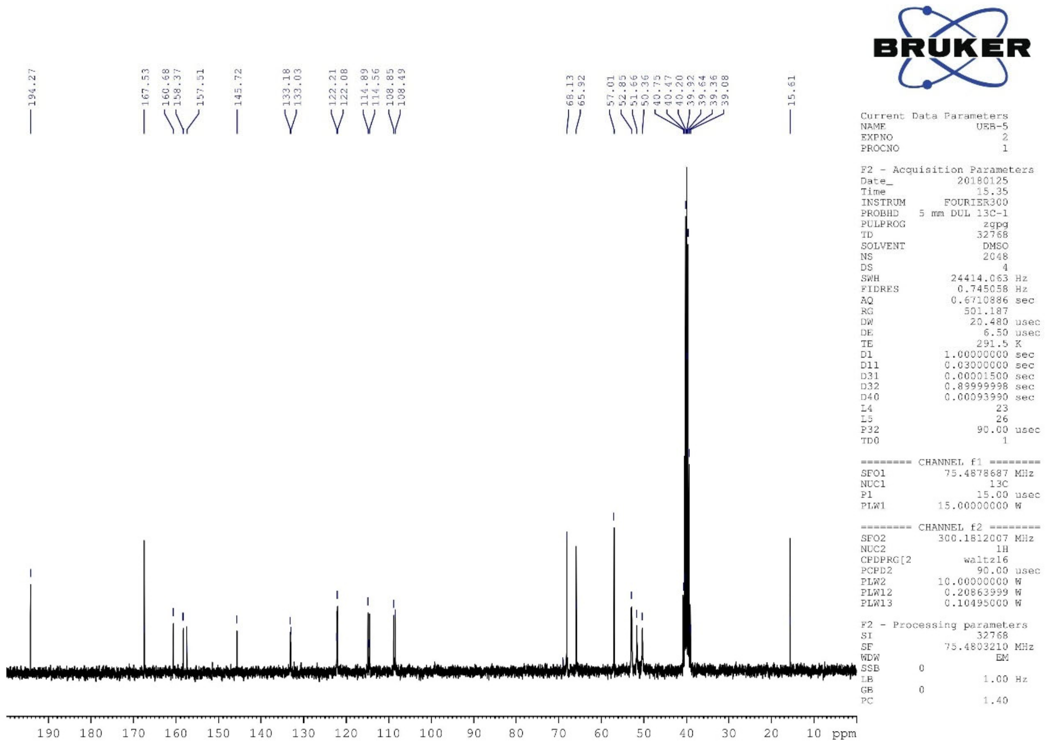

Figure S48. HRMS spectrum of compound **16**

Formula Predictor Report - UEB-5_17.lcd

Page 1 of 1

Data File: C:\LabSolutions\Data\Analiz\mdaltintop\UEB-5_17.lcd

Elmt	Val.	Min	Max	Elmt	Val.	Min	Max	Elmt	Val.	Min	Max	Elmt	Val.	Min	Max	Use Adduct
H	1	5	40	O	2	0	5	S	2	0	3	Ru	2	0	0	H
C	4	0	35	F	1	0	1	Cl	1	0	0	I	3	0	0	
N	3	3	6	P	3	0	0	Br	1	0	0					

Error Margin (ppm): 5
HC Ratio: unlimited
Max Isotopes: 3
MSn Iso RI (%): 10.00

DBE Range: 5.0 - 15.0
Apply N Rule: yes
Isotope RI (%): 1.00
MSn Logic Mode: AND

Electron Ions: both
Use MSn Info: yes
Isotope Res: 9000
Max Results: 500

Event#: 1 MS(E+) Ret. Time : 5.320 -> 6.000 Scan# : 799 -> 901

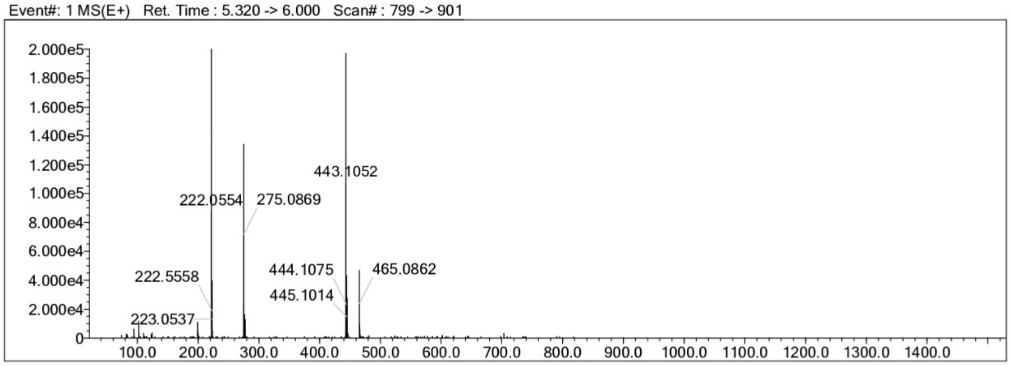

Measured region for 443.1052 m/z

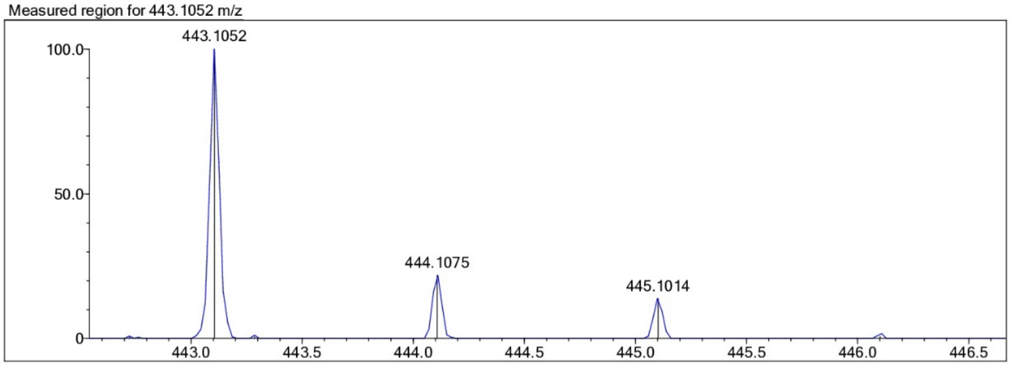

C18 H23 N4 O2 F S3 [M+H]+ : Predicted region for 443.1040 m/z

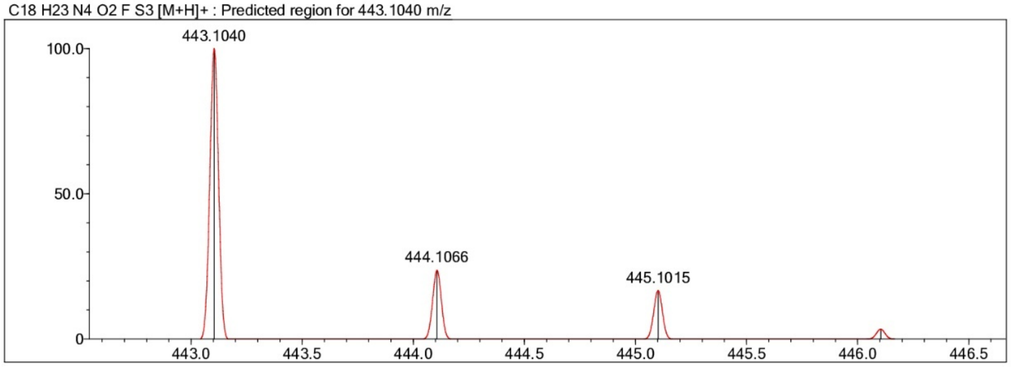

Rank	Score	Formula (M)	Ion	Meas. m/z	Pred. m/z	Df. (mDa)	Df. (ppm)	Iso	DBE
1	87.53	C18 H23 N4 O2 F S3	[M+H]+	443.1052	443.1040	1.2	2.71	91.43	9.0

Figure S49. ^{1}H NMR spectrum of compound **17**

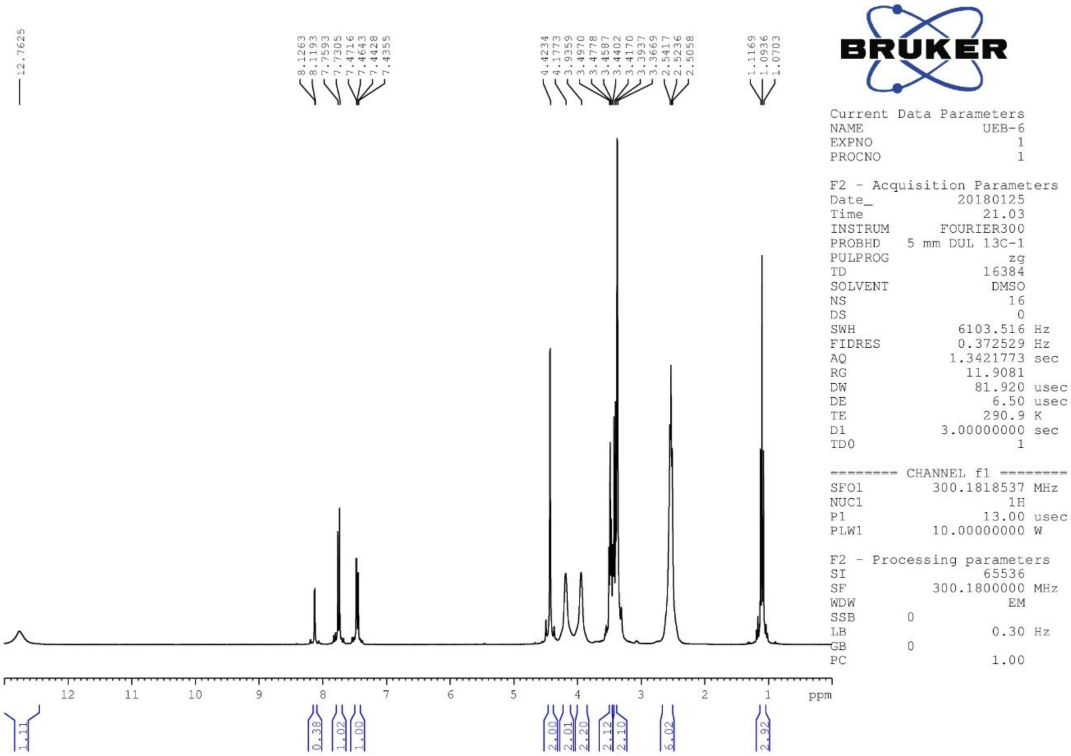

Figure S50. ^{13}C NMR spectrum of compound **17**

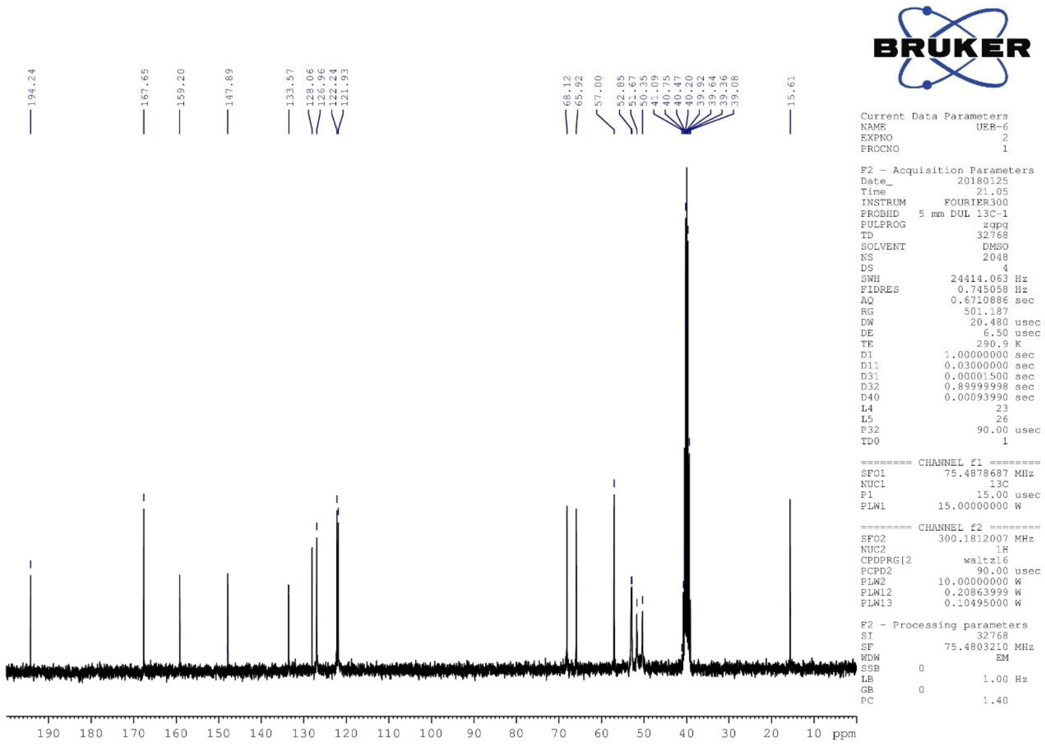

Figure S51. HRMS spectrum of compound **17**

Formula Predictor Report - UEB-6_18.lcd Page 1 of 1

Data File: C:\LabSolutions\Data\Analiz\mdaltintop\UEB-6_18.lcd

Elmt	Val.	Min	Max	Elmt	Val.	Min	Max	Elmt	Val.	Min	Max	Elmt	Val.	Min	Max	Use Adduct
H	1	5	40	O	2	0	4	S	2	0	3	Ru	2	0	0	H
C	4	0	35	F	1	0	0	Cl	1	0	1	I	3	0	0	
N	3	3	4	P	3	0	0	Br	1	0	0					

Error Margin (ppm):	5	DBE Range:	5.0 - 10.0	Electron Ions:	both
HC Ratio:	unlimited	Apply N Rule:	yes	Use MSn Info:	yes
Max Isotopes:	3	Isotope RI (%):	1.00	Isotope Res:	9000
MSn Iso RI (%):	10.00	MSn Logic Mode:	AND	Max Results:	500

Event#: 1 MS(E+) Ret. Time : 5.547 -> 6.333 Scan# : 833 -> 951

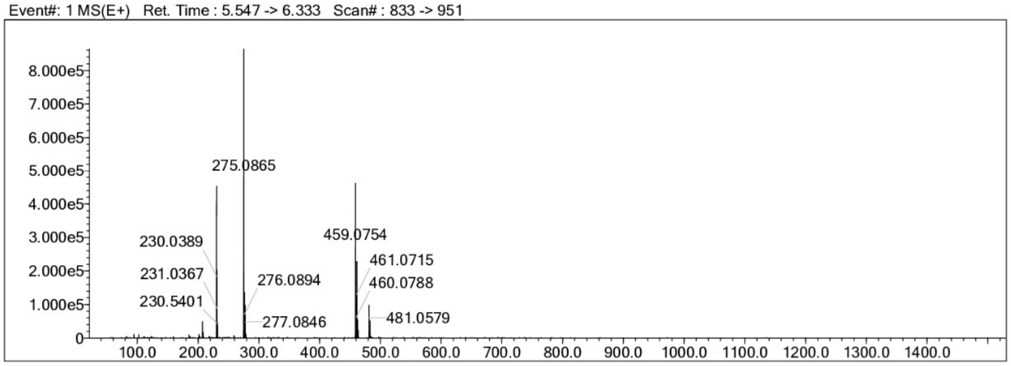

Measured region for 459.0754 m/z

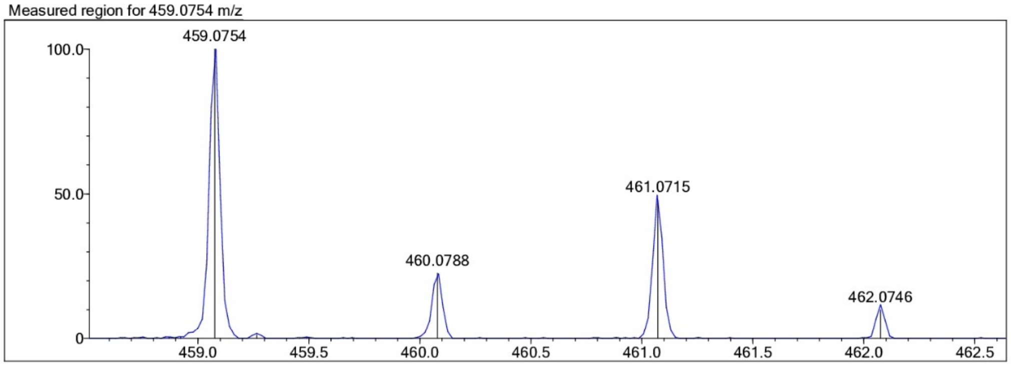

C18 H23 N4 O2 S3 Cl [M+H]+ : Predicted region for 459.0744 m/z

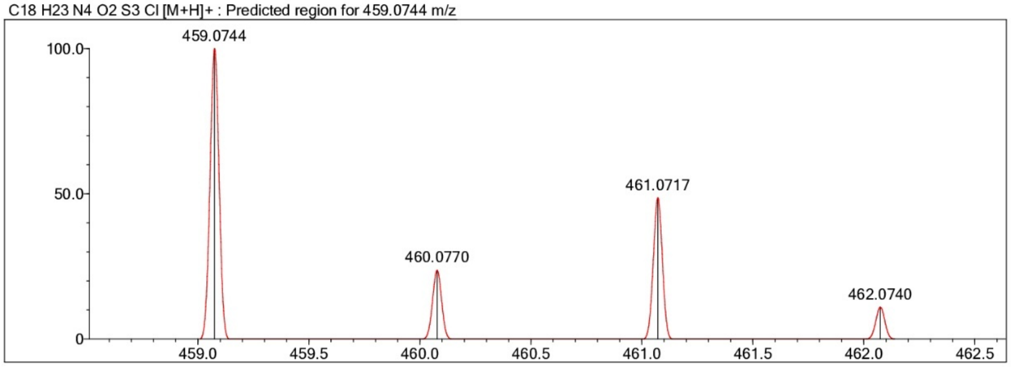

Rank	Score	Formula (M)	Ion	Meas. m/z	Pred. m/z	Df. (mDa)	Df. (ppm)	Iso	DBE
1	88.81	C18 H23 N4 O2 S3 Cl	[M+H]+	459.0754	459.0744	1.0	2.18	91.51	9.0

Figure S52. ^{1}H NMR spectrum of compound **18**

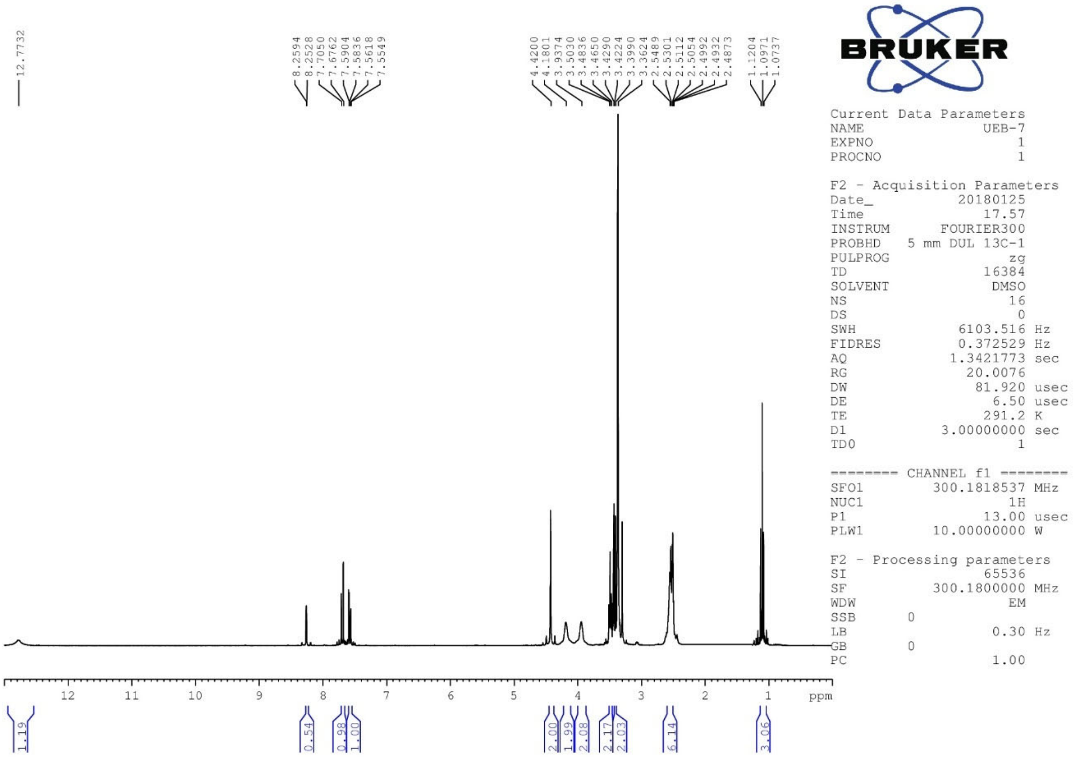

Figure S53. ^{13}C NMR spectrum of compound **18**

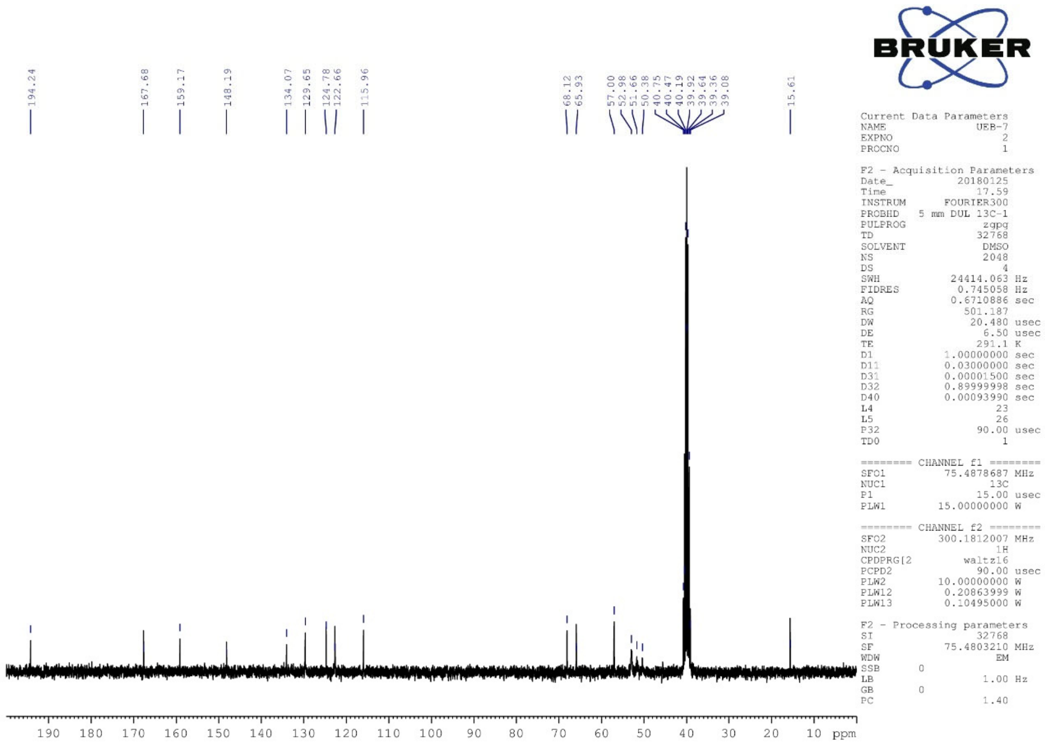

Figure S54. HRMS spectrum of compound **18**

Formula Predictor Report - UEB-7_18.lcd

Page 1 of 1

Data File: C:\LabSolutions\Data\Analiz\mdaltintop\UEB-7_18.lcd

Elmt	Val.	Min	Max	Elmt	Val.	Min	Max	Elmt	Val.	Min	Max	Elmt	Val.	Min	Max	Use Adduct
H	1	5	40	O	2	0	5	S	2	0	3	Ru	2	0	0	H
C	4	0	35	F	1	0	0	Cl	1	0	0	I	3	0	0	
N	3	3	6	P	3	0	0	Br	1	0	1					

Error Margin (ppm):	5	DBE Range:	5.0 - 15.0	Electron Ions:	both
HC Ratio:	unlimited	Apply N Rule:	yes	Use MSn Info:	yes
Max Isotopes:	3	Isotope RI (%):	1.00	Isotope Res:	9000
MSn Iso RI (%):	10.00	MSn Logic Mode:	AND	Max Results:	500

Event#: 1 MS(E+) Ret. Time : 6.133 Scan# : 921

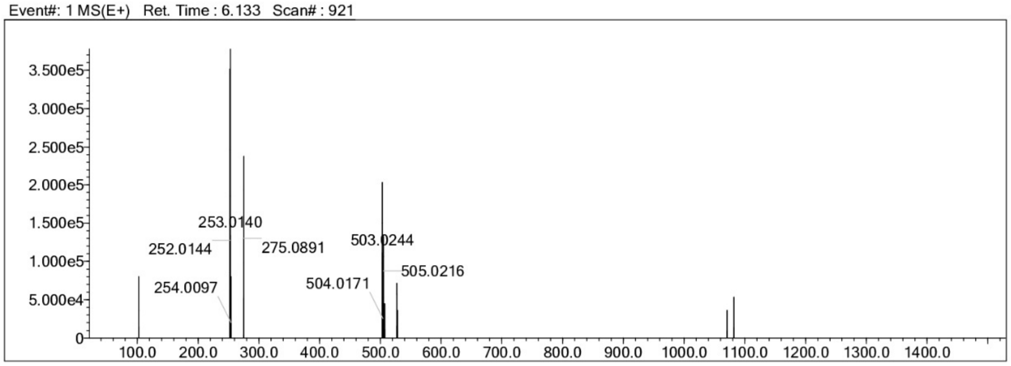

Measured region for 503.0244 m/z

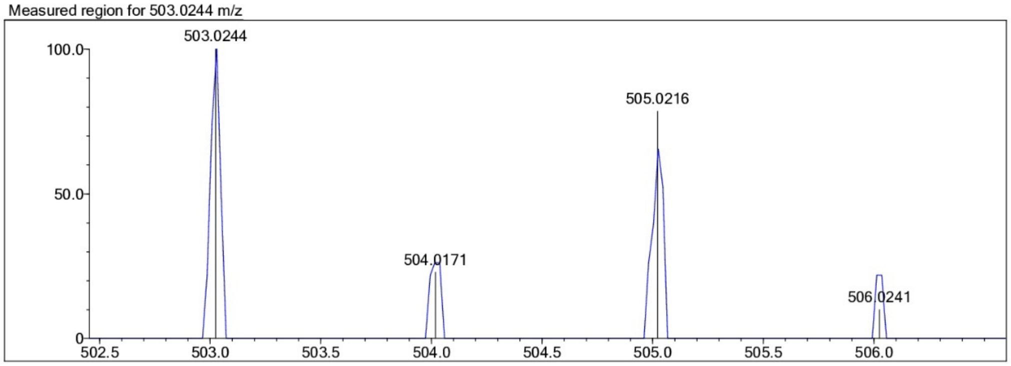

C18 H23 N4 O2 S3 Br [M+H]+ : Predicted region for 503.0239 m/z

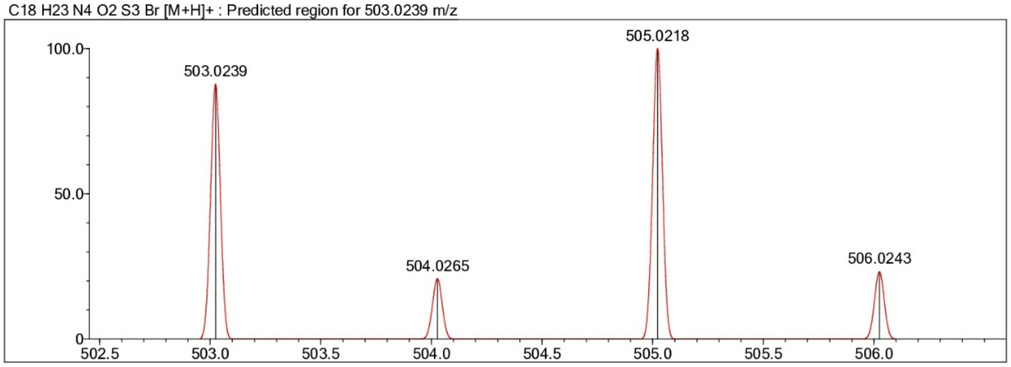

Rank	Score	Formula (M)	Ion	Meas. m/z	Pred. m/z	Df. (mDa)	Df. (ppm)	Iso	DBE
1	36.43	C18 H23 N4 O2 S3 Br	[M+H]+	503.0244	503.0239	0.5	0.99	36.43	9.0

Figure S55. ^{1}H NMR spectrum of compound **19**

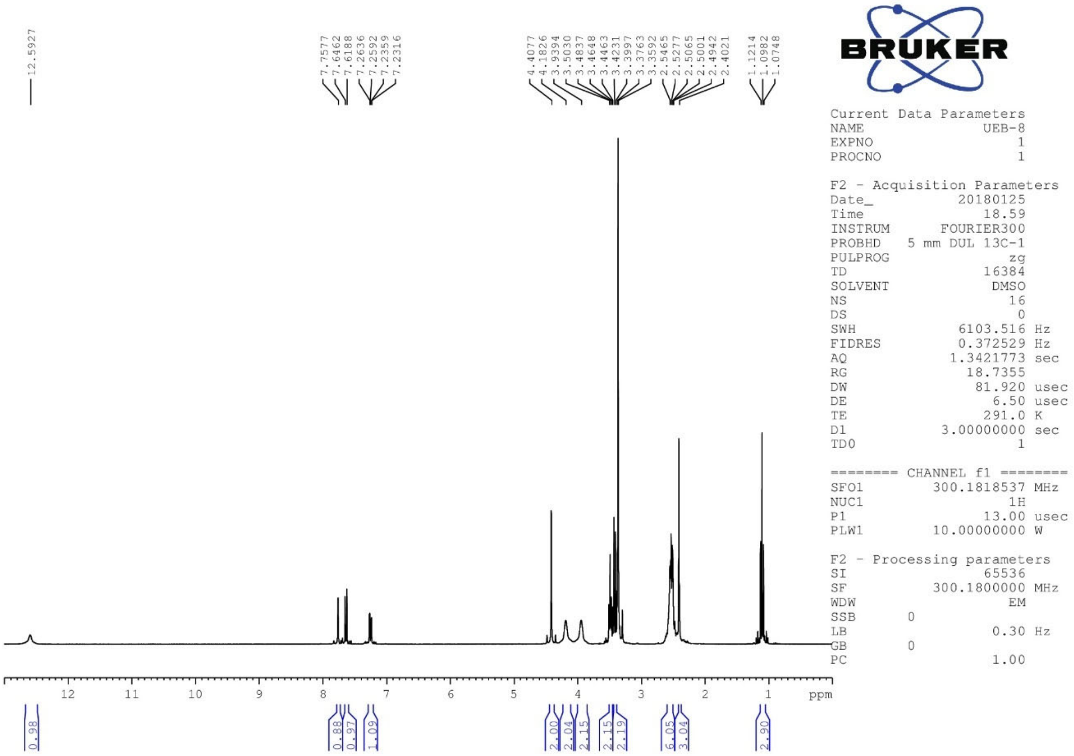

Figure S56. ^{13}C NMR spectrum of compound **19**

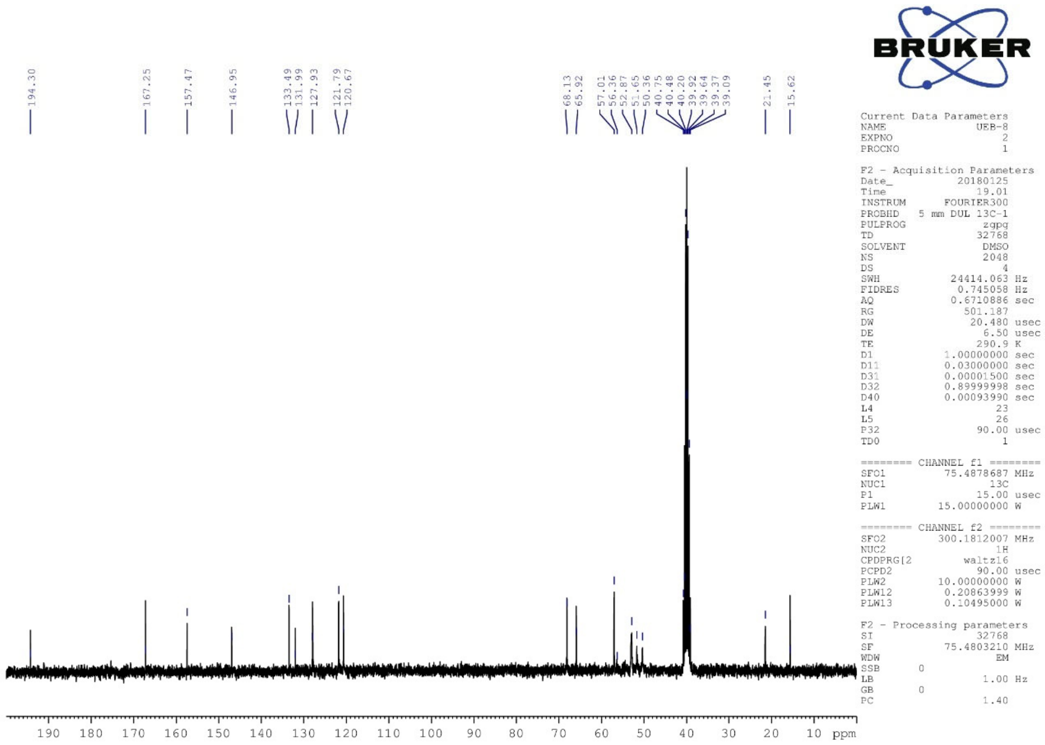

Figure S57. HRMS spectrum of compound **19**

Formula Predictor Report - UEB-8_19.lcd — Page 1 of 1

Data File: C:\LabSolutions\Data\Analiz\mdaltintop\UEB-8_19.lcd

Elmt	Val.	Min	Max	Elmt	Val.	Min	Max	Elmt	Val.	Min	Max	Elmt	Val.	Min	Max	Use Adduct
H	1	5	40	O	2	0	5	S	2	0	3	Ru	2	0	0	H
C	4	0	35	F	1	0	0	Cl	1	0	0	I	3	0	0	
N	3	3	6	P	3	0	0	Br	1	0	1					

Error Margin (ppm):	5	DBE Range:	5.0 - 15.0	Electron Ions:	both
HC Ratio:	unlimited	Apply N Rule:	yes	Use MSn Info:	yes
Max Isotopes:	3	Isotope RI (%):	1.00	Isotope Res:	9000
MSn Iso RI (%):	10.00	MSn Logic Mode:	AND	Max Results:	500

Event#: 1 MS(E+) Ret. Time : 5.573 -> 6.187 Scan# : 837 -> 929

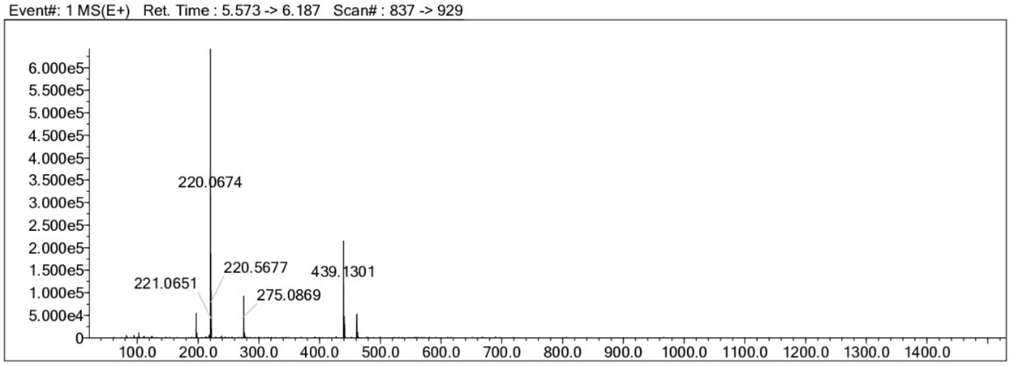

Measured region for 439.1301 m/z

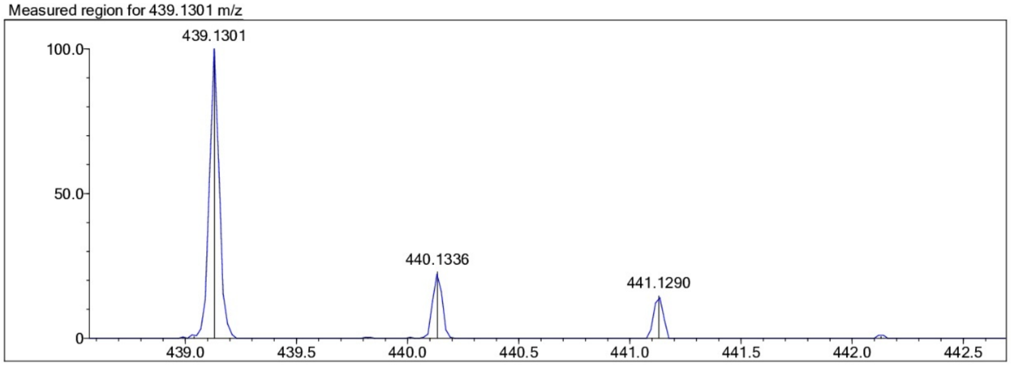

C19 H26 N4 O2 S3 [M+H]+ : Predicted region for 439.1291 m/z

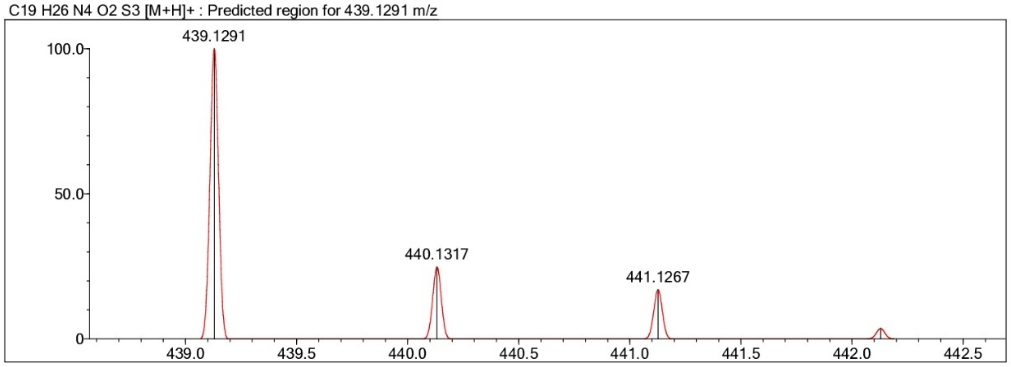

Rank	Score	Formula (M)	Ion	Meas. m/z	Pred. m/z	Df. (mDa)	Df. (ppm)	Iso	DBE
1	86.14	C19 H26 N4 O2 S3	[M+H]+	439.1301	439.1291	1.0	2.28	88.99	9.0

Figure S58. ^{1}H NMR spectrum of compound **20**

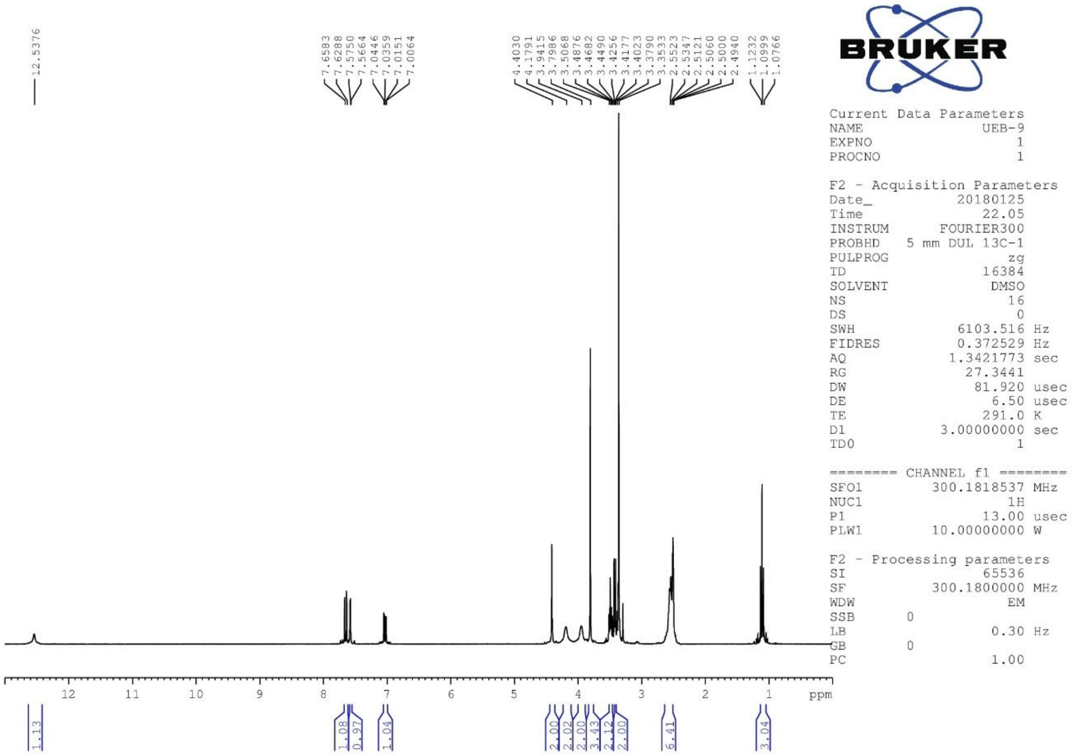

Figure S59. ^{13}C NMR spectrum of compound **20**

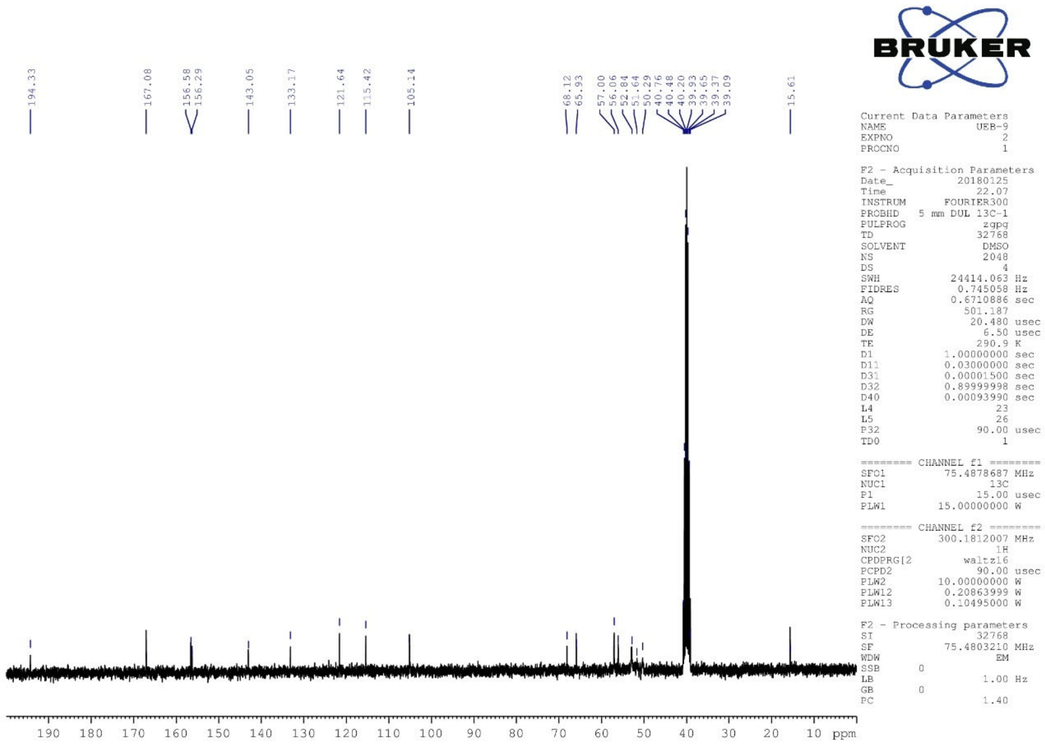

Figure S60. HRMS spectrum of compound **20**

Formula Predictor Report - UEB-9_20.lcd Page 1 of 1

Data File: C:\LabSolutions\Data\Analiz\mdaltintop\UEB-9_20.lcd

Elmt	Val.	Min	Max	Elmt	Val.	Min	Max	Elmt	Val.	Min	Max	Elmt	Val.	Min	Max	Use Adduct
H	1	5	40	O	2	0	5	S	2	0	3	Ru	2	0	0	H
C	4	0	35	F	1	0	0	Cl	1	0	0	I	3	0	0	
N	3	3	6	P	3	0	0	Br	1	0	1					

Error Margin (ppm):	5	DBE Range:	5.0 - 15.0	Electron Ions:	both
HC Ratio:	unlimited	Apply N Rule:	yes	Use MSn Info:	yes
Max Isotopes:	3	Isotope RI (%):	1.00	Isotope Res:	9000
MSn Iso RI (%):	10.00	MSn Logic Mode:	AND	Max Results:	500

Event#: 1 MS(E+) Ret. Time : 5.293 -> 5.787 Scan# : 795 -> 869

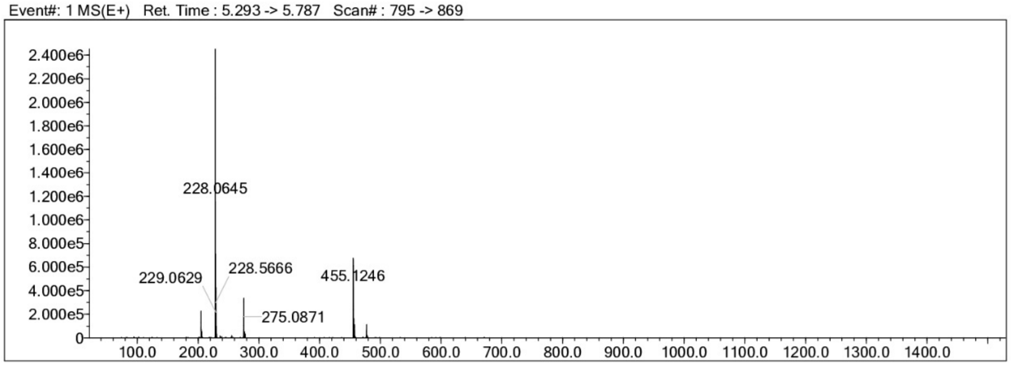

Measured region for 455.1246 m/z

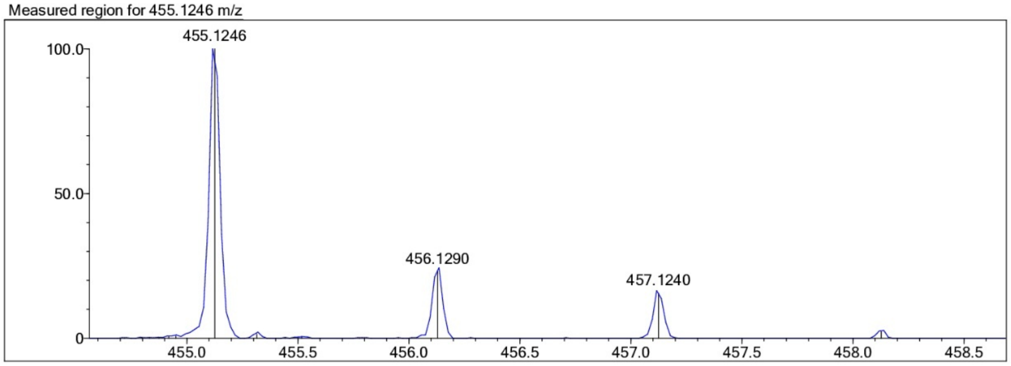

C19 H26 N4 O3 S3 [M+H]+ : Predicted region for 455.1240 m/z

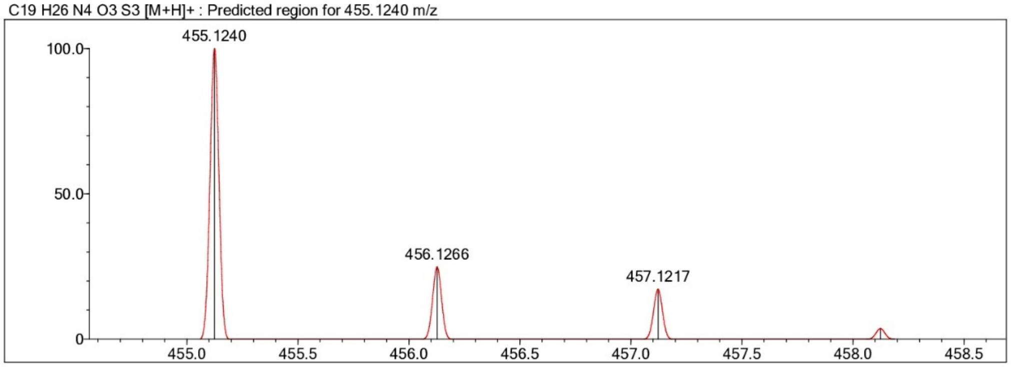

Rank	Score	Formula (M)	Ion	Meas. m/z	Pred. m/z	Df. (mDa)	Df. (ppm)	Iso	DBE
1	95.05	C19 H26 N4 O3 S3	[M+H]+	455.1246	455.1240	0.6	1.32	95.82	9.0

Figure S61. ^{1}H NMR spectrum of compound **21**

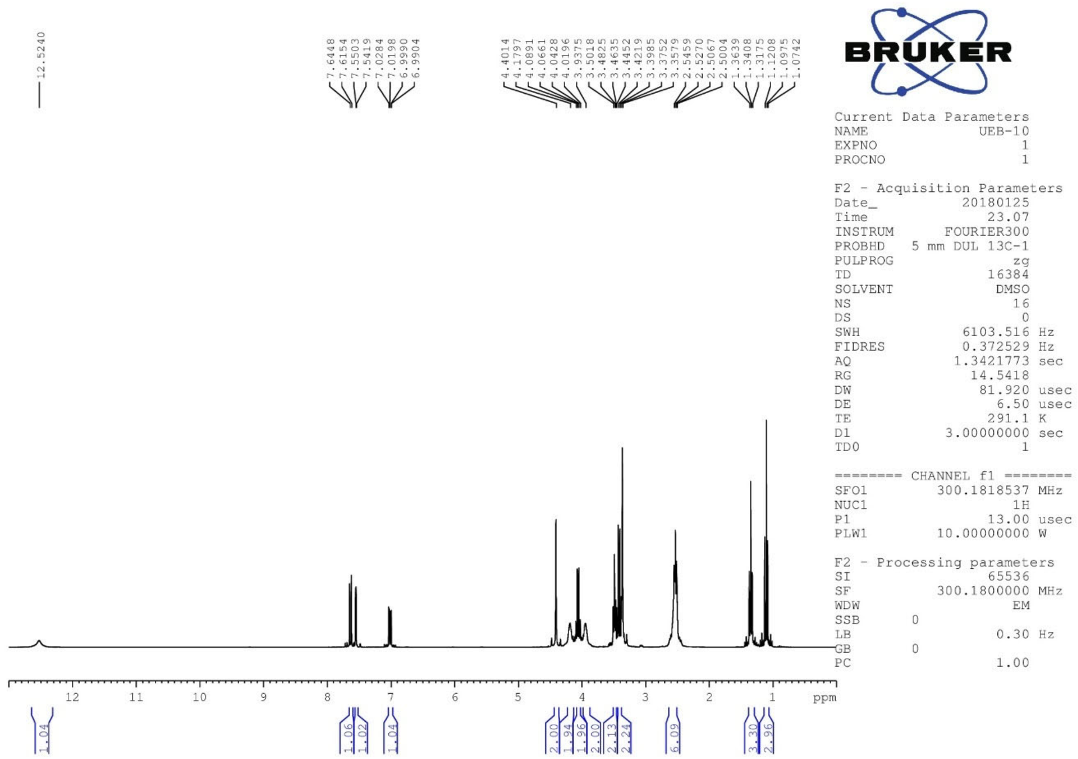

Figure S62. ^{13}C NMR spectrum of compound **21**

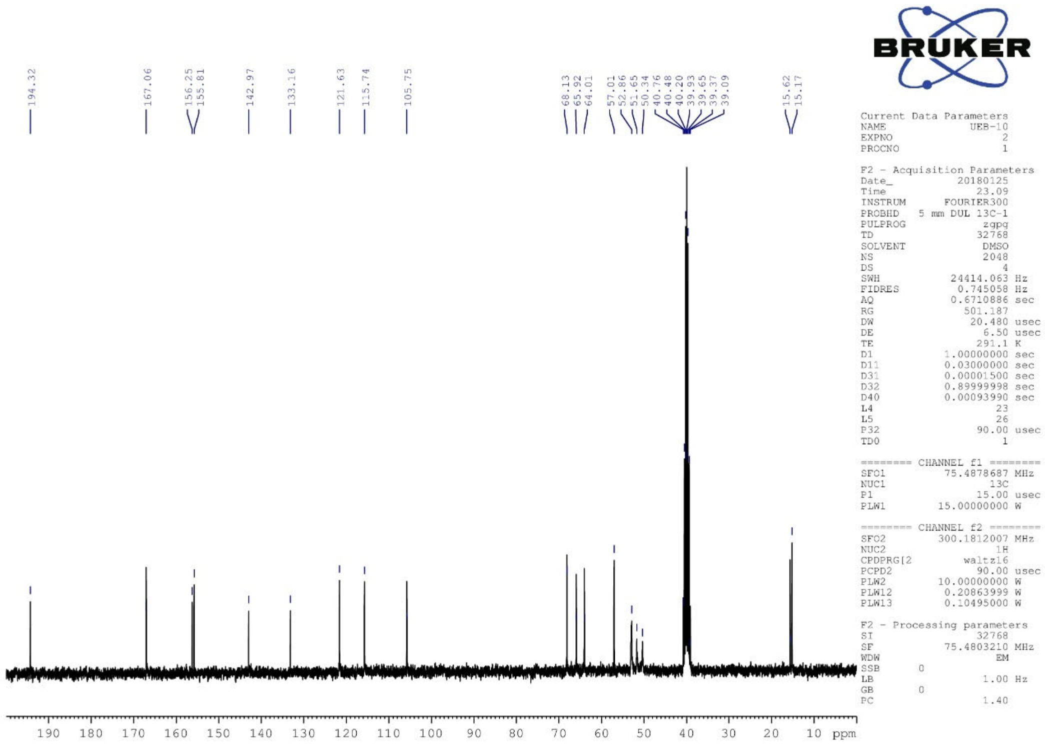

Figure S63. HRMS spectrum of compound **21**

Data File: C:\LabSolutions\Data\Analiz\mdaltintop\UEB-10_19.lcd

Elmt	Val.	Min	Max	Elmt	Val.	Min	Max	Elmt	Val.	Min	Max	Elmt	Val.	Min	Max	Use Adduct
H	1	5	40	O	2	0	4	S	2	0	3	Ru	2	0	0	H
C	4	0	35	F	1	0	0	Cl	1	0	1	I	3	0	0	
N	3	3	4	P	3	0	0	Br	1	0	0					

Error Margin (ppm):	5	DBE Range:	5.0 - 10.0	Electron Ions:	both
HC Ratio:	unlimited	Apply N Rule:	yes	Use MSn Info:	yes
Max Isotopes:	3	Isotope RI (%):	1.00	Isotope Res:	9000
MSn Iso RI (%):	10.00	MSn Logic Mode:	AND	Max Results:	500

Event#: 1 MS(E+) Ret. Time : 5.467 -> 6.307 Scan# : 821 -> 947

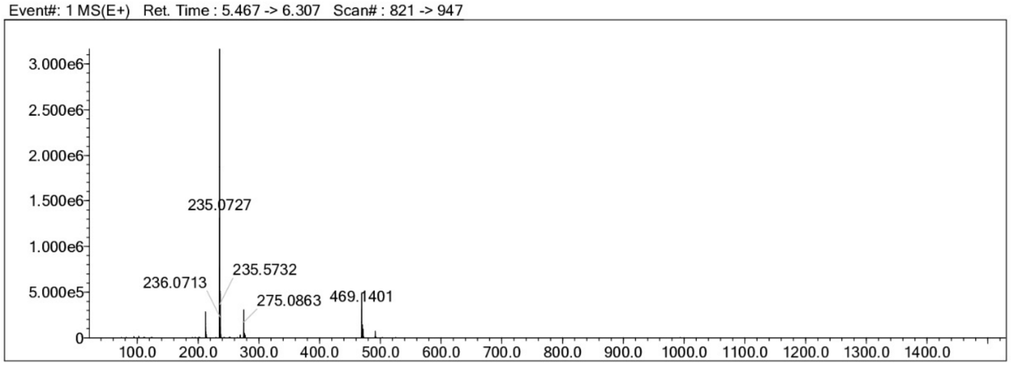

Measured region for 469.1401 m/z

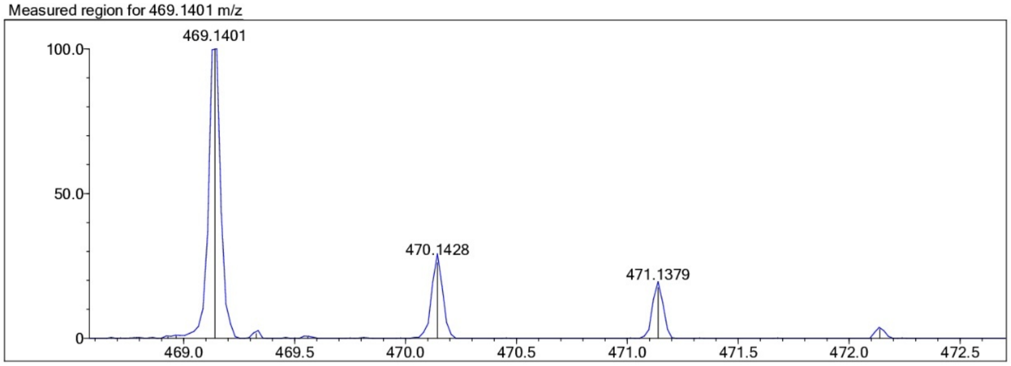

C20 H28 N4 O3 S3 [M+H]+ : Predicted region for 469.1396 m/z

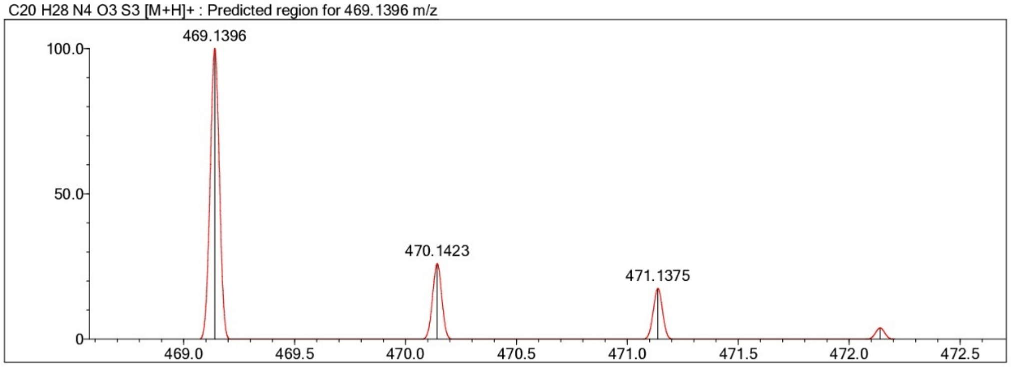

Rank	Score	Formula (M)	Ion	Meas. m/z	Pred. m/z	Df. (mDa)	Df. (ppm)	Iso	DBE
1	99.83	C20 H28 N4 O3 S3	[M+H]+	469.1401	469.1396	0.5	1.07	100.00	9.0

Figure S64. ^{1}H NMR spectrum of compound **22**

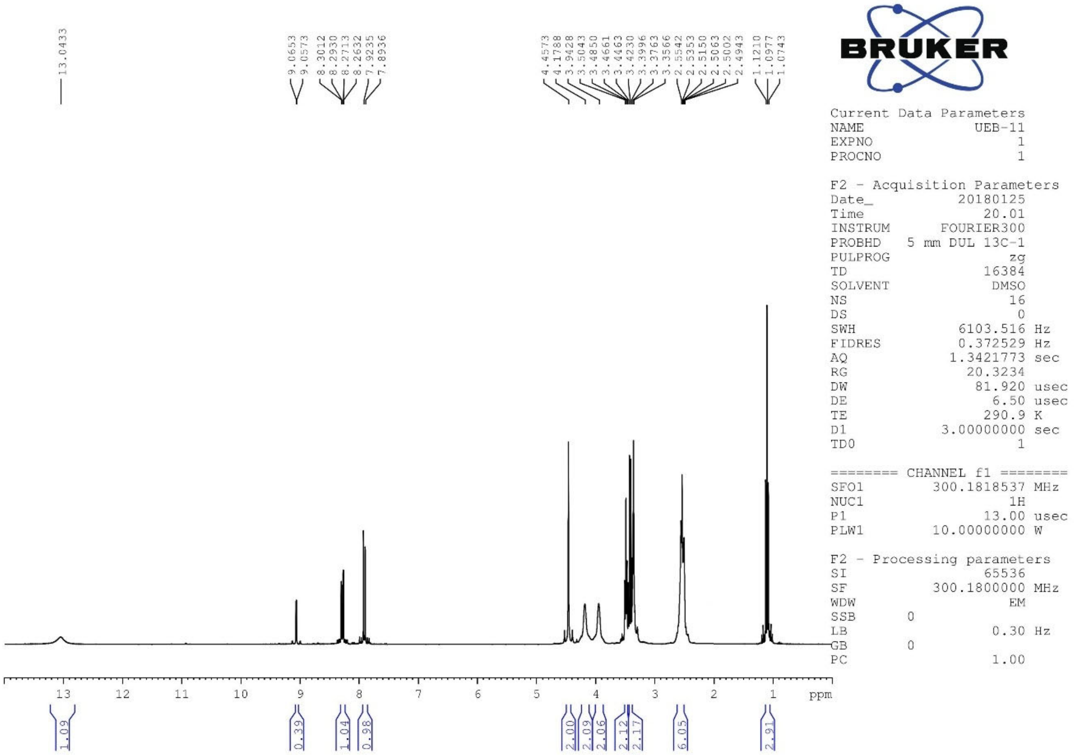

Figure S65. ^{13}C NMR spectrum of compound **22**

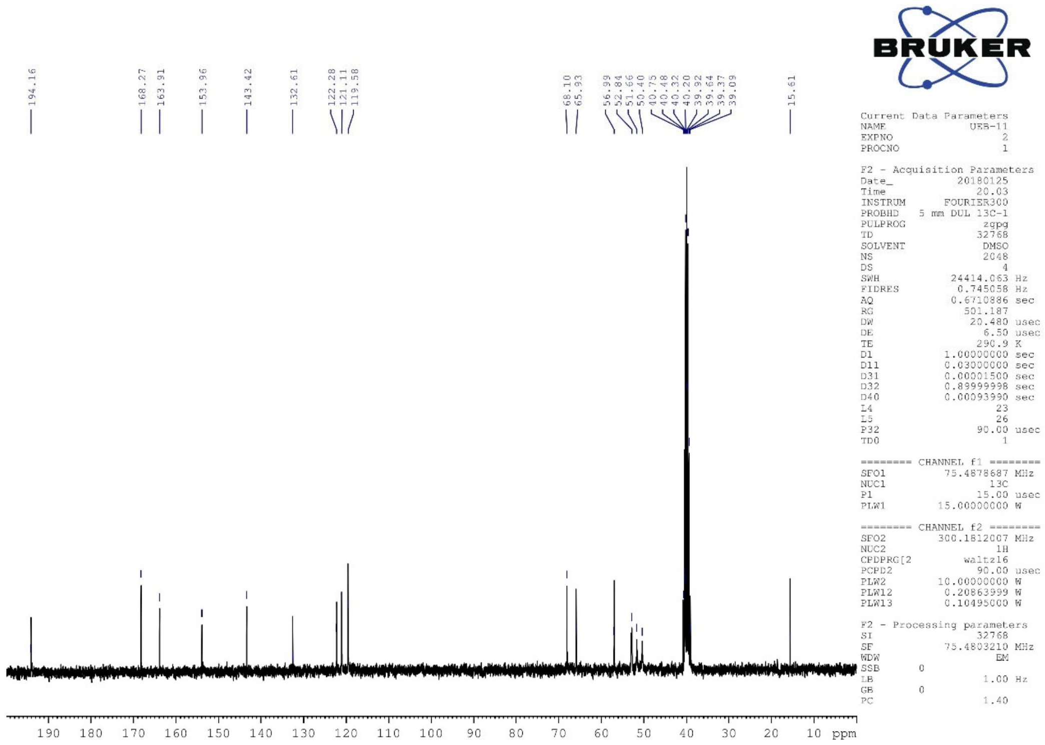

Figure S66. HRMS spectrum of compound **22**

Formula Predictor Report - UEB-11_20.lcd

Page 1 of 1

Data File: C:\LabSolutions\Data\Analiz\mdaltintop\UEB-11_20.lcd

Elmt	Val.	Min	Max	Elmt	Val.	Min	Max	Elmt	Val.	Min	Max	Elmt	Val.	Min	Max	Use Adduct
H	1	5	40	O	2	0	4	S	2	0	3	Ru	2	0	0	H
C	4	0	35	F	1	0	0	Cl	1	0	0	I	3	0	0	
N	3	3	5	P	3	0	0	Br	1	0	0					

Error Margin (ppm):	5	DBE Range:	5.0 - 15.0	Electron Ions:	both
HC Ratio:	unlimited	Apply N Rule:	yes	Use MSn Info:	yes
Max Isotopes:	3	Isotope RI (%):	1.00	Isotope Res:	9000
MSn Iso RI (%):	10.00	MSn Logic Mode:	AND	Max Results:	500

Event#: 1 MS(E+) Ret. Time : 5.360 -> 6.227 Scan# : 805 -> 935

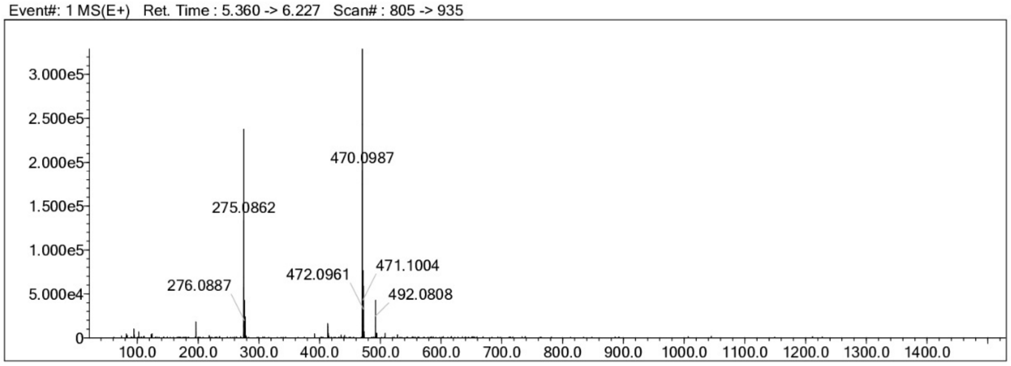

Measured region for 470.0987 m/z

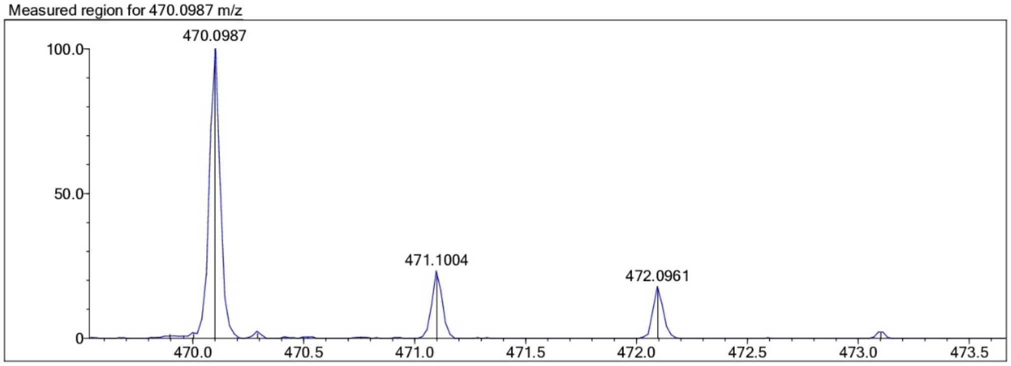

C18 H23 N5 O4 S3 [M+H]+ : Predicted region for 470.0985 m/z

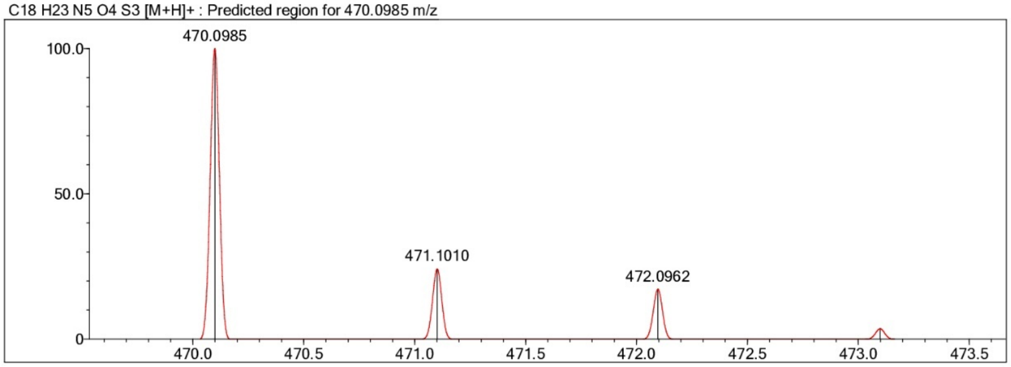

Rank	Score	Formula (M)	Ion	Meas. m/z	Pred. m/z	Df. (mDa)	Df. (ppm)	Iso	DBE
1	94.21	C18 H23 N5 O4 S3	[M+H]+	470.0987	470.0985	0.2	0.43	94.21	10.0

Figure S67. ^{1}H NMR spectrum of compound **23**

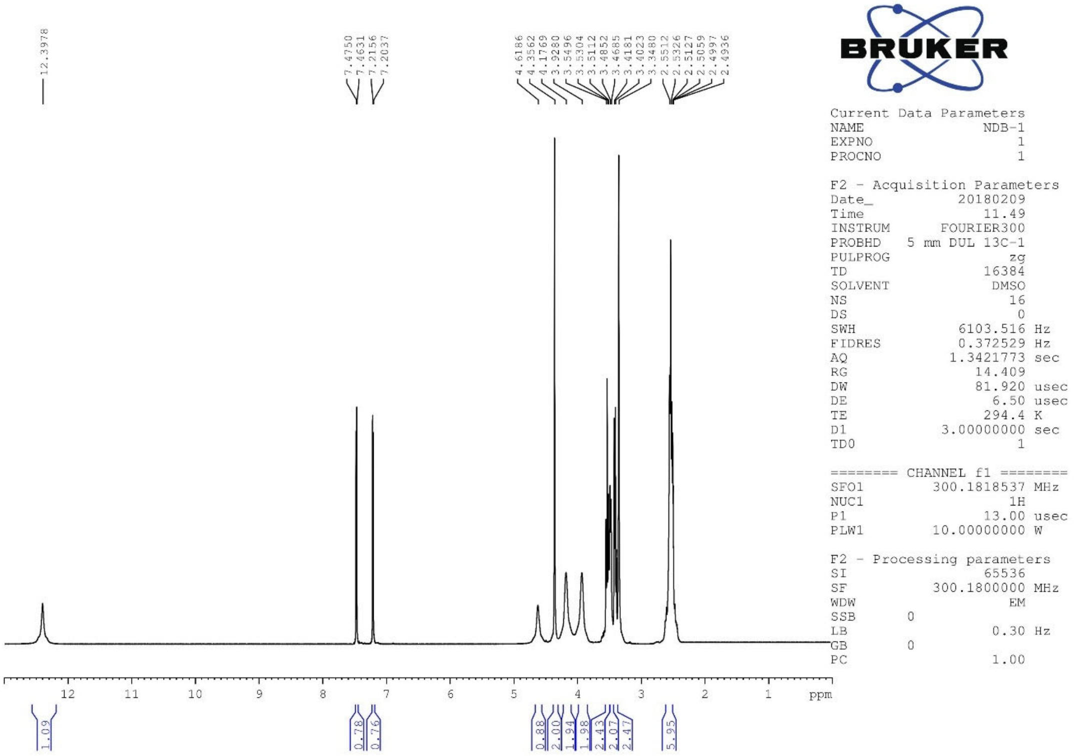

Figure S68. ^{13}C NMR spectrum of compound **23**

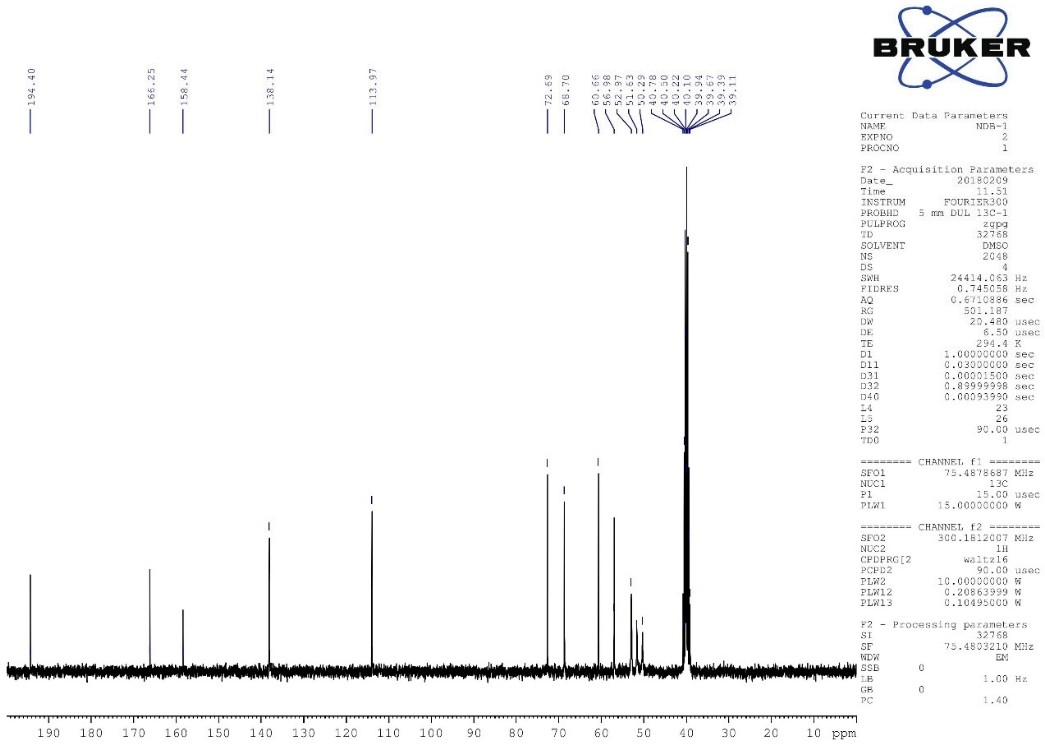

Figure S69. HRMS spectrum of compound **23**

Formula Predictor Report - NDB-1_31.lcd

Page 1 of 1

Data File: C:\LabSolutions\Data\Analiz\mdaltintop\NDB-1_31.lcd

Elmt	Val.	Min	Max	Elmt	Val.	Min	Max	Elmt	Val.	Min	Max	Elmt	Val.	Min	Max	Use Adduct
H	1	5	40	O	2	0	4	S	2	0	3	Ru	2	0	0	H
C	4	0	35	F	1	0	0	Cl	1	0	0	I	3	0	0	
N	3	3	5	P	3	0	0	Br	1	0	0					

Error Margin (ppm):	5	DBE Range:	5.0 - 10.0	Electron Ions:	both
HC Ratio:	unlimited	Apply N Rule:	yes	Use MSn Info:	yes
Max Isotopes:	3	Isotope RI (%):	1.00	Isotope Res:	9000
MSn Iso RI (%):	10.00	MSn Logic Mode:	AND	Max Results:	500

Event#: 1 MS(E+) Ret. Time : 2.627 -> 3.493 Scan# : 395 -> 525

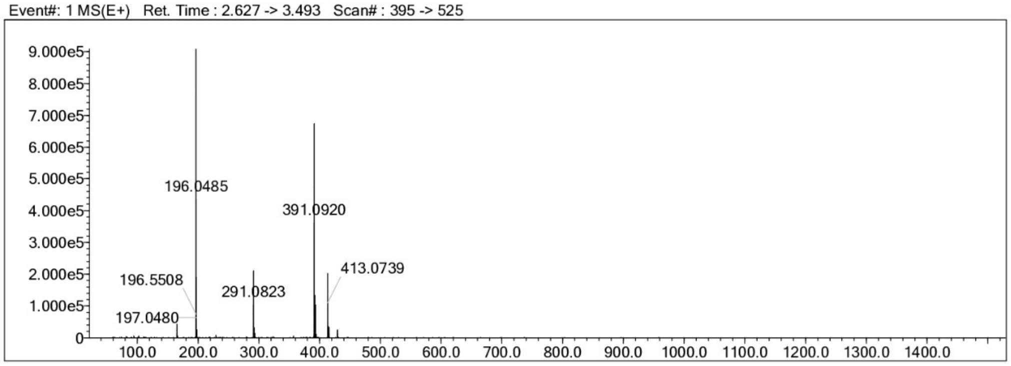

Measured region for 391.0920 m/z

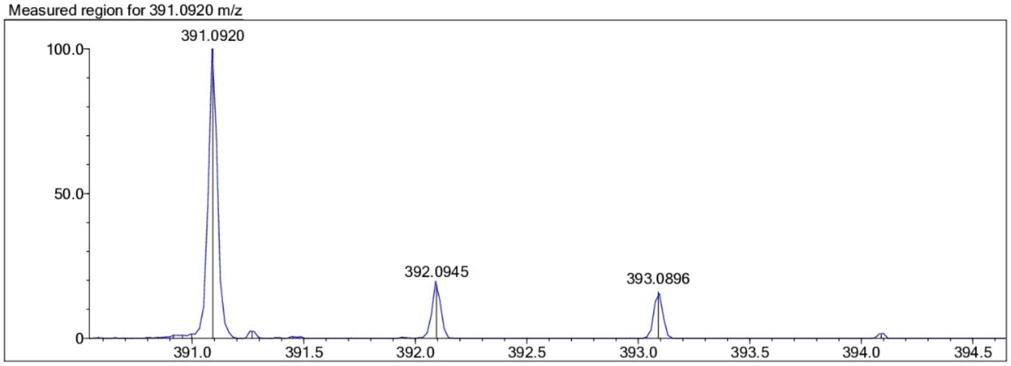

C14 H22 N4 O3 S3 [M+H]+ : Predicted region for 391.0927 m/z

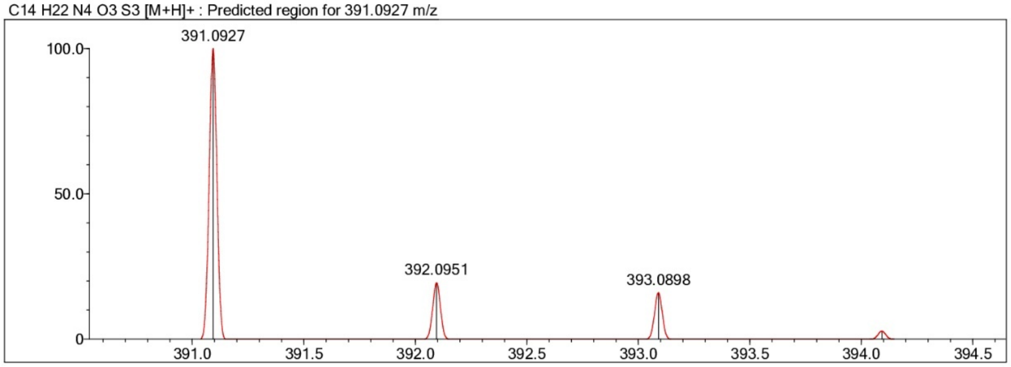

Rank	Score	Formula (M)	Ion	Meas. m/z	Pred. m/z	Df. (mDa)	Df. (ppm)	Iso	DBE
1	86.40	C14 H22 N4 O3 S3	[M+H]+	391.0920	391.0927	-0.7	-1.79	88.14	6.0

Figure S70. ^{1}H NMR spectrum of compound **24**

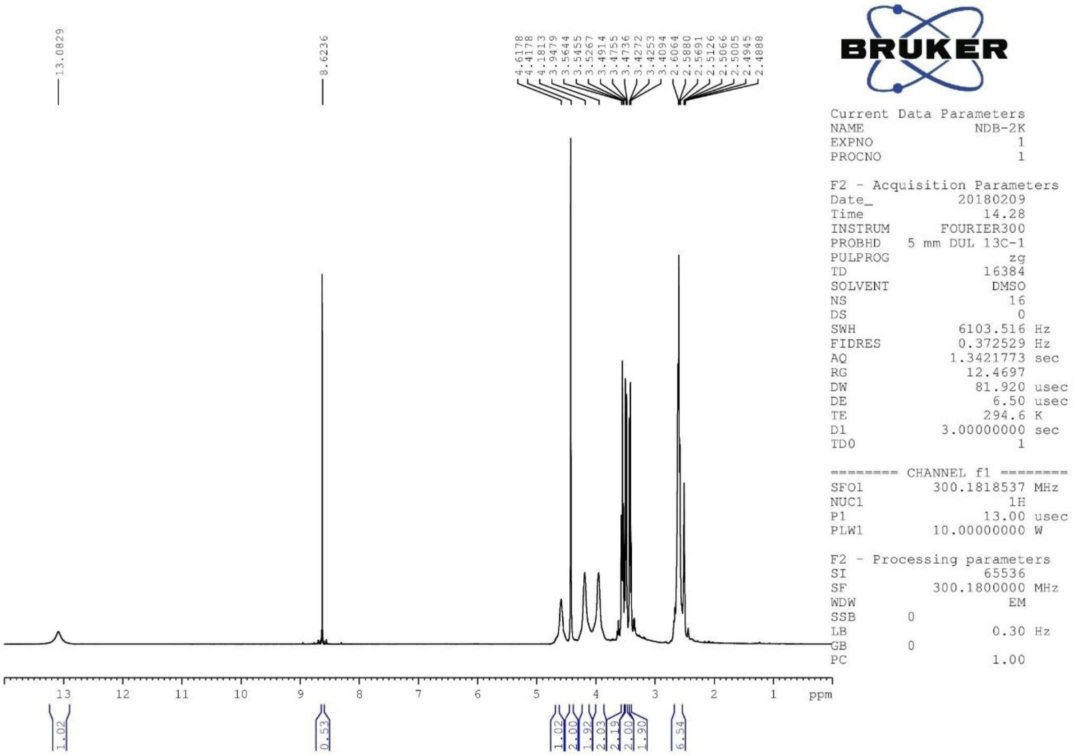

Figure S71. ^{13}C NMR spectrum of compound **24**

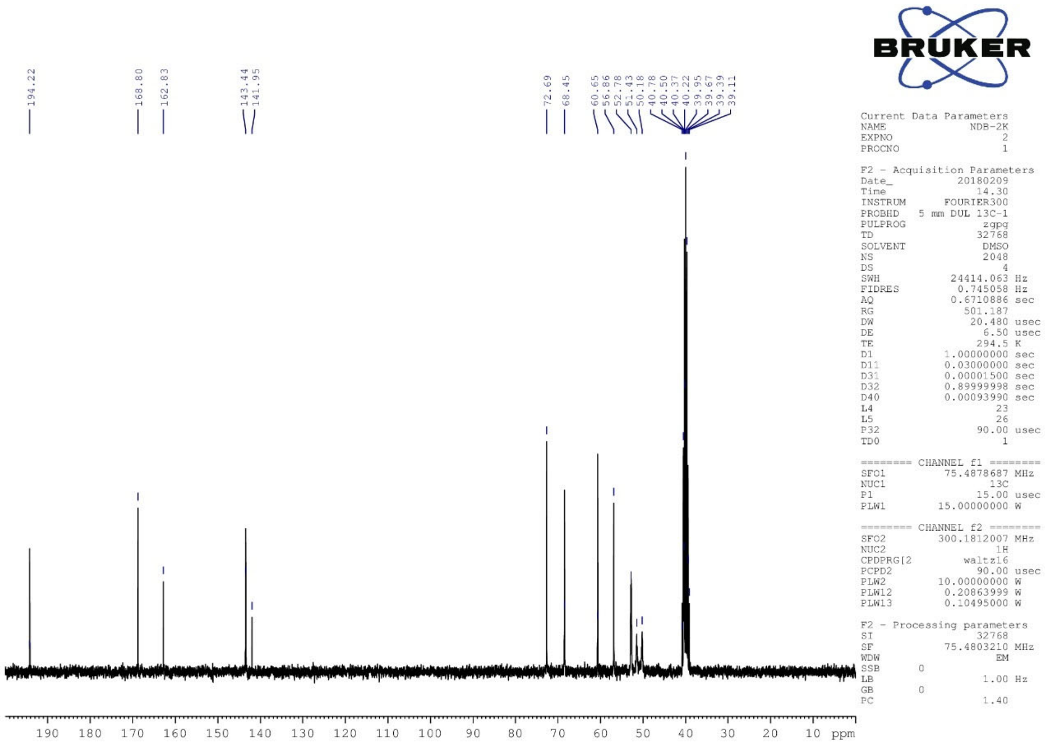

Figure S72. HRMS spectrum of compound **24**

Formula Predictor Report - NDB-2_32.lcd

Page 1 of 1

Data File: C:\LabSolutions\Data\Analiz\mdaltintop\NDB-2_32.lcd

Elmt	Val.	Min	Max	Elmt	Val.	Min	Max	Elmt	Val.	Min	Max	Elmt	Val.	Min	Max	Use Adduct
H	1	5	40	O	2	0	5	S	2	0	3	Ru	2	0	0	H
C	4	0	35	F	1	0	0	Cl	1	0	0	I	3	0	0	
N	3	3	5	P	3	0	0	Br	1	0	0					

Error Margin (ppm): 5
HC Ratio: unlimited
Max Isotopes: 3
MSn Iso RI (%): 10.00

DBE Range: 5.0 - 10.0
Apply N Rule: yes
Isotope RI (%): 1.00
MSn Logic Mode: AND

Electron Ions: both
Use MSn Info: yes
Isotope Res: 9000
Max Results: 500

Event#: 1 MS(E+) Ret. Time : 4.387 -> 5.080 Scan# : 659 -> 763

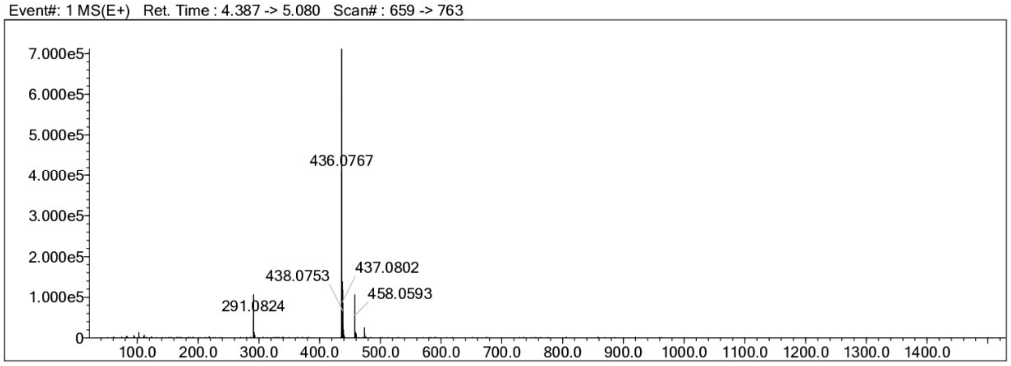

Measured region for 436.0767 m/z

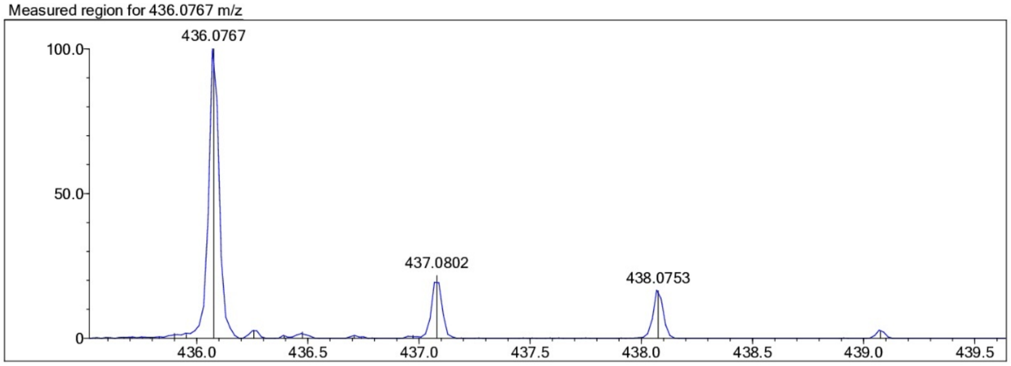

C14 H21 N5 O5 S3 [M+H]+ : Predicted region for 436.0778 m/z

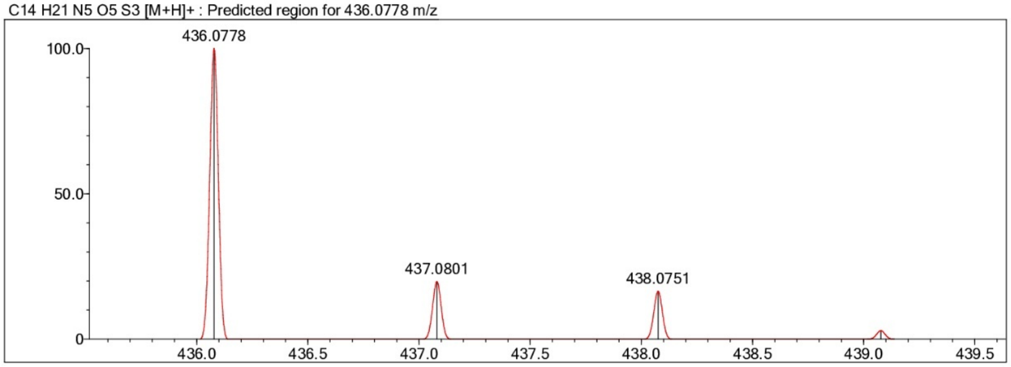

Rank	Score	Formula (M)	Ion	Meas. m/z	Pred. m/z	Df. (mDa)	Df. (ppm)	Iso	DBE
1	86.43	C14 H21 N5 O5 S3	[M+H]+	436.0767	436.0778	-1.1	-2.52	89.84	7.0

Figure S73. ^{1}H NMR spectrum of compound **25**

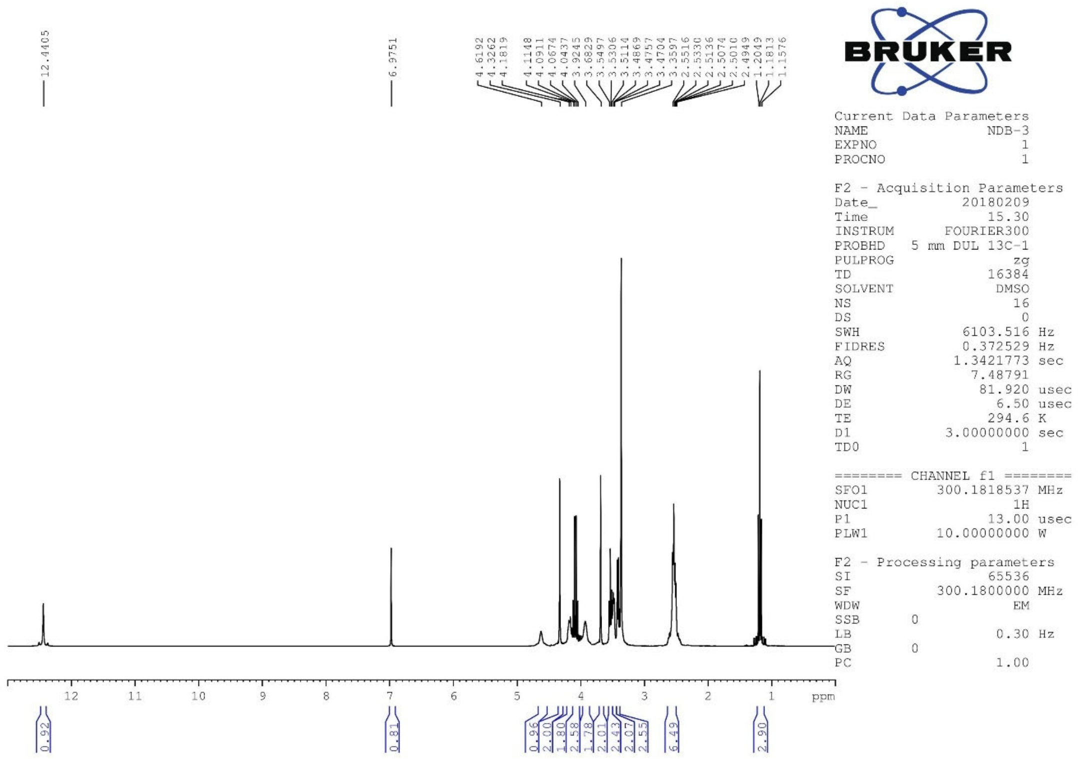

Figure S74. ¹³C NMR spectrum of compound **25**

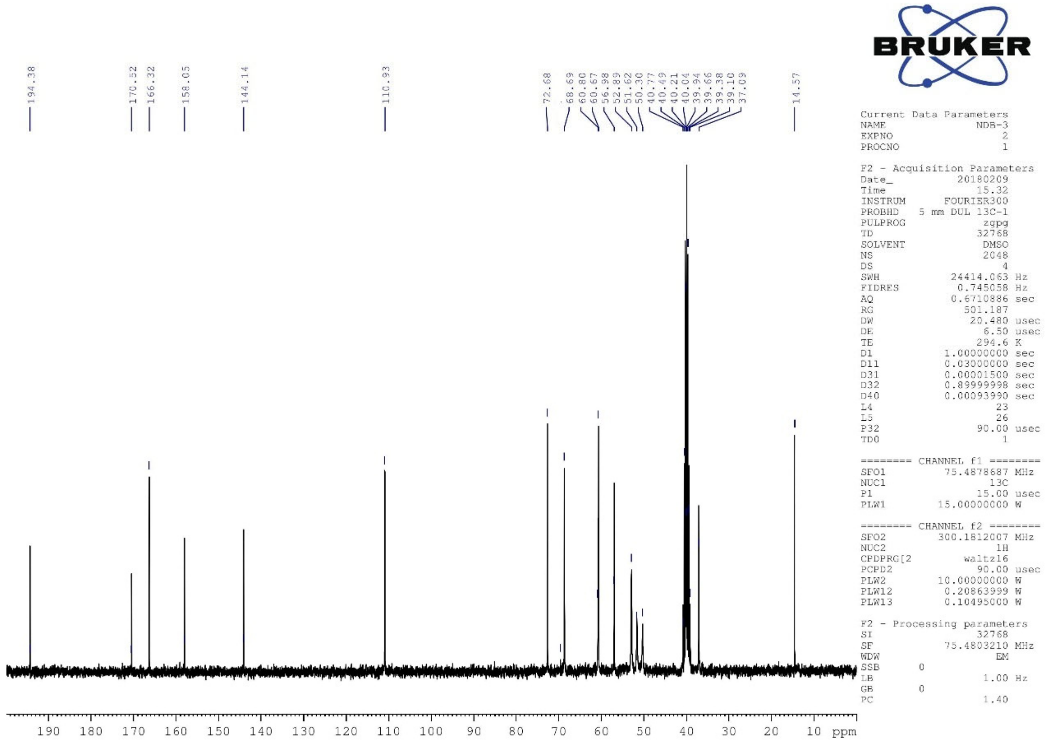

Figure S75. HRMS spectrum of compound **25**

Formula Predictor Report - NDB-3_33.lcd | Page 1 of 1

Data File: C:\LabSolutions\Data\Analiz\mdaltintop\NDB-3_33.lcd

Elmt	Val.	Min	Max	Elmt	Val.	Min	Max	Elmt	Val.	Min	Max	Elmt	Val.	Min	Max	Use Adduct
H	1	5	40	O	2	0	5	S	2	0	3	Ru	2	0	0	H
C	4	0	35	F	1	0	0	Cl	1	0	0	I	3	0	0	
N	3	3	5	P	3	0	0	Br	1	0	0					

Error Margin (ppm):	5	DBE Range:	5.0 - 10.0	Electron Ions:	both
HC Ratio:	unlimited	Apply N Rule:	yes	Use MSn Info:	yes
Max Isotopes:	3	Isotope RI (%):	1.00	Isotope Res:	9000
MSn Iso RI (%):	10.00	MSn Logic Mode:	AND	Max Results:	500

Event#: 1 MS(E+) Ret. Time : 4.520 -> 5.213 Scan# : 679 -> 783

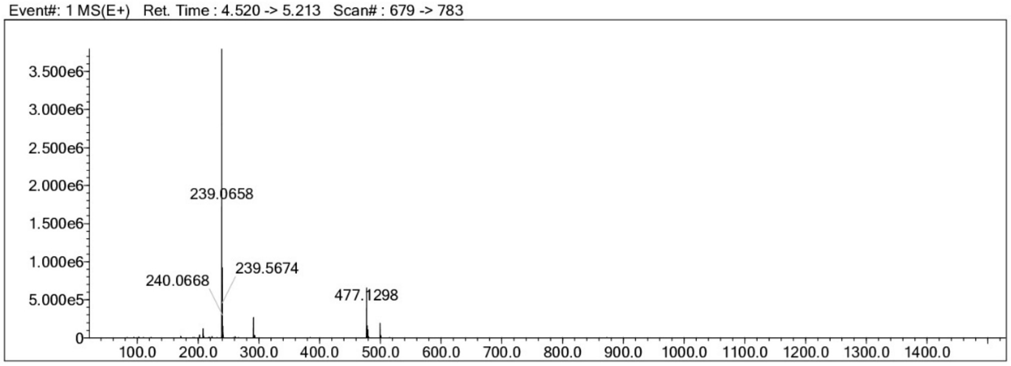

Measured region for 477.1298 m/z

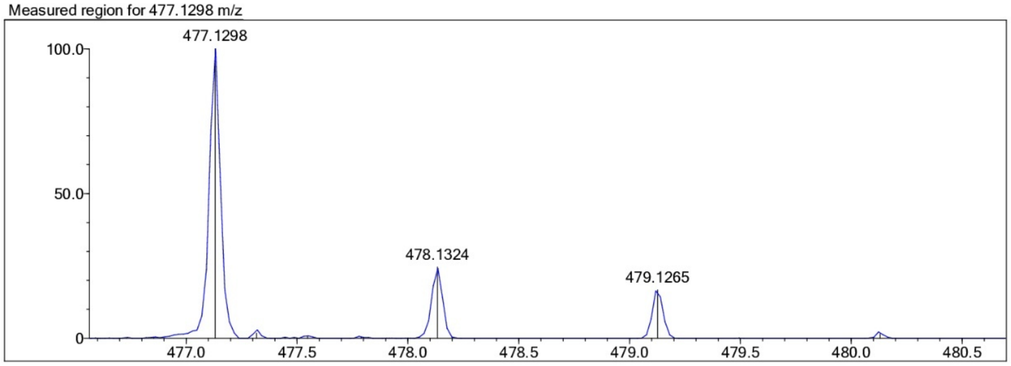

C18 H28 N4 O5 S3 [M+H]+ : Predicted region for 477.1295 m/z

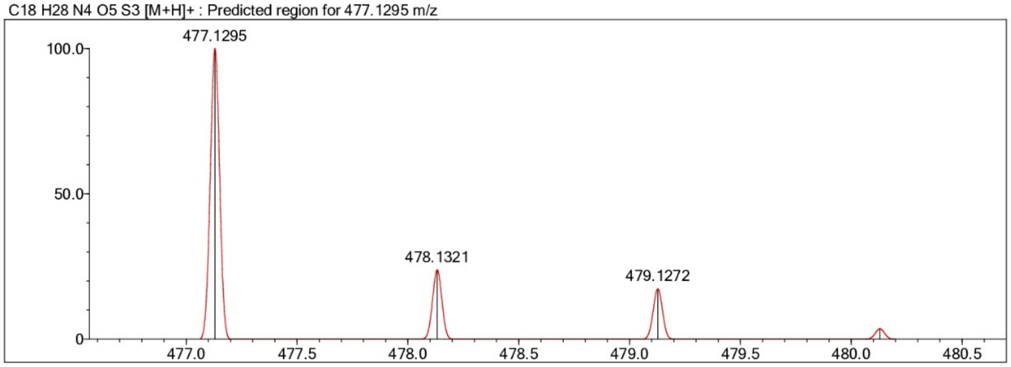

Rank	Score	Formula (M)	Ion	Meas. m/z	Pred. m/z	Df. (mDa)	Df. (ppm)	Iso	DBE
1	94.13	C18 H28 N4 O5 S3	[M+H]+	477.1298	477.1295	0.3	0.63	94.13	7.0

Figure S76. ^{1}H NMR spectrum of compound **26**

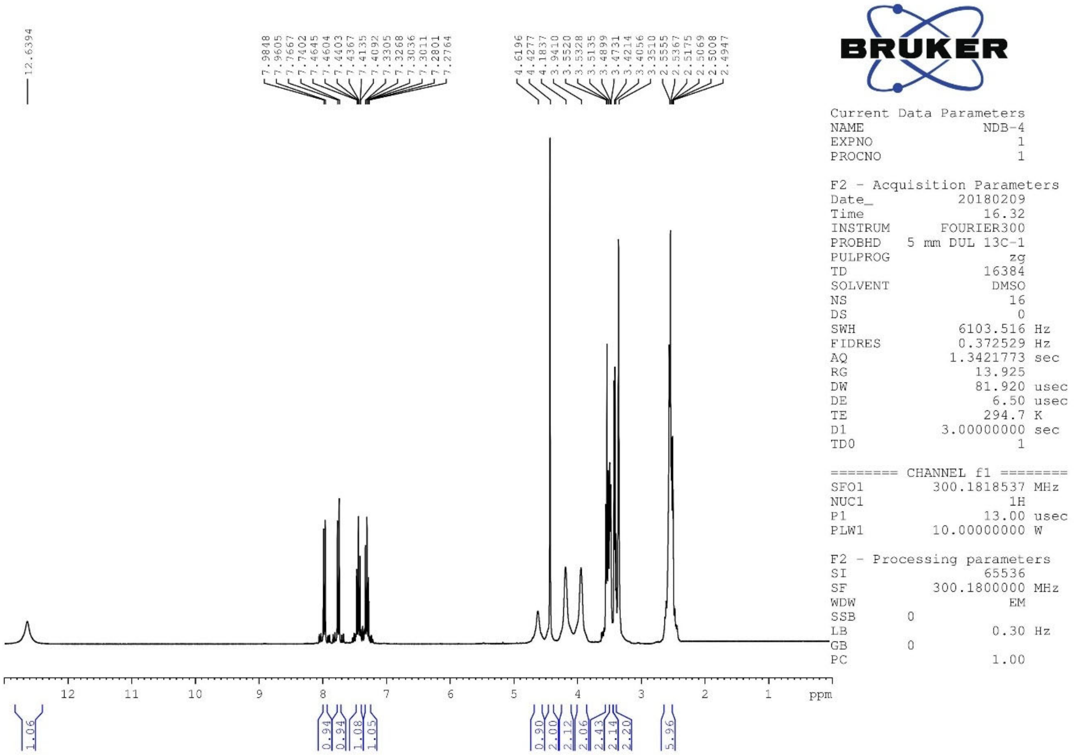

Figure S77. ^{13}C NMR spectrum of compound **26**

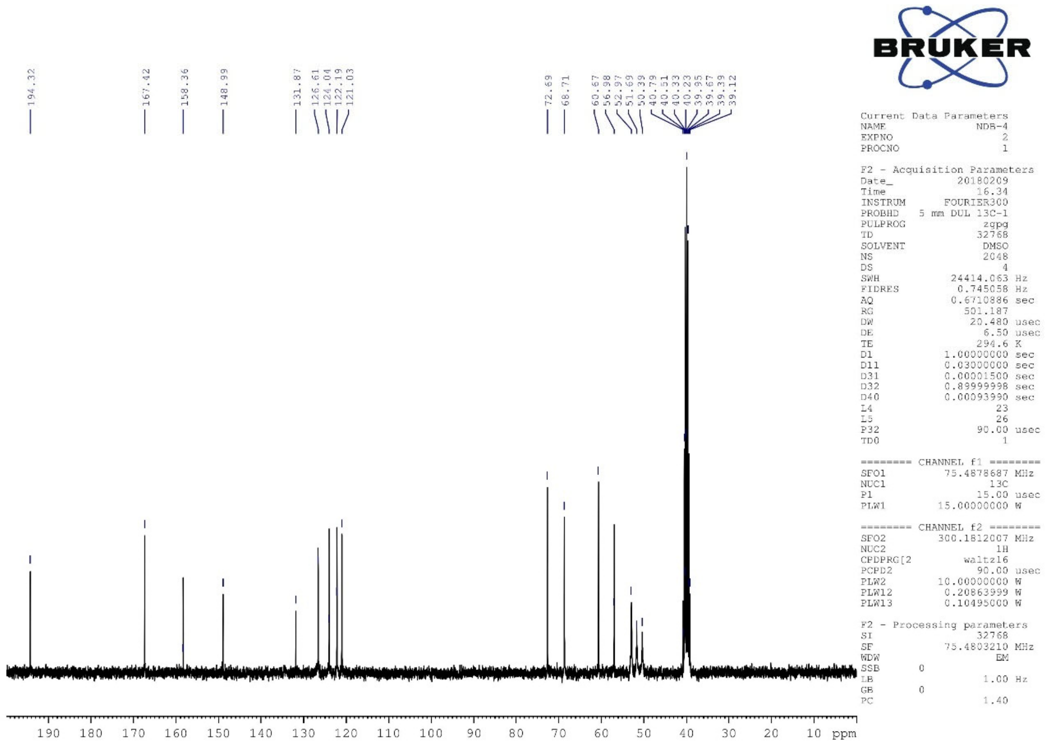

Figure S78. HRMS spectrum of compound **26**

Data File: C:\LabSolutions\Data\Analiz\mdaltintop\NDB-4_34.lcd

Elmt	Val.	Min	Max	Elmt	Val.	Min	Max	Elmt	Val.	Min	Max	Elmt	Val.	Min	Max	Use Adduct
H	1	5	40	O	2	0	5	S	2	0	3	Ru	2	0	0	H
C	4	0	35	F	1	0	0	Cl	1	0	0	I	3	0	0	
N	3	3	5	P	3	0	0	Br	1	0	0					

Error Margin (ppm): 5
HC Ratio: unlimited
Max Isotopes: 3
MSn Iso RI (%): 10.00

DBE Range: 5.0 - 10.0
Apply N Rule: yes
Isotope RI (%): 1.00
MSn Logic Mode: AND

Electron Ions: both
Use MSn Info: yes
Isotope Res: 9000
Max Results: 500

Event#: 1 MS(E+) Ret. Time : 4.933 -> 5.387 Scan# : 741 -> 809

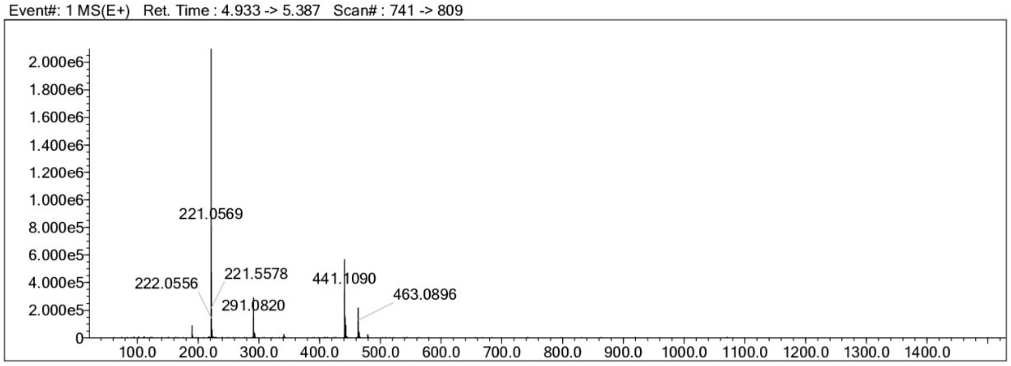

Measured region for 441.1090 m/z

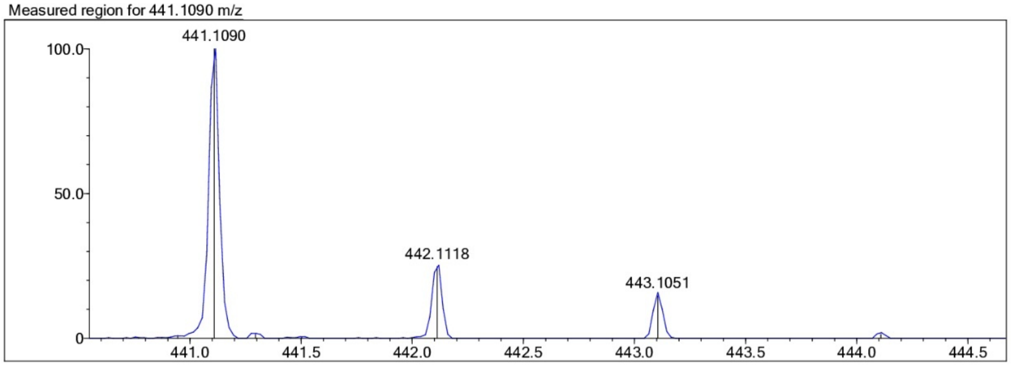

C18 H24 N4 O3 S3 [M+H]+ : Predicted region for 441.1083 m/z

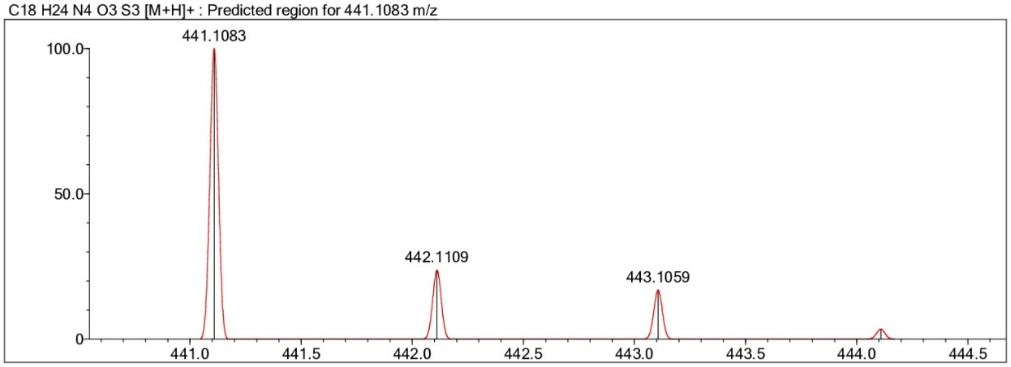

Rank	Score	Formula (M)	Ion	Meas. m/z	Pred. m/z	Df. (mDa)	Df. (ppm)	Iso	DBE
1	92.17	C18 H24 N4 O3 S3	[M+H]+	441.1090	441.1083	0.7	1.59	93.55	9.0

Figure S79. ^{1}H NMR spectrum of compound **27**

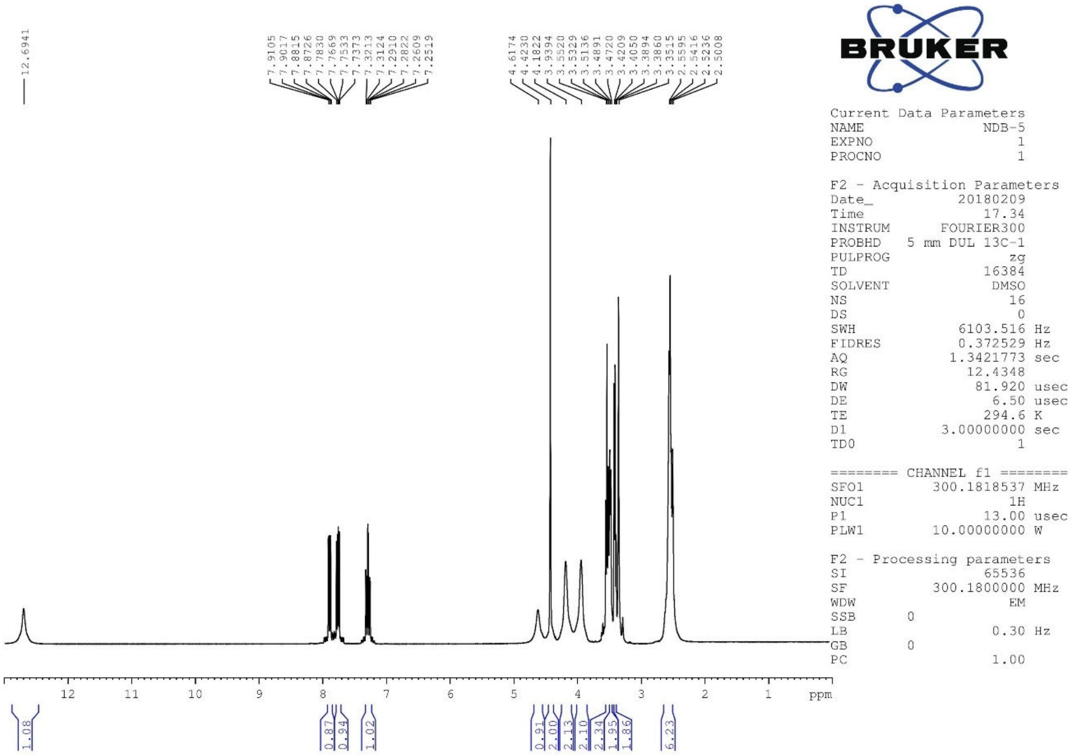

Figure S80. ^{13}C NMR spectrum of compound **27**

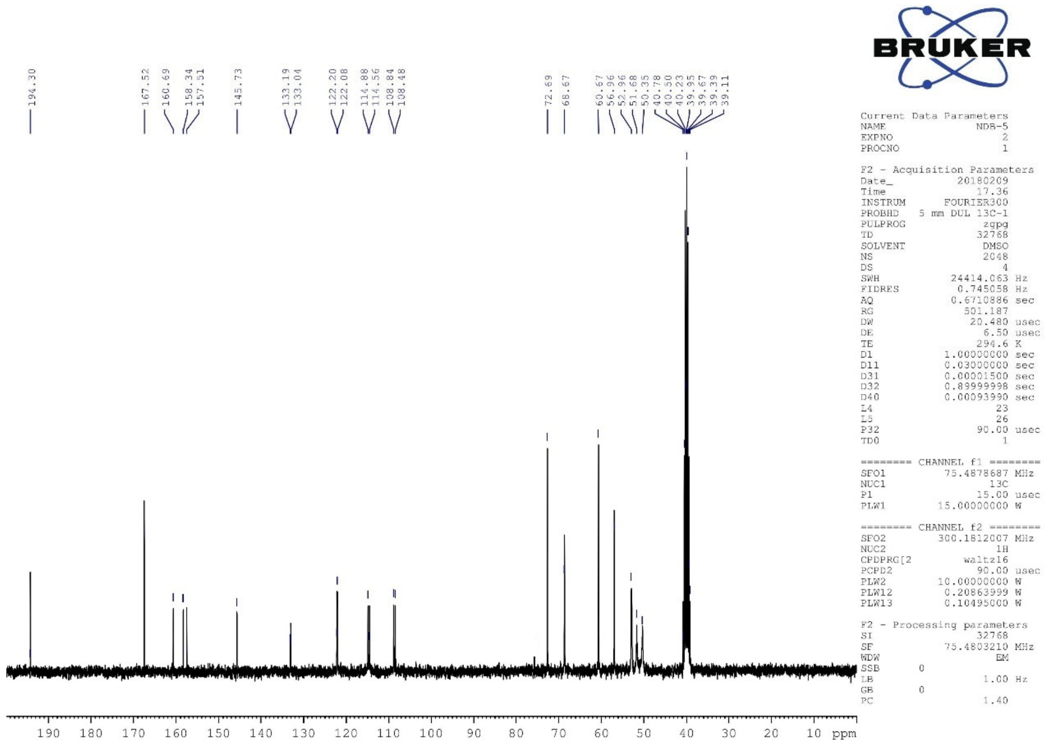

Figure S81. HRMS spectrum of compound **27**

Formula Predictor Report - NDB-5_35.lcd — Page 1 of 1

Data File: C:\LabSolutions\Data\Analiz\mdaltintop\NDB-5_35.lcd

Elmt	Val.	Min	Max	Elmt	Val.	Min	Max	Elmt	Val.	Min	Max	Elmt	Val.	Min	Max	Use Adduct
H	1	5	40	O	2	0	5	S	2	0	3	Ru	2	0	0	H
C	4	0	35	F	1	0	1	Cl	1	0	0	I	3	0	0	
N	3	3	5	P	3	0	0	Br	1	0	0					

Error Margin (ppm):	5	DBE Range:	5.0 - 10.0	Electron Ions:	both
HC Ratio:	unlimited	Apply N Rule:	yes	Use MSn Info:	yes
Max Isotopes:	3	Isotope RI (%):	1.00	Isotope Res:	9000
MSn Iso RI (%):	10.00	MSn Logic Mode:	AND	Max Results:	500

Event#: 1 MS(E+) Ret. Time : 4.933 -> 5.693 Scan# : 741 -> 855

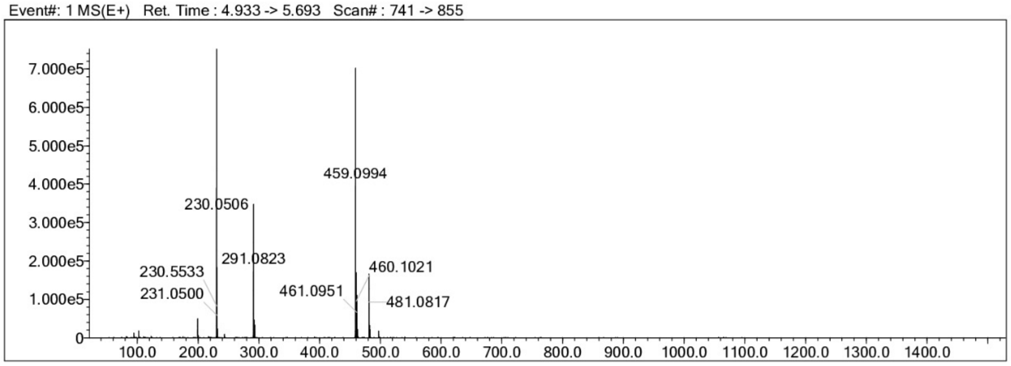

Measured region for 459.0994 m/z

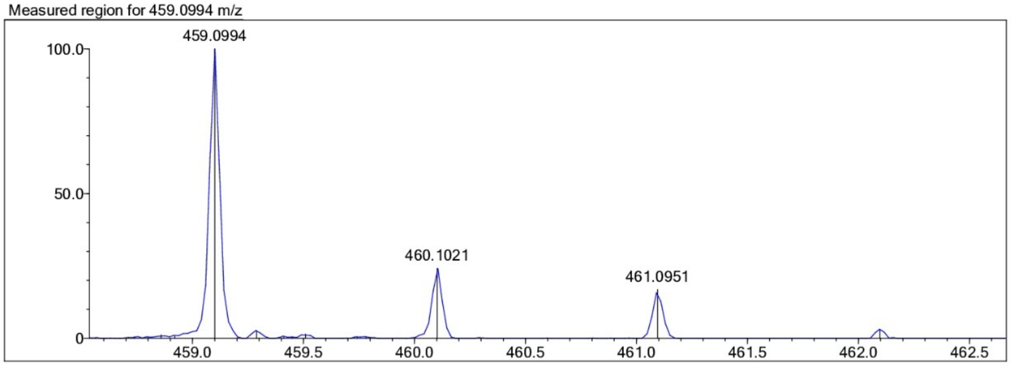

C18 H23 N4 O3 F S3 [M+H]+ : Predicted region for 459.0989 m/z

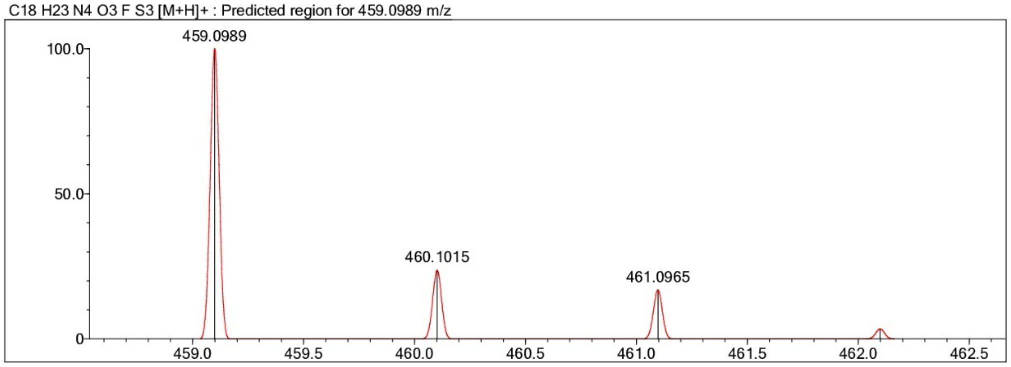

Rank	Score	Formula (M)	Ion	Meas. m/z	Pred. m/z	Df. (mDa)	Df. (ppm)	Iso	DBE
1	99.78	C18 H23 N4 O3 F S3	[M+H]+	459.0994	459.0989	0.5	1.09	100.00	9.0

Figure S82. ^{1}H NMR spectrum of compound **28**

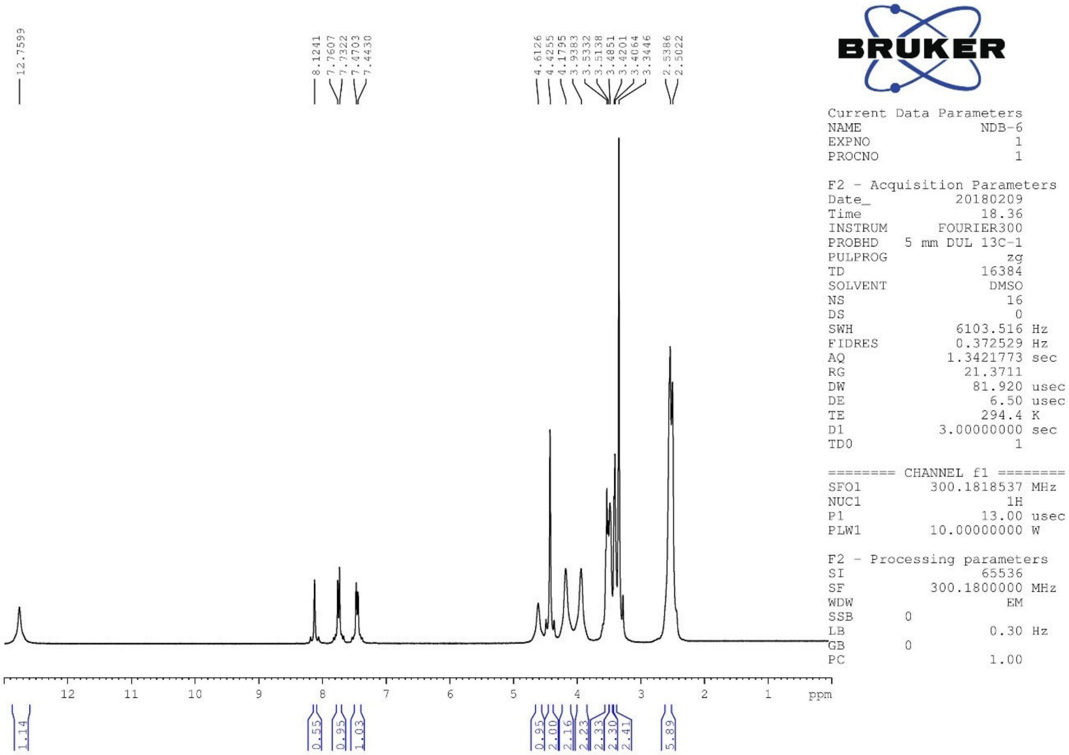

Figure S83. ^{13}C NMR spectrum of compound **28**

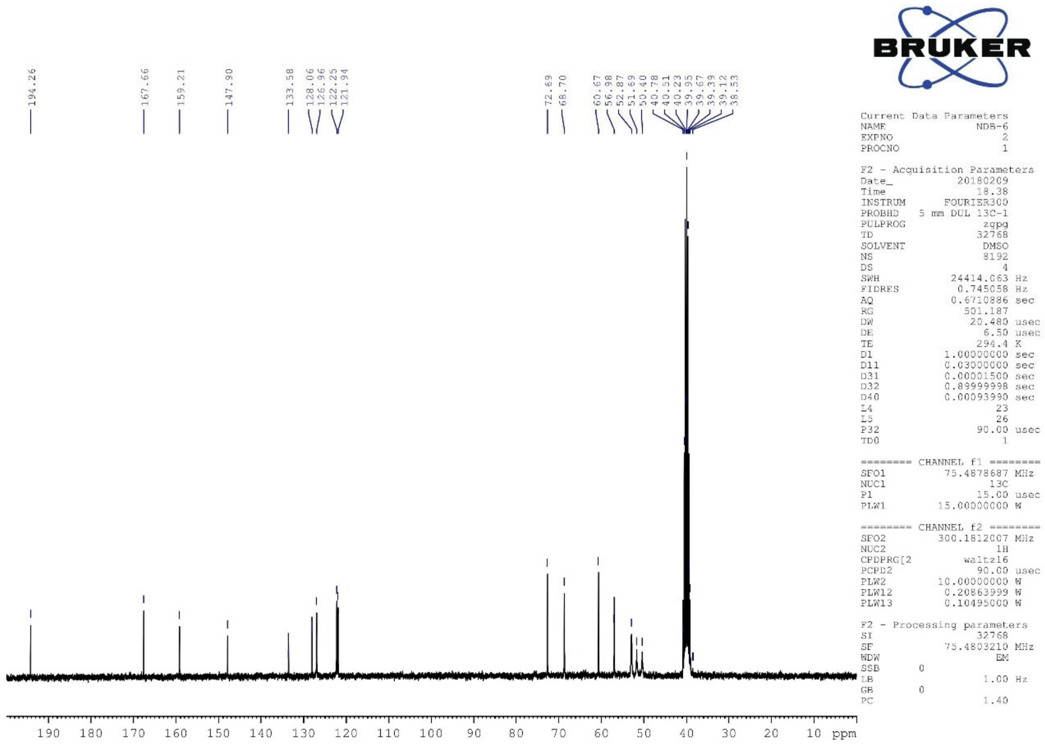

Figure S84. HRMS spectrum of compound **28**

Formula Predictor Report - NDB-6_36.lcd

Data File: C:\LabSolutions\Data\Analiz\mdaltintop\NDB-6_36.lcd

Elmt	Val.	Min	Max	Elmt	Val.	Min	Max	Elmt	Val.	Min	Max	Elmt	Val.	Min	Max	Use Adduct
H	1	5	40	O	2	0	5	S	2	0	3	Ru	2	0	0	H
C	4	0	35	F	1	0	0	Cl	1	0	1	I	3	0	0	
N	3	3	5	P	3	0	0	Br	1	0	0					

Error Margin (ppm): 5
HC Ratio: unlimited
Max Isotopes: 3
MSn Iso RI (%): 10.00

DBE Range: 5.0 - 10.0
Apply N Rule: yes
Isotope RI (%): 1.00
MSn Logic Mode: AND

Electron Ions: both
Use MSn Info: yes
Isotope Res: 9000
Max Results: 500

Event#: 1 MS(E+) Ret. Time : 5.360 -> 5.933 Scan# : 805 -> 891

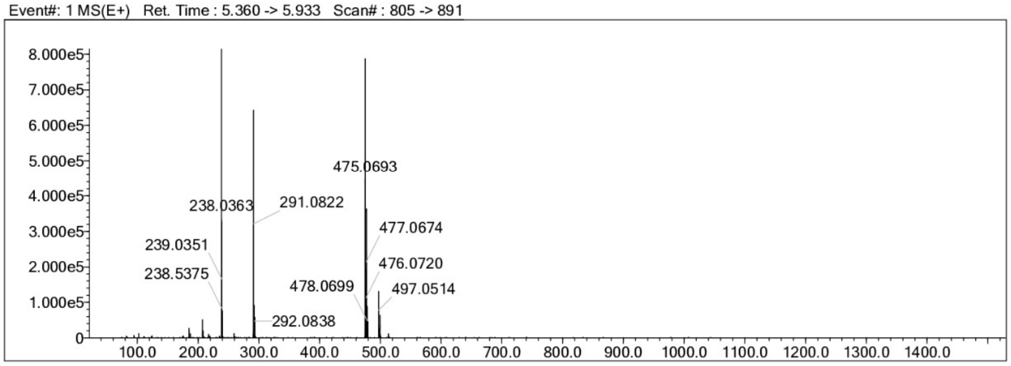

Measured region for 475.0693 m/z

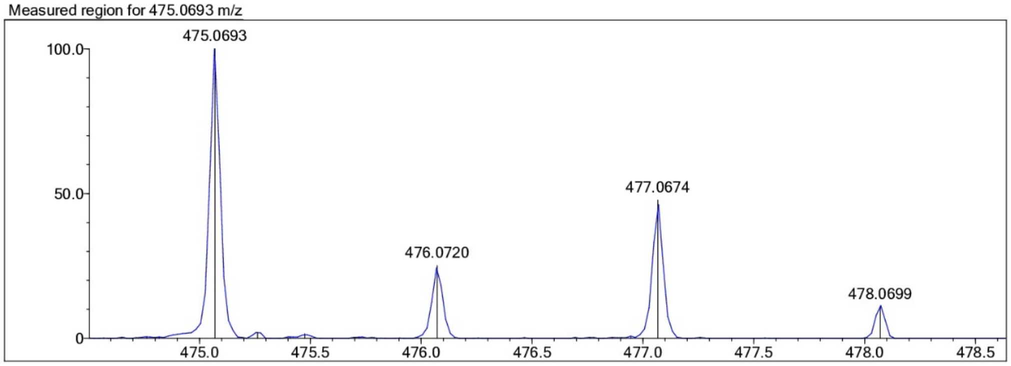

C18 H23 N4 O3 S3 Cl [M+H]+ : Predicted region for 475.0694 m/z

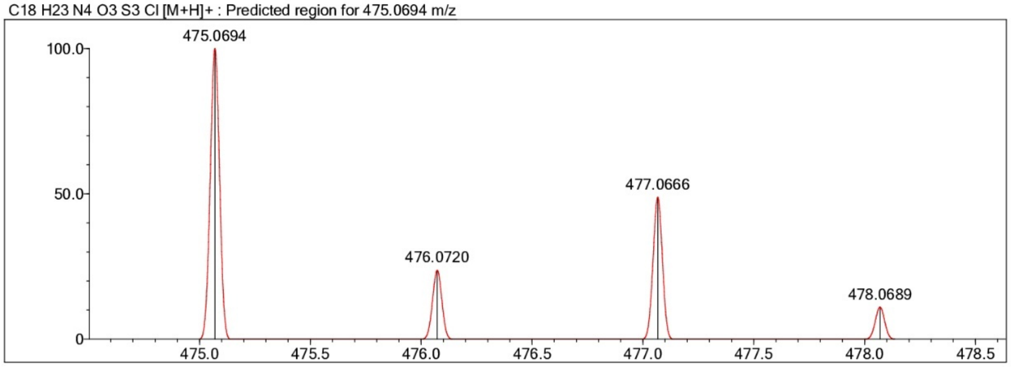

Rank	Score	Formula (M)	Ion	Meas. m/z	Pred. m/z	Df. (mDa)	Df. (ppm)	Iso	DBE
1	94.08	C18 H23 N4 O3 S3 Cl	[M+H]+	475.0693	475.0694	-0.1	-0.21	94.08	9.0

Figure S85. ^{1}H NMR spectrum of compound **29**

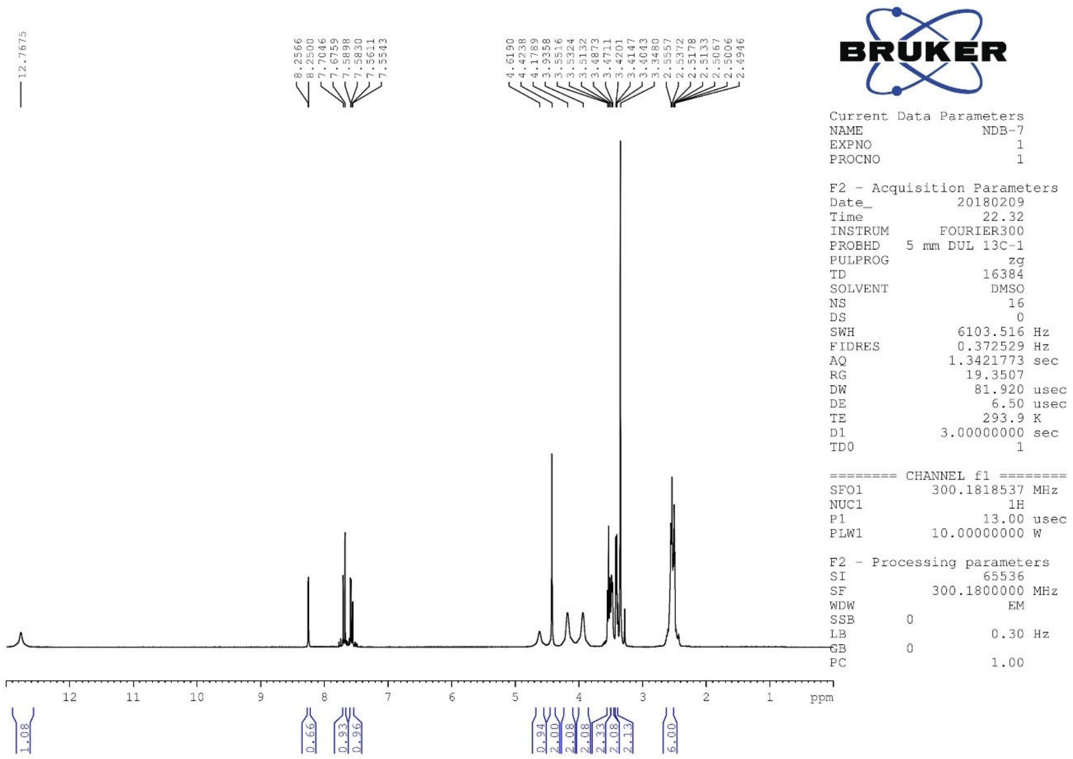

Figure S86. ^{13}C NMR spectrum of compound **29**

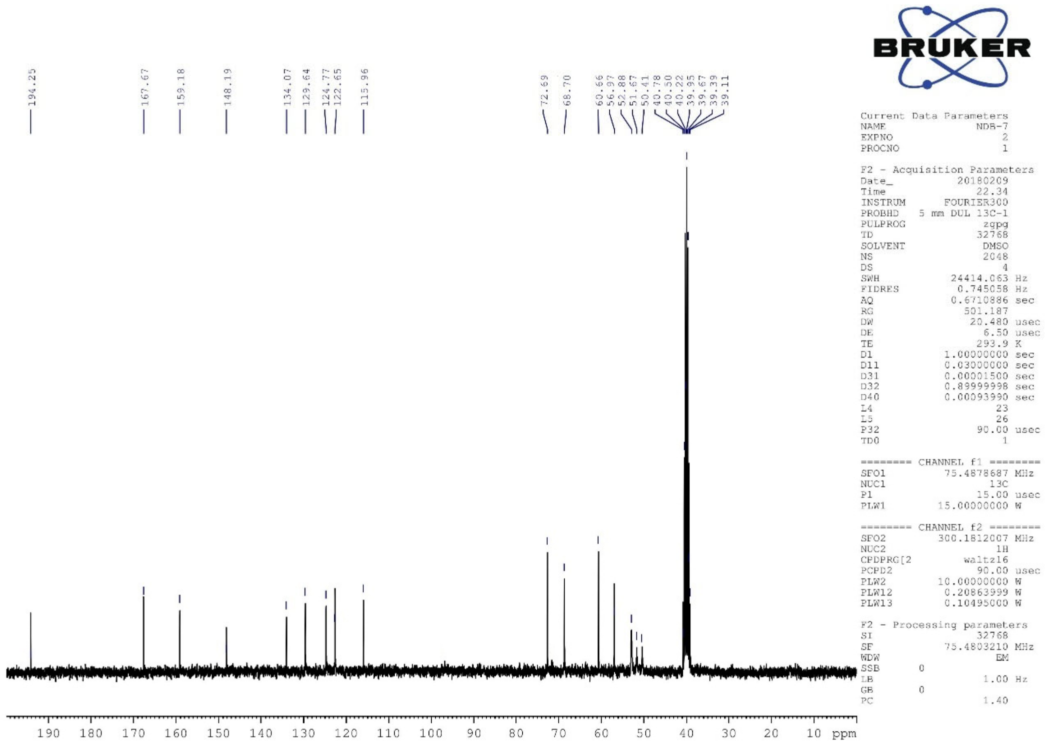

Figure S87. HRMS spectrum of compound **29**

Data File: C:\LabSolutions\Data\Analiz\mdaltintop\NDB-7_29.lcd

Elmt	Val.	Min	Max	Elmt	Val.	Min	Max	Elmt	Val.	Min	Max	Elmt	Val.	Min	Max	Use Adduct
H	1	5	40	O	2	0	5	S	2	0	3	Ru	2	0	0	H
C	4	0	35	F	1	0	0	Cl	1	0	0	I	3	0	0	
N	3	3	6	P	3	0	0	Br	1	0	1					

Error Margin (ppm):	5	DBE Range:	5.0 - 15.0	Electron Ions:	both
HC Ratio:	unlimited	Apply N Rule:	yes	Use MSn Info:	yes
Max Isotopes:	3	Isotope RI (%):	1.00	Isotope Res:	9000
MSn Iso RI (%):	10.00	MSn Logic Mode:	AND	Max Results:	500

Event#: 1 MS(E+) Ret. Time : 5.480 -> 6.053 Scan# : 823 -> 909

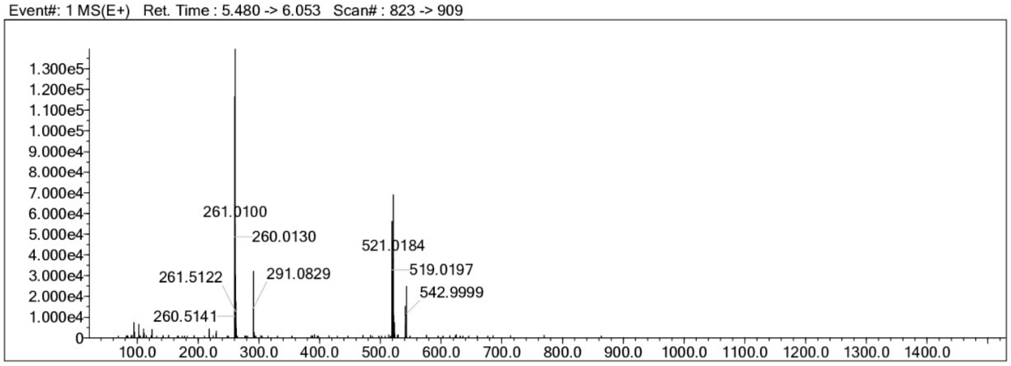

Measured region for 519.0197 m/z

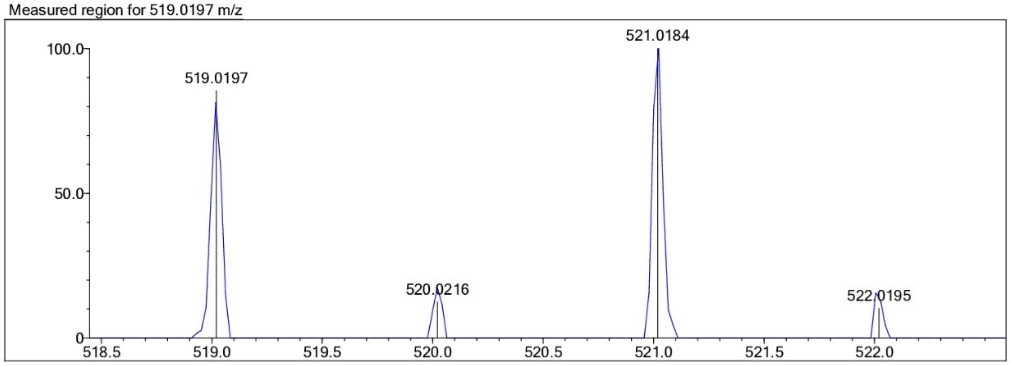

C18 H23 N4 O3 S3 Br [M+H]+ : Predicted region for 519.0188 m/z

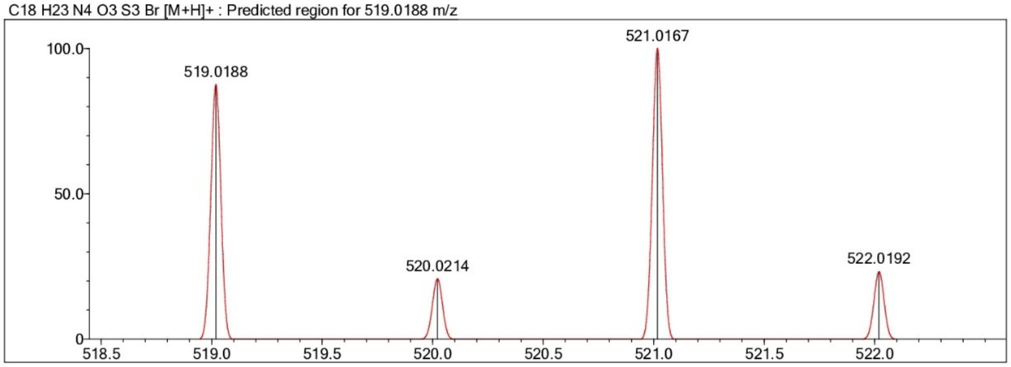

Rank	Score	Formula (M)	Ion	Meas. m/z	Pred. m/z	Df. (mDa)	Df. (ppm)	Iso	DBE
1	60.26	C18 H23 N4 O3 S3 Br	[M+H]+	519.0197	519.0188	0.9	1.73	61.38	9.0

Figure S88. ^{1}H NMR spectrum of compound **30**

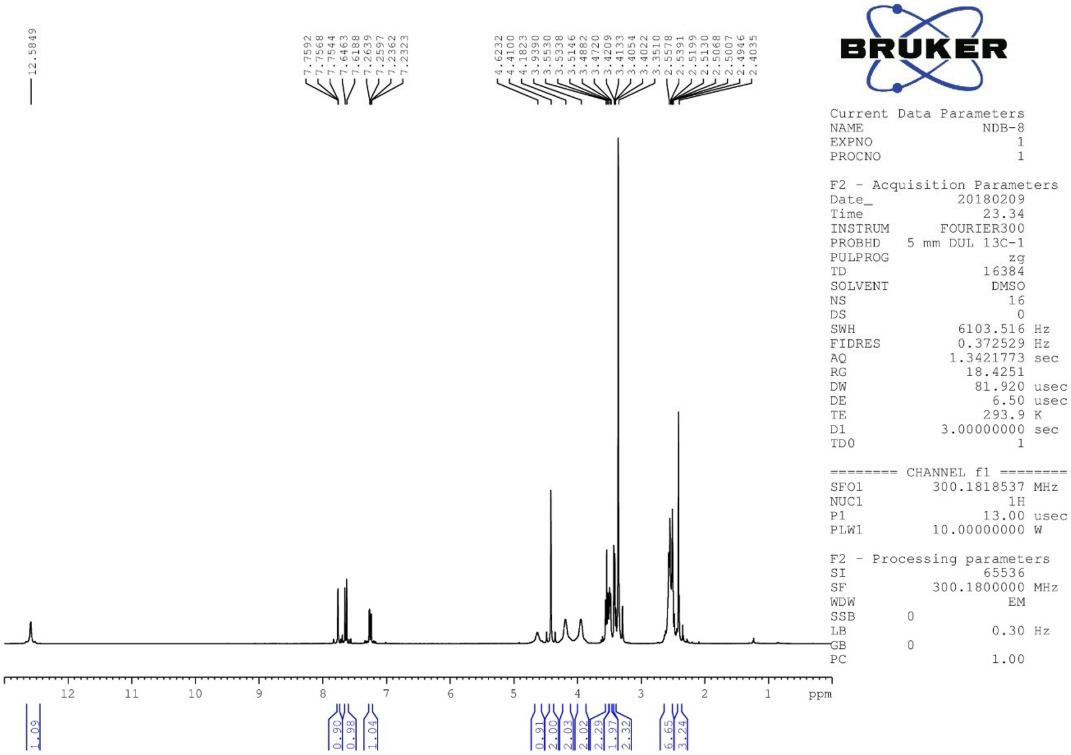

Figure S89. ^{13}C NMR spectrum of compound **30**

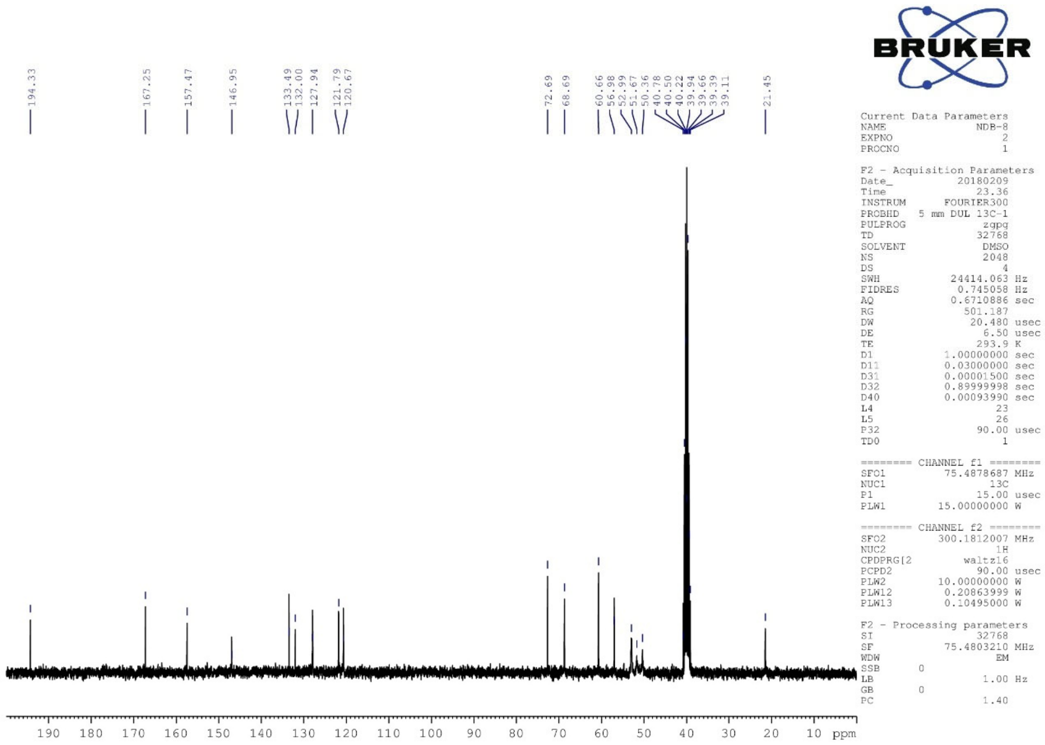

Figure S90. HRMS spectrum of compound **30**

Formula Predictor Report - NDB-8_30.lcd

Page 1 of 1

Data File: C:\LabSolutions\Data\Analiz\mdaltintop\NDB-8_30.lcd

Elmt	Val.	Min	Max	Elmt	Val.	Min	Max	Elmt	Val.	Min	Max	Elmt	Val.	Min	Max	Use Adduct
H	1	5	40	O	2	0	5	S	2	0	3	Ru	2	0	0	H
C	4	0	35	F	1	0	0	Cl	1	0	0	I	3	0	0	
N	3	3	6	P	3	0	0	Br	1	0	1					

Error Margin (ppm): 5
HC Ratio: unlimited
Max Isotopes: 3
MSn Iso RI (%): 10.00

DBE Range: 5.0 - 15.0
Apply N Rule: yes
Isotope RI (%): 1.00
MSn Logic Mode: AND

Electron Ions: both
Use MSn Info: yes
Isotope Res: 9000
Max Results: 500

Event#: 1 MS(E+) Ret. Time : 5.187 -> 5.707 Scan# : 779 -> 857

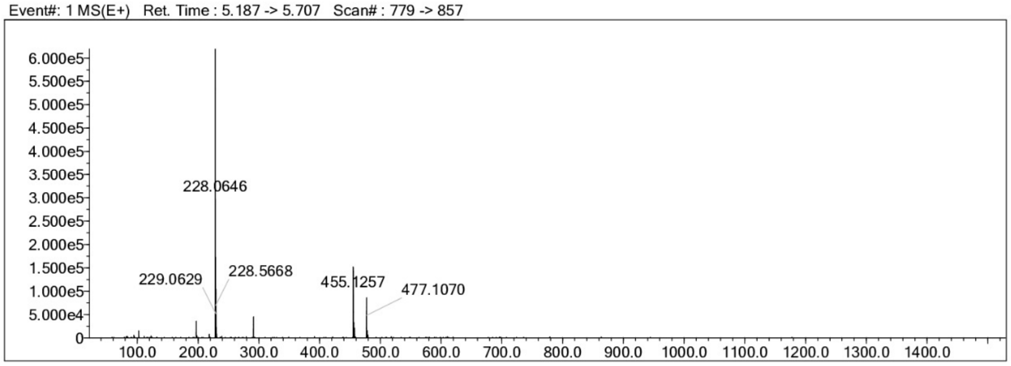

Measured region for 455.1257 m/z

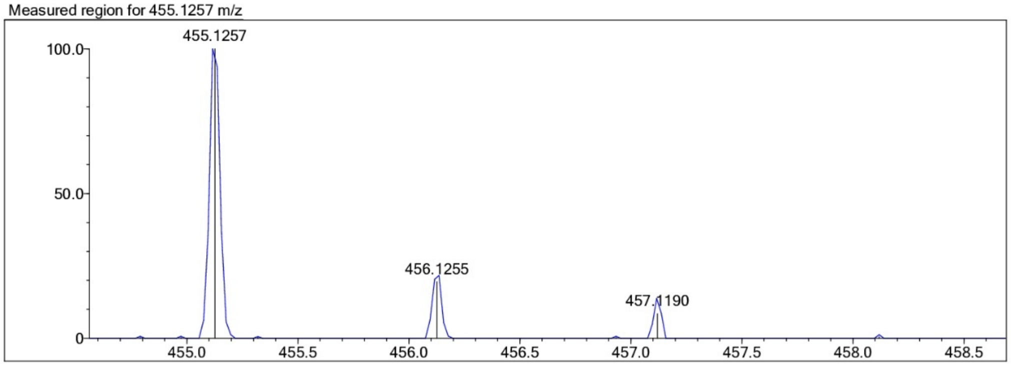

C19 H26 N4 O3 S3 [M+H]+ : Predicted region for 455.1240 m/z

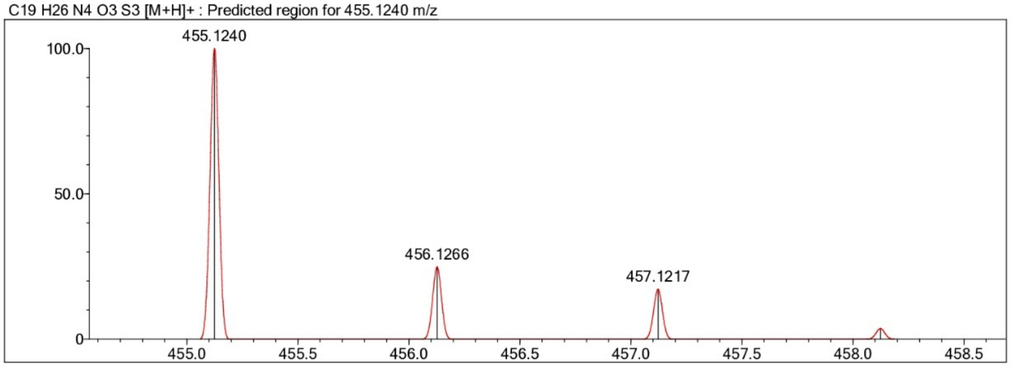

Rank	Score	Formula (M)	Ion	Meas. m/z	Pred. m/z	Df. (mDa)	Df. (ppm)	Iso	DBE
1	71.24	C19 H26 N4 O3 S3	[M+H]+	455.1257	455.1240	1.7	3.74	76.48	9.0

Figure S91. ^{1}H NMR spectrum of compound **31**

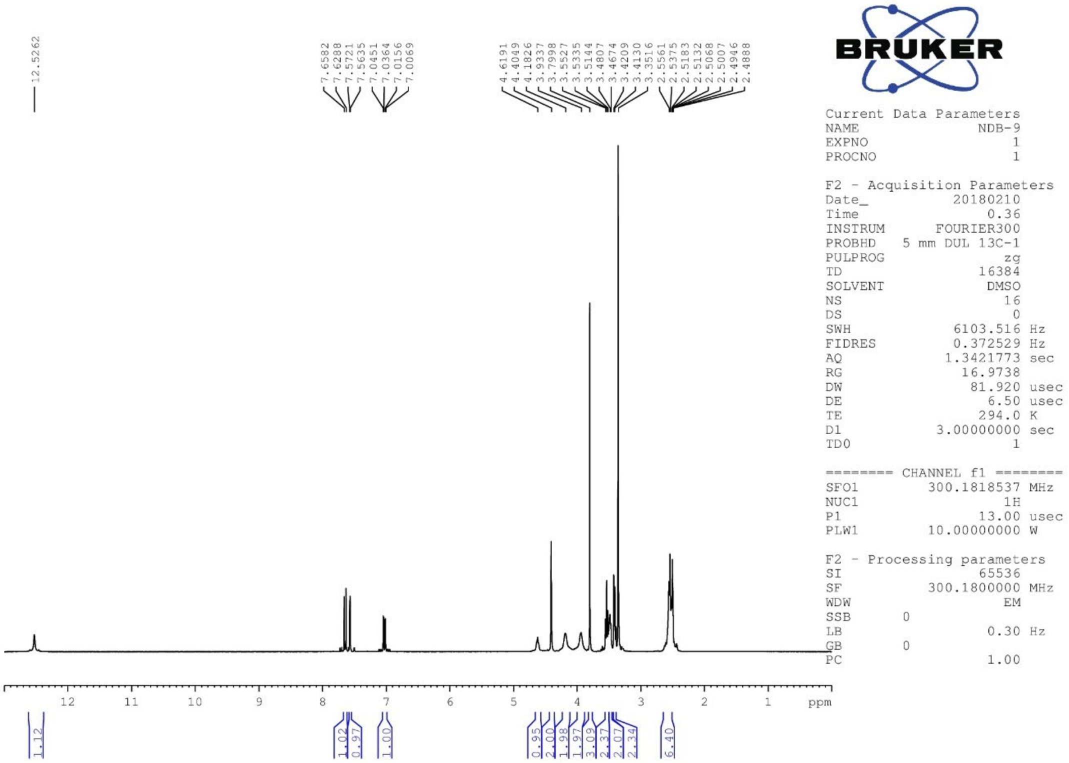

Figure S92. ^{13}C NMR spectrum of compound **31**

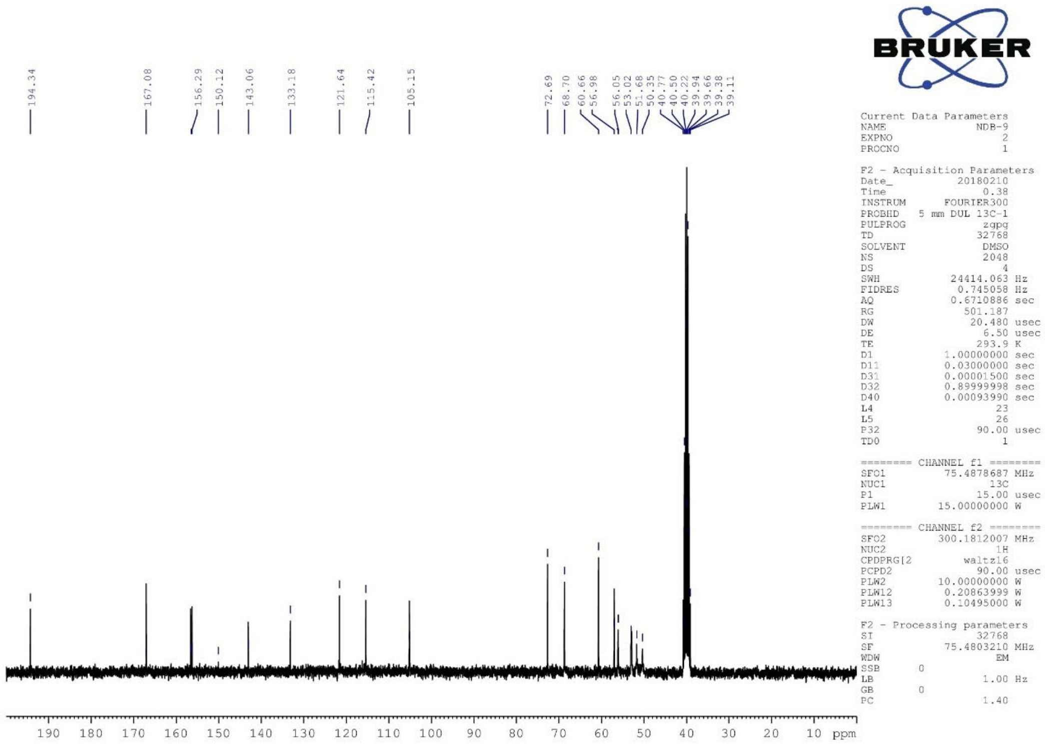

Figure S93. HRMS spectrum of compound **31**

Formula Predictor Report - NDB-9_37.lcd

Page 1 of 1

Data File: C:\LabSolutions\Data\Analiz\mdaltintop\NDB-9_37.lcd

Elmt	Val.	Min	Max	Elmt	Val.	Min	Max	Elmt	Val.	Min	Max	Elmt	Val.	Min	Max	Use Adduct
H	1	5	40	O	2	0	5	S	2	0	3	Ru	2	0	0	H
C	4	0	35	F	1	0	0	Cl	1	0	1	I	3	0	0	
N	3	3	5	P	3	0	0	Br	1	0	0					

Error Margin (ppm): 5
HC Ratio: unlimited
Max Isotopes: 3
MSn Iso RI (%): 10.00

DBE Range: 5.0 - 10.0
Apply N Rule: yes
Isotope RI (%): 1.00
MSn Logic Mode: AND

Electron Ions: both
Use MSn Info: yes
Isotope Res: 9000
Max Results: 500

Event#: 1 MS(E+) Ret. Time : 4.893 -> 5.387 Scan# : 735 -> 809

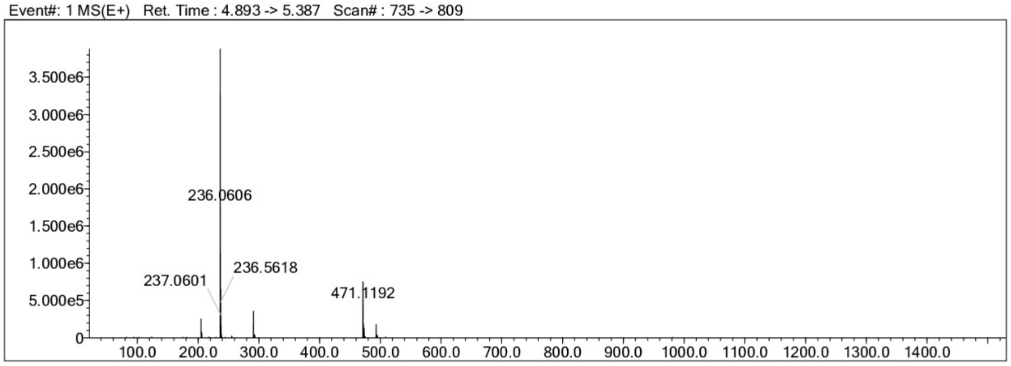

Measured region for 471.1192 m/z

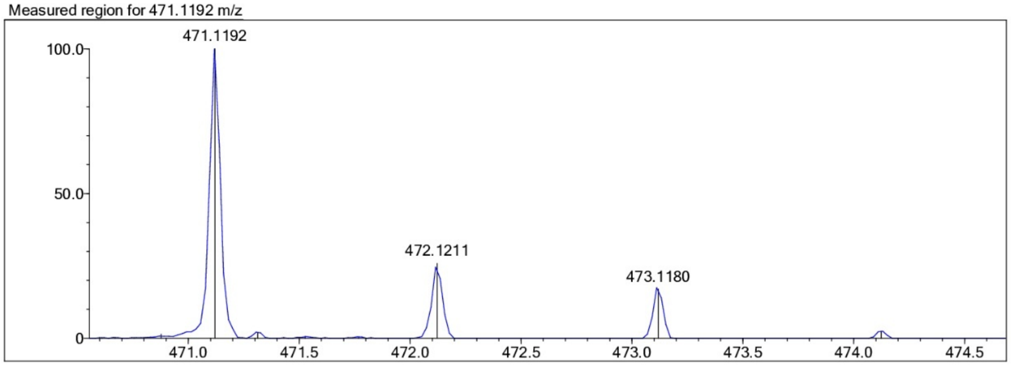

C19 H26 N4 O4 S3 [M+H]+ : Predicted region for 471.1189 m/z

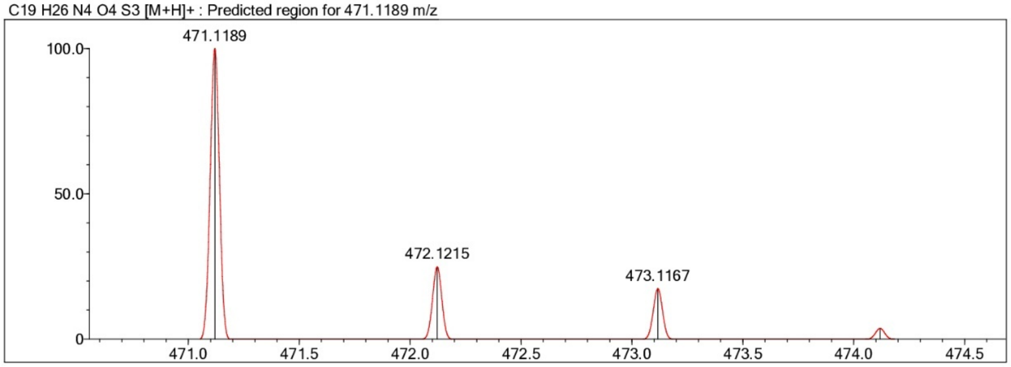

Rank	Score	Formula (M)	Ion	Meas. m/z	Pred. m/z	Df. (mDa)	Df. (ppm)	Iso	DBE
1	100.00	C19 H26 N4 O4 S3	[M+H]+	471.1192	471.1189	0.3	0.64	100.00	9.0

Figure S94. ^{1}H NMR spectrum of compound **32**

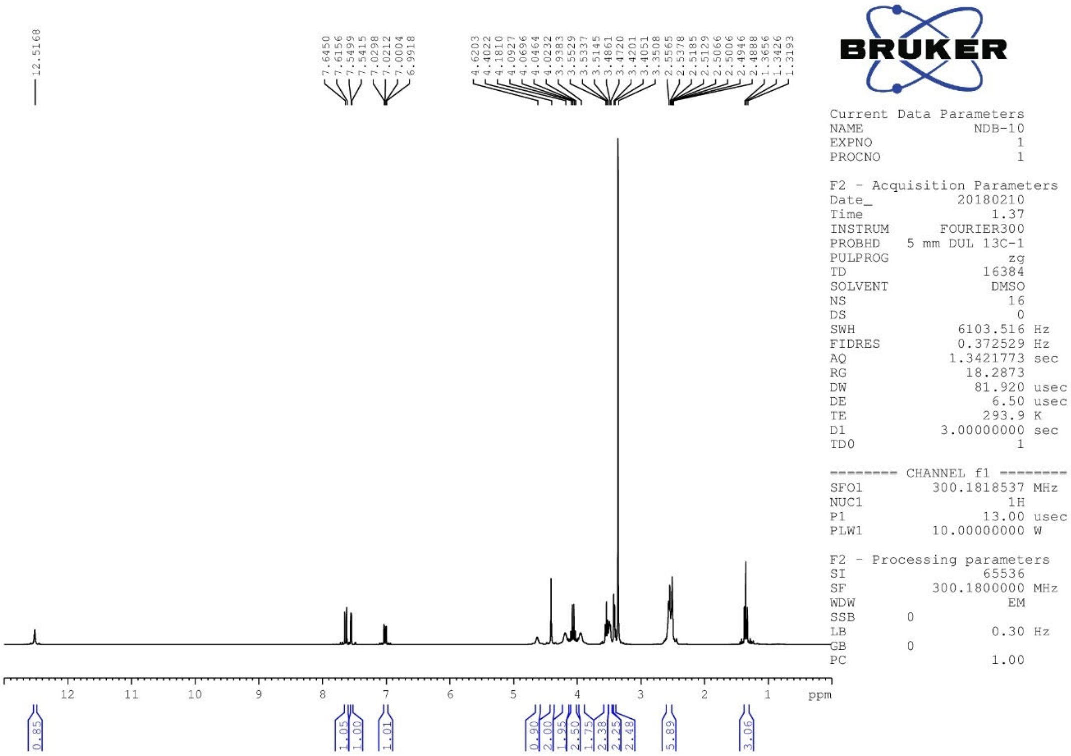

Figure S95. ^{13}C NMR spectrum of compound **32**

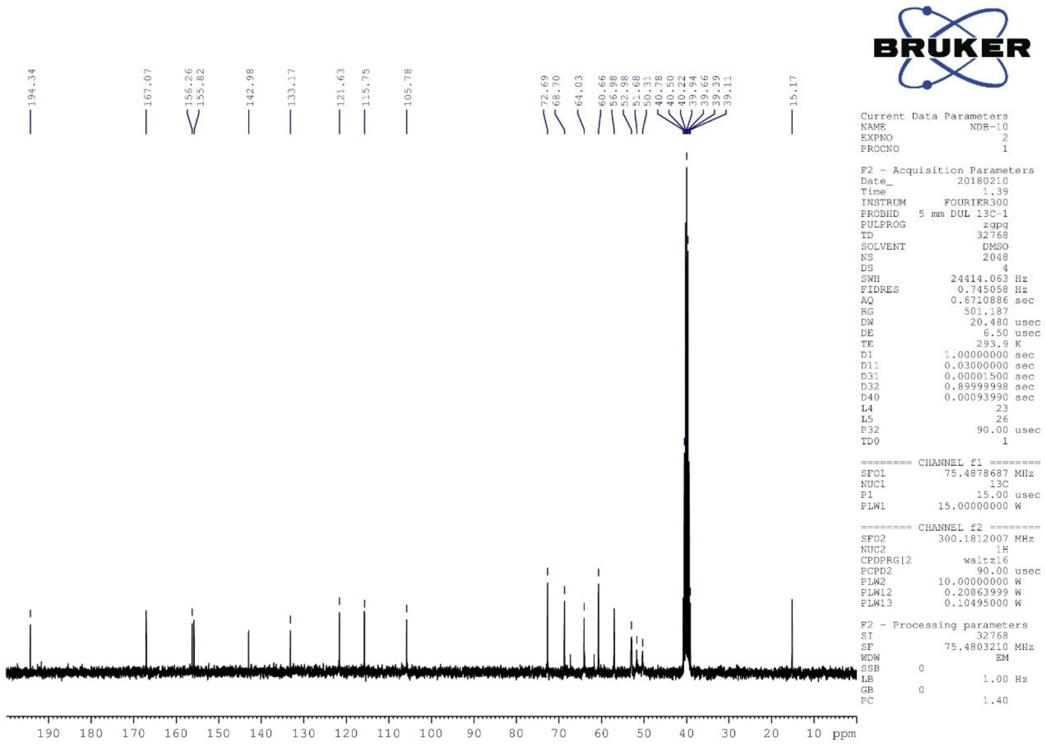

Figure S96. HRMS spectrum of compound **32**

Formula Predictor Report - NDB-10_31.lcd Page 1 of 1

Data File: C:\LabSolutions\Data\Analiz\mdaltintop\NDB-10_31.lcd

Elmt	Val.	Min	Max	Elmt	Val.	Min	Max	Elmt	Val.	Min	Max	Elmt	Val.	Min	Max	Use Adduct
H	1	5	40	O	2	0	5	S	2	0	3	Ru	2	0	0	H
C	4	0	35	F	1	0	0	Cl	1	0	0	I	3	0	0	
N	3	3	6	P	3	0	0	Br	1	0	0					

Error Margin (ppm):	5	DBE Range:	5.0 - 16.0	Electron Ions:	both
HC Ratio:	unlimited	Apply N Rule:	yes	Use MSn Info:	yes
Max Isotopes:	3	Isotope RI (%):	1.00	Isotope Res:	9000
MSn Iso RI (%):	10.00	MSn Logic Mode:	AND	Max Results:	500

Event#: 1 MS(E+) Ret. Time : 5.453 Scan# : 819

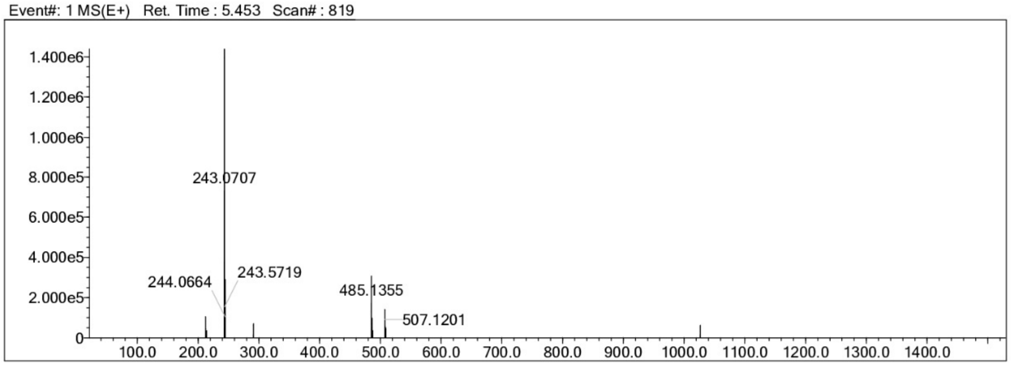

Measured region for 485.1355 m/z

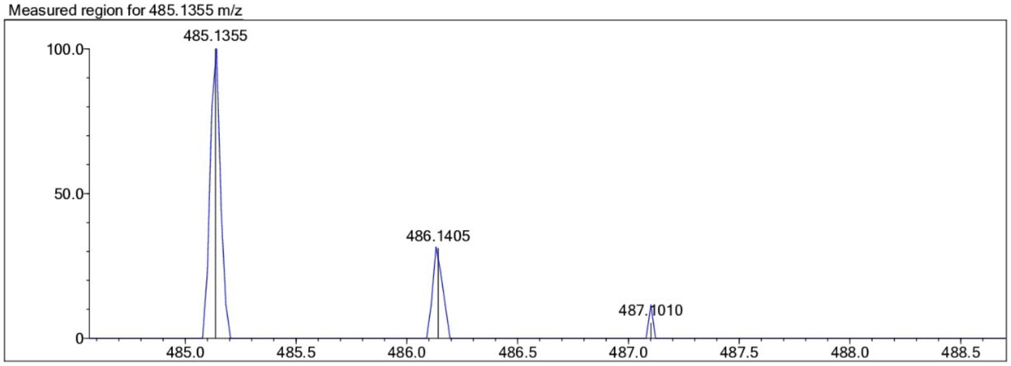

C20 H28 N4 O4 S3 [M+H]+ : Predicted region for 485.1345 m/z

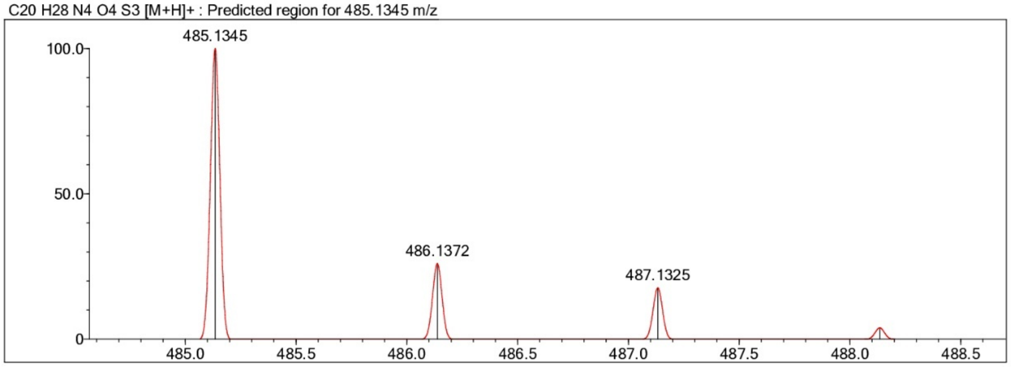

Rank	Score	Formula (M)	Ion	Meas. m/z	Pred. m/z	Df. (mDa)	Df. (ppm)	Iso	DBE
1	0.00	C20 H28 N4 O4 S3	[M+H]+	485.1355	485.1345	1.0	2.06	0.00	9.0

Figure S97. ^{1}H NMR spectrum of compound **33**

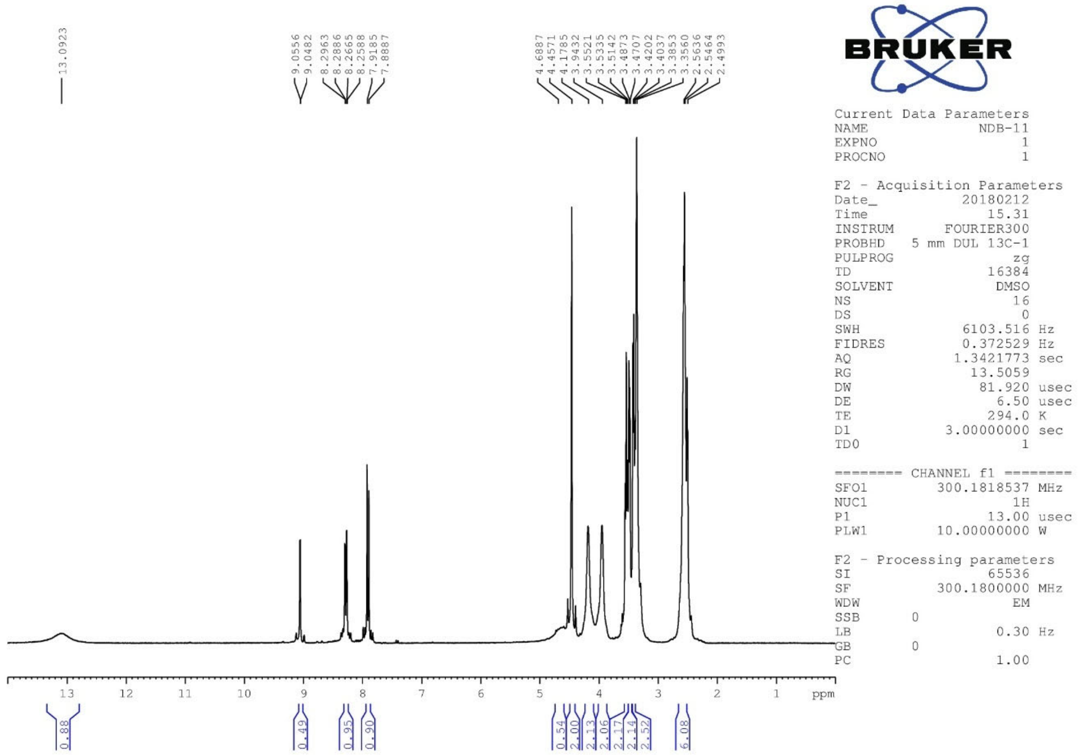

Figure S98. ^{13}C NMR spectrum of compound **33**

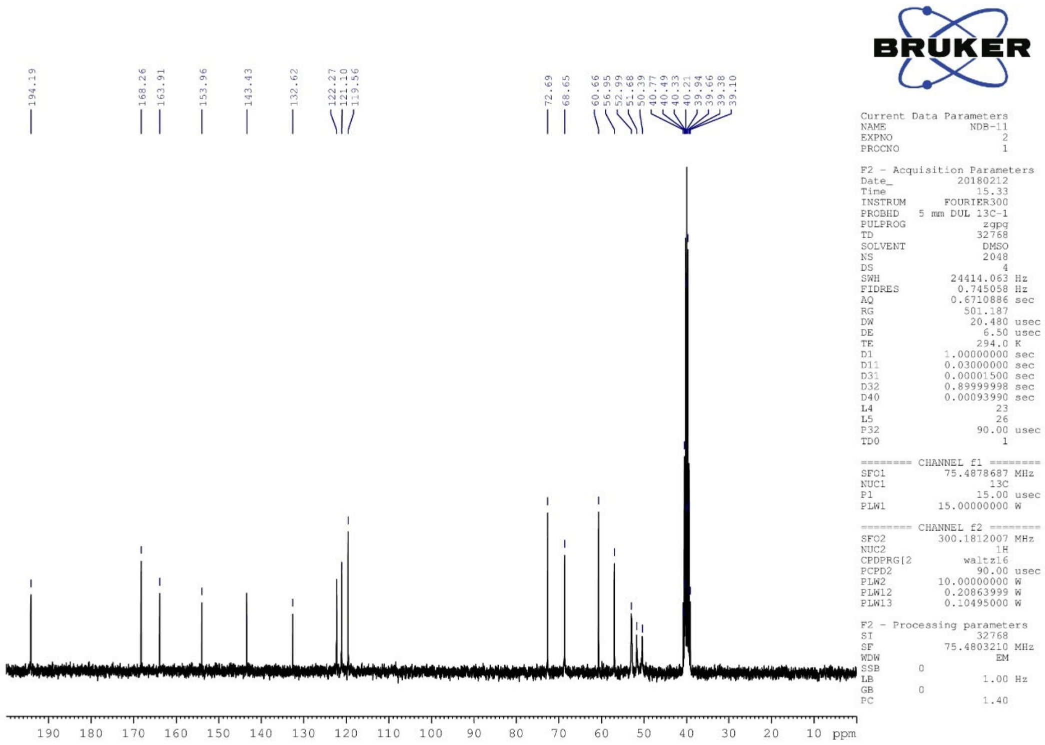

Figure S99. HRMS spectrum of compound **33**

Data File: C:\LabSolutions\Data\Analiz\mdaltintop\NDB-11_32.lcd

Elmt	Val.	Min	Max	Elmt	Val.	Min	Max	Elmt	Val.	Min	Max	Elmt	Val.	Min	Max	Use Adduct
H	1	5	40	O	2	0	5	S	2	0	3	Ru	2	0	0	H
C	4	0	35	F	1	0	0	Cl	1	0	0	I	3	0	0	
N	3	3	6	P	3	0	0	Br	1	0	0					

Error Margin (ppm): 5
HC Ratio: unlimited
Max Isotopes: 3
MSn Iso RI (%): 10.00

DBE Range: 5.0 - 16.0
Apply N Rule: yes
Isotope RI (%): 1.00
MSn Logic Mode: AND

Electron Ions: both
Use MSn Info: yes
Isotope Res: 9000
Max Results: 500

Event#: 1 MS(E+) Ret. Time : 5.093 -> 5.520 Scan# : 765 -> 829

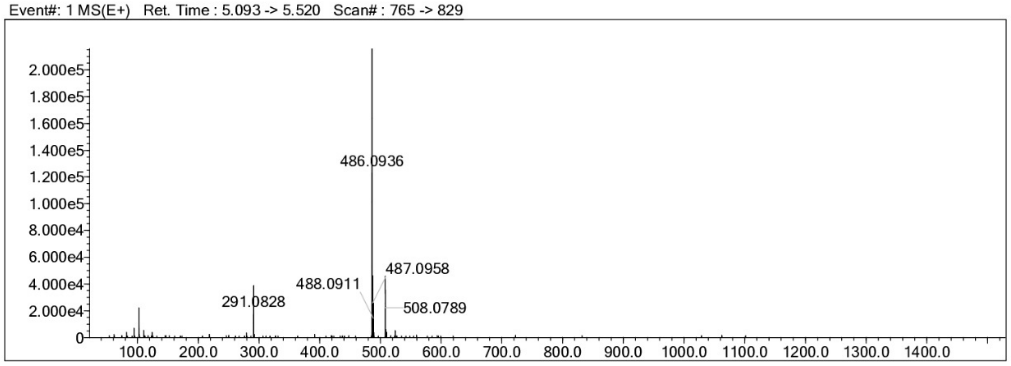

Measured region for 486.0936 m/z

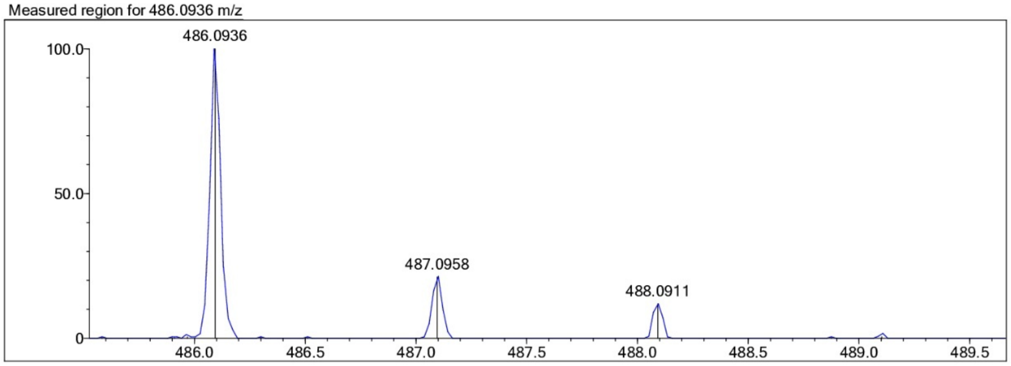

C18 H23 N5 O5 S3 [M+H]+ : Predicted region for 486.0934 m/z

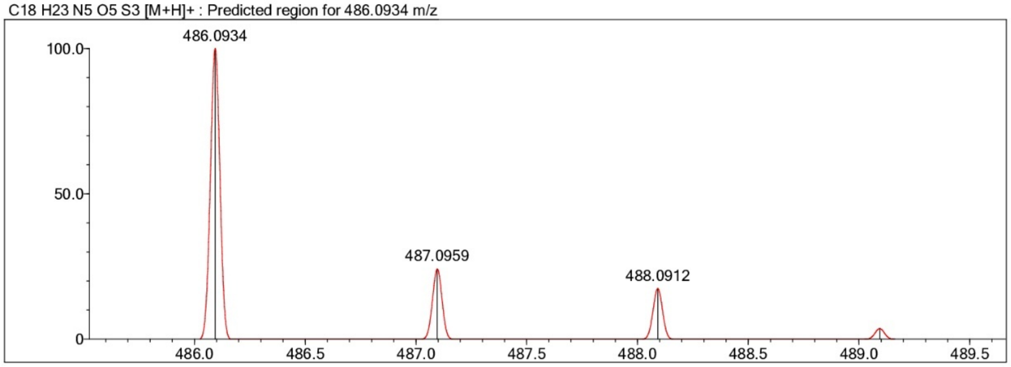

Rank	Score	Formula (M)	Ion	Meas. m/z	Pred. m/z	Df. (mDa)	Df. (ppm)	Iso	DBE
1	64.22	C18 H23 N5 O5 S3	[M+H]+	486.0936	486.0934	0.2	0.41	64.22	10.0

Figure S100. ^{1}H NMR spectrum of compound **34**

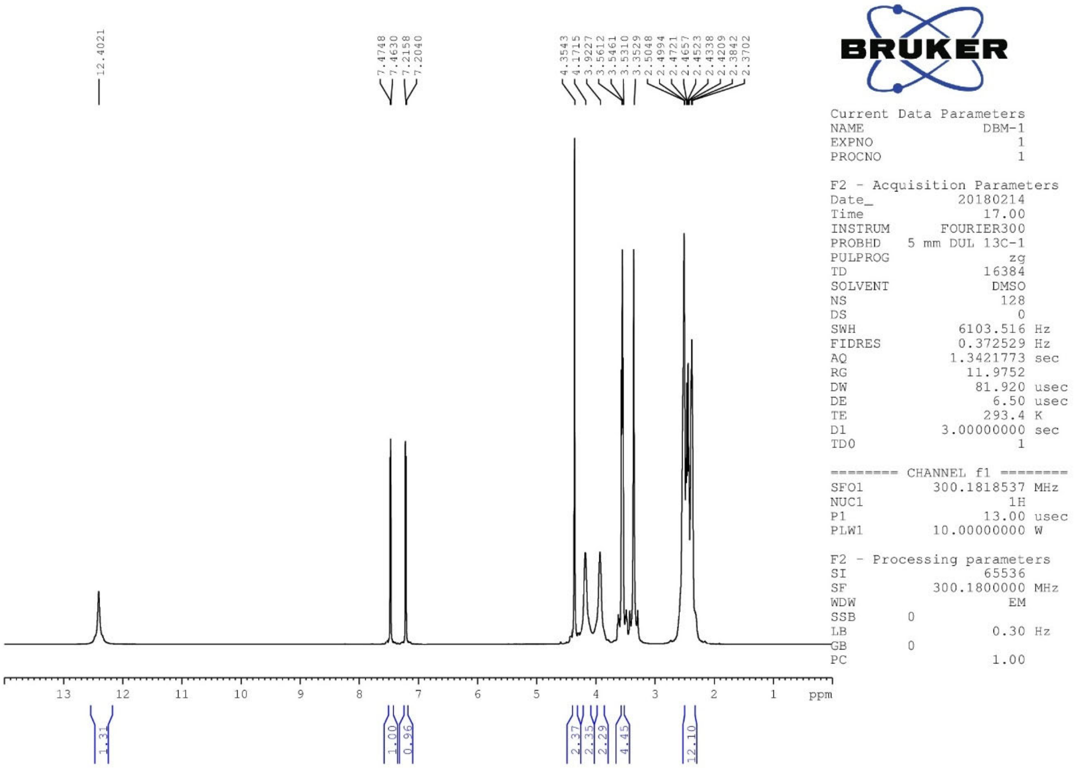

Figure S101. ^{13}C NMR spectrum of compound **34**

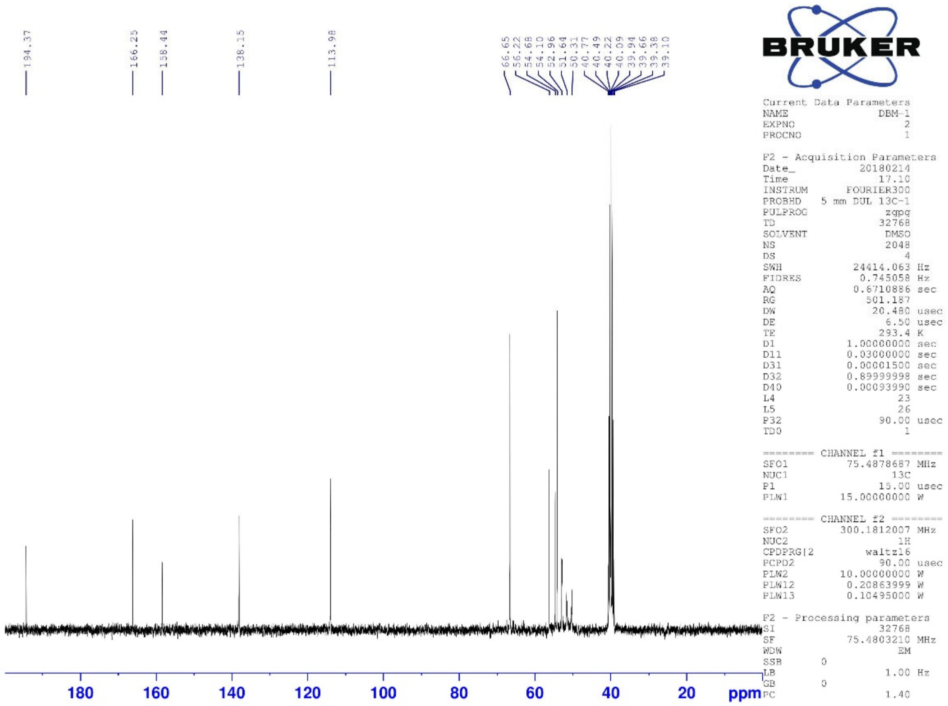

Figure S102. HRMS spectrum of compound **34**

Formula Predictor Report - DBM-1_34.lcd

Data File: C:\LabSolutions\Data\Analiz\mdaltintop\DBM-1_34.lcd

Elmt	Val.	Min	Max	Elmt	Val.	Min	Max	Elmt	Val.	Min	Max	Elmt	Val.	Min	Max	Use Adduct
H	1	5	40	O	2	0	5	S	2	0	3	Ru	2	0	0	H
C	4	0	35	F	1	0	0	Cl	1	0	0	I	3	0	0	
N	3	3	6	P	3	0	0	Br	1	0	0					

Error Margin (ppm):	5	DBE Range:	5.0 - 11.0	Electron Ions:	both
HC Ratio:	unlimited	Apply N Rule:	yes	Use MSn Info:	yes
Max Isotopes:	3	Isotope RI (%):	1.00	Isotope Res:	9000
MSn Iso RI (%):	10.00	MSn Logic Mode:	AND	Max Results:	500

Event#: 1 MS(E+) Ret. Time : 4.133 -> 4.293 Scan# : 621 -> 645

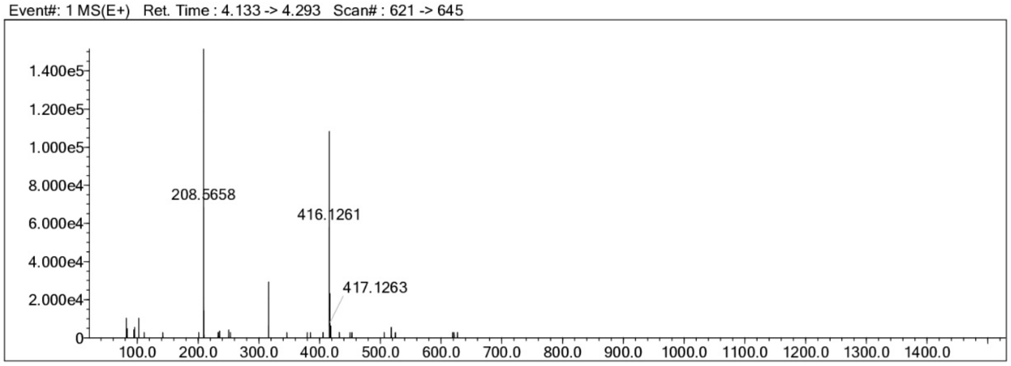

Measured region for 416.1261 m/z

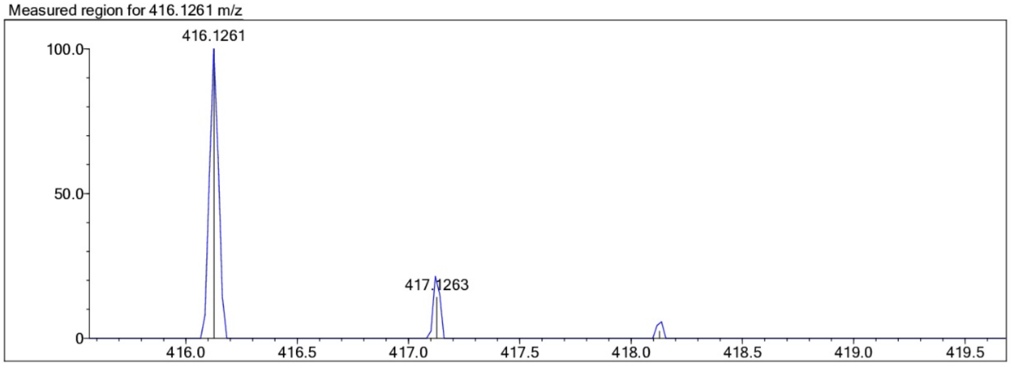

C16 H25 N5 O2 S3 [M+H]+ : Predicted region for 416.1243 m/z

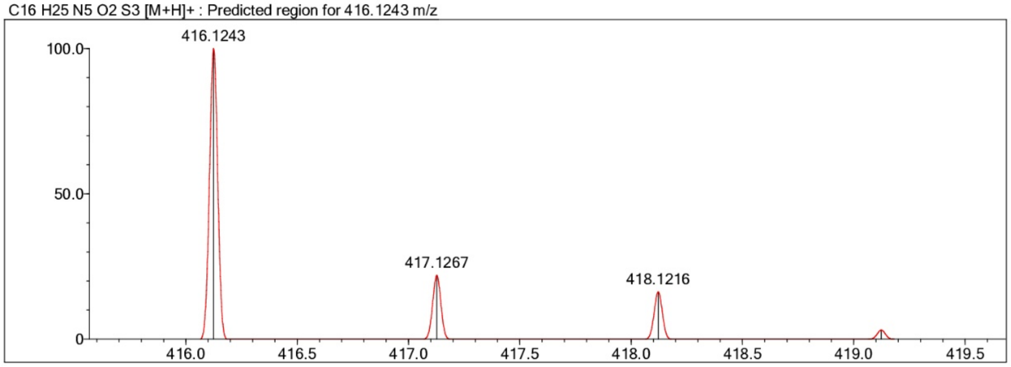

Rank	Score	Formula (M)	Ion	Meas. m/z	Pred. m/z	Df. (mDa)	Df. (ppm)	Iso	DBE
1	46.91	C16 H25 N5 O2 S3	[M+H]+	416.1261	416.1243	1.8	4.33	51.17	7.0

Figure S103. ^{1}H NMR spectrum of compound **35**

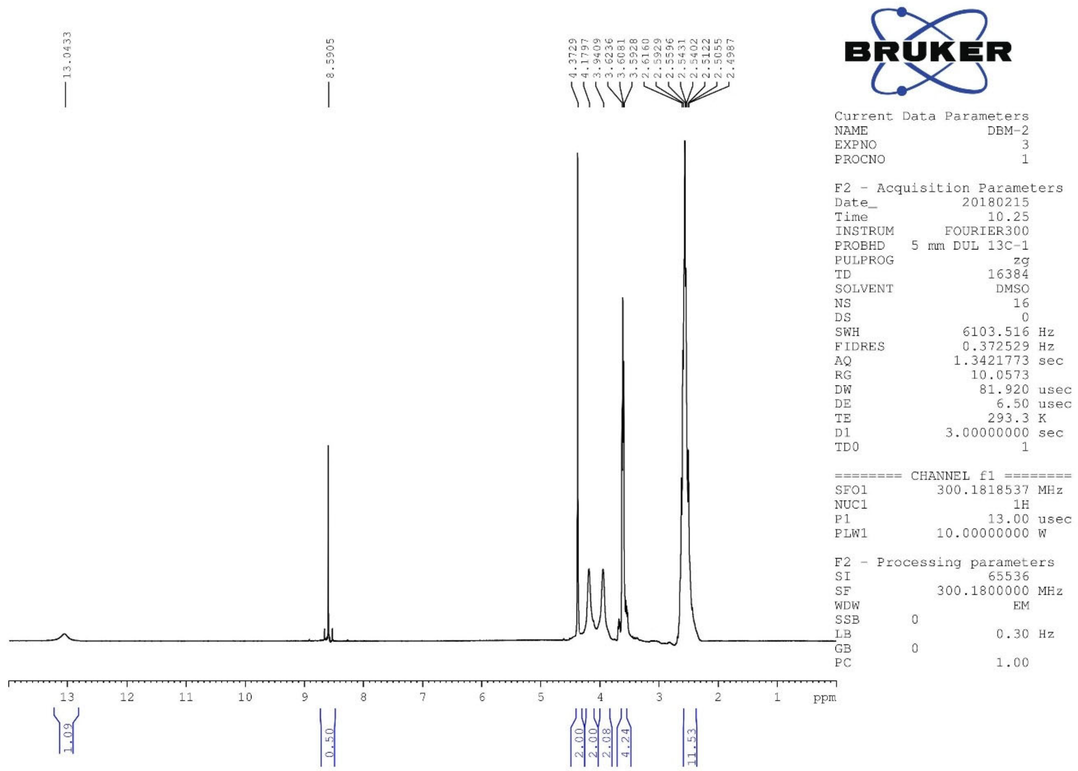

Figure S104. ^{13}C NMR spectrum of compound **35**

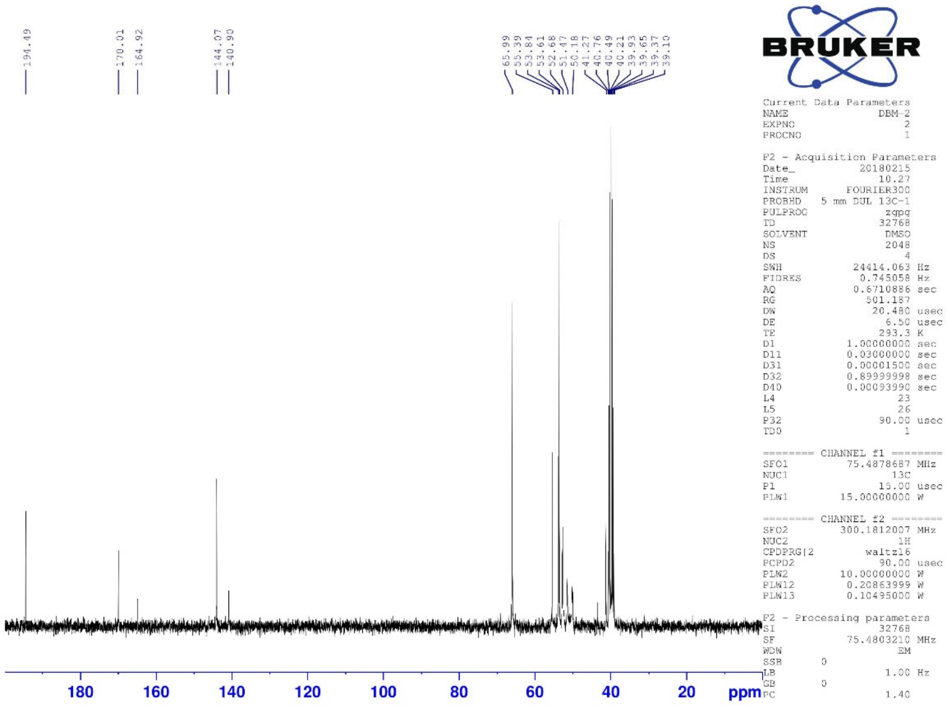

Figure S105. HRMS spectrum of compound **35**

Formula Predictor Report - DBM-2_39.lcd　　　Page 1 of 1

Data File: C:\LabSolutions\Data\Analiz\mdaltintop\DBM-2_39.lcd

Elmt	Val.	Min	Max	Elmt	Val.	Min	Max	Elmt	Val.	Min	Max	Elmt	Val.	Min	Max	Use Adduct
H	1	5	40	O	2	0	5	S	2	0	3	Ru	2	0	0	H
C	4	0	35	F	1	0	0	Cl	1	0	0	I	3	0	0	
N	3	3	6	P	3	0	0	Br	1	0	0					

Error Margin (ppm):	5	DBE Range:	5.0 - 10.0	Electron Ions:	both
HC Ratio:	unlimited	Apply N Rule:	yes	Use MSn Info:	yes
Max Isotopes:	3	Isotope RI (%):	1.00	Isotope Res:	9000
MSn Iso RI (%):	10.00	MSn Logic Mode:	AND	Max Results:	500

Event#: 1 MS(E+)　Ret. Time : 5.107　Scan# : 767

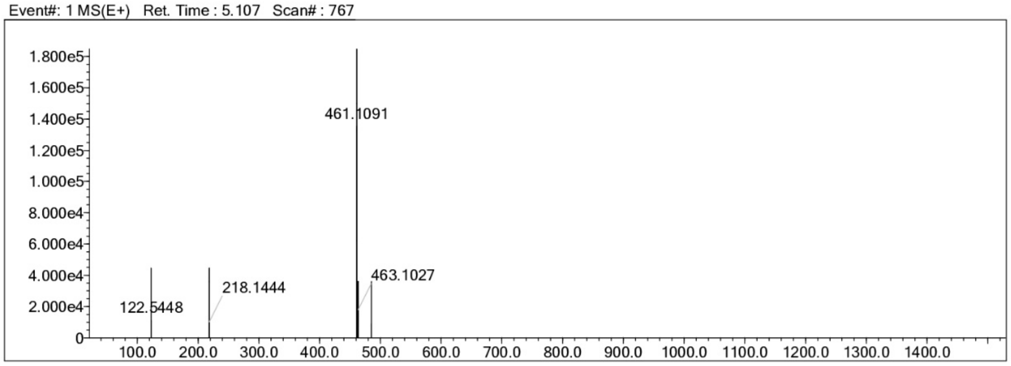

Measured region for 461.1091 m/z

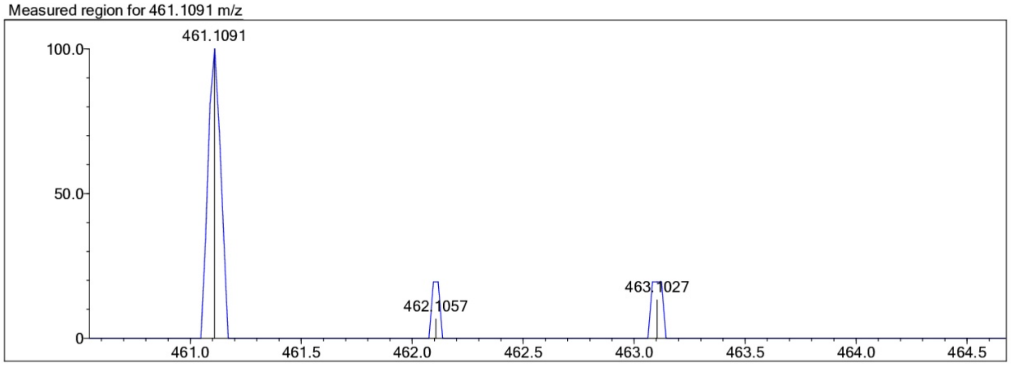

C16 H24 N6 O4 S3 [M+H]+ : Predicted region for 461.1094 m/z

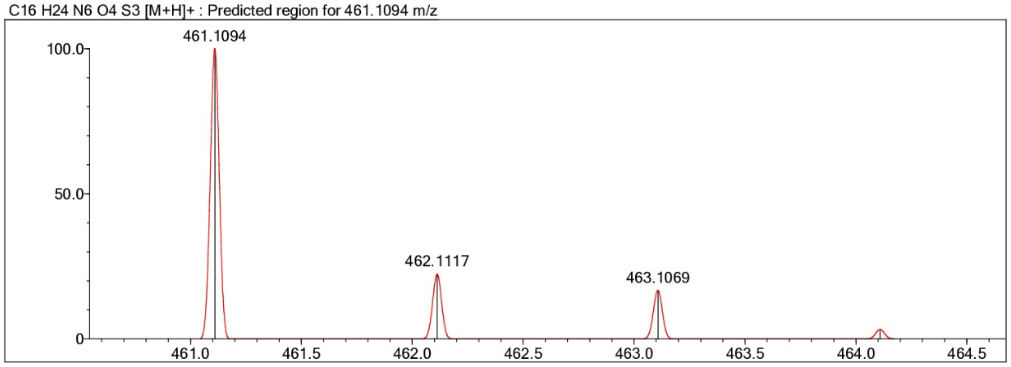

Rank	Score	Formula (M)	Ion	Meas. m/z	Pred. m/z	Df. (mDa)	Df. (ppm)	Iso	DBE
1	79.16	C16 H24 N6 O4 S3	[M+H]+	461.1091	461.1094	-0.3	-0.65	79.16	8.0

Figure S106. ^{1}H NMR spectrum of compound **36**

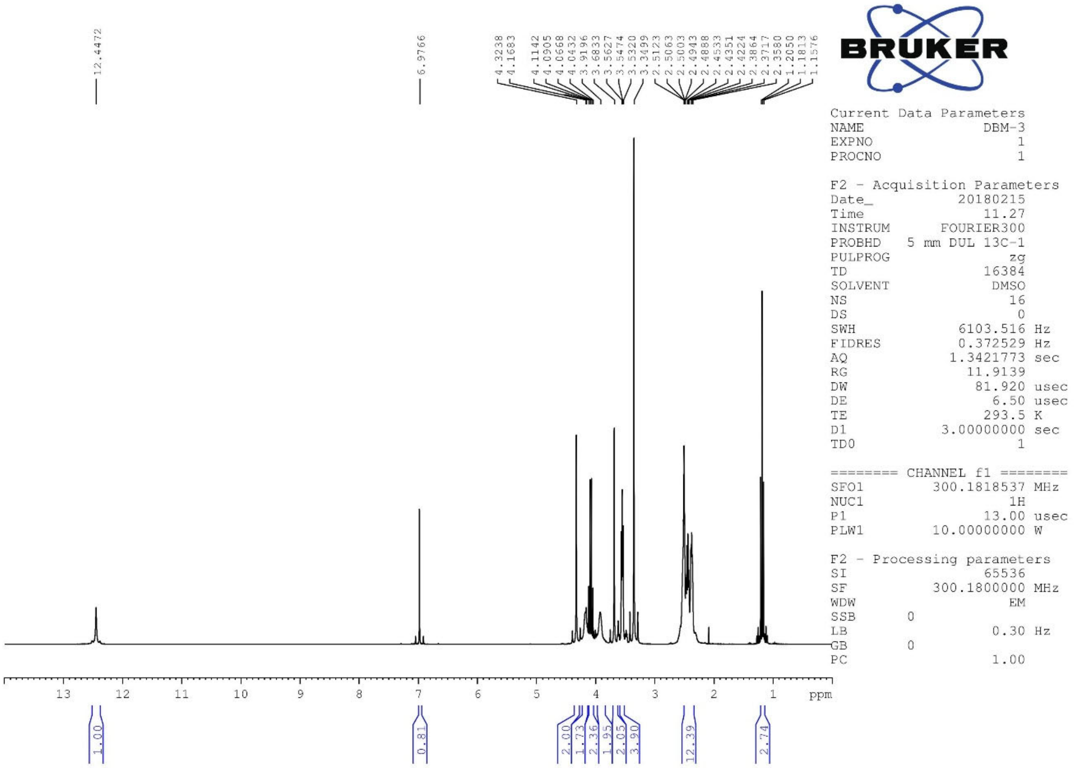

Figure S107. ^{13}C NMR spectrum of compound **36**

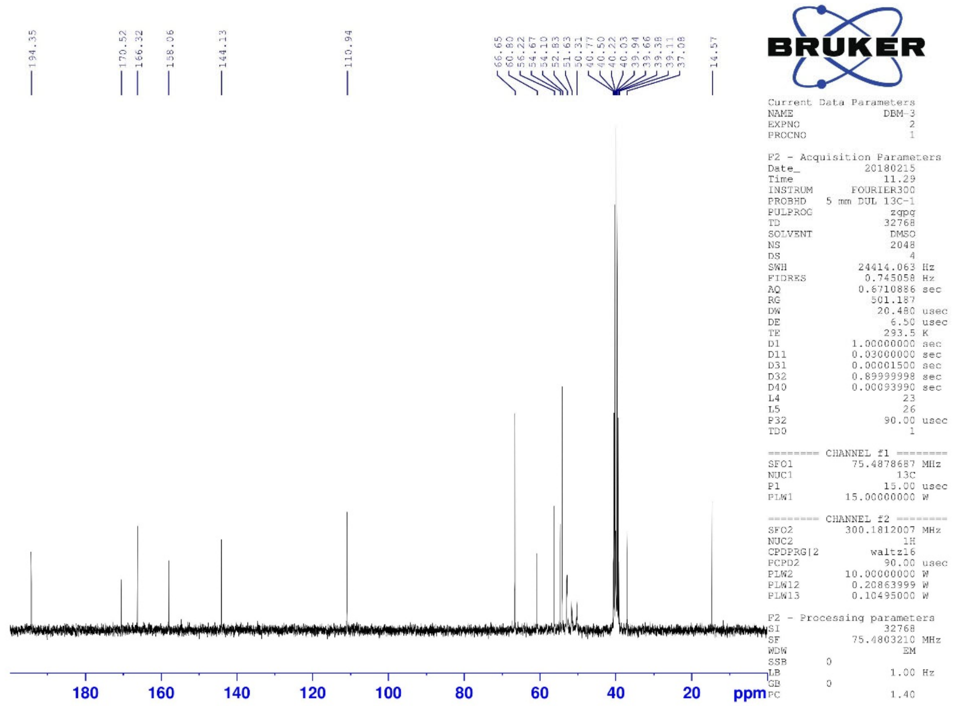

Figure S108. HRMS spectrum of compound **36**

Formula Predictor Report - DBM-3_35.lcd

Page 1 of 1

Data File: C:\LabSolutions\Data\Analiz\mdaltintop\DBM-3_35.lcd

Elmt	Val.	Min	Max	Elmt	Val.	Min	Max	Elmt	Val.	Min	Max	Elmt	Val.	Min	Max	Use Adduct
H	1	5	40	O	2	0	5	S	2	0	3	Ru	2	0	0	H
C	4	0	35	F	1	0	0	Cl	1	0	0	I	3	0	0	
N	3	3	6	P	3	0	0	Br	1	0	0					

Error Margin (ppm):	5	DBE Range:	5.0 - 15.0	Electron Ions:	both
HC Ratio:	unlimited	Apply N Rule:	yes	Use MSn Info:	yes
Max Isotopes:	3	Isotope RI (%):	1.00	Isotope Res:	9000
MSn Iso RI (%):	10.00	MSn Logic Mode:	AND	Max Results:	500

Event#: 1 MS(E+) Ret. Time : 4.840 -> 5.493 Scan# : 727 -> 825

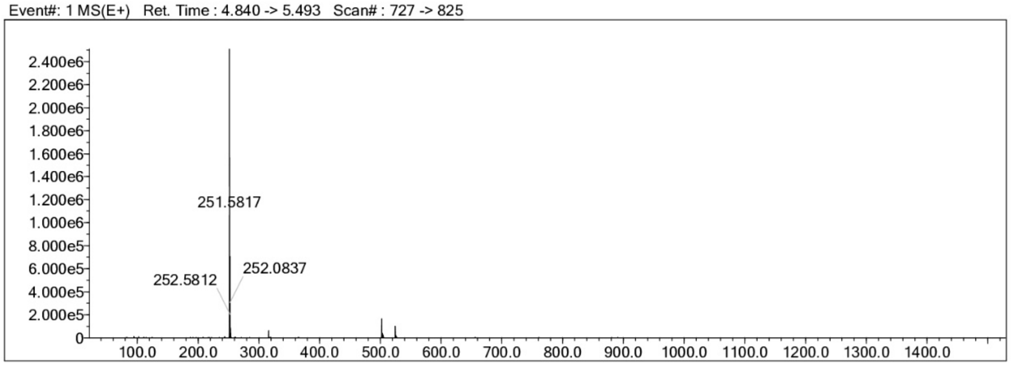

Measured region for 502.1621 m/z

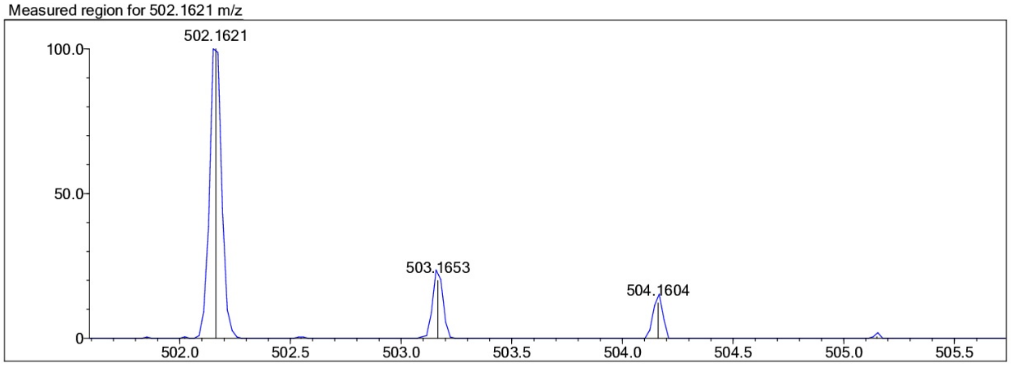

C20 H31 N5 O4 S3 [M+H]+ : Predicted region for 502.1611 m/z

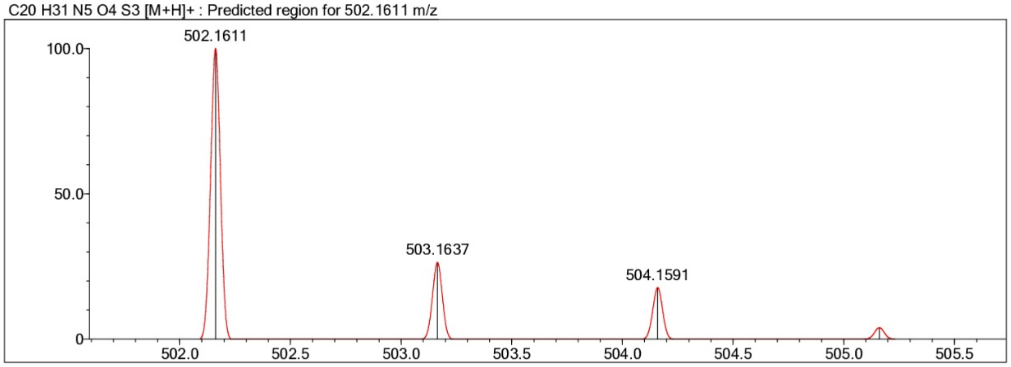

Rank	Score	Formula (M)	Ion	Meas. m/z	Pred. m/z	Df. (mDa)	Df. (ppm)	Iso	DBE
1	68.91	C20 H31 N5 O4 S3	[M+H]+	502.1621	502.1611	1.0	1.99	70.66	8.0

Figure S109. ^{1}H NMR spectrum of compound **37**

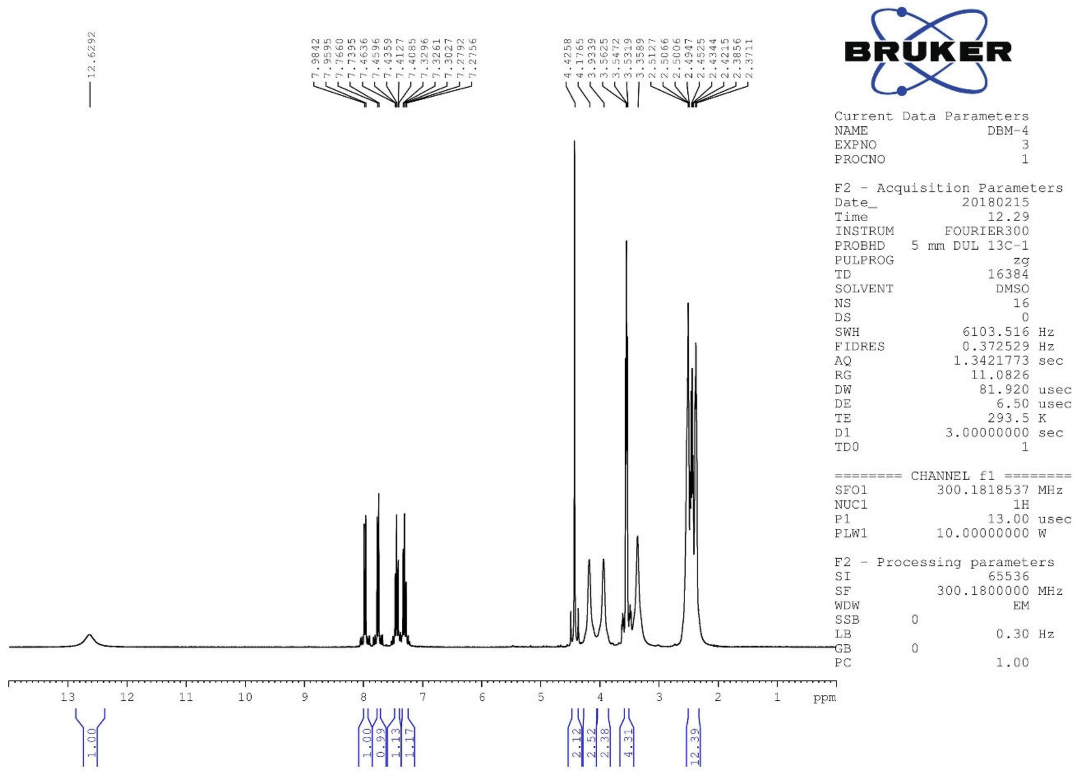

Figure S110. ^{13}C NMR spectrum of compound **37**

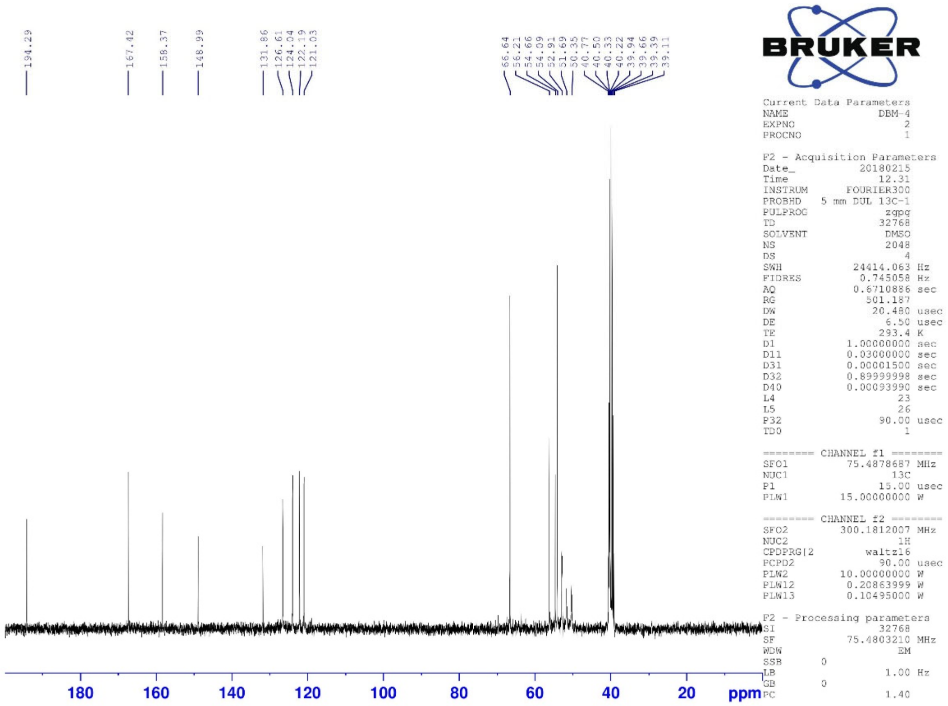

Figure S111. HRMS spectrum of compound **37**

Formula Predictor Report - DBM-4_36.lcd Page 1 of 1

Data File: C:\LabSolutions\Data\Analiz\mdaltintop\DBM-4_36.lcd

Elmt	Val.	Min	Max	Elmt	Val.	Min	Max	Elmt	Val.	Min	Max	Elmt	Val.	Min	Max	Use Adduct
H	1	5	40	O	2	0	5	S	2	0	3	Ru	2	0	0	H
C	4	0	35	F	1	0	0	Cl	1	0	0	I	3	0	0	
N	3	3	6	P	3	0	0	Br	1	0	0					

Error Margin (ppm):	5	DBE Range:	5.0 - 14.0	Electron Ions:	both
HC Ratio:	unlimited	Apply N Rule:	yes	Use MSn Info:	yes
Max Isotopes:	3	Isotope RI (%):	1.00	Isotope Res:	9000
MSn Iso RI (%):	10.00	MSn Logic Mode:	AND	Max Results:	500

Event#: 1 MS(E+) Ret. Time : 5.187 -> 5.720 Scan# : 779 -> 859

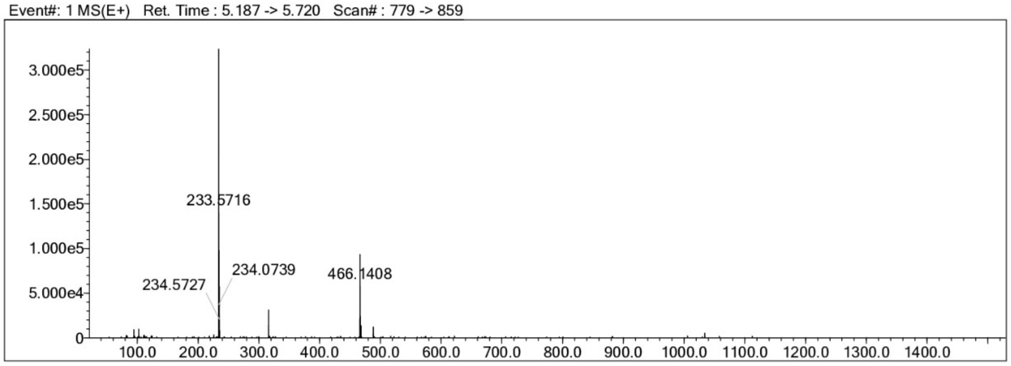

Measured region for 466.1408 m/z

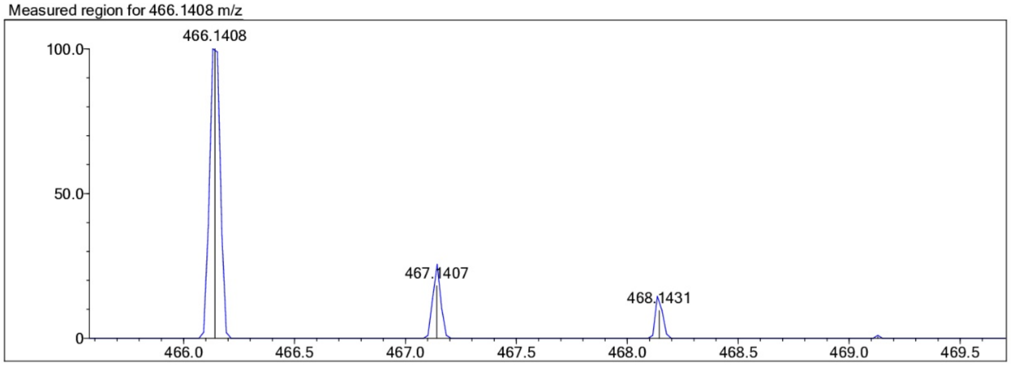

C20 H27 N5 O2 S3 [M+H]+ : Predicted region for 466.1400 m/z

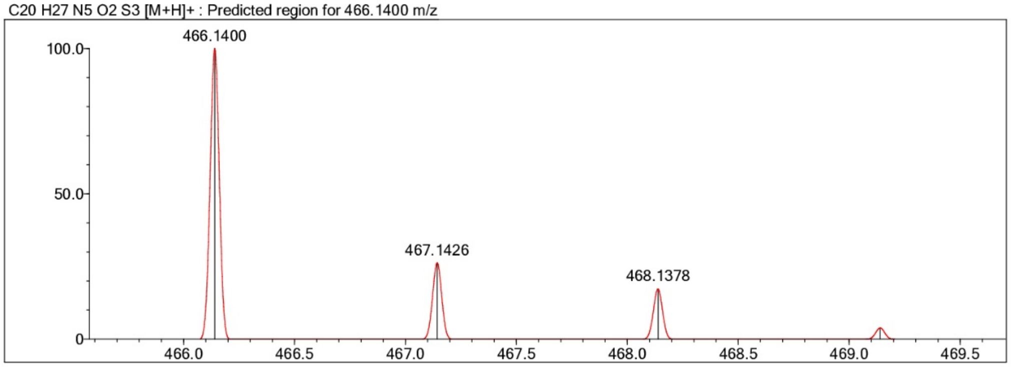

Rank	Score	Formula (M)	Ion	Meas. m/z	Pred. m/z	Df. (mDa)	Df. (ppm)	Iso	DBE
1	65.09	C20 H27 N5 O2 S3	[M+H]+	466.1408	466.1400	0.8	1.72	66.28	10.0

Figure S112. ^{1}H NMR spectrum of compound **38**

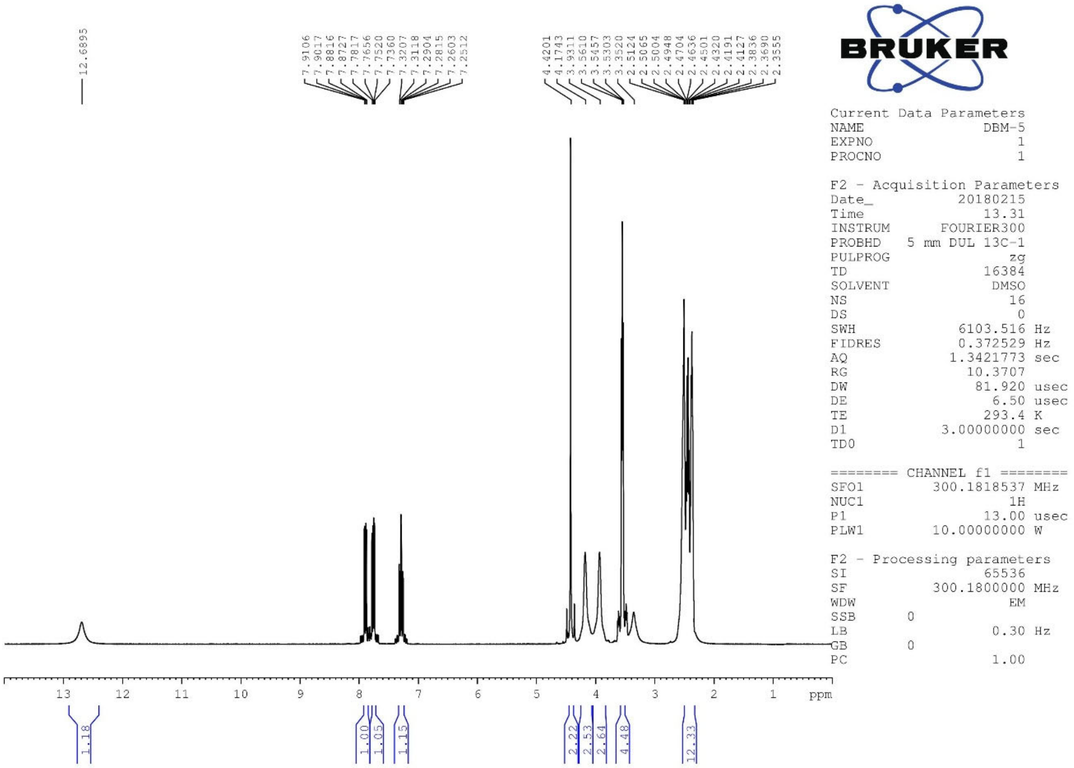

Figure S113. ^{13}C NMR spectrum of compound **38**

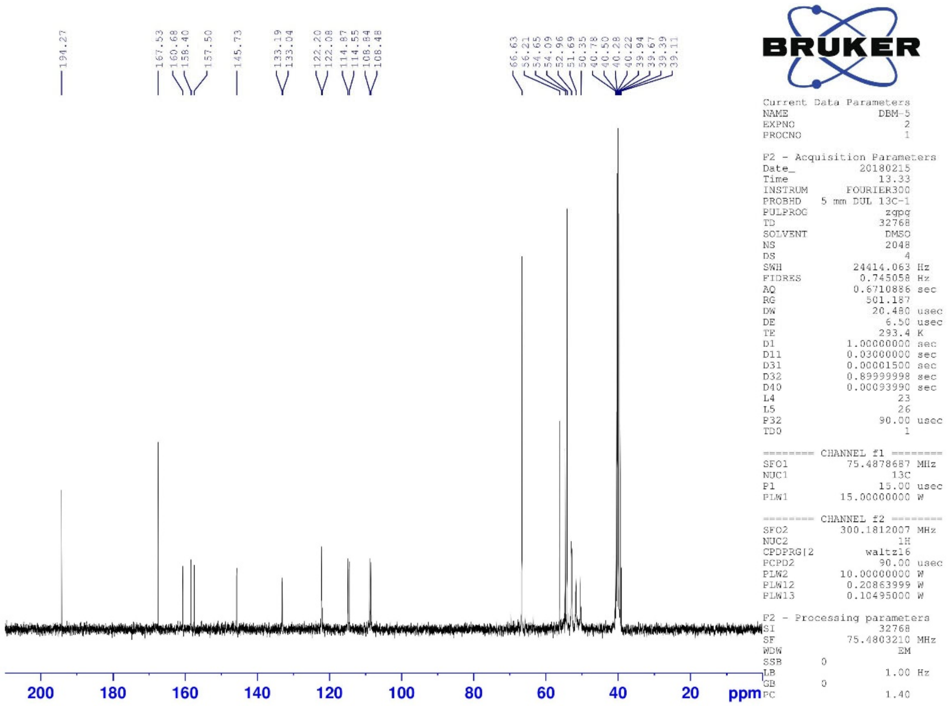

Figure S114. HRMS spectrum of compound **38**

Formula Predictor Report - DBM-5_37.lcd

Data File: C:\LabSolutions\Data\Analiz\mdaltintop\DBM-5_37.lcd

Elmt	Val.	Min	Max	Elmt	Val.	Min	Max	Elmt	Val.	Min	Max	Elmt	Val.	Min	Max	Use Adduct
H	1	5	40	O	2	0	5	S	2	0	3	Ru	2	0	0	H
C	4	0	35	F	1	0	1	Cl	1	0	0	I	3	0	0	
N	3	3	6	P	3	0	0	Br	1	0	0					

Error Margin (ppm):	5	DBE Range:	5.0 - 13.0	Electron Ions:	both
HC Ratio:	unlimited	Apply N Rule:	yes	Use MSn Info:	yes
Max Isotopes:	3	Isotope RI (%):	1.00	Isotope Res:	9000
MSn Iso RI (%):	10.00	MSn Logic Mode:	AND	Max Results:	500

Event#: 1 MS(E+) Ret. Time : 5.320 -> 6.040 Scan# : 799 -> 907

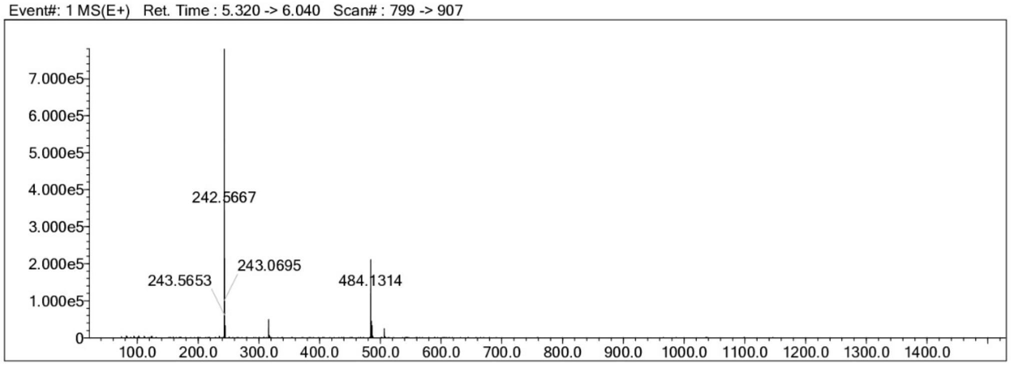

Measured region for 484.1314 m/z

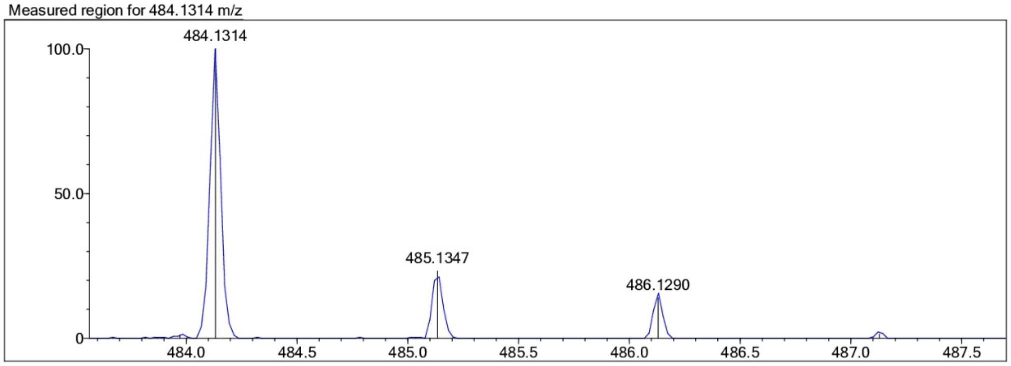

C20 H26 N5 O2 F S3 [M+H]+ : Predicted region for 484.1305 m/z

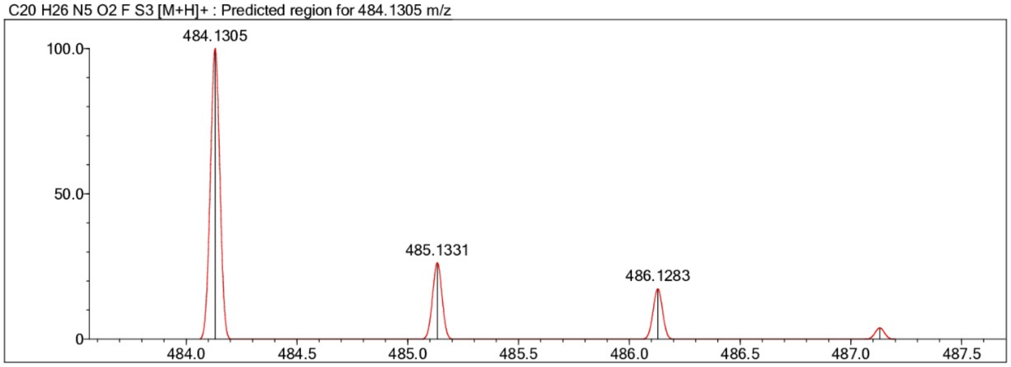

Rank	Score	Formula (M)	Ion	Meas. m/z	Pred. m/z	Df. (mDa)	Df. (ppm)	Iso	DBE
1	86.23	C20 H26 N5 O2 F S3	[M+H]+	484.1314	484.1305	0.9	1.86	88.13	10.0

Figure S115. ^{1}H NMR spectrum of compound **39**

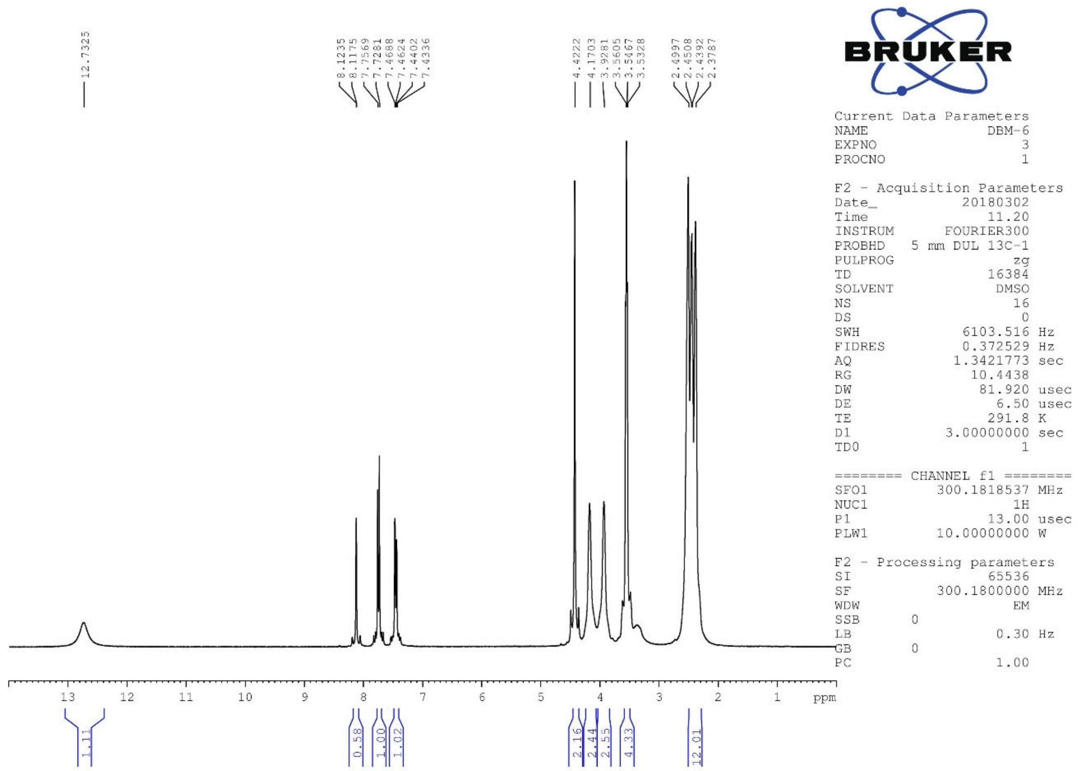

Figure S116. ^{13}C NMR spectrum of compound **39**

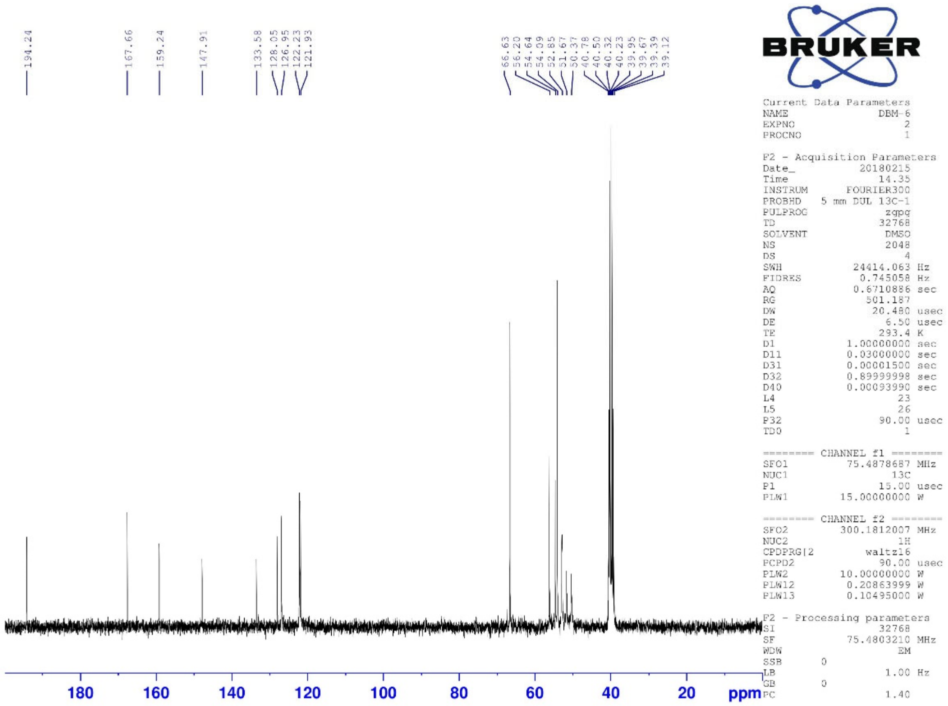

Figure S117. HRMS spectrum of compound **39**

Formula Predictor Report - DBM-6_40.lcd

Page 1 of 1

Data File: C:\LabSolutions\Data\Analiz\mdaltintop\DBM-6_40.lcd

Elmt	Val.	Min	Max	Elmt	Val.	Min	Max	Elmt	Val.	Min	Max	Elmt	Val.	Min	Max	Use Adduct
H	1	5	40	O	2	0	5	S	2	0	3	Ru	2	0	0	H
C	4	0	35	F	1	0	0	Cl	1	0	1	I	3	0	0	
N	3	3	6	P	3	0	0	Br	1	0	0					

Error Margin (ppm):	5	DBE Range:	5.0 - 10.0	Electron Ions:	both
HC Ratio:	unlimited	Apply N Rule:	yes	Use MSn Info:	yes
Max Isotopes:	3	Isotope RI (%):	1.00	Isotope Res:	9000
MSn Iso RI (%):	10.00	MSn Logic Mode:	AND	Max Results:	500

Event#: 1 MS(E+) Ret. Time : 5.667 -> 6.053 Scan# : 851 -> 909

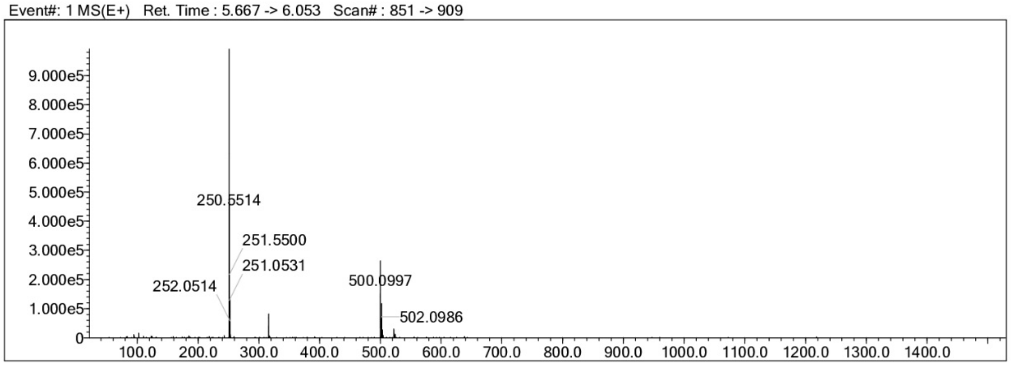

Measured region for 500.0997 m/z

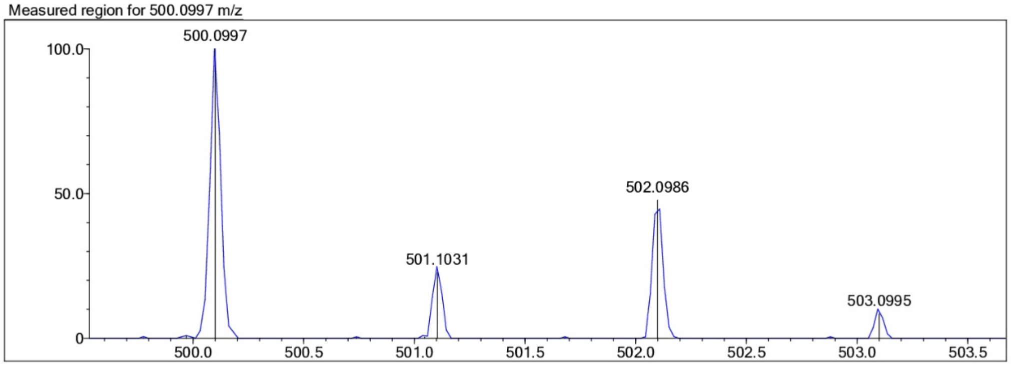

C20 H26 N5 O2 S3 Cl [M+H]+ : Predicted region for 500.1010 m/z

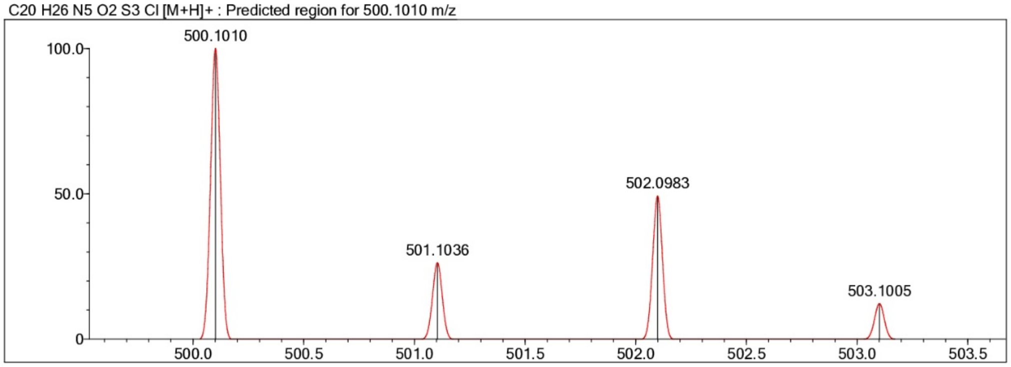

Rank	Score	Formula (M)	Ion	Meas. m/z	Pred. m/z	Df. (mDa)	Df. (ppm)	Iso	DBE
1	76.91	C20 H26 N5 O2 S3 Cl	[M+H]+	500.0997	500.1010	-1.3	-2.60	80.11	10.0

Figure S118. ^{1}H NMR spectrum of compound **40**

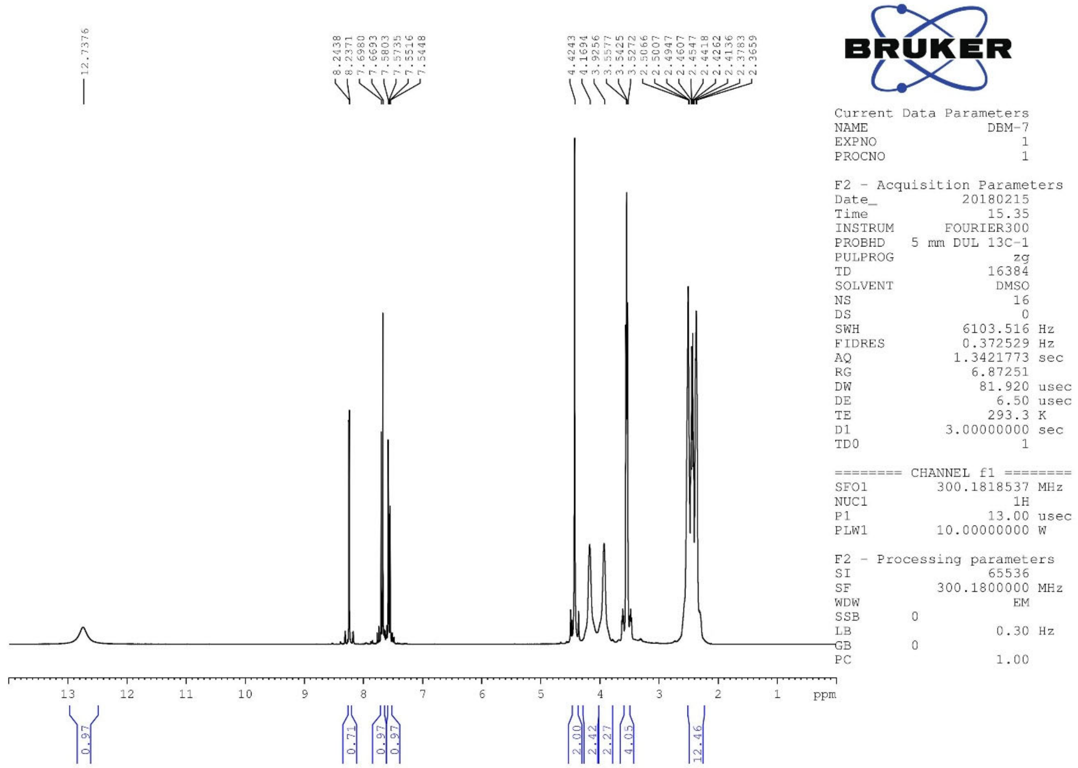

Figure S119. ^{13}C NMR spectrum of compound **40**

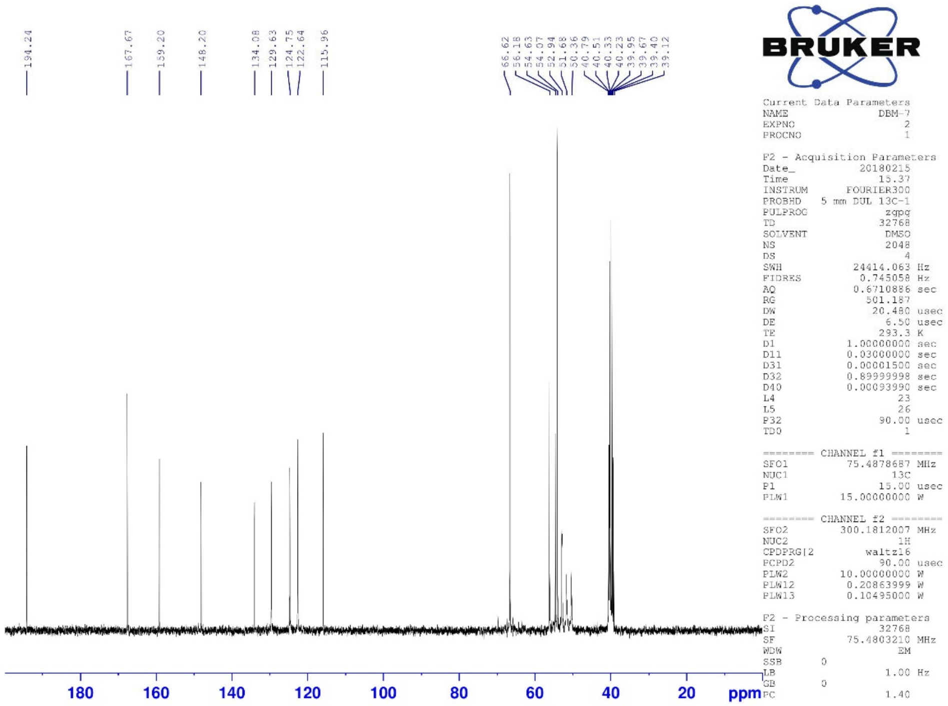

Figure S120. HRMS spectrum of compound **40**

Formula Predictor Report - DBM-7_41.lcd　　Page 1 of 1

Data File: C:\LabSolutions\Data\Analiz\mdaltintop\DBM-7_41.lcd

Elmt	Val.	Min	Max	Elmt	Val.	Min	Max	Elmt	Val.	Min	Max	Elmt	Val.	Min	Max	Use Adduct
H	1	5	40	O	2	0	5	S	2	0	3	Ru	2	0	0	H
C	4	0	35	F	1	0	0	Cl	1	0	0	I	3	0	0	
N	3	3	6	P	3	0	0	Br	1	0	1					

Error Margin (ppm):	5	DBE Range:	5.0 - 10.0	Electron Ions:	both
HC Ratio:	unlimited	Apply N Rule:	yes	Use MSn Info:	yes
Max Isotopes:	3	Isotope RI (%):	1.00	Isotope Res:	9000
MSn Iso RI (%):	10.00	MSn Logic Mode:	AND	Max Results:	500

Event#: 1 MS(E+)　Ret. Time : 5.667 -> 6.187　Scan# : 851 -> 929

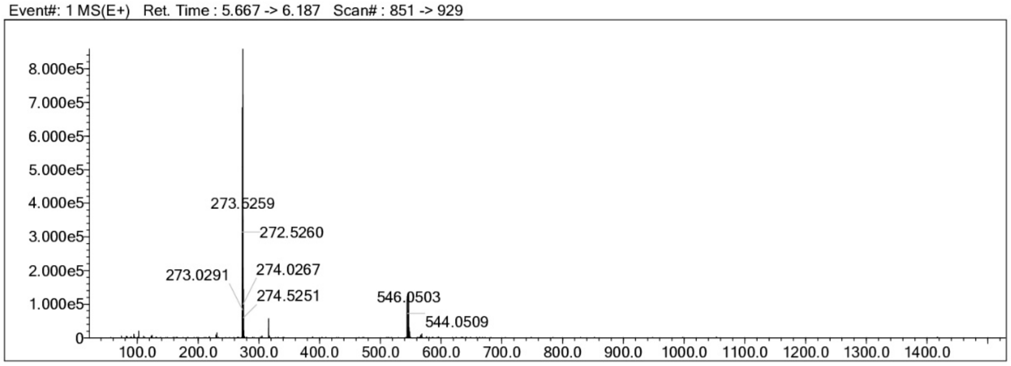

Measured region for 544.0509 m/z

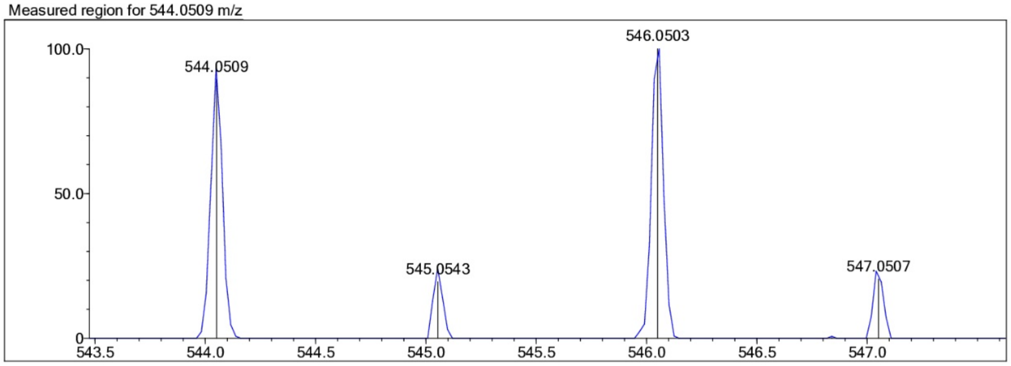

C20 H26 N5 O2 S3 Br [M+H]+ : Predicted region for 544.0505 m/z

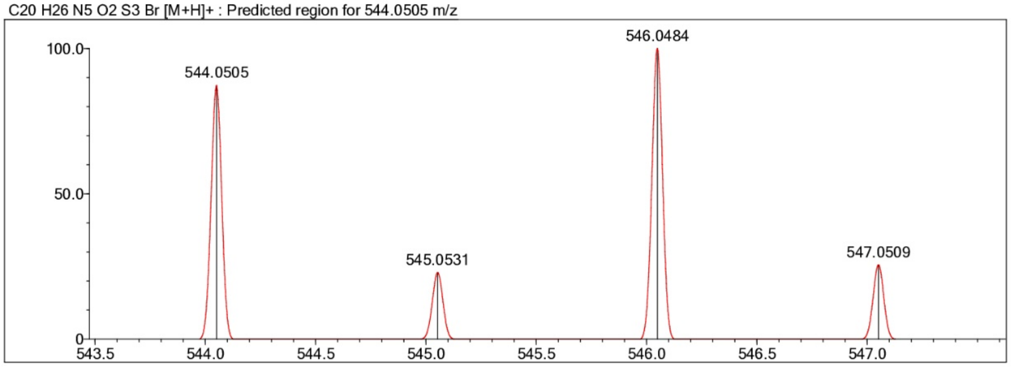

Rank	Score	Formula (M)	Ion	Meas. m/z	Pred. m/z	Df. (mDa)	Df. (ppm)	Iso	DBE
1	80.14	C20 H26 N5 O2 S3 Br	[M+H]+	544.0509	544.0505	0.4	0.74	80.14	10.0

Figure S121. ^{1}H NMR spectrum of compound **41**

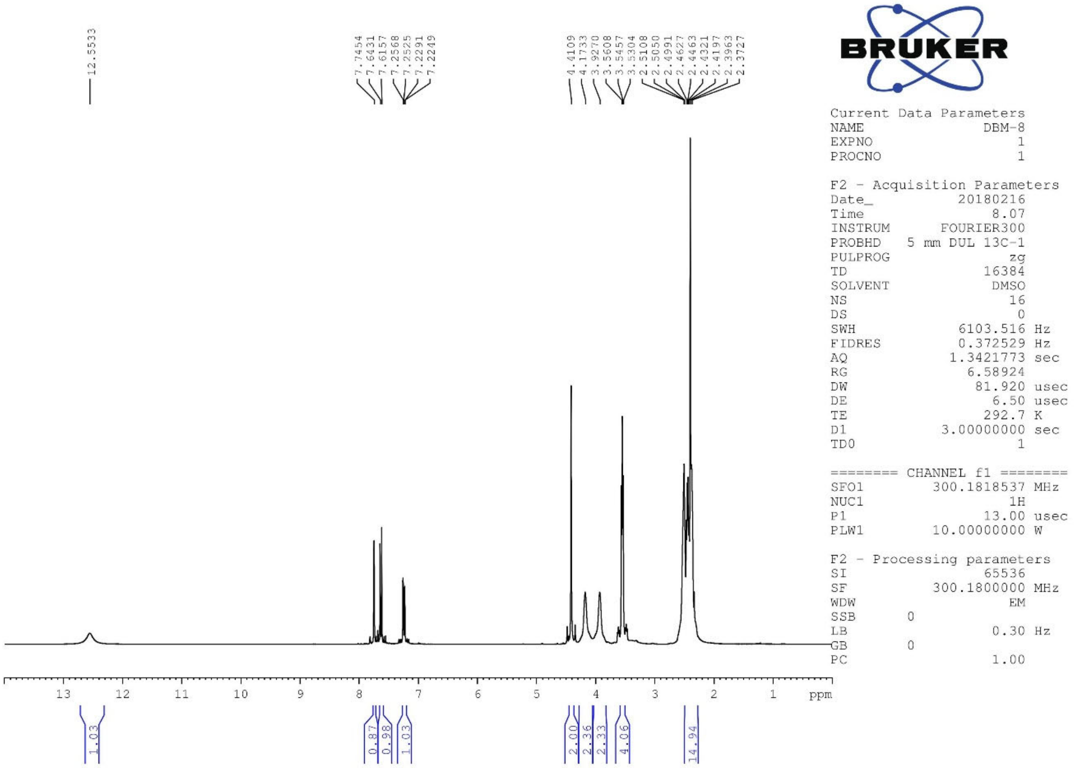

Figure S122. ^{13}C NMR spectrum of compound **41**

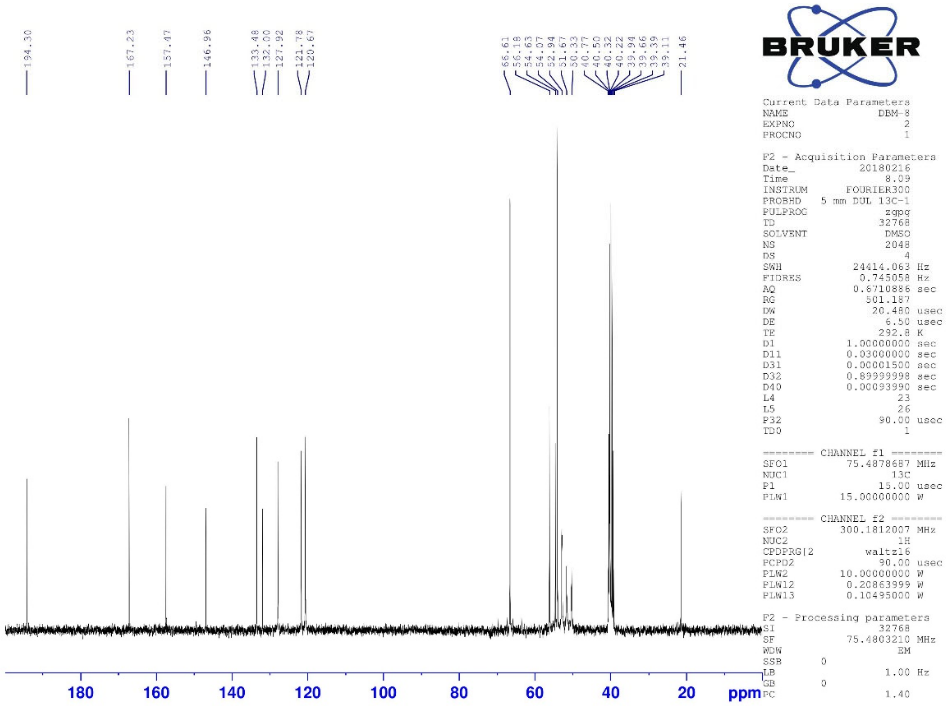

Figure S123. HRMS spectrum of compound **41**

Formula Predictor Report - DBM-8_42.lcd Page 1 of 1

Data File: C:\LabSolutions\Data\Analiz\mdaltintop\DBM-8_42.lcd

Elmt	Val.	Min	Max	Elmt	Val.	Min	Max	Elmt	Val.	Min	Max	Elmt	Val.	Min	Max	Use Adduct
H	1	5	40	O	2	0	5	S	2	0	3	Ru	2	0	0	H
C	4	0	35	F	1	0	0	Cl	1	0	0	I	3	0	0	
N	3	3	6	P	3	0	0	Br	1	0	1					

Error Margin (ppm):	5	DBE Range:	5.0 - 10.0	Electron Ions:	both
HC Ratio:	unlimited	Apply N Rule:	yes	Use MSn Info:	yes
Max Isotopes:	3	Isotope RI (%):	1.00	Isotope Res:	9000
MSn Iso RI (%):	10.00	MSn Logic Mode:	AND	Max Results:	500

Event#: 1 MS(E+) Ret. Time : 5.347 -> 6.200 Scan# : 803 -> 931

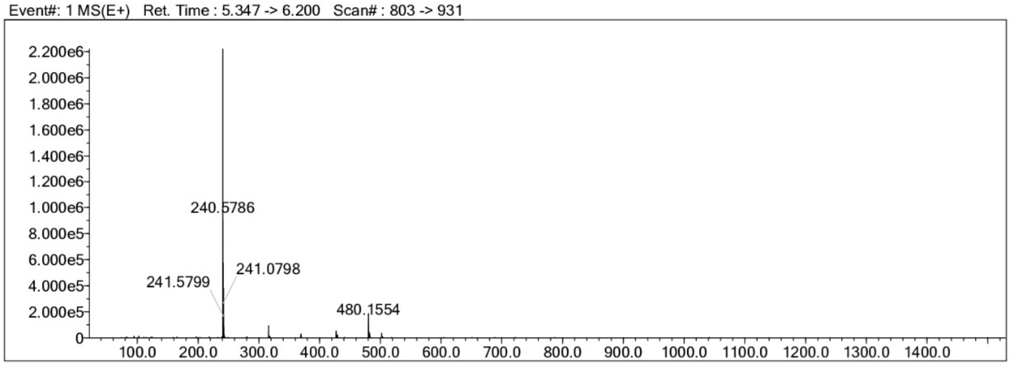

Measured region for 480.1554 m/z

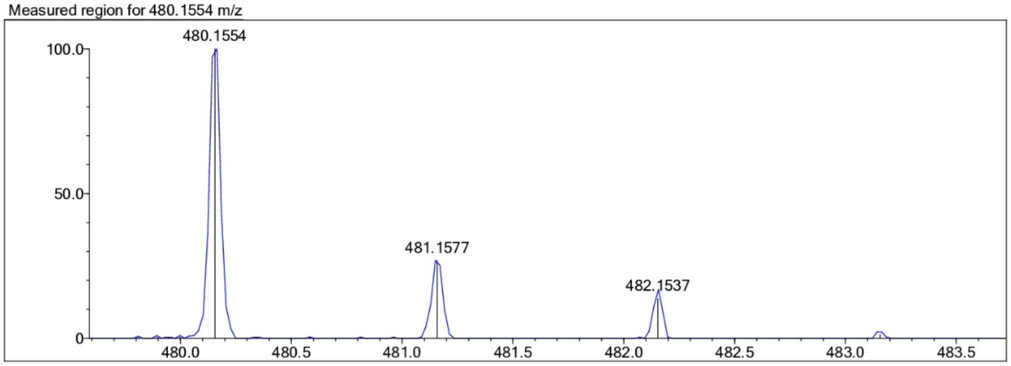

C21 H29 N5 O2 S3 [M+H]+ : Predicted region for 480.1556 m/z

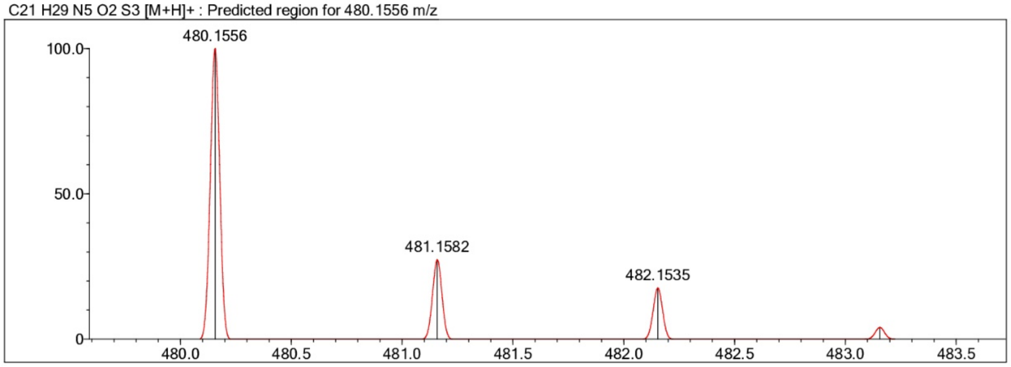

Rank	Score	Formula (M)	Ion	Meas. m/z	Pred. m/z	Df. (mDa)	Df. (ppm)	Iso	DBE
1	98.76	C21 H29 N5 O2 S3	[M+H]+	480.1554	480.1556	-0.2	-0.42	98.76	10.0

Figure S124. ^{1}H NMR spectrum of compound **42**

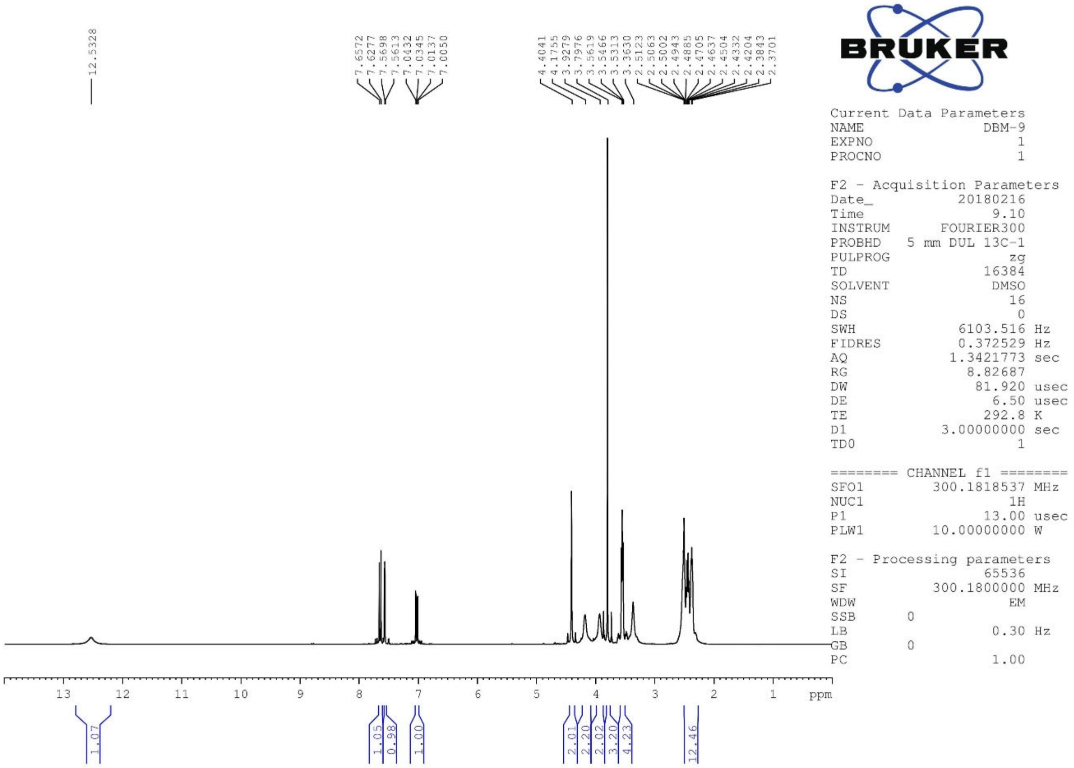

Figure S125. ^{13}C NMR spectrum of compound **42**

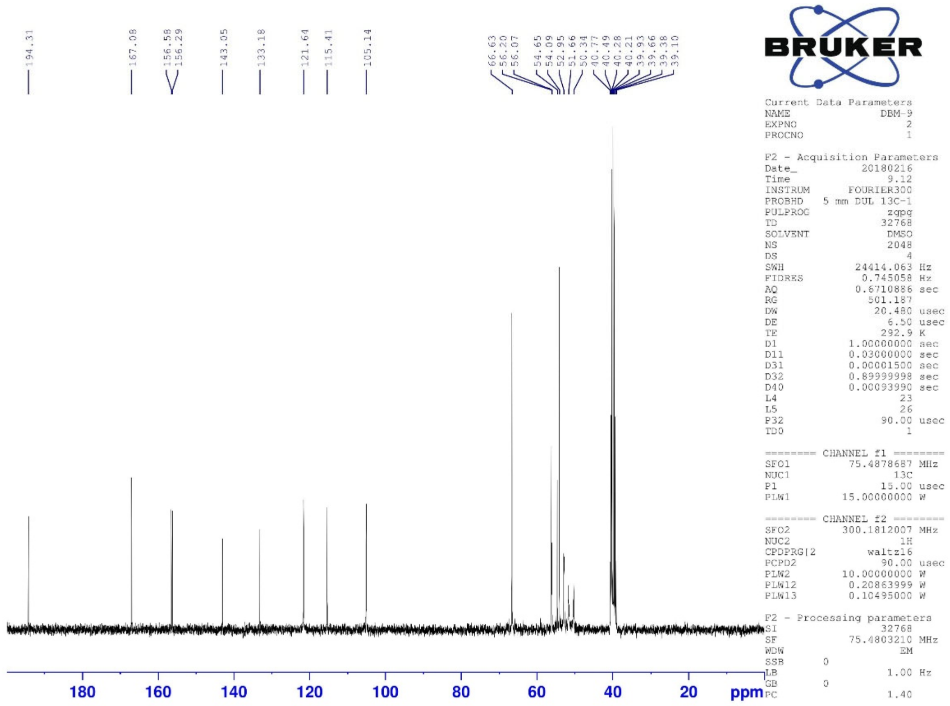

Figure S126. HRMS spectrum of compound **42**

Formula Predictor Report - DBM-9_43.lcd

Page 1 of 1

Data File: C:\LabSolutions\Data\Analiz\mdaltintop\DBM-9_43.lcd

Elmt	Val.	Min	Max	Elmt	Val.	Min	Max	Elmt	Val.	Min	Max	Elmt	Val.	Min	Max	Use Adduct
H	1	5	40	O	2	0	5	S	2	0	3	Ru	2	0	0	H
C	4	0	35	F	1	0	0	Cl	1	0	0	I	3	0	0	
N	3	3	6	P	3	0	0	Br	1	0	1					

Error Margin (ppm):	5	DBE Range:	5.0 - 10.0	Electron Ions:	both
HC Ratio:	unlimited	Apply N Rule:	yes	Use MSn Info:	yes
Max Isotopes:	3	Isotope RI (%):	1.00	Isotope Res:	9000
MSn Iso RI (%):	10.00	MSn Logic Mode:	AND	Max Results:	500

Event#: 1 MS(E+) Ret. Time : 5.040 -> 5.760 Scan# : 757 -> 865

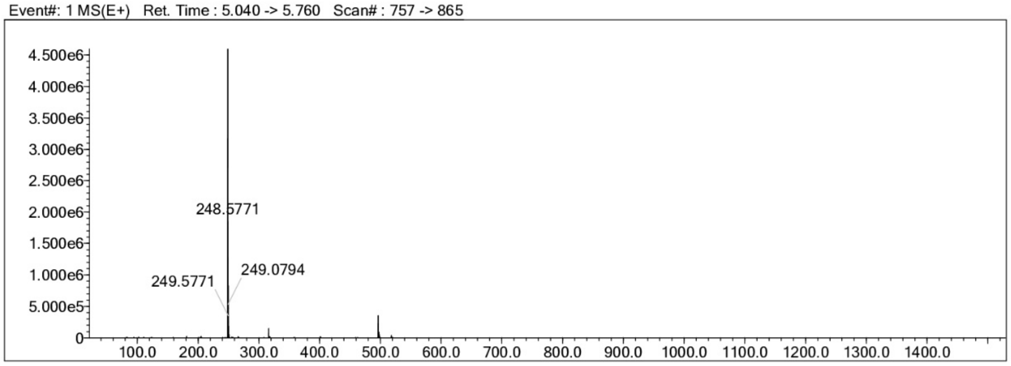

Measured region for 496.1502 m/z

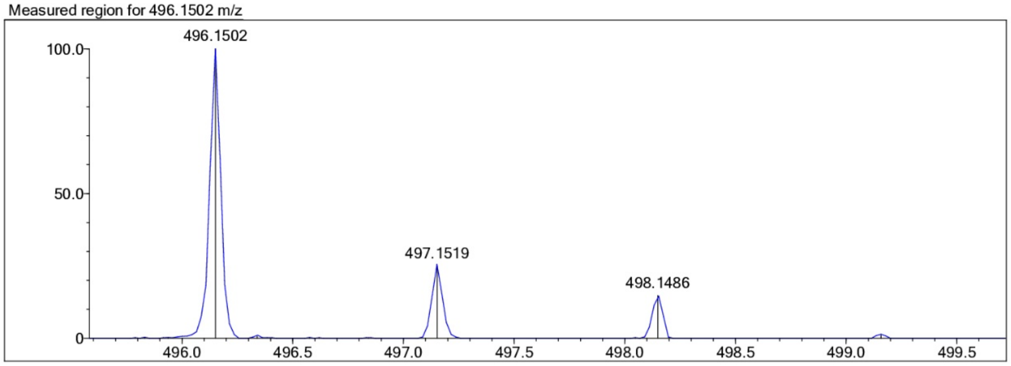

C21 H29 N5 O3 S3 [M+H]+ : Predicted region for 496.1505 m/z

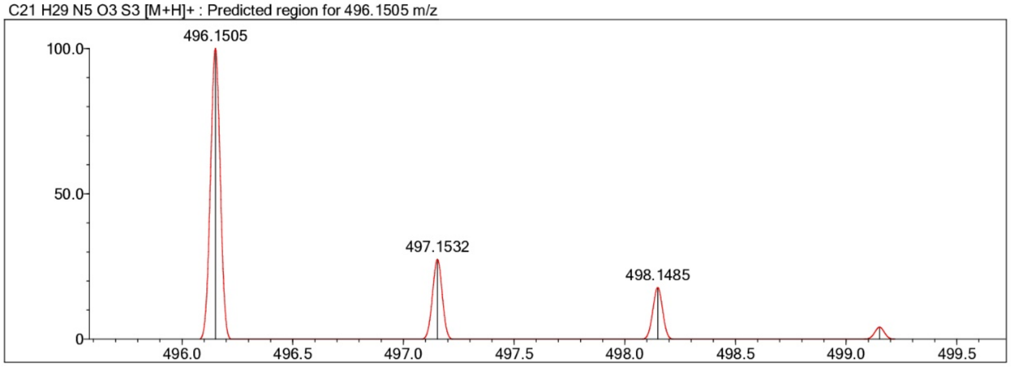

Rank	Score	Formula (M)	Ion	Meas. m/z	Pred. m/z	Df. (mDa)	Df. (ppm)	Iso	DBE
1	80.20	C21 H29 N5 O3 S3	[M+H]+	496.1502	496.1505	-0.3	-0.60	80.20	10.0

Figure S127. ^{1}H NMR spectrum of compound **43**

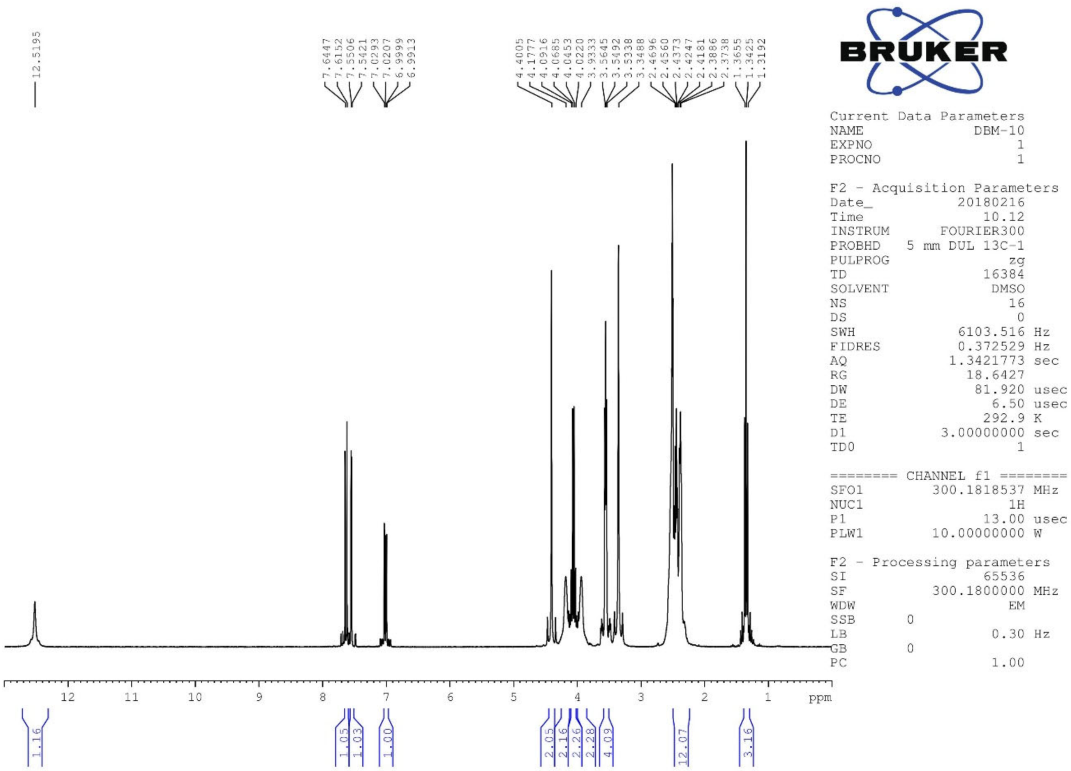

Figure S128. ^{13}C NMR spectrum of compound **43**

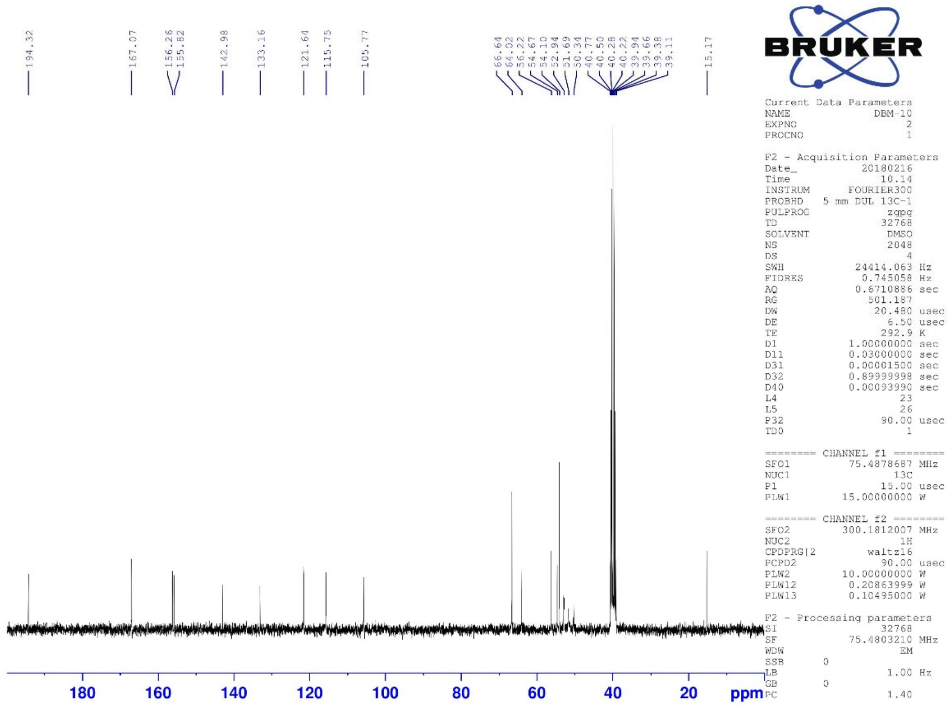

Figure S129. HRMS spectrum of compound **43**

Formula Predictor Report - DBM-10_44.lcd

Data File: C:\LabSolutions\Data\Analiz\mdaltintop\DBM-10_44.lcd

Elmt	Val.	Min	Max	Elmt	Val.	Min	Max	Elmt	Val.	Min	Max	Elmt	Val.	Min	Max	Use Adduct
H	1	5	40	O	2	0	5	S	2	0	3	Ru	2	0	0	H
C	4	0	35	F	1	0	0	Cl	1	0	0	I	3	0	0	
N	3	3	6	P	3	0	0	Br	1	0	1					

Error Margin (ppm): 5
HC Ratio: unlimited
Max Isotopes: 3
MSn Iso RI (%): 10.00

DBE Range: 5.0 - 10.0
Apply N Rule: yes
Isotope RI (%): 1.00
MSn Logic Mode: AND

Electron Ions: both
Use MSn Info: yes
Isotope Res: 9000
Max Results: 500

Event#: 1 MS(E+) Ret. Time : 5.440 -> 6.000 Scan# : 817 -> 901

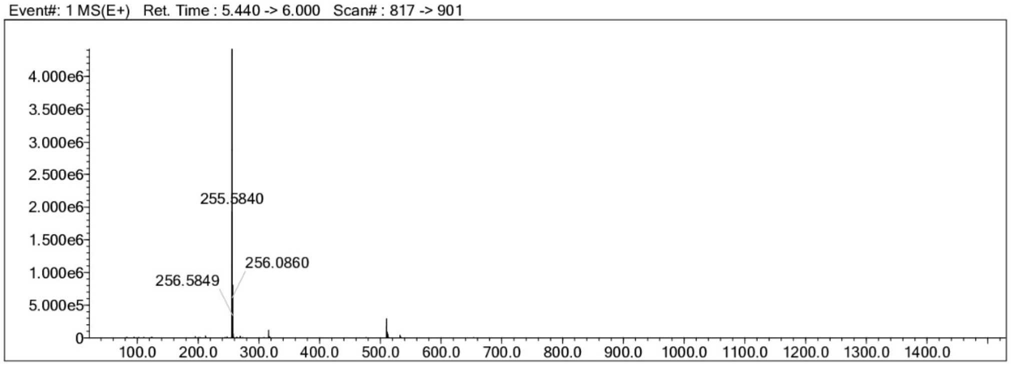

Measured region for 510.1677 m/z

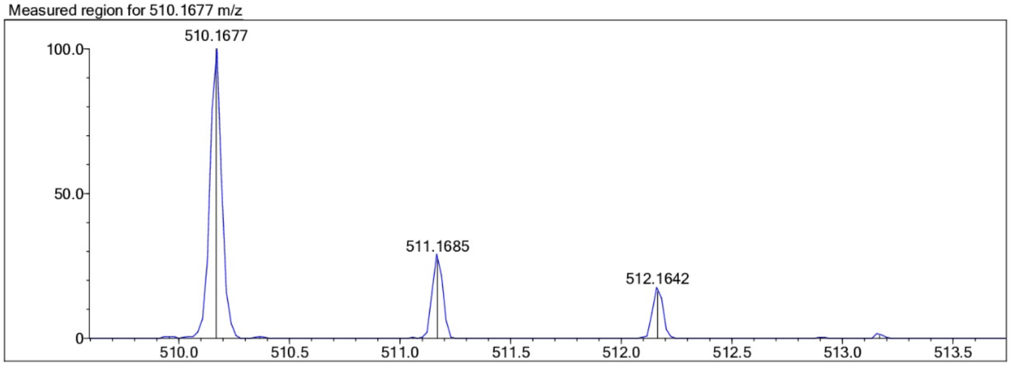

C22 H31 N5 O3 S3 [M+H]+ : Predicted region for 510.1662 m/z

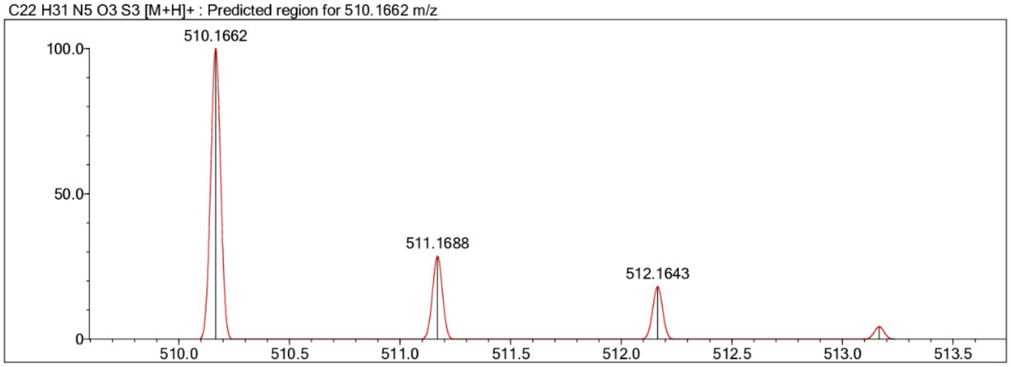

Rank	Score	Formula (M)	Ion	Meas. m/z	Pred. m/z	Df. (mDa)	Df. (ppm)	Iso	DBE
1	78.22	C22 H31 N5 O3 S3	[M+H]+	510.1677	510.1662	1.5	2.94	82.21	10.0

Figure S130. ^{1}H NMR spectrum of compound **44**

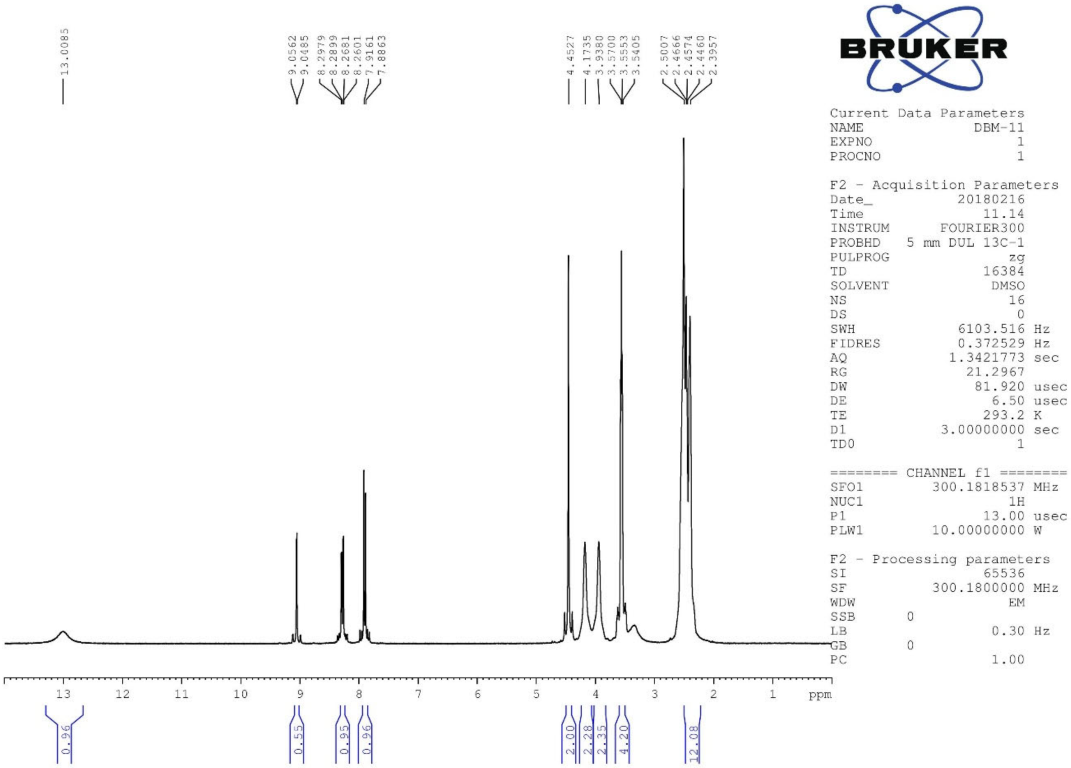

Figure S131. ^{13}C NMR spectrum of compound **44**

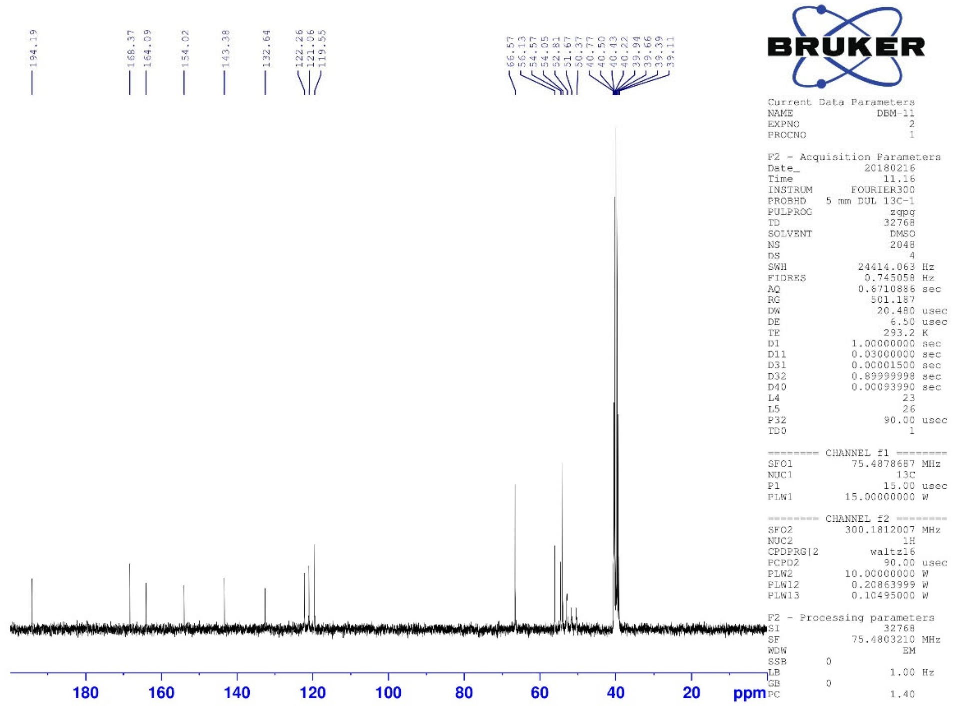

Figure S132. HRMS spectrum of compound **44**

Formula Predictor Report - DBM-11_38.lcd Page 1 of 1

Data File: C:\LabSolutions\Data\Analiz\mdaltintop\DBM-11_38.lcd

Elmt	Val.	Min	Max	Elmt	Val.	Min	Max	Elmt	Val.	Min	Max	Elmt	Val.	Min	Max	Use Adduct
H	1	5	40	O	2	0	5	S	2	0	3	Ru	2	0	0	H
C	4	0	35	F	1	0	0	Cl	1	0	0	I	3	0	0	
N	3	3	6	P	3	0	0	Br	1	0	0					

Error Margin (ppm): 5
HC Ratio: unlimited
Max Isotopes: 3
MSn Iso RI (%): 10.00

DBE Range: 5.0 - 13.0
Apply N Rule: yes
Isotope RI (%): 1.00
MSn Logic Mode: AND

Electron Ions: both
Use MSn Info: yes
Isotope Res: 9000
Max Results: 500

Event#: 1 MS(E+) Ret. Time : 5.267 -> 5.787 Scan# : 791 -> 869

Measured region for 511.1252 m/z

C20 H26 N6 O4 S3 [M+H]+ : Predicted region for 511.1250 m/z

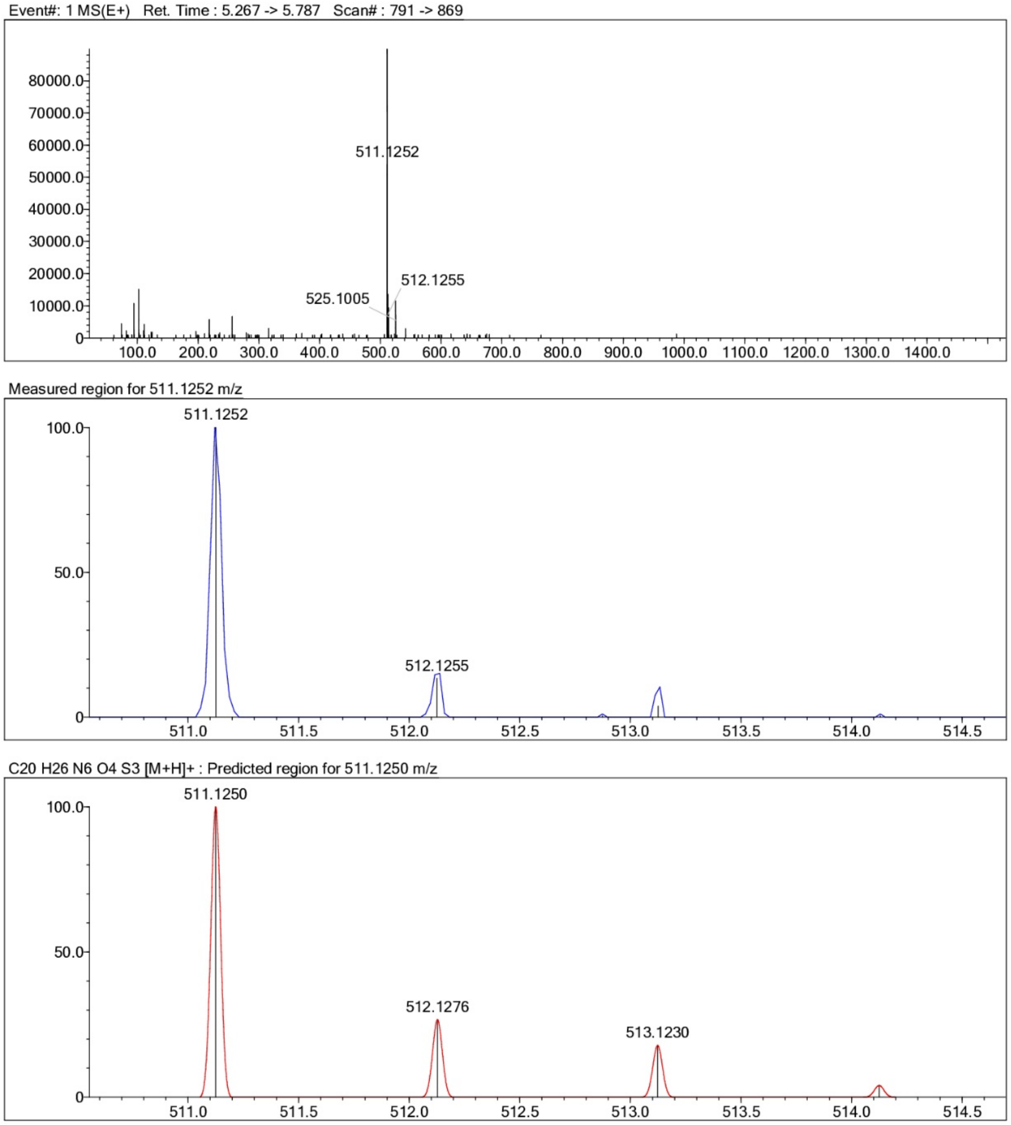

Rank	Score	Formula (M)	Ion	Meas. m/z	Pred. m/z	Df. (mDa)	Df. (ppm)	Iso	DBE
1	59.49	C20 H26 N6 O4 S3	[M+H]+	511.1252	511.1250	0.2	0.39	59.49	11.0

Figure S133. Docking poses of compound **14** (**A**), compound **20** (**B**), compound **21** (**C**), compound **36** (**D**), compound **39** (**E**), and AAZ (**F**) within the catalytic domain of hCA IX (PDB ID: 3IAI)

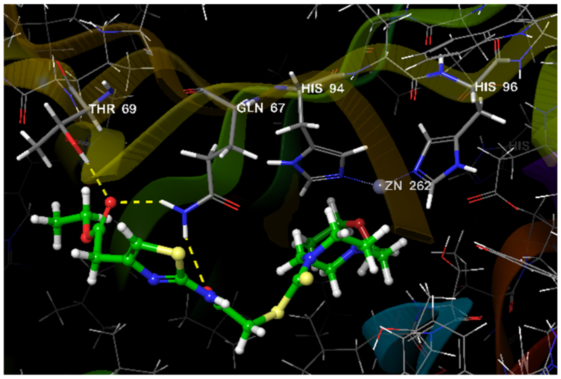

A

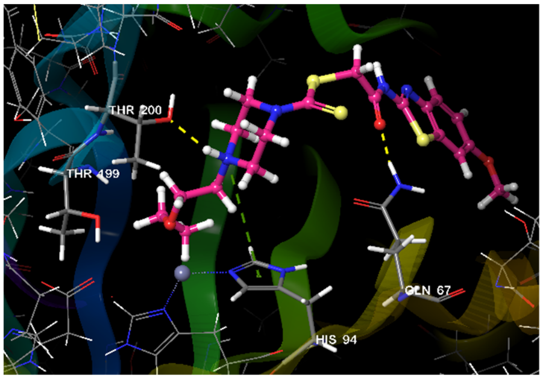

B

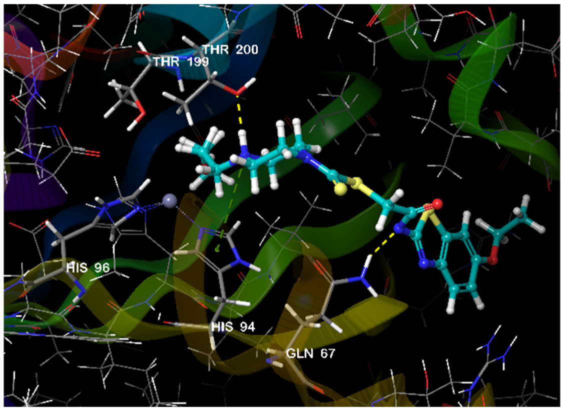

C

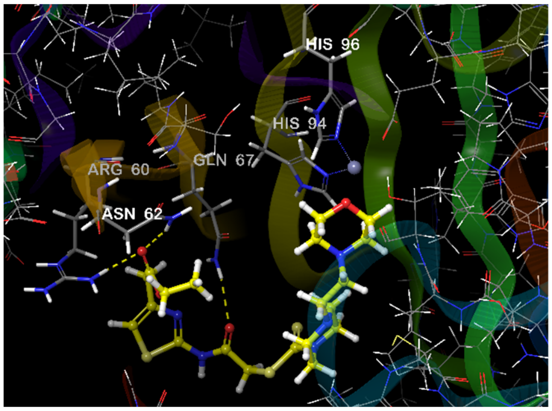

D

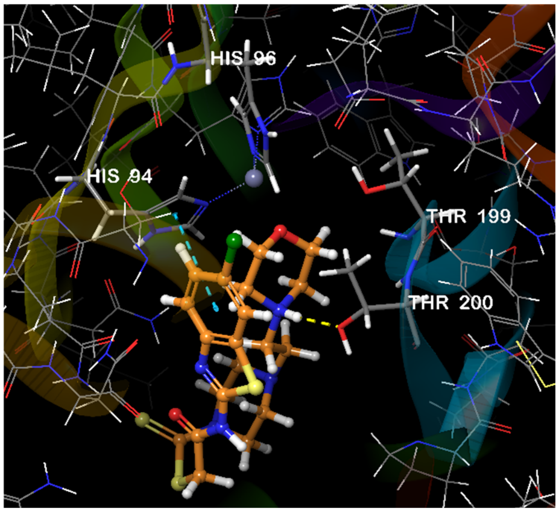

E

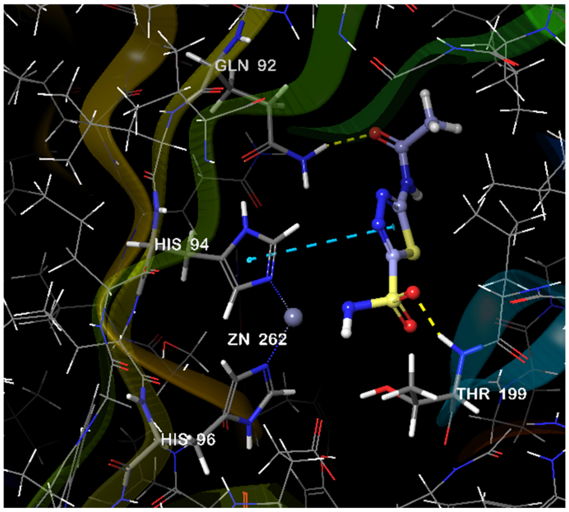

F

Figure S134. Docking poses of compound **4** (**A**), compound **9** (**B**), compound **15** (**C**), compound **16** (**D**), compound **17** (**E**), compound **18** (**F**), compound **20** (**G**), compound **29** (**H**), compound **30** (**I**), and AAZ (**J**) in the catalytic domain of hCA XII (PDB ID: 1JD0)

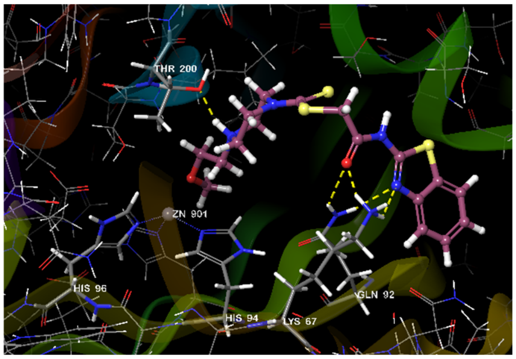

A

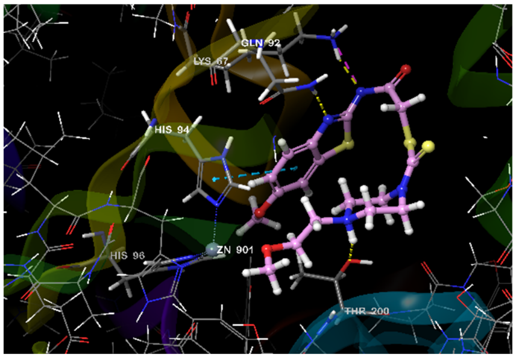

B

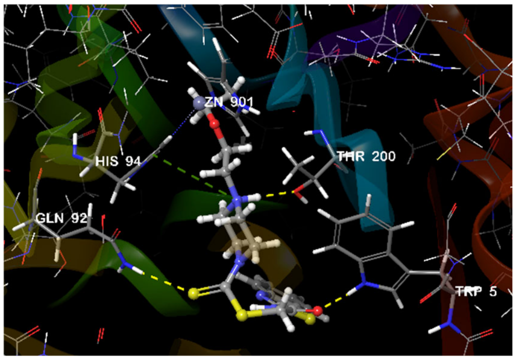

C

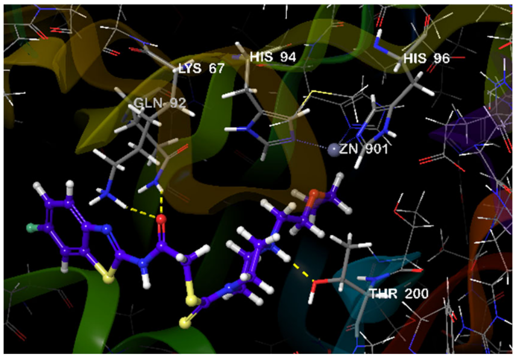

D

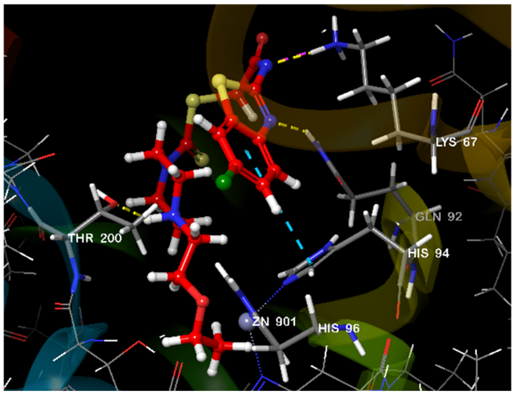

E

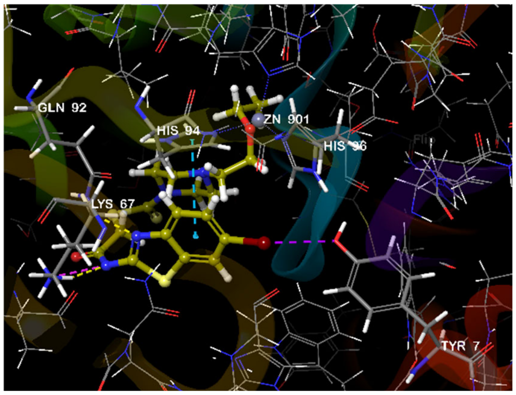

F

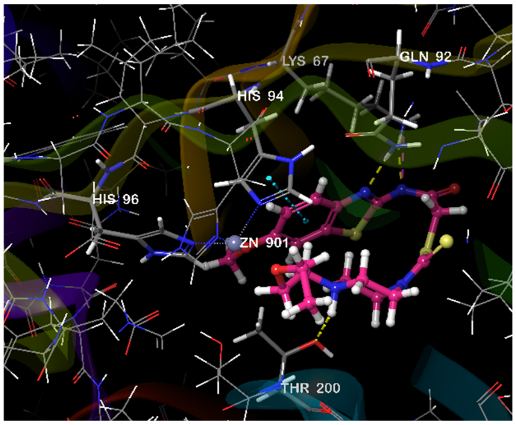
LYS 67
GLN 92
HIS 94
HIS 96
ZN 901
THR 200

G

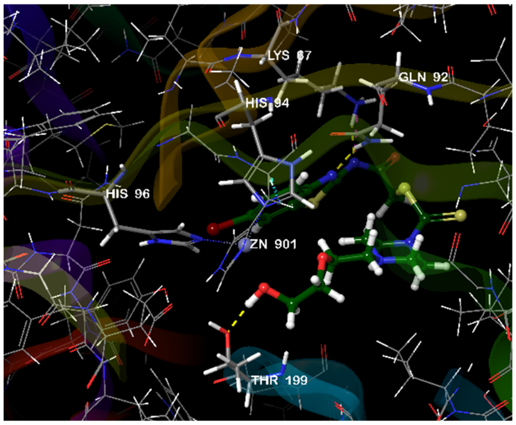
LYS 67
GLN 92
HIS 94
HIS 96
ZN 901
THR 199

H

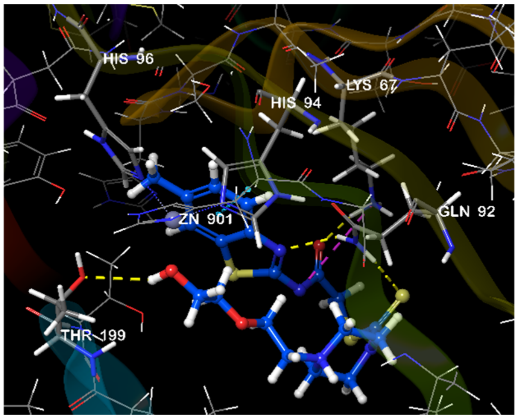
HIS 96
LYS 67
HIS 94
ZN 901
GLN 92
THR 199

I

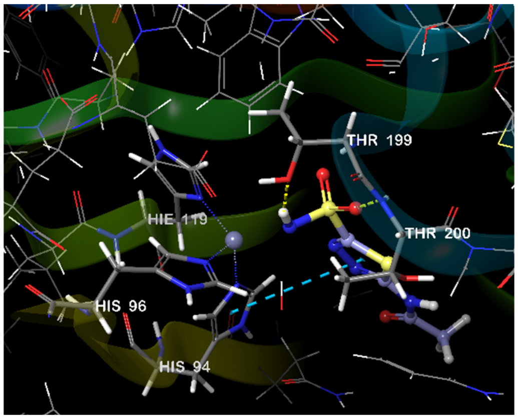
THR 199
HIE 119
THR 200
HIS 96
HIS 94

J
